# Supplementary material for: Communication and shared decision-making with patients with limited health literacy; helpful strategies, barriers and suggestions for improvement reported by hospital-based palliative care providers
Source: PLoS One. 2020 Jun 19;15(6):e0234926. doi: 10.1371/journal.pone.0234926 (PMC7304585; doi:10.1371/journal.pone.0234926)
Supplement: S1 Appendix — (DOCX) [file pone.0234926.s001.docx]

**Appendix 1.** Visual overview of the project ‘A Basic Understanding’
