# Supplementary material for: Communication and shared decision-making with patients with limited health literacy; helpful strategies, barriers and suggestions for improvement reported by hospital-based palliative care providers
Source: PLoS One. 2020 Jun 19;15(6):e0234926. doi: 10.1371/journal.pone.0234926 (PMC7304585; doi:10.1371/journal.pone.0234926)
Supplement: S2 Appendix — (DOCX) [file pone.0234926.s002.docx]

**Appendix 2.** Interview topic list

Interview topic list care providers / organization employees within palliative team

Subjects 1,2,3 and 4 for caregivers

Subject 4 for administrative employees within the hospital or palliative team

- Introduction
  - Welcome
- *Thanks in advance for your valuable time! We really need the insights of medical professionals like you to conduct our research. Ultimately, we want the same as you and your colleagues; the very best for patients.*
  - Introduction project
- *Today I would like to ask you some general questions about your role as a care provider in palliative care, about communication, about your patients and about the way your hospital is organized.*
  - Recording on audio
- *With your permission, I would like to record the conversation. This makes it possible for me to transcribe the interview. You will remain anonymous. Are you okay with that?*
  - Signing consent form
- *Before we start, could you perhaps sign for your permission?*
  - Do you have any questions so far?

1. Your role as a healthcare provider for patients in palliative care
   1. What is your role in the care of patients (and their loved ones) in the palliative phase?
   2. Is there always one designated central healthcare provider for the patient?
      1. Is it clear to the patient who that is?
   3. How do you collaborate with other healthcare providers within your team / hospital?
   4. How do you collaborate with the general practitioner?
   5. Do you collaborate with social workers? (e.g. a pastoral worker, imam, with informal caregivers or with volunteers?)
2. Communicating, informing and shared decision-making

*Within palliative care, attention for the physical, psychological, social and spiritual well-being of the patient is important.*

- 1. To what extent are these different aspects addressed in your conversations with the patient?
  2. Do you bring up these aspects yourself?
     1. With all patients?
     2. Also, for people with a low level of education, or who don't seem to understand much? If yes, which aspects?
  3. How do you ask about these aspects?
     1. In which phase of the consultation or supervision?
     2. Do you ask for it every time?
  4. Do you work with an individual care plan?
  5. Are agreements on all these aspects (physical, psychological, social and spiritual well-being) recorded in an individual care plan?
     1. Which is and which is not?

*Good communication is the basis for good care, so that the patient is well informed and there can be shared decision-making.*

- 1. Are there patients for whom you find communication more difficult? (e.g. if they have a low level of education, or appear to come from a low social class, or have a migration background?)
  2. In what way is communication more difficult for these people?
  3. What do you do to improve communication?
  4. Are there moments when you consciously adjust your communication and how?
     1. Can you give an example?
  5. Do you ever check what your patients have understood everything?
  6. Do you discuss fewer things with these people compared with others?
  7. Do you use videos or visual information materials?
  8. To what extent do you think there is shared decision-making with patients in the palliative phase?
     1. Is this the same for all patients?
  9. Do you think it would be better if more shared decision-making took place?
  10. Can you give an example of a situation where there a clear case of shared decision-making?
  11. Can you give an example of a situation in which shared decision-making did not happen?
      1. What does that mean for healthcare, for the patient and for you?
  12. If you think it shared decision-making could be better practiced, what would it take to improve this?

*The interviewer puts paper cards on the table, with important themes in patient care in the palliative phase written on them; philosophy of life and cultural background, treatment and non-treatment agreements, hospital admissions, place of care and death, crisis situations, legal representation, and end-of-life decisions. After putting the cards on the table, the interviewer continues the interview.*

- 1. Can you indicate to what extent these topics are covered during your consultations with the help of a ranking in the cards? On the left you can lay the cards that are the most dealt with in your consultations, the right on the cards that are the least dealt with.
  2. If not (the cards on the right-hand side), do you think this will be discussed with another healthcare provider (within your team or with the doctor, for example)?
     1. To what extent does shared decision-making take place?

1. Limited health literacy (LHL) (in Dutch ‘beperkte gezondheidsvaardigheden’).
   1. Have you ever heard of the concept of LHL?
      1. What do you think that means?

*The interviewer introduces the HCP with a definition/meaning of LHL:*

*People's skills to obtain, understand and apply information on health and healthcare when making health decisions.* ***Functional*** *(reading and writing, math, etc.),* ***interactive / communicative*** *(reading comprehension, separating main from side issues, asking questions, etc.) and* ***critical assessment*** *(applying information, ordering/organizing information, thinking ahead, setting priorities, etc.).*

- 1. How often do you deal with patients with LHL?
  2. How do you recognize patients with LHL?
  3. In what way do you try to take them into account in your oral and/or written communication?
  4. Do you experience barriers in the care of patients with LHL?
     1. If so, which barriers?
     2. Do you also experience these barriers with other patients?
  5. What do you need to ensure that care is better aligned for patients with LHL?

1. (if applicable) Your hospital/organization
   1. Is there policy within your hospital to make healthcare more accessible for patients with LHL?
   2. Are certain methods or instruments used within your organization to make healthcare more accessible for people with LHL?
   3. What do you think is important for your hospital to make healthcare for people with LHL more accessible?
   4. What are possibilities for this (for example, adjusting patient records or schedule additional consultations)?
   5. What barriers do you perceive at the organizational level?
   6. Would you want specific policy on this?
      1. Why or why not?
   7. Who (care providers, the organization within your own hospital or outside your own hospital) should play a role in this?
   8. And at what level within the hospital do you think this should be visible (in the general hospital policy, within your own team or within palliative care)?

*Do you have any additions to this interview / are there things you would like to add to this conversation?*

- Thanks for the interview.
- *Inform the participant about the dissemination of results.*
- *Explain that transcripts will be returned for them to check the authenticity of the interview.*
