# Supplementary material for: Communication and shared decision-making with patients with limited health literacy; helpful strategies, barriers and suggestions for improvement reported by hospital-based palliative care providers
Source: PLoS One. 2020 Jun 19;15(6):e0234926. doi: 10.1371/journal.pone.0234926 (PMC7304585; doi:10.1371/journal.pone.0234926)
Supplement: S1 Text — (DOCX) [file pone.0234926.s003.docx]

Steps of analysis

# 1^st^ step

Two coders coded the data and, to identify initial and preliminarily themes in the material, read the first 10 transcripts, generating, discussing and reviewing initial and preliminarily codes (RR & AV).

## 1^st^ initial research question/concept used for coding: What needs do palliative care providers have to provide good care to patients with LHL?

- Healthcare providers need time.
- Healthcare providers need in-depth conversations with their patients.
- Healthcare providers need initiative from the patient (e.g., preparing for consultations, think about the future).
- Healthcare providers need the involvement of significant others during the conversations.
- Healthcare providers need training / support / skills for communicating with LHL patients (e.g., recognizing LHL).
- Healthcare providers need changes in planning and organization (e.g., more attention for palliative care and LHL).

## 2^nd^ initial research question/concept used for coding: What strategies do caregivers use to explain information understandable to patients with LHL?

- Making communication more accessible (e.g., by talking slowly, providing space for patients, avoid jargon).
- Perform a check by asking patient for a recall of information (checking comprehensibility of information).
- Using (visual) aids.
- Creating and sustaining a suitable conversation atmosphere (e.g., no follow-up consultations on the same day that the initial diagnosis is communicated, avoiding words like ‘palliative’, and ‘morphine’).
- Involving significant others in the conversation.
- Finding an entrance / boding with the patient (so that the information actually reaches the patient and the right subjects are discussed).
- Involving other healthcare providers for advice on communicating with patients.
- Planning and organization (e.g., selecting the information patients need to hear, scheduling more time).

# 1^st^ proposition of (more general) themes regarding barriers of HCPs communicating (and conducting SDM) with LHL patients

# BARRIERS

*Lack of time*

1. During the consultation
2. For a patient to make decisions

*Lack of communication skills and used strategies of healthcare providers*

- 1. No agreement between fellow HCPs on how to communicate with patients
  2. Assessment of the skills of the patient? (overestimating the knowledge/skills of the patient)
  3. Disease/treatment is to technical to explain understandably
  4. Language and cultural barriers

*Lack of preparation and skills patient*

- 1. Lack of knowledge
  2. Not seeing the consequences of choices
  3. Not able to express complaints properly
  4. No preparation before the consultation
  5. Patient wants the physician to choose

*Emotional distress patient*

- 1. Fear
  2. Anger
  3. Problems accepting the disease

*Unilateral communication*

- 1. Focussing on the physical aspect of healthcare
  2. Focussing on curing patients

*Lack of relationship building with patient?*

- 1. No ‘bond’ with the patient
  2. No ‘connection’ with the patient

## OTHERS (ORGANIZATIONAL BARRIERS)

- 1. Hassles of day-to-day healthcare in the hospital
  2. Unclear who to approach when having problems
  3. Unclear who to call during crisis
  4. Lack of budget

# 2^nd^ proposition of (more general) themes regarding suggestions to improve communication (and SDM) with LHL patients?

*More time*

1. During a consultation
2. For a patient to make a decision

*Collaboration with other healthcare providers*

- 1. With the general practitioner
  2. Aligning communication between fellow HCPs
  3. Delegating more tasks to nurses
  4. Have one point of contact for patients

*Communication strategies healthcare providers*

- 1. Reflect on own communication
  2. Communication training
  3. Visual information material
  4. Assessing / recognizing LHL
  5. Checking patient understanding

*Sensitive communication and awareness*

- 1. Not mentioning the word ‘palliative’ during conversation
  2. Acknowledge ‘not treating’ as a serious option
  3. Awareness LHL

*Deep conversations*

- 1. Advanced care planning
  2. Talk about the end of life
  3. Philosophy of life
  4. Existential topics

*Improved reporting*

- 1. Reading ‘advanced care planning reports’ by other HCPs

*Commitment patient*

- 1. Ask the patient to bring their supervisor to the consultation
  2. Initiative patient
  3. Patient asks questions
  4. Patient records the conversation

# 2^nd^ step


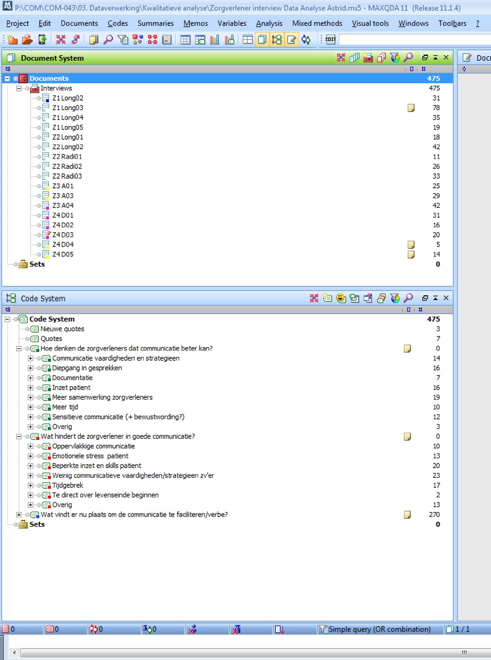
Transcripts were imported in MAXQDA11 and coded by one researcher (AV). To increase reliability, investigator triangulation was applied: ten of the interviews were additionally coded by another researcher (RR).

The ‘document system’ window shows the imported transcripts. The ‘code system’ window shows themes in MaxQDA.

Translation of themes (from top to bottom, starting with the research question: ‘hoe denken de zorgverleners dat communicatie beter kan’, ‘how do HCPs think communication can be improved’):

1. Communication, skills and strategies
2. ‘Depth’ in conversations
3. Documentation
4. Effort by patients
5. Increased collaboration HCPs
6. More time
7. Sensitive communication (and awareness)
8. Miscellaneous

Translation of themes (from top to bottom: ‘wat hindert de zorgverlener in goede communicatie’, ‘what hinders or obstructs HCPs in their communication’):

1. Superficial communication
2. Emotional distress patients
3. Limited effort and skills by patients
4. Limited communication-skills by HCPs
5. Lack of time
6. Bringing up the end of life too immediate or abrupt in conversation
7. Effort by patients
8. Increased collaboration HCPs
9. Miscellaneous

Translation of themes (from top to bottom: ‘wat vindt er nu plaats om de communicatie te faciliteren/verbeteren’, ‘what is currently happeling to facilitate/improve communication’):

- Indirect (‘verbetering van zorg maar niet van communicatie’; ‘improvement of healthcare, not of communication’, we will skip this, since there is no focus on communication)
- (top panel on next page) ‘Wat helpt de zorgverleners om goed te kunnen communiceren’; ‘What helps HCPs to communicate well’ (only translating themes with multiple codes)?

1. Communication skills of the HCP
2. Involvement of significant others
3. Being clear and honest
4. Good communication skills of the HCP
5. Character traits of HCPs
6. Having a bond / connection with the patient
7. Talking about the meaning of life with the patient
8. SDM (shared decision-making)

- (bottom panel on next page) ‘Wat doet de zorgverlener zelf om de communicatie te kunnen verbeteren’; ‘What do HCPs currently do to improve communication’ (only translating themes with multiple codes)?

1. Address more informally
2. Asking closed questions (that need a yes/no answer)
3. Use an interpreter
4. Use other language (not using medical language, or ‘physician-talk’)
5. Forming or building a bond with the patient
6. Checking patient understanding
7. Involving significant others
8. Repeating information
9. Recognizing LHL
10. Preparing consultations by reading notes of other physicians
11. Limiting information
12. Writing down information
13. Freeing up more time for the patient
14. Trying different methods or styles of communication
15. Let the patients have their say
16. Calling patients
17. SDM
18. Use a standardized format for the conversation
19. Ask patients about their feelings
20. Use visual information

#
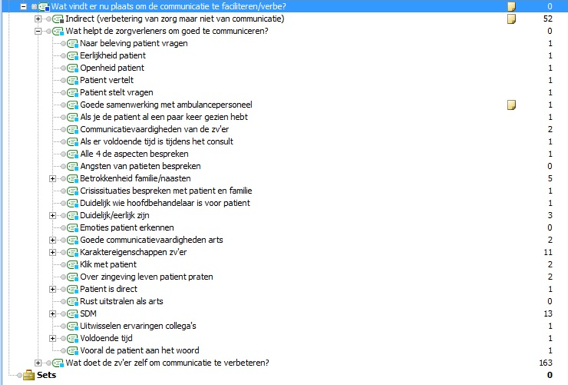

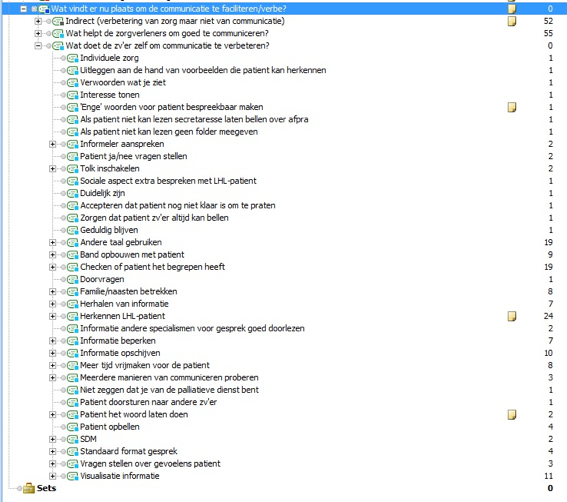


# 3^rd^ step

The themes and sub-themes that emerged during the analysis were discussed among three researchers (SvD, RR & AV), who then came to an agreement on themes.

##
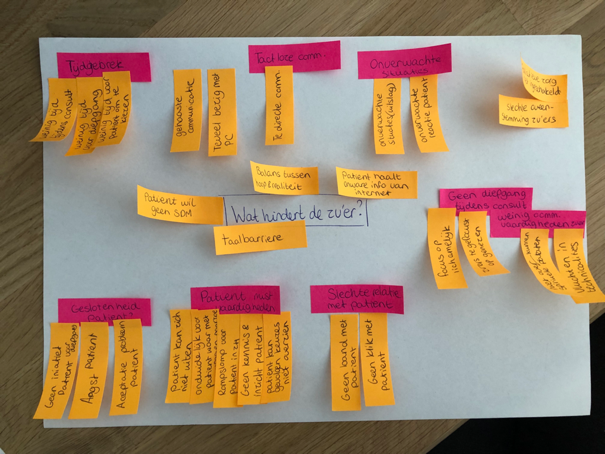
BARRIERS (prior to discussing themes and sub-themes with SvD, RR and AV)

Translation of themes (starting in the upper left corner):

- Tijdgebrek 🡪 Lack of time
- Tactloze communicatie 🡪 Tactless communication
- Onverwachte situaties 🡪 Unexpected situations
- Geslotenheid patiënt 🡪 Closeness patient
- Patiënt mist vaardigheden 🡪 Patient lacking skills
- Slechte relatie met patiënt 🡪 Bad relationship with the patient
- Geen diepgang tijdens consult 🡪 No ‘conversation-depth’ during consultations
- Weinig communicatievaardigheden zorgverlener 🡪 Limited communication skills HCP

##
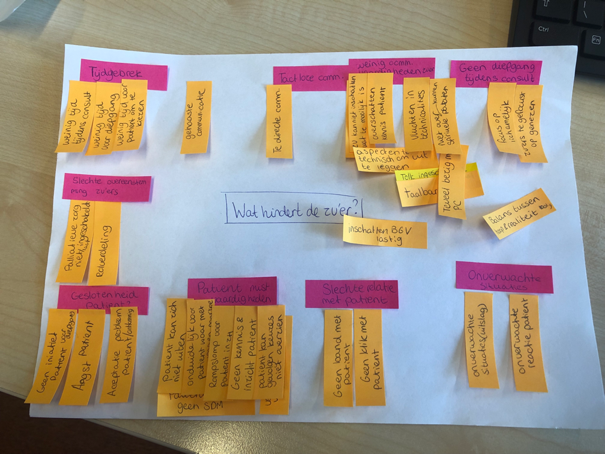
BARRIERS (after discussing themes and sub-themes with SvD, RR and AV)

Translation of themes (starting in the upper left corner):

- Tijdgebrek 🡪 Lack of time
- Tactloze communicatie 🡪 Tactless communication
- Weinig communicatievaardigheden zorgverlener 🡪 Limited communication skills HCP
- Geen diepgang tijdens consult 🡪 No ‘conversation-depth’ during consultations
- (new theme) Slechte overeenstemming zorgverleners 🡪 Limited agreement between HCPs
- Geslotenheid patiënt 🡪 Closeness patient
- Patiënt mist vaardigheden 🡪 Patient lacking skills
- Slechte relatie met patiënt 🡪 Bad relationship with the patient
- Onverwachte situaties 🡪 Unexpected situations

##
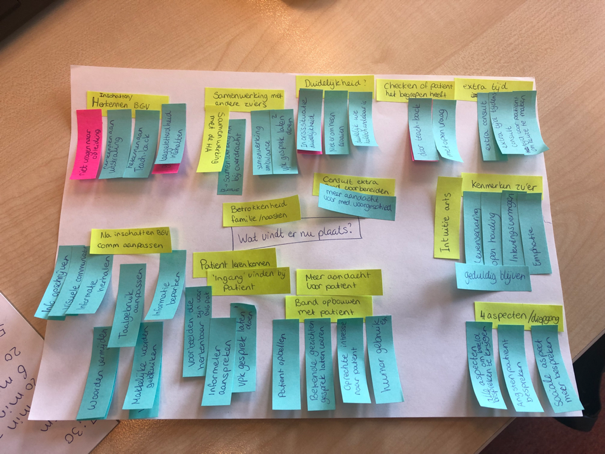
CURRENT PRACTICES (prior to discussing themes and sub-themes with SvD, RR and AV)

Translation of themes (starting in the upper left corner):

- Inschatting / herkennen BGV 🡪 Estimating / recognizing LHL
- Samenwerking met andere zorgverleners 🡪 Tactless communication
- Duidelijkheid 🡪 Clarity
- Checken of de patient het begrepen heeft 🡪 Checking patient understanding
- Extra tijd 🡪 Additional time
- Na inschatting BGV communicatie aanpassen 🡪 After estimating/recognizing LHL tailor communication
- Patiënt leren kennen / ‘ingang’ vinden bij patiënt 🡪 Getting to know the patient / connecting/bonding
- Betrokkenheid familie/naasten 🡪 Involvement by significant others
- Meer aandacht voor patiënt / band opbouwen 🡪 More attention for the patient / building a bond
- Consult extra goed voorbereiden 🡪 Preparing for the consultation
- Kenmerken zorgverlener / intuïtie arts 🡪 Characteristics HCP / intuition HCP
- 4 aspecten / diepgang 🡪 4 aspects / ‘Conversation-depth’

##
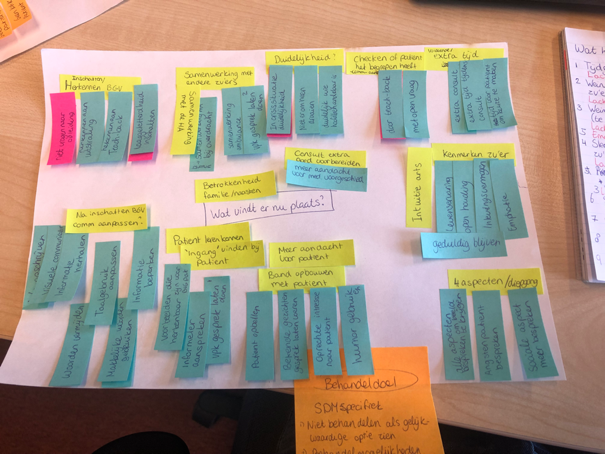
CURRENT PRACTICES (after discussing themes and sub-themes with SvD, RR and AV)

Translation of themes (starting in the upper left corner):

- Inschatting / herkennen BGV 🡪 Estimating / recognizing LHL
- Samenwerking met andere zorgverleners 🡪 Tactless communication
- Duidelijkheid 🡪 Clarity
- Checken of de patient het begrepen heeft 🡪 Checking patient understanding
- Extra tijd 🡪 Additional time
- Na inschatting BGV communicatie aanpassen 🡪 After estimating/recognizing LHL tailor communication
- Patiënt leren kennen / ‘ingang’ vinden bij patiënt 🡪 Getting to know the patient / connecting/bonding
- Betrokkenheid familie/naasten 🡪 Involvement by significant others
- Meer aandacht voor patiënt / Band opbouwen 🡪 More attention for the patient / building a bond
- Consult extra goed voorbereiden 🡪 Preparing for the consultation
- Kenmerken zorgverlener / intuïtie arts 🡪 Characteristics HCP / intuition HCP
- 4 aspecten / diepgang 🡪 4 aspects / ‘Conversation-depth’

##
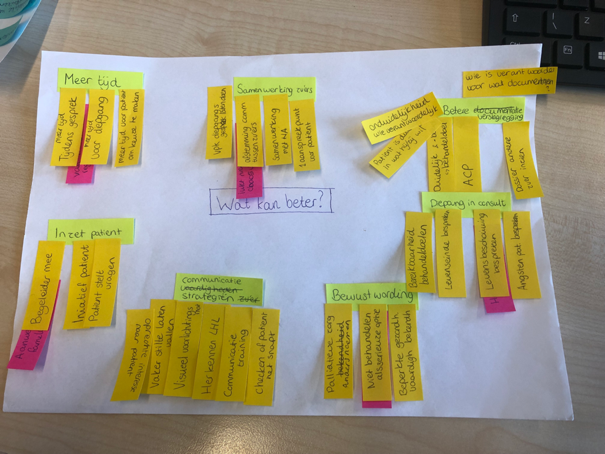
SUGGESTIONS FOR IMPROVEMENT (prior to discussing themes and sub-themes with SvD, RR and AV)

Translation of themes (starting in the upper left corner):

- Meer tijd 🡪 More time
- Samenwerking zorgverleners 🡪 Collaboration HCPs
- Betere verslaglegging 🡪 Improved reporting
- Inzet patiënt 🡪 Effort by patients
- Communicatiestrategieën 🡪 Communication strategies
- Bewustwording 🡪 Awareness
- Diepgang in consult 🡪 ‘Conversation-depth’ during consultations

##
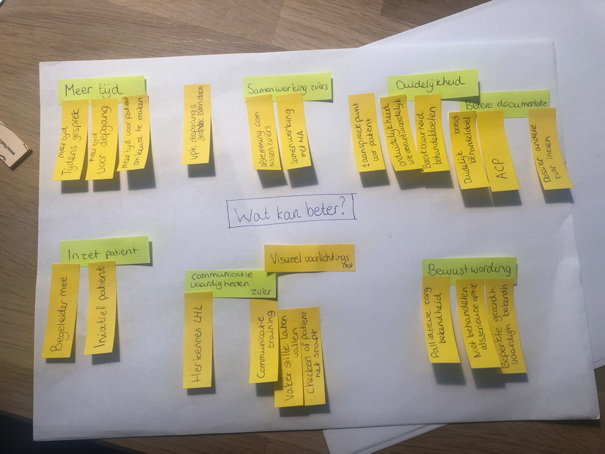
SUGGESTIONS FOR IMPPROVEMENT (after discussing themes and sub-themes with SvD, RR and AV)

Translation of themes (starting in the upper left corner):

- Meer tijd 🡪 More time
- Samenwerking zorgverleners 🡪 Collaboration HCPs
- (new theme) Duidelijkheid 🡪 Clarity
- Betere verslaglegging 🡪 Improved reporting
- Inzet patiënt 🡪 Effort by patients
- Communicatiestrategieën 🡪 Communication strategies
- (new theme) Visueel voorlichtingsmateriaal 🡪 Visual information material
- Bewustwording 🡪 Awareness
- ~~Diepgang in consult 🡪 ‘Conversation-depth’ during consultations~~

# 4^th^ step

By analyzing segments and codes within themes, one researcher (RR) finalized the naming, positioning and describing of (sub)themes and completed the analyses. A coding scheme was created (Table 2, from the manuscript), in which themes, sub-themes and elements within sub-themes were presented.

**Table 2** (Sub)themes emerging from the analyses regarding HCPs’ strategies, barriers and suggestions for improvements in communication and SDM with patient with LHL.

| **Themes** | **Sub-themes** | **Elements** |
| --- | --- | --- |
| **1. Time management** | Limited time | - practical or organizational barriers  - substantive barriers  - executing SDM |
|  | Additional time | - provide information |
|  |  | - forming a relationship - bonding |
|  |  | - lengthening the SDM process |
| **2. HCPs’ communication skills** | Observing and assessing LHL  Tools & aides |  |
|  | Limited skills | - adjusting to LHL  - unilateral outlook on treatment/care by HCPs |
|  | More collaboration | - between HCPs and disciplines  - between colleagues in the hospital |
|  | Education | - communication skills |
|  |  | - the sharing of experiences |
|  |  | - additional tools supporting HCPs during and patients prior to the consultation |
| **3. Tailoring** | Simplifying |  |
|  | Sensitizing | - comprehensive and supportive interaction |
|  | Repeating |  |
| **4. Characteristics of patients and significant others** | Knowledge |  |
|  | Attitude, mood or condition |  |
|  | Language and culture |  |
| **5. Content of medical information** |  |  |
