# Supplementary material for: Communication and shared decision-making with patients with limited health literacy; helpful strategies, barriers and suggestions for improvement reported by hospital-based palliative care providers
Source: PLoS One. 2020 Jun 19;15(6):e0234926. doi: 10.1371/journal.pone.0234926 (PMC7304585; doi:10.1371/journal.pone.0234926)
Supplement: S2 Text — (DOCX) [file pone.0234926.s004.docx]

Interview schedules

# INITIAL VERSION (5 oktober 2017)

**Interview topic list zorgverleners/organisatie medewerkers binnen palliatieve team**

**Onderwerpen 1,2 en 3 voor zorgverleners**

**Onderwerp 4 voor organisatie medewerkers binnen palliatieve team**

**Introductie**

- Welkom
- Introductie project
- Opname op audio
- Zijn er vragen?
- Tekenen toestemmingsformulier

**Topic list:**

- Uw rol als zorgverlener in de palliatieve zorg aan patiënten
- Communiceren, informeren en gezamenlijke besluitvorming
- Lage gezondheidsvaardigheden
- Uw ziekenhuis/organisatie

**Start opnamen na introductie!**

TRANSLATION

Interview topic list care providers / organization employees within palliative team

*Subjects 1,2,3 and 4 for caregivers*

*Subject 4 for administrative employees within the hospital or palliative team*

- - Welcome
  - Introduction project
  - Recording on audio
  - Questions?
  - Signing consent form

Topic list:

- - Your role as a care provider in palliative care for patients
  - Communicating, informing and shared decision making
  - Low / limited health literacy (LHL)
  - Your hospital / organization

1. **Uw rol als zorgverlener in de palliatieve zorg aan patiënten**

- Wat is uw rol in de zorg voor patiënten (en hun naasten) in de palliatieve fase?
- Wordt er altijd één centrale zorgverlener aangewezen? Is het duidelijk voor de patiënt wie dat is?
- Hoe werkt u hierin samen met andere zorgverleners binnen uw team/ziekenhuis?
- Hoe werkt u samen met de huisarts? Wat is de rol van de huisarts in deze zorg aan patiënten?
- Wat is de rol van eventuele andere zorgverleners? En van sociale en spirituele ondersteuners (pastoraal werker, imam, mantelzorgers, vrijwilligers)?

TRANSLATION

1. Your role as a healthcare provider for patients in palliative care
   - What is your role in the care of patients (and their loved ones) in the palliative phase?
   - Is there always one designated central healthcare provider for the patient?
     - Is it clear to the patient who that is?
   - How do you collaborate with other healthcare providers within your team / hospital?
   - How do you collaborate with the general practitioner?
     - What is the role of the general practitioner in the care for patients in palliative care?
   - What is the role of other care providers? And of social or spiritual care assistants (a pastoral worker, imam, informal caregivers or volunteers)?
2. **Communiceren, informeren en gezamenlijke besluitvorming**

- Bent u bekend met het kwaliteitskader palliatieve zorg Nederland (van het IKNL en palliatief, uitgekomen in september 2017)? Hierin worden vier dimensies van beoordeling en behandeling van problemen binnen de palliatieve zorg aan patiënten gegeven: fysiek, psychisch, sociaal en spiritueel welzijn van de patiënt. In hoeverre komen deze dimensies terug tijdens het communiceren en informeren van de patiënt?
- (Indien van toepassing) worden afspraken met aandacht voor het fysiek, psychisch, sociaal en spiritueel welzijn vastgelegd in een individueel zorgplan?
- De basis voor goede zorg is effectieve communicatie, een goed geïnformeerde patiënt en gezamenlijke besluitvorming. In hoeverre is er volgens u sprake van gezamenlijke besluitvorming met patiënten in de palliatieve fase? Ziet u verbeterpunten op dit gebied?
- Belangrijke thema’s in de zorg voor patiënten in deze fase zijn onder meer: levensbeschouwing en culturele achtergrond, (niet)-behandelafspraken, ziekenhuisopnames, plaats van zorg en sterven, crisissituaties, wettelijke vertegenwoordiging in de situatie van (acute) verslechtering en wilsonbekwaamheid en levenseinde-beslissingen. In hoeverre komen deze onderwerpen aanbod tijdens uw consulten? Zo niet, wordt dit volgens u besproken met een andere zorgverlener (binnen uw team of bijvoorbeeld de huisarts)? In hoeverre is er sprake van gezamenlijke besluitvorming?

TRANSLATION

1. Communicating, informing and shared decision-making

*Are you familiar with the quality framework for palliative care in the Netherlands (published by the IKNL, released in September 2017)? It provides patients with four dimensions of assessing problems within palliative care: the physical, psychological, social and spiritual well-being of the patient.*

- - To what extent do these dimensions recur during communicating and informing the patient?
  - (If applicable) are agreements with patients on the physical, psychological, social and spiritual well-being recorded in an individual care plan?

*The foundation for delivering good healthcare is effective communication, a well-informed patient and shared decision-making.*

- To what extent do you think there is shared decision-making with patients in the palliative phase?
  - What are areas for improvement?

*Important themes in the care of patients in this phase include: philosophy of life and cultural background, treatment and non-treatment agreements, hospital admissions, place of care and death, crisis situations, legal representation, and end-of-life decisions.*

- - To what extent do these topics come up during your consultations with patients?
  - If not, do you think this will be discussed with another healthcare provider (within your team or with the doctor, for example)?
  - To what extent does shared decision-making take place?

1. **Lage gezondheidsvaardigheden**

- Wat verstaat u onder het begrip lage gezondheidsvaardigheden of low health literacy?
- Heeft u weleens te maken met patiënten met lage gezondheidsvaardigheden?
- Hoe herkent u patiënten met lage gezondheidsvaardigheden?
- Op welke manier probeert u rekening met hen te houden in uw mondelinge en schriftelijke communicatie?/ (Hoe gaat u daar mee om?)
- Ervaart u barrières in de zorg aan patiënten met lage gezondheidsvaardigheden? Zo ja, welke barrières?
- Ervaart u deze barrières ook met andere patiënten of alleen met deze groep?
- Wat heeft u nodig om zorg beter aan te laten sluiten op patiënten met lage gezondheidsvaardigheden?

TRANSLATION

1. Limited health literacy (LHL) (in Dutch ‘beperkte gezondheidsvaardigheden’).
   - What do you think limited or low health literacy means?
   - Do you ever deal with patients with LHL?
   - How do you recognize patients with LHL?
   - In what way do you try to take them into account in your oral and/or written communication?
     - How do you deal with that?
   - Do you experience barriers in the care of patients with LHL?
     - If so, which barriers?
     - Do you also experience these barriers with other patients?
   - What do you need to ensure that care is better aligned for patients with LHL?
2. **Uw ziekenhuis/organisatie**

- Is er beleid binnen uw ziekenhuis om de zorg toegankelijk(er) te maken voor patiënten met lage gezondheidsvaardigheden?
- Worden binnen uw organisatie bepaalde methodieken/instrumenten gebruikt om de zorg toegankelijk(er) te maken voor mensen met lage gezondheidsvaardigheden?
- Wat zou volgens u belangrijk zijn voor uw ziekenhuis om de zorg voor mensen met lage gezondheidsvaardigheden toegankelijk(er) te maken?
- Welke mogelijkheden ziet u? (bv. Aanpassen patiënten records, informatie, extra consult inplannen)
- Welke barrières ziet u op organisatieniveau?
- Zou u hier specifiek beleid op willen? Waarom niet/wel?
- Wie (zorgverleners, organisatie binnen uw ziekenhuis of daarbuiten) zou volgens u hierin een rol moeten/kunnen spelen?
- En op welk niveau binnen het ziekenhuis zou dit volgens u zichtbaar moeten zijn? (algeheel beleid ziekenhuis, binnen mijn team, binnen de palliatieve zorg…)

TRANSLATION

1. (if applicable) Your hospital/organization
   - Is there policy within your hospital to make healthcare more accessible for patients with LHL?
   - Are certain methods or instruments used within your organization to make healthcare more accessible for people with LHL?
   - What do you think is important for your hospital to make healthcare for people with LHL more accessible?
   - What are possibilities for this (for example, adjusting patient records or schedule additional consultations)?
   - What barriers do you perceive at the organizational level?
   - Would you want specific policy on this?
     - Why or why not?
   - Who (care providers, the organization within your own hospital or outside your own hospital) should play a role in this?
   - And at what level within the hospital do you think this should be visible (in the general hospital policy, within your own team or within palliative care)?

**Overig**

- Heeft u nog aanvullingen op dit interview/ zijn er dingen die u zou willen toevoegen aan dit gesprek die volgens u van belang zijn?

**Afsluiting**

- Bedanken
- Vertellen wat we met deze resultaten gaan doen
- Eventueel uitgetypt interview opsturen indien ze dat willen.

TRANSLATION

*Do you have any additions to this interview / are there things you would like to add to this conversation?*

- Thanks for the interview.
- Inform the participant about the dissemination of results.
- Explain that transcripts will be returned for them to check the the authenticity of the interview.

#

# DEFINITIVE VERSION (5 april 2018)

**Interview topic list zorgverleners/organisatie medewerkers binnen palliatieve team**

**Onderwerpen 1,2,3 en 4 voor zorgverleners**

**Onderwerp 4 voor organisatie medewerkers binnen palliatieve team**

**Introductie**

- Welkom

*Alvast bedankt voor uw waardevolle tijd! We hebben de inzichten van medici als u hard nodig om ons onderzoek te kunnen uitvoeren. Uiteindelijk willen we hetzelfde als u en uw collega’s; het allerbeste voor de patiënt.*

- Introductie project

*Vandaag wil ik u graag wat algemene vragen stellen over uw rol als zorgverlener in de palliatieve zorg, over communicatie, over patiënten en over de manier waarop uw ziekenhuis georganiseerd is.*

- Opname op audio

*Met uw toestemming zou ik het gesprek graag opnemen. Dit maakt het uitwerken van het interview mogelijk voor mij. U blijft natuurlijk anoniem. Vindt u dat goed?*

- Tekenen toestemmingsformulier

*Kunt u dan, voordat we beginnen, tekenen voor uw toestemming? Dan hebben we dat maar geregeld.*

- Zijn er vragen tot zover?

**Start opnamen na introductie!**

TRANSLATION

Interview topic list care providers / organization employees within palliative team

*Subjects 1,2,3 and 4 for caregivers*

*Subject 4 for administrative employees within the hospital or palliative team*

- Introduction
  - Welcome
- *Thanks in advance for your valuable time! We really need the insights of medical professionals like you to conduct our research. Ultimately, we want the same as you and your colleagues; the very best for patients.*
  - Introduction project
- *Today I would like to ask you some general questions about your role as a care provider in palliative care, about communication, about your patients and about the way your hospital is organized.*
  - Recording on audio
- *With your permission, I would like to record the conversation. This makes it possible for me to transcribe the interview. You will remain anonymous. Are you okay with that?*
  - Signing consent form
- *Before we start, could you perhaps sign for your permission?*
  - Are there any questions so far?

1. **Uw rol als zorgverlener in de palliatieve zorg aan patiënten**

- Wat is uw rol in de zorg voor patiënten (en hun naasten) in de palliatieve fase?
- Wordt er altijd één centrale zorgverlener aangewezen?
  - Is het duidelijk voor de patiënt wie dat is?
- Hoe werkt u hierin samen met andere zorgverleners binnen uw team/ziekenhuis?
- Hoe werkt u samen met de huisarts?
- Werkt u samen met maatschappelijk werk?

(of bijvoorbeeld pastoraal werker of imam of met mantelzorgers of met vrijwilligers?)

TRANSLATION

1. Your role as a healthcare provider for patients in palliative care
   - What is your role in the care of patients (and their loved ones) in the palliative phase?
   - Is there always one designated central healthcare provider for the patient?
     - Is it clear to the patient who that is?
   - How do you collaborate with other healthcare providers within your team / hospital?
   - How do you collaborate with the general practitioner?
   - Do you collaborate with social workers? (e.g. a pastoral worker, imam, with informal caregivers or with volunteers?)
2. **Communiceren, informeren en gezamenlijke besluitvorming**

*Binnen de palliatieve zorg is aandacht van belang voor meerdere aspecten, namelijk voor zowel het* ***fysieke****,* ***psychische****,* ***sociale*** *als* ***spirituele welzijn*** *van de patiënt.*

- In hoeverre komen deze verschillende aspecten aan bod in uw gesprekken met de patiënt?
- Brengt u uit u zelf deze aspecten ter sprake?
  - Bij alle patiënten?
  - Ook bij mensen die laag opgeleid zijn, of niet veel lijken te snappen?
    - Zo ja, welke aspecten?
    - Hoe vraagt u daarnaar?
    - In welke fase van het consult of van de begeleiding?
    - Vraagt u er elke keer naar?
- Werkt u met een individueel zorgplan?
  - Bij alle patiënten?
- Worden afspraken rond al deze aspecten (fysiek, psychisch, sociaal en spiritueel welzijn) vastgelegd in een individueel zorgplan?
  - Welke wel en welke niet?

TRANSLATION

1. Communicating, informing and shared decision-making

*Within palliative care, attention for the physical, psychological, social and spiritual well-being of the patient is important.*

- - To what extent are these different aspects addressed in your conversations with the patient?
  - Do you bring up these aspects yourself?
    - With all patients?
    - Also, for people with a low level of education, or who don't seem to understand much? If yes, which aspects?
  - How do you ask about these aspects?
    - In which phase of the consultation or supervision?
    - Do you ask for it every time?
  - Do you work with an individual care plan?
  - Are agreements on all these aspects (physical, psychological, social and spiritual well-being) recorded in an individual care plan?
    - Which is and which is not?

*Goede communicatie is de basis voor goede zorg, zodat de patiënt goed geïnformeerd is en er sprake kan zijn van gezamenlijke besluitvorming.*

- Zijn er patiënten bij wie u de communicatie moeilijker vindt?

(bijvoorbeeld als ze laag opgeleid zijn, of uit een lage sociaal klasse lijken te komen, of een migratie achtergrond hebben?)

- In welk opzicht is de communicatie moeilijker bij deze mensen?
- Wat doet u om de communicatie te verbeteren?
- Zijn er momenten dat u bewust uw communicatie aanpast en hoe dan?
  - Kunt u een voorbeeld geven?
- Checkt u wel eens wat uw patiënten hebben begrepen?
  - Bespreekt u met deze mensen minder zaken dan met anderen?
  - Gebruikt u filmpjes of andere visueel voorlichtingsmateriaal?
- In hoeverre is er volgens u sprake van gezamenlijke besluitvorming met patiënten in de palliatieve fase?
  - Is dit voor alle patiënten hetzelfde?
  - Denkt u dat het beter zou zijn als er meer gezamenlijke besluitvorming plaats vindt?
  - Kunt u een voorbeeld geven van een situatie waarin er duidelijk sprake was van gezamenlijke besluitvorming,
  - Kunt u een voorbeeld geven van een situatie waarin dat juist niet gebeurde?
    - Wat betekent dat voor de zorg, voor de patiënt en voor u?
    - Als u vindt dat het beter zou kunnen, wat zou daarvoor nodig zijn?

*(leg de kaartjes op tafel)*

*Belangrijke thema’s in de zorg voor patiënten in deze fase zijn onder meer: levensbeschouwing en culturele achtergrond, (niet)-behandelafspraken, ziekenhuisopnames, plaats van zorg en sterven, crisissituaties, wettelijke vertegenwoordiging in de situatie van (acute) verslechtering en wilsonbekwaamheid en levenseinde-beslissingen.*

- Kunt u met behulp van een rangordening in de kaartjes aangeven in hoeverre deze onderwerpen aan bod komen tijdens uw consulten? Het meest links mag u de kaartjes leggen die het meest aan bod komen in uw consulten, het meest rechts de kaartjes die het minst aan bod komen.
- Zo niet (de kaartjes die aan de rechterkant liggen), wordt dit volgens u besproken met een andere zorgverlener (binnen uw team of met bijvoorbeeld de huisarts)?
  - In hoeverre is er sprake van gezamenlijke besluitvorming?

TRANSLATION

*Good communication is the basis for good care, so that the patient is well informed and there can be shared decision-making.*

- Are there patients for whom you find communication more difficult? (e.g. if they have a low level of education, or appear to come from a low social class, or have a migration background?)
- In what way is communication more difficult for these people?
- What do you do to improve communication?
- Are there moments when you consciously adjust your communication and how?
  - - Can you give an example?
- Do you ever check what your patients have understood everything?
- Do you discuss fewer things with these people compared with others?
- Do you use videos or visual information materials?
- To what extent do you think there is shared decision-making with patients in the palliative phase?
  - - Is this the same for all patients?
- Do you think it would be better if more shared decision-making took place?
- Can you give an example of a situation where there a clear case of shared decision-making?
- Can you give an example of a situation in which shared decision-making did not happen?
  - - What does that mean for healthcare, for the patient and for you?
- If you think it shared decision-making could be better practiced, what would it take to improve this?

*The interviewer puts paper cards on the table, with important themes in patient care in the palliative phase written on them; philosophy of life and cultural background, treatment and non-treatment agreements, hospital admissions, place of care and death, crisis situations, legal representation, and end-of-life decisions. After putting the cards on the table, the interviewer continues the interview.*

- Can you indicate to what extent these topics are covered during your consultations with the help of a ranking in the cards? On the left you can lay the cards that are the most dealt with in your consultations, the right on the cards that are the least dealt with.
- If not (the cards on the right-hand side), do you think this will be discussed with another healthcare provider (within your team or with the doctor, for example)?
  - - To what extent does shared decision-making take place?

1. **Beperkte gezondheidsvaardigheden**

- Hebt u wel eens gehoord van het begrip beperkte gezondheidsvaardigheden?
  - Wat betekent dat denkt u?

*Introductie van definitie LHL:*

*De vaardigheden van mensen om informatie over gezondheid te krijgen, te begrijpen en toe te passen bij het nemen van beslissingen over gezondheid.* ***Functioneel*** *(lezen en schrijven, rekenen etc.),* ***interactief/communicatief*** *(begrijpend lezen, hoofd- van bijzaken scheiden, vragen stellen etc.) en* ***kritisch*** *(toepassen informatie, ordenen, vooruitdenken, prioriteiten stellen etc.).*

- Hoe vaak hebt u te maken met patiënten met beperkte gezondheidsvaardigheden?
- Hoe herkent u patiënten met beperkte gezondheidsvaardigheden?
- Op welke manier probeert u rekening met hen te houden in uw mondelinge en schriftelijke communicatie?
- Ervaart u barrières in de zorg aan patiënten met beperkte gezondheidsvaardigheden?
  - Zo ja, welke barrières?
  - Ervaart u deze barrières ook met andere patiënten?
- Wat heeft u nodig om zorg beter aan te laten sluiten op patiënten met beperkte gezondheidsvaardigheden?

TRANSLATION

1. Limited health literacy (LHL) (in Dutch ‘beperkte gezondheidsvaardigheden’).
   - Have you ever heard of the concept of LHL?
     - What do you think that means?

*The interviewer introduces the HCP with a definition/meaning of LHL:*

*People's skills to obtain, understand and apply information on health and healthcare when making health decisions.* ***Functional*** *(reading and writing, math, etc.),* ***interactive / communicative*** *(reading comprehension, separating main from side issues, asking questions, etc.) and* ***critical assessment*** *(applying information, ordering/organizing information, thinking ahead, setting priorities, etc.).*

- - How often do you deal with patients with LHL?
  - How do you recognize patients with LHL?
  - In what way do you try to take them into account in your oral and/or written communication?
  - Do you experience barriers in the care of patients with LHL?
    - If so, which barriers?
    - Do you also experience these barriers with other patients?
  - What do you need to ensure that care is better aligned for patients with LHL?

1. **Uw ziekenhuis/organisatie**

- Is er beleid binnen uw ziekenhuis om de zorg toegankelijk(er) te maken voor patiënten met beperkte gezondheidsvaardigheden?
- Worden binnen uw organisatie bepaalde methodieken/instrumenten gebruikt om de zorg toegankelijk(er) te maken voor mensen met beperkte gezondheidsvaardigheden?
- Wat zou volgens u belangrijk zijn voor uw ziekenhuis om de zorg voor mensen met beperkte gezondheidsvaardigheden toegankelijk(er) te maken?
- Welke mogelijkheden ziet u (bijvoorbeeld, aanpassen patiënten records, informatie, extra consult inplannen)?
- Welke barrières ziet u op organisatieniveau?
- Zou u hier specifiek beleid op willen?
  - Waarom niet/wel?
- Wie (zorgverleners, organisatie binnen uw ziekenhuis of daarbuiten) zou volgens u hierin een rol moeten/kunnen spelen?
- En op welk niveau binnen het ziekenhuis zou dit volgens u zichtbaar moeten zijn (algeheel beleid ziekenhuis, binnen mijn team, binnen de palliatieve zorg)?

TRANSLATION

1. (if applicable) Your hospital/organization
   - Is there policy within your hospital to make healthcare more accessible for patients with LHL?
   - Are certain methods or instruments used within your organization to make healthcare more accessible for people with LHL?
   - What do you think is important for your hospital to make healthcare for people with LHL more accessible?
   - What are possibilities for this (for example, adjusting patient records or schedule additional consultations)?
   - What barriers do you perceive at the organizational level?
   - Would you want specific policy on this?
     - Why or why not?
   - Who (care providers, the organization within your own hospital or outside your own hospital) should play a role in this?
   - And at what level within the hospital do you think this should be visible (in the general hospital policy, within your own team or within palliative care)?

**Overig**

- Heeft u nog aanvullingen op dit interview/ zijn er dingen die u zou willen toevoegen aan dit gesprek die volgens u van belang zijn?

**Afsluiting**

- Bedanken
- Vertellen wat we met deze resultaten gaan doen
- Eventueel uitgetypt interview opsturen indien ze dat willen.

TRANSLATION

*Do you have any additions to this interview / are there things you would like to add to this conversation?*

- Thanks for the interview.
- Inform the participant about the dissemination of results.
- Explain that transcripts will be returned for them to check the authenticity of the interview.
