# Supplementary material for: Communication and shared decision-making with patients with limited health literacy; helpful strategies, barriers and suggestions for improvement reported by hospital-based palliative care providers
Source: PLoS One. 2020 Jun 19;15(6):e0234926. doi: 10.1371/journal.pone.0234926 (PMC7304585; doi:10.1371/journal.pone.0234926)
Supplement: S3 Text — (DOCX) [file pone.0234926.s005.docx]

Z1long02

I: interviewer

R: respondent

**I: Ja, nou, dank je wel. Verpleegkundig specialist. Normaal, als ik interviews heb… Ik had die met jou geloof ik in augustus of zo gepland hè, dus die haal ik nou uit mijn agenda als je het goed vindt.**

R: Ja, dat is goed.

**I: Normaal zoek ik even zelf op van tevoren wat iemand precies doet en zo, maar kun jij misschien kort iets vertellen over jouw rol in het ziekenhuis?**

R: Ja, ik werk dus nu sinds een jaar, dik een jaar, als verpleegkundig specialist hier bij het palliatieve team en voorheen heb ik altijd als verpleegkundige gewerkt, gewoon op de verpleegafdeling van de Longziekten. Ik geloof dat daar ook heel veel interviews van dit onderzoek…

**I: Inderdaad. We hebben gefilmd bij G., bij B., bij P., bij S. Zeggen die namen je wat?**

R: Ja, zeker, zeker, zeker. Daar heb ik vaker mee samengewerkt. Daar als verpleegkundige en toen de opleiding tot verpleegkundig specialist, dus dan ben je gespecialiseerd in palliatieve zorg, heb je dus artsenbevoegdheden binnen een klein vakgebied, dus voorschrijfbevoegdheid en beleidsbevoegdheid. Ja. Dus dat is wel heel leuk. Ja.

**01.46**

**I: Dat is goed om te weten. Ik heb een aantal vragen over jouw rol als zorgverlener in de palliatieve zorg. Wat is jouw rol in de zorg voor patiënten in de palliatieve fase?**

R: Ik denk dat het met name mijn rol is om de patiënt centraal te stellen, dus om alle facetten van de zorg te belichten. Dan heb ik het over het lichamelijke aspect… Dan heb ik het over de palliatieve zorg. Het lichamelijke aspect, het psychische aspect, het sociale aspect, maar ook het zingevingsaspect, om die facetten allemaal te belichten en te kijken, wat wil de patiënt, waar hoopt hij op, wat zijn zijn wensen, wat wij hij juist niet? Ik denk dat mijn rol daarin is om samen met de patiënt duidelijk te krijgen wat hij wel en niet wil. Ja.

**I: Als ik mag inhaken op die aspecten; ik heb ze hier ook opgeschreven, maar een beetje anders. Het fysieke, het psychische, het sociale en het spirituele. Dat zijn natuurlijk belangrijke aspecten binnen de palliatieve zorg, zoals je ook aanhaalt. In hoeverre komen die verschillende aspecten aan bod in gesprekken met patiënten?**

R: Daar zit inderdaad wat verschil in. We worden geconsulteerd door de artsen hier in huis, dus we komen niet uit…

**I: Dus jullie worden erbij geroepen.**

R: Ja, wij komen niet uit onszelf. Wij komen op basis van consultatie bij een patiënt. Daarmee is er meestal ook een ingangsvraag en dat is afhankelijk van de arts. In de meeste gevallen heeft de ingangsvraag een lichamelijk aspect, dus mensen hebben klachten, pijn, kortademigheid, misselijkheid, braken, wat door de arts zelf niet onder controle gebracht wordt en ze vragen daarom advies. Dat zijn eigenlijk de meeste consultvragen, dus dat gaat met name om het lichamelijke stuk en wat wij wel proberen is om tijdens zo’n bezoek aan de patiënt ook die andere drie dimensies, die andere drie aspecten wel ter sprake te brengen. Meestal wordt daar niet over gesproken of niet naar gevraagd en dat proberen wij wel naar boven te krijgen. “Zijn er daar nog klachten of dingen die de patiënt graag wil bespreken?”

**I: Is dat bij alle patiënten hetzelfde?**

R: Nee. Nee, zeker niet. Nee. Kijk, ieder mens is verschillend en niet alle patiënten willen het bijvoorbeeld over het zingevingsstukje hebben of zijn daar nog niet klaar voor of zijn heel realistisch ingesteld en hebben het alleen over de feiten en niet over hun gevoel. Dat is heel verschillend. Ja.

**I: Dus als ik jou goed begrijp – als ik het verkeerd zeg moet je me verbeteren – is er een openingsvraag. Jij noemde het anders.**

R: Ja, een consultvraag. De arts heeft een ingangsvraag.

**I: De ingangsvraag, ja, precies. Dus je hebt die ingangsvraag en dan is het vaak, wat je aangaf, een lichamelijke klacht die eruit komt. Maar er is op een gegeven moment denk ik een moment waarop jij denkt van, hé, maar hier moet ik toch echt even gaan doorvragen over die andere drie dingen of zo.**

R: Ja. Ja.

**I: Hoe werkt dat dan? Kun je daar iets meer over vertellen?**

R: Meestal merk je gedurende het gesprek wel… Wij krijgen nogal veel tijd. Wij mogen zo lang… Nou, “zo lang”… Wij mogen vrij lang een gesprek aangaan met een patiënt en wat je dan ziet is dat de patiënt soms zelf al een paar van die aspecten en van die facetten belicht, maar soms vragen wij er ook naar, van, hoe gaat het, bent u angstig, bent u somber, wat doet de ziekte met u, hoe ziet u de toekomst? Dat zijn wel vragen die meestal de patiënt triggeren om ook over die andere facetten te spreken. Ja.

**05.51**

**I: Dus je hebt zo’n consult gehad, zo’n uitgebreid consult gehad met zo’n patiënt. Dan is het over een aantal aspecten gegaan of over allemaal. Leggen jullie die ook vast in een individueel zorgplan of zo?**

R: Wat wij doen is inderdaad… Het gaat over de documentatie, denk ik hè?

**I: Ja.**

R: De documentatie doen wij in een nationaal registratiesysteem – dat heet PRADO – maar tevens ook hier in het dossier van het ziekenhuis, waardoor dus alle medici en paramedici die ook betrokken zijn bij de patiënt kunnen lezen wat wij hebben gedaan en wat ons advies is. Wij hebben nog altijd een adviserende rol en geven het advies terug aan de behandelend arts. Zij kijken wat ze met het advies doen.

**I: Oké, dus al die aspecten worden daarin vastgelegd.**

R: Ja. Ja.

**I: Even kijken. Dan maak ik even een stapje terug, weer over die betrokkenheid. Je gaf aan dus dat jullie een adviserende rol hebben. Jullie zijn eigenlijk de spin in het web, als ik het goed begrijp, toch?**

R: Ja, wij proberen met een helikopterview te kijken van, wat kan er in de zorg op al die vier aspecten nog verbeterd worden en wat is daarin de wens van de patiënt? Dat betekent niet dat wij alles kunnen oplossen, maar misschien kan er wel een andere paramedicus betrokken worden, is een geestelijk verzorger in deze casus van belang/nodig. Dus zo proberen wij er een beetje boven te hangen en te kijken van, wat heeft de patiënt nodig en wat wil de patiënt?

**I: Ja, precies. Behalve intern in het ziekenhuis, hoe werken jullie samen met bijvoorbeeld een huisarts?**

R: Wij werken ook transmuraal, dus wij krijgen consultvragen van artsen hier in huis, maar zeker ook telefonische consulten van huisartsen. Eigenlijk bestaat het overgrote deel uit vragen van huisartsen.

**I: Ja, dat had ik gezien in… Ik had dit gekregen van A. Even kijken hoor. Maar goed, sorry, ga verder.**

R: Ja. Het overgrote deel. Aan hen geven wij telefonisch advies en wij kunnen hun ook de optie van een huisbezoek aanbieden.

**I: Oké, dat jullie even langskomen mocht het…**

R: Ja, precies. Mocht de huisarts er zelf niet uitkomen en hij zegt, goh, kun je toch eens een keer meekijken, dan kunnen wij hier in de regio een huisbezoek doen. Wat ook meestal wordt gedaan; als wij hier betrokken zijn geweest bij een patiënt, lichten wij de huisarts in over onze betrokkenheid, waarbij zij de mogelijkheid hebben om te blijven communiceren met ons en te sparren over, hoe voer ik nou het beste beleid?

**08.38**

**I: Volgens mij noemde je het net al hoor, maar werken jullie ook samen met maatschappelijk werk?**

R: Ja.

**I: Ook met de pastoraal werker of met de imam.**

R: Ja, precies. In ons team zijn een geestelijk verzorger en maatschappelijk werker betrokken. Met hen hebben we korte lijntjes en we kunnen hen ook betrekken bij de zorg van de patiënt.

**I: Ja, die zitten eigenlijk ook echt in jullie team, toch?**

R: Ja, precies. Die zitten ook wekelijks bij ons MDO, waar wij alle consulten die wij die week hebben gehad nabespreken en kijken of er andere visies zijn, misschien aanpassingen in het beleid of in het advies. Ja.

**I: Even denken hoor. Als je het goed vindt wil ik weer even terug naar het communiceren, informeren en gezamenlijke besluitvorming.**

R: Ja.

**I: Een goede communicatie is de basis voor goede zorg, zodat de patiënt goed geïnformeerd is en er sprake kan zijn van gezamenlijke besluitvorming. Zijn er patiënten bij wie jij de communicatie moeilijker vindt?**

R: Absoluut. Ja. Daar heb ik denk ik wel het meeste mee te maken, omdat ik het meeste betrokken ben bij de Longafdeling, nu nog steeds. Daar merk je gewoon dat het inderdaad door de laaggeletterdheid soms moeilijk is om uitleg te geven over al die medicatie, over al die adviezen die je hebt. Soms zien de mensen door de bomen het bos niet meer en dan is het heel moeilijk om eenvoudig uitleg te geven die blijft hangen, die ze ook daadwerkelijk kunnen toepassen. Ja. Dat vind ik wel moeilijk. Vooral bij de COPD-patiënten. Ik zeg niet bij allemaal, maar bij een groot deel daarvan wel.

**I: Bij een groot gedeelte. Mijn volgende vraag zou zijn, in welk opzicht is die communicatie dan moeilijker? Maar als ik jou goed begrijp is dat echt het begrijpelijk, eenvoudig…**

R: Ja, het eenvoudig uitleggen. Natuurlijk gebruik je geen vakjargon, maar er zijn soms woorden of gezegdes die de mensen anders interpreteren dan jij ze uitlegt, waarbij je zelf achteraf denkt van, dat was toch heel normaal uitgelegd, heel eenvoudig? Maar dan blijkt het toch niet zo eenvoudig te zijn als dat jij het uitlegt.

**I: Zoals bijvoorbeeld? Of kun je een voorbeeld daarvan geven?**

R: Poeh.

**I: Ja, dat is een lastige vraag, sorry.**

R: Daar moet ik heel goed over nadenken. Ja, je hebt dan bijvoorbeeld mensen die lang in bed liggen, zoals mensen die in de palliatieve fase vaak bedlegerig zijn. Die krijgen doorligplekken. Wij noemen dat decubitus. In de volksmond weten sommige mensen dat ook. Heel veel mensen denken, decubitus, waar heb jij het over? Je moet het dan echt over de plekken, de wonden hebben door het lange liggen. Dat is een voorbeeld. Ja. Dat is dan sneller gezegd… Dan denk ik achteraf, ja, misschien begrijpen ze helemaal niet wat ik zeg.

**I: Maar dat is dan een voorbeeld van dat je vakjargon vermijdt, toch?**

R: Ja, ik probeer vakjargon te vermijden, maar soms…

**I: Of is dit niet eens vakjargon, want ik wist het niet eerlijk gezegd. Maar dat zegt misschien meer over mij dan over…**

R: Ja, omdat het voor ons zo normaal is denken wij, nou, dat is toch helemaal niet moeilijk? Maar voor hen is dat zeker moeilijk. Dat moet je gewoon inderdaad daaruit… Ja, het is moeilijk om je daarin te verplaatsen dan.

**12.22**

**I: Dat kan ik me voorstellen. En behalve het vermijden van vakjargon, wat doe je nog meer om de communicatie te verbeteren?**

R: Om het helderder voor de patiënt te maken, bedoel je?

**I: Ja.**

R: Ik gebruik soms tekeningen of aantekeningen als ik bijvoorbeeld de werkzaamheid… Als ik het gebruik van bepaalde medicatie uitleg probeer ik dat met tekeningen te ondersteunen en ik schrijf het ook op, zodat ze wat ik heb uitgelegd nog eens kunnen nalezen.

**I: Dus voor thuis, dat ze het nog even weten.**

R: Ja. Ja. Ja. Ja.

**I: Zijn er momenten dat je bewust de communicatie aanpast? Zijn daar specifieke momenten voor?**

R: Meestal zie ik de patiënt meerdere keren, dus niet één keer. Op het moment dat ik tijdens het eerste consult merk dat er gewoon sprake is van laaggeletterdheid of moeite om dingen te begrijpen, ga ik dat wel in een volgend consult aanpassen. Ja. Dus het is niet dat ik standaard bij iedere patiënt met een tekening aan kom zetten, maar dat schat ik in aan de hand van, wat kan de patiënt begrijpen?

**I: Hoe schat je dat dan in?**

R: Meestal vraag ik aan het einde van het consult van, goh, wat heeft u begrepen? Ik ga niet vragen, heeft u het begrepen, want dan zegt meestal iedereen “ja”. Maar wel, wat heeft u ervan begrepen? Aan de hand van de uitleg die de patiënt dan geeft aan mij schat ik in in hoeverre ze het begrepen hebben en of daar aanpassingen aan moeten… Ja.

**I: Dus het eerste consult is eigenlijk heel belangrijk. Daar maak je die inschatting dan ook bij. Aan het einde check je eigenlijk of de mensen het hebben begrepen, als ik je goed begrijp.**

R: Ja. Ja. Dat. En ik vraag meestal ook bij de verpleegkundige die eigenlijk de hele dag bij die mensen betrokken is – want ik zie hen maar een half uur, een uur – wel na van, hoe zien jullie dat? Dus ik vraag wel de mening van de zorgverleners die daar de hele dag over de kamer lopen. Ja.

**I: In hoeverre is er volgens jou sprake van gezamenlijke besluitvorming met patiënten in de palliatieve fase?**

R: Bedoel je dan met mij of met de artsen? Wij hebben een adviserende rol. Kijk, bij alles wat wij adviseren vraag ik of ze dat goed vinden, wat ik adviseer. Maar omdat wij natuurlijk geen beleid voeren vind ik dat moeilijk. Ik stel altijd voor en vraag inderdaad of ze akkoord gaan, ja of nee, omdat het hun lichaam is en hun keuze. Pas als zij akkoord geven ga ik het advies ook daadwerkelijk aan de arts voorleggen.

**I: Ja, precies. Dus de besluitvorming in jouw geval, in jouw adviserende rol, is eigenlijk, vind je het goed dat ik dit en dat adviseer aan de…**

R: Ja. Ja, precies. Ja.

**15.35**

**I: Is dat voor alle patiënten hetzelfde ook weer? Nogmaals, als we er even vanuit gaan dat alle patiënten verschillend zijn, wat je ook in het begin zei.**

R: Dat is zeker niet voor iedere patiënt hetzelfde. Met een patiënt die goed kan begrijpen wat mijn adviezen zijn en mijn argumentatie waarom ik tot een bepaald advies gekomen ben zou ik het sneller daarover hebben, omdat hij mijn argumentatie begrijpt. Maar soms is het heel moeilijk, vooral als het gaat om medicatie gerelateerde adviezen, om uit te leggen aan een patiënt waarom je voor bepaalde medicatie hebt gekozen.

**I: Ah zo, dan wordt het heel snel technisch.**

R: Ja, dan wordt het heel snel heel technisch. Hoe verhoudt dat zich tot het lichaam en waarom kies je voor dat bepaalde middel en niet dat bepaalde middel? Dat is soms heel moeilijk uit te leggen. Dan heb ik er wel moeite mee hoor.

**I: Dus dan is het eigenlijk afhankelijk van wat een patiënt heeft…**

R: Ik ga dan niet het hele technische verhaal uitleggen, waarom ik voor dat middel kies en niet voor dat middel. Ja. Terwijl ik dat soms bij mensen bij wie ik inschat dat ze dat wel begrijpen wel doe.

**I: Denk je dat het beter zou zijn als er meer gezamenlijke besluitvorming plaatsvindt?**

R: Ja, absoluut. Ja, dat denk ik wel.

**I: Zou je een voorbeeld kunnen geven waarbij er heel duidelijk sprake was van gezamenlijke besluitvorming? Ik ga dadelijk ook vragen naar een voorbeeld waarbij dat helemaal niet het geval was.**

R: Dan bedoel je met een casus die ik heb meegemaakt.

**I: Ja.**

R: Poeh, dat zijn er zoveel. Even denken.

**I: Ja, ik zag dat jullie meer dan zeshonderd consulten doen.**

R: Ja. Ja.

**I: Dat is echt veel, ja. Met zo’n klein team ook nog.**

R: Ja, precies. Het is maar een deel waarbij ik echt de patiënt zie, dus over gezamenlijke besluitvorming bij telefonische consulten kan ik het eigenlijk niet hebben, want daar doet de huisarts de gesprekken. Daarbij heb ik de patiënt zelf niet aan de telefoon. Maar hier in huis… Even denken hoor. Nou ja, gezamenlijk besluitvorming gaat bijvoorbeeld over een patiënt bij wie de thuissituatie eigenlijk onhoudbaar is door de aandoening, waarbij de mantelzorgers overbelast zijn en waarbij zij een keuze moet gaan maken, van, kan ze nog terug naar huis, is dat haalbaar, of moet ze naar een zorginstelling? Wij hebben alle mogelijke opties aan haar voorgelegd en haar tot bijna een week de tijd gegeven om daarover na te denken. Uiteindelijk heeft zij daar zelf wel een keuze in gemaakt. Ik denk dat dat een goed voorbeeld is van gezamenlijke besluitvorming. Dus ook mensen de tijd geven om over bepaalde keuzes na te denken. Natuurlijk, iedereen wil graag naar huis en dat was in eerste instantie haar reactie, maar ik ga naar huis! Uiteindelijk zei ze een week later toch van, nee, ik denk dat ik beter in een zorginstelling kan verblijven, want daar heb ik een betere kwaliteit van leven. Dus ik denk dat dat een goed voorbeeld is van een goede gezamenlijke besluitvorming. Je legt de keuzes voor en je laat haar daarover nadenken en niet haar tijdens hetzelfde gesprek de keuze laten maken. Ja. Dat is denk ik een goed voorbeeld daarvan. Ja.

**19.14**

**I: Zeker. En een voorbeeld waarbij dat juist niet gebeurde?**

R: Nou ja, er zijn genoeg voorbeelden waarbij er geen gezamenlijke besluitvorming is, omdat wij vaak patiënten treffen die wilsonbekwaam zijn, dus dan zegt de patiënt, dit en dit wil ik, in een verwardheid/een delier en dan ga je toch een ander beleid voeren. Dan is er dus totaal geen sprake van besluitvorming. Het wordt nog moeilijker als iemand dan geen wettelijk vertegenwoordiger heeft aangewezen.

**I: Ja, als er geen advanced care planning is of zo.**

R: Precies en als er niemand is die voor hem de keuze kan maken, van familie of naasten. Ja. Dat zijn wel voorbeelden inderdaad, dat je beslissingen neemt en die dus niet kan overleggen door de toestand van de patiënt.

**I: Mijn volgende vraag zou zijn, wat zou dan beter kunnen in dat soort gevallen?**

R: Goede advanced care. Ja. Ik denk dat het heel belangrijk is dat… Kijk, verwardheid kan bij iedereen voorkomen. Dat weet je van tevoren niet. Ik denk dat het gewoon goed is om inderdaad je wensen op papier te zetten en ook een medische vertegenwoordiger, die voor jou de keuzes maakt, dat je daarmee de besluitvorming regelt. Ja. Ja.

**I: De volgende vraag heeft te maken met allerlei belangrijke thema’s in de zorg voor patiënten. Even kijken hoor. De plaats van zorg en sterven. Ziekenhuisopnames. Crisissituaties. Wettelijke vertegenwoordiging in situaties van acute verslechtering. Wilsonbekwaamheid en levenseindebeslissingen. Levensbeschouwing en culturele achtergrond. Niet-behandelafspraken. Nu is mijn vraag of jij met behulp van een rangordening in deze kaartjes kan aangeven in hoeverre deze aan bod komen tijdens consulten met patiënten.**

R: Dus degene die bovenaan ligt moet het vaakst aan bod komen?

**I: Ja.**

R: Ik denk dat deze bovenaan komt.

**I: Plaats van zorg en sterven.**

R: Ja. Deze als tweede.

**I: Niet-behandelafspraken.**

R: Wat bedoel je precies met “crisissituaties”?

**I: Even kijken hoor.**

R: Bedoel je crisissituaties hier in het ziekenhuis of thuis?

**I: Ik denk dat thuis wordt bedoeld.**

R: Oké. Levensbeschouwing en culturele achtergrond als minste, denk ik. Ja. Die komt ook veel voor. Ziekenhuisopnames, dan gaat het er met name om of de patiënt nog opgenomen wil worden?

**I: Ja, volgens mij sluit dat aan bij het voorbeeld dat je net gaf.**

R: Ja. Ik denk dat het zo is. Ja.

**22.48**

**I: Even kijken hoor. Even voor de audio-opname. Plaats van zorg en sterven, niet-behandelafspraken, wilsonbekwaamheid en levenseindebeslissingen, ziekenhuisopnames, wettelijke vertegenwoordiging in een situatie van acute verslechtering, crisissituaties en levensbeschouwing en culturele achtergrond. Ja, nu is mijn vraag eigenlijk, die onderste kaartjes eigenlijk, die in de minste gevallen in consulten met patiënten in jouw geval ter sprake komen, die komen dus waarschijnlijk wel bij andere zorgverleners ter sprake, toch?**

R: Ik hoop het. Ik hoop het.

**I: Met welke zorgverleners zou dat dan…**

R: Dan hebben we het over de onderste drie hè?

**I: Ja. Dus die het minst voorkomen eigenlijk in jouw rangordening.**

R: Ja, die wettelijke vertegenwoordiging, ik hoop dat dat besproken is met de behandelend arts, bij wie de patiënt al langer onder behandeling is en met wie de patiënt dus ook de beste behandelrelatie heeft. Dat vind ik eigenlijk ook gelden voor crisissituaties. Ik denk dat dat vooraf besproken moet worden met de behandelend arts. Levensbeschouwing en culturele achtergrond; ik heb het nu helemaal onderaan staan, maar daarmee zeg ik niet dat het niet onze taak is, want ik vind dat het wel ook onze taak is om dat te bespreken. Meestal zie je dat mensen andere normen en waarden hebben, ook om dat duidelijk te maken tijdens bijvoorbeeld zo’n opname, omdat deze mensen misschien met een andere levensbeschouwing of culturele achtergrond andere keuzes maken op basis van… Maar ik vind dat onze taak. Ondanks dat we dat soms te weinig aankaarten vind ik dat ook onze taak, maar ik vind dat de taak van alle betrokken zorgverleners. Dus niet alleen van de arts, maar ook van de verpleegkundige en ook van de maatschappelijk werker en ook de diëtist.

**I: Dus als ik jou goed begrijp zouden de kaartjes die wat lager in de rangordening zijn eigenlijk bij iedereen met wie de patiënt te maken heeft ter sprake moeten komen.**

R: Ja. Ja. Ja. Zeker.

**I: Dat is duidelijk, met de kaartjes. Dan had ik nog een laatste onderdeel. Ik had ook nog een paar korte vragen over gezondheidsvaardigheden. Daar had ik het daarstraks ook met A. over en die zei, dit moet je echt dadelijk vragen aan J. Dus daar heb ik dadelijk wat vragen over.**

R: Is goed. Ja, prima.

**I: Even kijken, het laatste gedeelte gaat over beperkte gezondheidsvaardigheden. Heb jij weleens gehoord van die term, van het begrip?**

R: Nou, eigenlijk niet heel veel voordat ik in aanraking kwam met jullie onderzoek, met J. Toen was dat eigenlijk een soort bewustwording van, eigenlijk hebben we er wel heel veel mee te maken. Ik was er nog niet zoveel mee bezig. Pas toen ik het hoorde dacht ik, ja, inderdaad, veel mensen hebben beperkte gezondheidsvaardigheden en daar doen we eigenlijk niet zo heel veel mee. Dat dacht ik. Of, daar is verbetering… Dat denk ik.

**I: Daar zouden jullie meer mee kunnen doen.**

R: Ja. Ja.

**I: Daar kom ik zo even op terug.**

R: Ja, is goed.

**26.19**

**I: Wat betekent “beperkte gezondheidsvaardigheden” volgens jou?**

R: Beperkte gezondheidsvaardigheden, dat is dat patiënten moeite hebben om informatie in zich op te nemen die ze bijvoorbeeld van de arts krijgen, om te kunnen filteren of prioriteren en ook te kunnen toepassen bij hen in de praktijk, thuis of waar ze ook verblijven.

**I: Ja, precies. Dat sluit heel mooi aan bij onze definitie inderdaad, dus dat is mooi. Als ik even mag inhaken op wat je net zei, hoe vaak hebben jullie te maken met patiënten met beperkte gezondheidsvaardigheden?**

R: Ik denk heel vaak en dat we het zelf bij sommige mensen niet eens door hebben. Dus ik denk vaker dan dat wij zelf door hebben. Ja.

**I: Hoe herken je patiënten met beperkte gezondheidsvaardigheden?**

R: Poeh.

**I: Dat je het niet door hebt, geef je al aan.**

R: Ja. Hoe herken ik hen? Meestal deels aan het taalgebruik van de patiënt tijdens de gespreksvoering.

**I: Dat gaf je inderdaad al aan, tijdens dat eerste consult wat je dan hebt met mensen.**

R: Kijk, mensen kunnen het hebben over hun medicatie. De een kan dat perfect verwoorden en de ander zegt, dat blauwe pilletje, dat oranje pilletje, dat soort dingen. Daar pik ik het wel aan op. En als patiënten zelf zeggen van, wat de arts net verteld heeft, ik weet het niet meer. Dan geven ze het soms zelf aan. “Niks van begrepen, van wat hij net verteld heeft.”

**I: Die is wel heel duidelijk.**

R: Die is duidelijk.

**I: Ja, precies. Dus stel, je herkent zo iemand in een consult… Je gaf al aan van, als je dan een vervolgconsult hebt, ga je dus dingen aanpassen.**

R: Ja.

**I: Maar wat pas je dan aan? Op welke manier houd je rekening in de communicatie met die mensen?**

R: Ik probeer niet te veel onderwerpen te bespreken in één gesprek, dus dat wordt verspreid over verschillende gesprekken. Als de mogelijkheid er is. Kijk, als ze snel met ontslag gaan heb ik die mogelijkheid niet. Maar als ze hier langer opgenomen liggen probeer ik dat te verdelen, dus dan houd ik het bij één onderwerp per gesprek. Zoals ik eerder ook zei, wil ik dat weleens met tekeningen verduidelijken of dat ik het opschrijf als reminder, zodat ik het bij hen kan laten liggen.

**I: Ervaar je ook barrières in de zorg aan dit soort patiënten?**

R: Ja. Zeker. Ik denk dat de manier van communiceren met de patiënt van verschillende zorgverleners op één lijn moet liggen en dat dat soms moeilijk is, als je begrijpt wat ik bedoel.

**I: Bedoel je dan dat bijvoorbeeld de arts op eenzelfde manier communiceert als jij bijvoorbeeld?**

R: Ja. Ja. Dat we daar eigenlijk vooraf voor-bespreken van, wat gaan we die patiënt uitleggen en hoe? En dat je dan pas inderdaad de patiënt benadert. Dat gebeurt niet, natuurlijk niet. De drukte van het ziekenhuis maakt dat… Dan kom ik bij de arts binnen, een uur later komt de arts, die loopt langs de patiënt. Die vertelt dat, ik vertel dat. Ja. Ik denk dat als je multidisciplinair eerst samenkomt en daar ook afspraken over maakt, van, hoe benader je zo’n patiënt, dat mogelijk oplevert dat de patiënt het beter begrijpt en daardoor ook misschien meer trouw is aan zijn therapie. Ja.

**30.29**

**I: Inderdaad. Even kijken hoor. Dus zo’n barrière is dus dat je, inderdaad wat je aangeeft, multidisciplinair werkt en dat je dus allemaal een andere soort, een andere wijze van communiceren hebt. Toch?**

R: Ja. Ja.

**I: Dus jij zou zeggen van, nou, dat zou eigenlijk wel beter kunnen aansluiten. Zijn er nog meer dingen die beter kunnen, zodat de zorg beter aansluit bij dat soort patiënten?**

R: Dat vind ik moeilijk om nu zo één, twee, drie te bedenken, hoe de zorg beter kan.

**I: Het voorbeeld dat je gaf vond ik al heel mooi, dat je zegt van, dat we eigenlijk allemaal hetzelfde praten met die patiënt, dat dat al duidelijker zou zijn.**

R: Ja.

**I: Dat vond ik al een heel fraai voorbeeld.**

R: Ja. Ja. Als me iets te binnenschiet, dan zeg ik het.

**I: Ja, precies. Ik had nog een aantal vragen waarvan A. zei van, hé, dat moet je eventjes aan L. vragen. Is er beleid binnen het ziekenhuis om zorg toegankelijker te maken voor mensen met beperkte gezondheidsvaardigheden?**

R: Niet dat ik weet. Niet dat ik weet.

**I: Dat is hetzelfde antwoord als wat A. gaf ☺.**

R: Misschien is het er, maar dan heb ik er geen weet van. Ja.

**I: Zijn er dan bepaalde methodieken of instrumenten om de zorg toegankelijker te maken voor mensen met beperkte gezondheidsvaardigheden?**

R: Niet naar mijn weten, ook niet. Nee. Nee. Er zijn bijvoorbeeld niet bepaalde checklists of wat dan ook, waarvan ik weet, die het makkelijker maken voor hen. Nee. Nee.

**I: Dat is heel duidelijk.**

R: Ja.

**I: Heb je nog aanvullingen of vragen over het interview?**

R: Nee, ik vond het eigenlijk wel duidelijk.

**I: Ja?**

R: Ja. Ja.

**[33.39 – einde opname]**

Z1long03

I: interviewer

R: respondent

**I: Ja, ik wil in het interview wat vragen over uw rol als zorgverlener in de palliatieve zorg, over communicatie met uw patiënten en over beperkte gezondheidsvaardigheden. Dat zijn eigenlijk een beetje de hoofdthema’s waar ik het graag met u over zou willen hebben. Heeft u nog vragen tot zover?**

R: Nee, laat maar komen.

**I: Wat is uw rol in de zorg voor patiënten in de palliatieve fase?**

R: Om maar even met een makkelijke vraag te beginnen.

**I: Ja, we beginnen breed inderdaad.**

R: Zorgen voor een optimale kwaliteit van leven. Zorgen voor goede uitleg en begeleiding. Misschien wel overdragen aan de huisarts, want het is maar de vraag of je dat allemaal in de tweede lijn moet willen doen. Huisartsen hebben een palliatief team, investeren veel in palliatieve zorg. Dus misschien wel het moment bepalen dat de huisarts er beter mee verder kan dan de medisch specialist. Maar verder is het vooral oog houden voor de kwaliteit van leven en kijken of je daar zoveel mogelijk aan kunt bijdragen.

**01.25**

**I: U noemde al de huisarts, maar zijn er bijvoorbeeld binnen het ziekenhuis; hoe werkt u dan samen met andere zorgverleners?**

R: Nou, we hebben het palliatieve team.

**I: Daar heb ik al mensen van gesproken inderdaad.**

R: Die helpen en denken soms gevraagd en soms ongevraagd mee voor mensen die opgenomen zijn en opgenomen blijven of voor mensen die naar huis moeten en waar iets mee moet. Dus die helpen daar ook bij. Het is even de vraag wie dan de hoofdbehandelaar is of blijft. Ik denk dat patiënten er prijs op stellen om bij hun eigen specialist te blijven, maar zo’n palliatief team heeft natuurlijk ook een eigen autonomie en een eigen verantwoordelijkheid, dus die doen soms ook dingen die zij goed achten. Maar in ieder geval, bij de opgenomen patiënten is dat palliatieve team denk ik heel actief. Bij de poliklinische patiënten volgens mij veel minder. Ik denk dat je dat onderscheid wel even moet maken.

**I: Bedoelt u dan opgenomen bij u op de afdeling bijvoorbeeld of in het algemeen opgenomen in het ziekenhuis of poliklinisch, dat verschil?**

R: Ik heb het nu maar even over longpatiënten en dan de mensen die bij ons op de afdeling opgenomen zijn en de mensen die wij op de poli zien. Voor poliklinische patiënten is het denk ik nog meer zorgen hier dat er een goed overleg met de huisarts is, want die mensen zijn thuis. En dan is het denk ik goed om te weten dat huisartsen daar al heel veel meer in doen dan een aantal jaren geleden. Niet alleen stervensbegeleiding, want dat doen ze natuurlijk toch al, maar ook de fase daarvoor, zicht hebben op kwaliteit van leven, symptoombestrijding, dat ze daar een pro-actievere rol in spelen.

**I: Even voor mijn beeld, dus als longarts; de centrale zorgverlener, is dat… U gaf aan ten aanzien van de centrale zorgverlener dat mensen het dan fijn vinden als dat dan de specialist is, in dit geval.**

R: Ja.

**I: Bent u dan als longarts ook weleens centrale zorgverlener van patiënten?**

R: Hoofdbehandelaar. Ja.

**I: Hoofdbehandelaar, ja.**

R: Ja. Dat is nog weleens verwarrend, want dan denken huisartsen misschien, ja, maar die is bij de specialist, dus daar gaat de specialist over. Terwijl ze maar heel weinig bij mij komen. Poliklinische patiënten komen af en toe eens een half uurtje langs, maar die zitten natuurlijk het grootste deel van de tijd bij de huisarts of in ieder geval thuis en je zou willen dat de huisarts zich daar ook verantwoordelijk voor voelt. Dat gebeurt overigens ook hoor. Het is geen verwijt, maar om aan te geven dat het meer voor de hand ligt dat voor poliklinische patiënten de huisarts een voorname rol speelt dan dat het van de specialist komt. Een belangrijke is al het herkennen van de fase; wij moeten nu gaan praten over de palliatieve fase. We hebben natuurlijk heel lang de neiging om mensen te genezen. Als je wat langer medisch specialist bent dan realiseer je je dat dat lang niet altijd lukt en dat patiënten allemaal doodgaan, maar het moment herkennen dat je in die fase komt en dan niet de indruk geven dat je het hoofd in de schoot legt, maar dat je op een andere manier voor mensen moet gaan zorgen dan nog maar weer een kuur, nog maar weer dit en nog maar weer dat, noem het maar op allemaal; die fase herkennen is vaak het lastigste. We hebben natuurlijk als medisch specialist een beetje overspannen verwachtingen van onze uitkomsten. We denken natuurlijk allemaal dat we mensen beter kunnen maken.

**I: Nou ja, dat is ook vaak zo, denk ik, toch?**

R: Nou, dat is niet altijd zo. Bij COPD niet en bij longkanker niet en bij pulmonale hypertensie niet. Een heleboel mensen gaan natuurlijk dood aan een longaandoening, ondanks het feit dat die dokter en misschien ook wel de patiënt zelf verschrikkelijk hun best doen om dat te voorkomen. Dus de fase herkennen waarin je dat soort zaken moet gaan bespreken, waarbij je je niet langer moet richten op curatie, maar op palliatie of zorg, is vaak een hele lastige. In de oncologie; maar daar kun je nog zeggen, uitbehandeld, ik heb niks meer. Uitbehandeld is in de oncologie een bekend begrip, maar uitbehandeld bij COPD of uitbehandeld bij chronische ziekten is veel moeilijker.

**I: Waarom is dat?**

R: We hebben wel iets voor die mensen. We kunnen hun een beetje zuurstof geven, we kunnen hun nog maar weer een pufje extra geven, we kunnen hen nog eens een keer naar de fysiotherapeut sturen. Dus in de zin van, we hebben nog wel wat voor je, maar eigenlijk is dat gewoon achter de feiten aanlopen of dweilen met de kraan open of zijn de opbrengsten van dat soort inspanningen al te voorspellen heel gering. Dus het herkennen van een palliatieve fase bij kankerpatiënten is vaak iets makkelijker, want dan is het gewoon, nou hebben we echt niks meer, we kunnen niet meer bestralen, we hebben geen kuren meer, we moeten ons erbij neerleggen dat de kanker het gaat winnen. Dat is bij kanker vaak makkelijker dan bij een chronische ziekte. Nou, ik doe geen kanker, dus bij mij zijn het inderdaad vooral chronische aandoeningen, zoals COPD. Die mensen gaan in de loop van tien jaar heel geleidelijk aan achteruit, totdat het echt niet meer gaat. Dan is er dus een fase waarin je over het palliatieve traject zou moeten praten. Dat herkennen en bespreekbaar maken, zonder dat die patiënt de indruk heeft, ik ben opgegeven, of, ze doen niks meer voor me, of, ze kunnen niks meer voor me doen; palliatieve zorg is wel iets voor mensen doen. Ik bedoel, we kunnen heel waardevol zijn, maar als de patiënt het idee heeft, ik ben opgegeven en daar is niets meer aan te doen; dat is natuurlijk iets heel anders dan, we gaan ons nu richten op kwaliteit van leven en niet per se op langer leven.

**07.34**

**I: Dat is voor mij nog niet helemaal helder - dat komt door mij hoor - maar waarom is het moeilijk om dat te herkennen, die palliatieve fase?**

R: Misschien omdat er niet eens een goede definitie van is en misschien omdat het een hele geleidelijke achteruitgang is.

**I: Wat u zegt over die tien jaar bijvoorbeeld.**

R: Ja. Prik dan maar eens het moment van, ja, nu had ik er eigenlijk over moeten beginnen. Meestal realiseer je je dat pas achteraf. Als iemand aan de zuurstof komt bijvoorbeeld; een COPD-patiënt, gaat geleidelijk achteruit, heeft een zuurstoftekort ontwikkeld en die gaan we zuurstof geven. Eigenlijk is dat een vorm van palliatieve zorg, want het is symptoombestrijding zonder dat je hem er nou echt mee geneest. Maar we zien het wel als een interventie, we zien het wel als therapie en in die zin zien we het dus niet als palliatief. Hetzelfde bij extreme dyspnoeklachten. Sommige mensen zijn echt Spaans benauwd. Dat kun je bestrijden met morfine, want dat is een prima middel tegen kortademigheid. Maar morfine is ook een heel beladen geneesmiddel. Op het moment dat je iemand met ernstige kortademigheidsklachten morfine geeft als symptoombestrijding, dan gaat er onmiddellijk bij die patiënt een lampje branden, morfine is einde oefening. Dat zou dus een moment kunnen zijn dat je over de palliatieve fase spreekt. Maar eigenlijk is iedere chronische ziekte waar je uiteindelijk aan kunt overlijden; ja, dan ben je palliatief bezig. Kwaliteit van leven – misschien ook wel een klein beetje langer leven – wordt natuurlijk geleidelijk aan steeds belangrijker. Als je twintig bent wil je nog vele jaren meegaan, maar als je tachtig bent wil je vooral niet akelig zijn. Nou, dat is dan denk ik maar iets palliatiefs. Heb ik het zo een klein beetje kunnen schetsen?

**I: Ja, zeker, zeker. Ik wilde graag wat meer vragen gaan stellen over het communiceren met de patiënt. Ik heb het idee dat we het nu vooral over het fysieke hebben gehad, maar er is natuurlijk ook het psychische, sociale en spirituele welzijn van de patiënt. In hoeverre komen deze verschillende aspecten aan bod in uw gesprek met patiënten?**

R: Heel weinig. Veel te weinig. Ja.

**I: Oh, heel weinig. En dan bedoelt u de aspecten, behalve het fysieke? Wat bedoelt u dan?**

R: Ja, we praten wel over de technische aspecten, de pufjes en de pilletjes en de zuurstof en de fysio en misschien zelfs wel de getalletjes van de longfunctie of de getalletjes van de bloeduitslagen. Maar alleen al de vraag, wat verwacht u eigenlijk van mij, of, wat denkt u zelf hoe het het komende jaar met u gaat, of, bent u er eigenlijk nog wel over een jaar, verwacht u er over een jaar nog te zijn… Het consult zoals we dat nu hebben van acht minuten – mijn consulten duren gemiddeld acht minuten – is daar niet geschikt voor.

**I: Nee, dat lijkt me te kort.**

R: Op het moment dat je zo’n vraag stelt, weet je al op voorhand dat dat even gaat duren. Maar eigenlijk zou je dat moeten doen. Eigenlijk zou je moeten zeggen, ik vind dat het achteruitgaat, dat merkt u ook, we moeten eens even gaan nadenken over de nabije toekomst. Een enkele keer doe je dat wel en een enkele keer lukt het wel. Een enkele keer komt een patiënt er zelf mee, maar er zijn niet veel mensen die zich afvragen, hoe gaat het eigenlijk met mij? Eén van de problemen is dat die patiënt helemaal niet voorbereid naar het consult komt. Het mooiste zou natuurlijk zijn dat je als patiënt denkt, nou, ik ga morgen naar de longarts of naar de hartspecialist, heb ik eigenlijk vragen, hoe gaat het eigenlijk met mij? Die dokter gaat straks vragen, hoe gaat het met u?

**I: Ja, dat is vaak de beginvraag.**

R: Dat is vaak de beginvraag. Daar komt bijna altijd het foute antwoord op, want dan zeggen ze namelijk, goed. Maar als je dan even doorvraagt – en dan moet je toch echt wel even echt doorvragen – dan blijkt dat het helemaal niet goed gaat. Dat “goed” is beleefdheid.

**I: Ja, daar moet je eigenlijk even doorheen.**

R: Maar het is geen adequaat antwoord. Nou, voordat je daar mee verder bent; het is natuurlijk veel fijner als de patiënt komt met, doc., het wil helemaal niet en volgens mij komt het ook niet meer goed. Nou, dat heb ik nog nooit iemand horen zeggen. Dus het moet in die zin toch van de zorgverlener komen om dat gesprek aan te gaan en de eerlijkheid gebied te zeggen dat een consult van acht minuten daar natuurlijk niet de ideale setting voor is.

**I: Nee, dat lijkt mij te kort. Ik zou het niet weten, maar…**

R: Ja, dat is het natuurlijk ook. Ja. Ik kan snel communiceren, ik kan snel een ‘rapport’ (Franstalige uitspraak) maken met een patiënt – de een kan dat makkelijker dan de ander – maar even snel dit ei kraken en uitbroeden in de zin van, wij moeten nu echt eens gaan nadenken over kwaliteit van leven en niet over honderd worden; ik zie dat er nu in een consult niet vaak van komen. Veel te weinig. Daar doen we patiënten tekort mee, dus dat zou je eigenlijk anders moeten organiseren, in de zin van: wij moeten nu eens wat langer praten, kom morgenmiddag even terug, want tussen half vier en half vijf heb ik er wel tijd voor.

**12.59**

**I: Dus dan zou je eigenlijk meer tijd willen.**

R: Ja. Of je besteedt het uit. Je zegt, ik zou graag willen dat u nou toch eens praat met de… Nou, dan komt het. Maar dat zou al gauw afschuiven van de verantwoordelijkheid kunnen betekenen. Maar we hebben natuurlijk verpleegkundigen die dat prima zouden kunnen, die dichter bij de patiënt staan, minder bedreigend zijn, wat meer tijd hebben om goed door te vragen. Een palliatieve zuster, daar zou je enorm je voordeel mee kunnen doen, maar dat levert geen geld op, dus dat is in zo’n ziekenhuis niet zomaar aan te stellen. Maar ik denk dat de zorg er zeker beter van wordt. De vraag is of je zomaar palliatieve zusters vindt, want het is natuurlijk wel slopend om voortdurend met mensen te praten over, nou is het echt gedaan en hoe gaan we het nou nog een beetje gezellig houden?

**I: Ik dacht eigenlijk dat u ging zeggen, het palliatieve team erbij betrekken. Maar dat werkt dus niet zo?**

R: Die hebben waarschijnlijk ook maar een beperkte capaciteit. En bij poliklinische patiënten; dat zit in ieder geval nog niet in de routine, dat we hen daar ook voor kunnen gebruiken.

**I: Aha, dus het is echt alleen voor mensen die hier opgenomen worden.**

R: Ja. En als je hier opgenomen bent, dan kunnen we op de rand van het bed gaan zitten. Dan kun je nog eens zeggen, ik heb nou even tijd, we moeten praten. Of, laat de kinderen eens even komen. Of, laat de partner eens even komen. Als je in je eentje op je drieëntachtigste bij mij op de poli zit en je had dat gesprek eigenlijk graag met een schoondochter of met een zoon willen voeren, dan is de kans alweer voorbijgegaan. Maar van tevoren zeggen, u komt volgende week bij mij op de poli, neem eens even uw zoon mee; ja, dan ben ik aan de telefoon al een kwartier aan het uitleggen wat ik wil gaan bespreken. Dan zeggen ze, heb ik dan kanker of zo? Waarom moet mijn zoon dan meekomen, want die heeft een baan en die heeft wel wat anders te doen.

**I: Die aspecten die u aangeeft; eigenlijk te weinig - als ik iets verkeerd zeg, moet u mij gewoon verbeteren natuurlijk hè - maar u geeft eigenlijk aan, eigenlijk te weinig, daar doen we de patiënt wellicht mee tekort. Maar is dat eigenlijk bij alle patiënten zo hetzelfde, hoe die aspecten ter sprake komen?**

R: Nee, dat is niet bij alle patiënten hetzelfde. Dat hangt af van de soort patiënt. Sommige patiënten geven zelf een beetje een opening en bij andere patiënten komt het helemaal niet in hen op om daarover na te denken of om daarover te praten. Het ene spreekuur is nog weer wat drukker dan het andere spreekuur, dus soms heb ik er wel een klein beetje tijd voor en soms echt helemaal niet. Per aandoening kan het verschillen. Wat ik zei, kanker of chronische ziekten. Ik denk dat in de oncologie, in de kankerzorg, dat soort gesprekken explicieter gevoerd worden, misschien ook wel met een rol voor de verpleegkundige. Bij COPD, wat een andere voorname doodsoorzaak binnen mijn vak is, is dat voor mijn gevoel veel minder.

**15.56**

**I: Verschilt dat dan bij mensen die bijvoorbeeld wat lager opgeleid zijn of die niet zo heel veel lijken te snappen? Dus de wijze waarop u die aspecten ter sprake brengt, verschilt dat bij dat soort patiënten misschien?**

R: Ja. Ik denk dat er collosale verschillen zijn. Ik had een hele intelligente jazzmusicus en die heb ik eens een keer gevraagd, wat kan ik eigenlijk voor u betekenen? Toen zei hij, ik wil maar twee dingen, ik wil kunnen roken en ik wil naar jazzmuziek luisteren. Voor de rest hoef je niets voor mij te betekenen. Nou, dat vond ik een verfrissend concreet plan en daar kon ik wel iets mee.

**I: Het lijkt me ook een ingewikkeld plan…**

R: Nee, het was echt leuk. Die man is inmiddels overleden, het maakt niet uit hoe, maar in ieder geval, dat was een fijn contact. Ik wist precies wat die man wilde en hij wist ook wel zo ongeveer wat ik wel en wat ik niet voor hem kon betekenen. Ik hoefde ook niet meer over dat roken te zeuren, want hij wilde helemaal niet stoppen. Dat was namelijk één van zijn behandeldoelen, blijven roken. Het was fijn dat dat zo helder was geformuleerd, want dan hoef ik ook niet te gaan trekken aan een dood paard. Als ik zou vragen, wat zou u eigenlijk het komende jaar allemaal nog willen doen, of, wat kunt u nu niet meer wat u twee jaar geleden nog kon, of, bent u er eigenlijk nog over twee jaar, wat best aardige vragen zijn om het gesprek te openen, maar ik denk dat bij veel patiënten daar geen al te diepgaand antwoord op komt. Wat denkt u zelf, dokter? Of, u bent toch de dokter? Of, ik wil het helemaal niet weten. Dat kan natuurlijk ook nog. Een heleboel mensen willen dat niet weten. Als ik zeg, wat vindt u nou belangrijk, dan zeggen ze wel allemaal, ik wil niet kortademig zijn, ik wil de trap op kunnen. Nou, nog niet eens zozeer de trap op kunnen, maar, ik wil niet kortademig zijn. Nou, als dat het behandeldoel is, minder kortademigheid, en ik kan dat niet bereiken met mijn pufjes en mijn pilletjes en mijn andere flauwekul, dan moet ik ook durven zeggen, ja, het spijt me reusachtig, maar dat zit er nou eenmaal niet in, het is zoals het is. Dan kun je gaan praten over, hoe kunnen we het nou nog zo gezellig mogelijk houden? Dat soort vragen stellen; het is betrekkelijk makkelijk om die vragen te verzinnen, maar je moet wel het contact hebben met de patiënt om die opening te kunnen maken, je moet ook de tijd hebben om met het antwoord wat te doen en je moet ook nog wel een beetje de communicatieve vaardigheden hebben om het in goede banen te leiden. Dan moet er het liefst ook nog een familielid bij zijn die met je meedenkt, want bij een tweeëntachtigjarige die drie jaar lagere school heeft gehad wordt dit natuurlijk een lastig gesprek. Dan is het fijn als er een zoon is die zegt, ja, maar pa, je kunt toch niks meer, je zit toch alleen maar de hele dag op de bank, je vond het toch al zo jammer dat je niet meer de trap op kon, je hebt het bed beneden staan en het gaat allemaal niet meer. Daar heb je bijna een ander voor nodig om dat boven tafel te krijgen. De meeste patiënten willen natuurlijk ook gewoon met rust gelaten worden. Eén van de dingen die wij bereiken is dat mensen zich schuldig voelen of dat mensen zich insufficiënt voelen. Als ik zeg, je zou meer moeten bewegen, dat gaat helemaal niet, want die mensen zijn Spaans benauwd. Dan proberen ze te bewegen en dan worden ze meer kortademig. Ja, dat is wel een hele lastige natuurlijk. Dus die patiënt denkt, als je mij nou maar gewoon met rust laat, dan word ik niet kortademig. Even om aan te geven dat zo’n gesprek met, wat doen we dan met de antwoorden en wat wilt u zelf, nou eenmaal een hoop tijd kost en ook wel wat gespreksvaardigheden vergt. Ja, dat gaat bij de ene patiënt echt veel makkelijker dan bij de andere.

**19.59**

**I: Als ik mag doorvragen; u gaf aan dat u op een gegeven moment contact moet maken met de patiënt en dat je binnen moet komen, maar hoe werkt dat dan? Hoe maakt u dan bijvoorbeeld contact?**

R: Daar heb je die filmpjes voor gemaakt.

**I: Ja, dat klopt, maar ik ben nou nieuwsgierig naar hoe u dat…**

R: Als je elkaar voor de eerste keer ziet, dan wil dat nauwelijks. Je moet elkaar toch een paar keer gezien hebben. Je moet ook een beetje een inschatting kunnen maken, van, hoe kijkt een patiënt naar zichzelf of hoe kan een patiënt naar zichzelf kijken? Je moet ook een beetje het vertrouwen hebben in de manier waarop je dingen vraagt en waarom je dingen vraagt, dus je moet elkaar een paar keer gezien hebben en dat moet ook een heel klein beetje klikken. Ik heb patiënten bij wie het helemaal niet klikt. Die zouden misschien beter af zijn bij een andere dokter. Dat kan natuurlijk. Misschien dat het daar beter gaat. Jonger of vrouw of geduldiger, het kan me niet schelen. Maar het gebeurt dus al heel weinig, dat je echt dat gesprek aangaat of dat je er echt over begint en dat heeft dus met een heleboel factoren te maken, ik wil niet te veel uitlopen, ik heb met deze patiënt nog niet de band dat ik dat makkelijk bespreekbaar kan maken, ik verwacht niet dat deze patiënt daar zelf al een beetje mee bezig is of over nagedacht heeft of snapt waar ik naartoe wil, zonder het meteen als een bedreiging te zien of als, nou ben ik natuurlijk blijkbaar opgegeven, ze kunnen niks meer voor me doen en ik ga in de put zitten. Dat gesprek komt maar weinig op gang. Het gebeurt dus minder dan ik graag zou willen, om de redenen die ik zojuist een beetje heb geprobeerd te schetsen.

**I: Als ik ook daar op mag doorvragen, we hebben nu die aspecten besproken eigenlijk hè? Maar als u dan kijkt naar communicatie in het algemeen, zijn er dan ook patiënten bij wie u de communicatie echt moeilijker vindt?**

R: Ja, dat is bij ongeveer vijfenzeventig procent van mijn patiënten zo, omdat we niet dezelfde taal spreken of omdat het begrip over en weer een klein beetje ontbreekt, of omdat ik de vaardigheden mis om het bij iedere individuele patiënt allemaal vlot in goede banen te leiden, of omdat die patiënt nou eenmaal niet in staat is om daar rationeel mee om te gaan zonder angsten of spanningen of noem het maar op allemaal, of verdriet. Dus ik denk dat er nogal wat patiënten zijn bij wie het niet vanzelf gaat. Dat is dus voor een deel mijn skills en mijn geduld en mijn tijd, maar het is voor een deel ook wel gewoon het begrip of gezondheidsvaardigheden, laat ik dat dan toch maar zeggen, aan de kant van de patiënt zelf. Daarom is het ook zo belangrijk dat een familielid meekomt, omdat die natuurlijk ook de patiënt een beetje een spiegel voor kan houden, van, ja, dat zeg je nou wel, pa, maar dat is helemaal niet zo.

**I: Ja, om toch die dingen boven te krijgen die u eerder aangaf.**

R: Ja. Ja. Ja.

**I: Behalve zo’n naaste die er dan bij zit, wat doet u dan verder om de communicatie te verbeteren?**

R: Ja, misschien geduldig blijven. Misschien het op een andere manier nog eens proberen. Of misschien wel gewoon het gesprek uit de weg gaan, want het komt er vaak niet van. Misschien wel omdat ik denk, nou, dat wordt toch niks, ik ga het maar uit de weg. En ik vind het echt een gemis dat het zo weinig gebeurt, om dat bijtijds en goed met elkaar te bespreken. Ik realiseer me dat een deel daarvan het gevolg is van mijn beperkingen of tekortkomingen, van mijn part is het mijn schuld, het kan me niet schelen hoe je het noemt. Dus het is deels, ik blijf in gebreke. Maar het is waarschijnlijk ook voor een deel een patiënt die er niet zelf mee komt, een patiënt die er niet zelf over nagedacht heeft, een patiënt die de urgentie ervan niet snapt of die je juist afschrikt doordat je zegt, nou moeten we toch eens een keer goed praten, dat schiet allemaal niet op, het gaat alleen maar verder achteruit en volgens mij bent u er over een jaar niet meer. Nou, er zijn nogal wat patiënten die dan onmiddellijk klaar zijn en niet zeggen, leg dat eens even uit, waarom denk je dat en wat kan ik dan over een jaar niet meer, wanneer denkt u dan dat ik doodga? Nou, dat soort concrete vragen komen er zeker niet. Het is onmiddellijk angst of verdriet of blokkeren. Dus je hebt ook wel gespreksvaardigheden nodig om toe te lichten waarom je dingen vraagt en wat het belang is van goede antwoorden, dat je mensen daar een handje bij moet helpen.

**24.52**

**I: Zijn er ook momenten dat u bewust de communicatie aanpast?**

R: Ja.

**I: Hoe gaat dat dan? Kunt u daar iets meer over vertellen?**

R: Nou, misschien toch door langzamer te praten of misschien nog eens een keer een nieuwe afspraak te maken om er even wat langer op door te gaan of door een familielid erbij te betrekken of door toch maar gewoon in Jip en Janneketaal te proberen dingen uit te leggen, maar dan wordt het minder concreet. Het is natuurlijk heel fijn als je kunt zeggen, volgens mij bent u er over een jaar niet meer en we gaan daar nu even samen over sparren.

**I: Ja, dat is bijzonder concreet.**

R: Dat is heel concreet. Dan gaat het ook lekker vlot.

**I: Maar dat werkt niet?**

R: Bij de overgrote meerderheid van de patiënten hoef ik niet aan te komen met de opmerking, volgens mij bent u er over een jaar niet meer, want dan komt er geen gesprek meer op gang.

**I: Ja, wat u aangeeft, dan blokkeert het en dan…**

R: Dan blokkeert het. Dus soms komt er nog eens een familielid terug de kamer in, als opa net naar de balie gegaan is, van, wat denkt u eigenlijk, doc., hoe gaat het met hem? En als ik dan zeg, volgens mij is hij er over een jaar niet meer, dan zegt zo’n schoondochter, dat zie ik zelf ook wel, dat weten wij zelf ook wel, maar hoe moet het dan verder? Nou, dat is fijn, want dan heb je een opening. Maar als opa daar zelf zit en liever met rust gelaten wil worden en ook de confrontatie uit de weg wil gaan, volgens mij ben je er over een jaar niet meer, want opa wil graag die confrontatie uit de weg gaan… Een heleboel mensen willen dat namelijk helemaal niet weten. Dus dan komt het gesprek eigenlijk niet goed op gang. En dan spelen we dus een heel klein beetje stommetje naar elkaar toe, over en weer, van, er is iets wat u zou moeten weten en er is iets wat ik met u zou moeten bespreken en wij leggen ons erbij neer dat het niet gebeurt.

**I: Checkt u ook weleens bij patiënten of zij het zelf hebben begrepen?**

R: Ja. “Heeft u begrepen wat ik u heb uitgelegd?” Ja, zeggen ze dan. Hoewel sommige mensen denken, waarom vraagt hij dat, zou hij denken dat ik dom ben? Dus dat is ook wel weer een vervelende. “Kunt u nog eens in het kort samenvatten wat ik u verteld heb?” Dat zou een hele mooie zijn, maar daar zit ook weer in, volgens mij snap jij het niet. Dus hoe stel je die vraag? “Ik heb u heel veel verteld, ik zou willen dat u dat allemaal opgeslagen heeft en dat u dat goed gehoord heeft. Kunt u even een korte samenvatting voor mij geven?” Dat zou een hele mooie zijn. Maar dan komen er wel weer twee minuten bij, bij dat consult, en dat was al een beetje krap.

**I: Ja, dan zit je toch weer aan die acht minuten die…**

R: Dus wat zeg ik? “Snapt u wat ik u verteld heb?” En als het antwoord dan “ja” is, terwijl ik aan mijn water voel dat het waarschijnlijk “nee” is, dan vind ik het goed. Maar het is natuurlijk een klein beetje een schijnzekerheid. Het mooiste is, vat het eens even kort samen.

**I: Ja, dat je dat aan de patiënt vraagt.**

R: Of aan een familielid. Want als dat familielid dat kan, dan komt het thuis goed, want dan kan die nog eens zeggen, wat de dokter verteld heeft… Dus dat samenvatten of kort reproduceren zou behulpzaam kunnen zijn. Dat doe ik dus heel weinig. Ook wel omdat wij ons verschuilen achter de technicalities van de pufjes en de pilletjes en de getalletjes. Dat echte gesprek van, hoe staat het er nou voor en hoe gaat het er de komende jaren uitzien, komt er maar heel weinig van. Het kan best zijn dat andere dokters – ik praat voor mezelf – dat veel meer doen dan ik, maar ik doe het in ieder geval te weinig.

**28.50**

**I: Mijn persoonlijke observatie is dat u heel eerlijk bent en dat dat heel fijn is, dat u steeds aangeeft dat u dat eigenlijk te weinig doet. Ik zat ook te denken, bijvoorbeeld het concept gezamenlijke besluitvorming, in hoeverre denkt u dat daar sprake van is in de palliatieve fase, bij patiënten in de palliatieve fase?**

R: Misschien wel meer in de palliatieve fase dan in het curatieve traject. Ik geloof niet in shared decision making, dat zeg ik er dan nu ook maar even heel eerlijk bij. De echte kennis en de echte inzichten in meedenken en meebeslissen, dat is een hele lastige. Veel mensen zeggen toch iets in de geest van, wat zou je zelf doen, wat zou je doen als het je vader was? Het zou wel helpen als je zegt, wat kan ik voor u betekenen, waar bent u op uit als patiënt? Genezing of kwaliteit van leven of zo lang mogelijk leven of van de pijn afkomen of van de kortademigheid afkomen. Dan zal je merken dat sommige patiënten dingen belangrijk vinden die eigenlijk heel anders zijn dan wat ik gedacht zou hebben. Ik ben namelijk van de getalletjes en van de longfunctie en van de dingetjes, terwijl die patiënt denkt, het interesseert me geen donder, die longfunctie, ik ben kortademig en daar wil ik van af. Of, ik ben kortademig en als het niet beter wordt, dan hoeft het voor mij niet meer. Dat kan natuurlijk ook nog. Dus ik denk dat shared decision making… Het kost tijd. Het veronderstelt van de kant van de patiënt ook een klein beetje kennis en inzicht, wat er niet altijd is. Het veronderstelt skills van de zorgverlener, die er niet altijd zijn. Wij zijn natuurlijk toch tamelijk paternalistisch opgeleid. Die patiënt komt met een probleem, ik zeg wat er moet gebeuren en als het een beetje meezit dan doet die patiënt dat en dan levert het ook nog wat op. Juist in het palliatieve traject gebeurt nogal eens, ik wil niet meer opgenomen worden, ik wil niet meer naar … (naam revalidatiecentrum) om te revalideren. “Waarom niet?” “Ja, het is het me niet waard, ik wil met rust gelaten worden.” Ik denk dat in die fase shared decision making of gezamenlijke besluitvorming iets makkelijker gaat dan wanneer ik denk dat er echt iets moet gebeuren, omdat ik denk dat die patiënt daar beter van wordt.

**I: Dus dat bedoelt u met, ik denk dat het in de palliatieve fase eigenlijk beter gaat of meer voorkomt.**

R: Ja. In ieder geval zijn de omstandigheden dan wat beter, want daar gaat het wel om wat die patiënt graag wil. Kijk, als je longkanker hebt en de beste behandeling is een kwab eruit halen, dan kan die patiënt wel zeggen, heb je niet wat anders, maar ja, sorry hoor, ik heb niks anders. Het is óf behandelen en doorpakken óf we leggen ons erbij neer dat het niks meer wordt. Daar is shared decision making, nou, dat zie ik niet goedkomen. Maar in de palliatieve fase kan ik de patiënt niet meer beter maken. “Wat worden dan eigenlijk de behandeldoelen? Waar gaan we nou aan werken samen? Waar gaat u aan werken?” Daar zou het wat makkelijker kunnen zijn.

**32.28**

**I: Ik zit even te denken hoor. Ja, ik wilde vragen, waarom gelooft u dan niet in gezamenlijke besluitvorming? Maar dat heeft u eigenlijk al verteld.**

R: Eigenlijk is het een oneerlijke situatie. Ik moet je zeggen dat als een patiënt al komt met ideeën over een bepaalde behandeling, er dan vaak zoveel crap bij zit van dingen die ze op het internet hebben gevonden. “Ik zou heel graag gemalen spinnenkoppenthee willen drinken tegen mijn astma, want ik heb op het internet gevonden dat je met gemalen spinnenkoppenthee astma kunt verhelpen.” Dan ben ik gauw klaar. Dan is het niet, jij doet jouw spinnenkoppenthee en ik doe mijn pufjes. Nee, als je er zo instaat, dan moet je maar een ander zoeken, want daar kan ik niks mee. Dat is een beetje gechargeerd hoor. Eigenlijk is therapietrouw of therapie-ontrouw al een vorm van shared decision making. Ik doe een voorstel, volgens mij moet je twee keer per dat dit pufje inhaleren, want dat zou voor jouw astma weleens een oplossing kunnen zijn. De manier waarop die patiënt omgaat met mijn advies is eigenlijk al een klein beetje gezamenlijke besluitvorming. Ik doe een aanbeveling en sommige patiënten nemen dat over, omdat ze merken dat het helpt of omdat ze denken dat ik gelijk heb of uit angst. En sommige mensen denken, die lange kan lullen wat hij wil, maar ik ga gemalen spinnenkoppenthee drinken en die pufjes geloof ik wel, want daar krijg ik bijwerkingen van en daar zitten hormonen in, ik denk er niet voldoende aan of ik vergeet het steeds. Eigenlijk is dat al een vorm van, ik neem ook een stukje verantwoordelijkheid in mijn behandeling.

**I: Of niet…**

R: Of niet. Maar dan in ieder geval je eigen keuzes maken. Ik denk dat het in die zin veel meer een soort verborgen vorm van shared decision making is, als dat ik zeg, u heeft astma, wat denkt u zelf dat we het beste kunnen doen? Dan zegt hij, doc., daar heb jij voor doorgeleerd, zeg het maar. “Nou, volgens mij moet je stoppen met roken.” Nou, daar hebben we er alweer een. Dat wil niet iedereen of dat kan niet iedereen. Dus dat is eigenlijk ook een vorm van gedeeld keuzes maken of gedeelde verantwoordelijkheid.

**35.00**

**I: Kunt u een voorbeeld geven van een situatie waarin er wel gezamenlijke besluitvorming plaatsvond? U gaf al aan, die therapietrouw vind ik een hele interessante en ook bijvoorbeeld het wel of niet roken. Dat u zegt, nee, u moet niet roken, maar dat iemand het wel doet. Dat is ook een zekere vorm van… Maar bijvoorbeeld in uw consulten met patiënten…**

R: Soms met pufjes. “Ik heb liever geen ontstekingsremmers, want dat zijn hormonen.” Oké. Dan doen we een lagere dosis of we kiezen een alternatief. Of, oké, maar dan heb je klachten. Ook goed. Ik heb een aanbeveling. Die wilt u niet opvolgen of die kunt u niet opvolgen om wat voor reden dan ook. Ja, dan moet je je erbij neerleggen dat je klachten houdt. Die afweging van het middel aan de ene kant en de kwaal aan de andere kant, daar kun je best met elkaar over praten. “U heeft twee inhalatoren geprobeerd.” Dat is een beetje technisch, bijna vluchten als het gaat om shared decision making. “Je hebt er twee gehad. Qua getallen maakt het allemaal niet zo heel veel uit. Welke heb je nou het liefste?” Patiënten vinden dat al verschrikkelijk moeilijk. Dan is het vaak meer het kleurtje dan het feitelijke effect van het middel, want dat is dan een hele lastige. “Nou, doe die rode dan maar.” Dat heet dan officieel shared decision making, maar dat stelt natuurlijk niks voor. Dat is geen shared decision making. Shared decision making is, ik heb een aantal ideeën over wat er nou zou moeten gebeuren, hier heb je vijf opties, wat zou je het liefste gaan doen? Nou, dat is vaak niet zo en bij longkanker, opereren, ja of nee, zijn die vijf opties er niet. Dan zijn er maar twee, opereren en beter worden of niet opereren en doodgaan. Zeg het maar. Ik weet niet of dat nou een faire vorm van shared decision making is. En aan de andere kant, de triviale keuze van, heb je liever een blauwe of rode puffer, nou, het geeft mij natuurlijk wel een heel warm gevoel als ik die patiënt erover laat meedenken, maar dat is geen shared decision making. Dus, oké, u bent drieëntachtig, u heeft lelijk COPD, u komt de deur niet meer uit, wat gaan we nou wel of niet nog doen? Dat is shared decision making. Met de beperkingen van de duur van het consult, met de beperkingen van de gezondheidsvaardigheden van de kant van de patiënt, met de beperkingen van de communicatieve vaardigheden en skills van de zorgverlener. Maar dan heb je een situatie waarin je kunt zeggen, oké, we kunnen een aantal dingen doen. Wat denk je zelf, wat zullen we wel doen en wat zullen we niet doen? Dat zou je shared decision making kunnen noemen. “Nou, ik kom niet meer naar het ziekenhuis.” Dat vind ik een hele mooie. Ik heb patiënten, die zeggen, ik kom niet meer naar het ziekenhuis. Maar dat is meer omdat de schoondochter geen auto meer heeft dan omdat ze denken, volgens mij kan die lange toch niks meer voor mij doen, ik ga daar niet meer naartoe. Sommige mensen bellen mij op en die zeggen, ik kan niet meer naar de poli komen. Dat is een vorm van shared decision making. Maar als dat een praktische reden heeft, is het natuurlijk iets anders dan dat die patiënt eens even goed bij zichzelf te rade is gegaan, ik heb nou twintig jaar COPD, ik kan echt helemaal niks meer. Iedere keer als ik bij die dokter kom praten we over pufjes, maar niet over, hoe gaat het nou eigenlijk met u, of over, hoe moet het nou straks? Dan denkt die patiënt, dan hoef ik daar niet meer naartoe. Dat is een mooie vorm van shared decision making, maar dan hebben we geen goed gesprek gevoerd. Dan is die patiënt gewoon op mij uitgekeken. Ja. Dat vind ik wel een vorm van shared decision making, maar ik zou dat liever vanuit een positieve grondhouding doen. “U kunt niks meer voor mij betekenen, dat is prima, dan zal ik u ook niet lastigvallen, maar u kunt mij nog wel even helpen met de thuiszorg of met een hoog-laagbed bestellen.” Noem het maar op allemaal. Of, zorg voor een fatsoenlijke overdracht naar de huisarts, in de zin van, ik heb deze patiënt een aantal jaren met COPD behandeld. Ik ben echt klaar, ik kan daar nou niks meer aan veranderen en toevoegen. Wil jij het overnemen om de laatste fase zo goed mogelijk te begeleiden?

**I: Gaan jullie ooit zo ver dat jullie ook echt zorg voor thuis gaan regelen en zo?**

R: Of in ieder geval zorgen dat een ander het doet.

**I: Of dat dat bij de huisarts ligt.**

R: Dat het bij de huisarts komt. Dingen thuis regelen is voor de huisarts makkelijker dan voor ons. Dus ja, dat doen we wel. Ook wel met een overdracht met wat adviezen en instructies, maar dan maken we er dus een einde behandeling van. Dan zeggen wij, wij hadden deze patiënt tot nu toe in de tweede lijn in behandeling, maar ik zou graag willen dat de huisarts nu in ‘the lead’ komt en hoofdbehandelaar wordt. Eigenlijk betekent dat dat wij onze behandeling afsluiten en dat we de patiënt overdragen aan de huisarts. Dat is jammer, want je zou het eigenlijk een beetje samen willen doen. Een hele enkele keer – en dat gebeurt misschien één keer per jaar – ga ik met een huisarts samen thuis kijken. De patiënt zegt, ik kan niet meer komen. De huisarts denkt, volgens mij is er toch nog wel wat te palliëren of te winnen. Dan spring ik op mijn fietsje en gaan we samen een huisbezoek doen. Dat wordt enorm gewaardeerd door de patiënt, door de familie, vaak ook door de huisarts, maar het kost me wel een uur. Het is toch een beetje lastig plannen. Het is vaak aan het eind van de dag. De laatste keer dat ik er geweest ben, zeek het van de regen, dus ik kwam daar werkelijk als een verzopen kat binnen. Dat belast het consult. Ik kon niet eens zitten. Ik kon nergens zitten. Ik was gewoon echt soaked. Dus het duurde even voordat wij dat gesprek konden voeren. Die huisarts zit daar dan te wachten en die denkt, ja, waarom komt die lul niet met de auto? Dus ja, het is allemaal niet zo heel erg makkelijk. Maar dat zou denk ik wel een hele goede zijn. “Tot nu toe heeft die longarts voor u gezorgd. We gaan nu even samen de boel bekijken. Dat doen we bij u thuis. Eigenlijk is dat ook een soort overdrachtsmoment.” Dat zou geweldig zijn. Het zou fijn zijn als daar een tariefje voor was, want dit uur wat ik daar dan nu aan besteed wordt niet vergoed. Overigens, het geeft mij een goed gevoel en ik heb het er graag voor over, maar als de kachel moet branden – en dat moet hij hier natuurlijk ook – kun je niet uren besteden aan dingen die eigenlijk niks opleveren. Dus het zou fijn zijn als dat een beetje…

**I: Maar bedoelt u dan hoe die overdracht met die huisarts gaat? Dat gebeurt al. U bedoelt echt het huisbezoek.**

R: Het huisbezoek. In die fase een huisbezoek. Volgens mij zou dat heel waardevol kunnen zijn.

**41.59**

**I: Ik had een volgende vraag, die gaat wat meer over de thema’s die in een gesprek met de patiënt aan bod komen. Ik heb hier een aantal thema’s op een rijtje gezet. Even kijken hoor. Levensbeschouwing en culturele achtergrond. Niet-behandelafspraken. Ziekenhuisopnames. De plaats van zorg en sterven. Crisissituaties. Wettelijke vertegenwoordiging. Wilsonbekwaamheid en levenseindebeslissingen. Kunt u misschien per thema aangeven in hoeverre die thema’s aan bod komen in uw gesprek met patiënten?**

R: Levensbeschouwing en culturele achtergrond, niks, niet. Niet-behandelafspraken, bijna altijd wel, maar dat zijn dan dingen als niet-reanimeren, niet-beademen, geen IC-opname, wel of niet niet-invasief beademen. Dus een aantal technische aspecten van de zorg. Dat gebeurt bijna altijd. Daar worden we ook wel een klein beetje in getraind, om dat in elk geval wel te bespreken. Ziekenhuisopnames ook wel. “Als u nou weer zo beroerd wordt, wilt u dan toch weer opnieuw in het ziekenhuis opgenomen worden?” Bijna alle patiënten zeggen “ja”, omdat ze bang zijn dat ze anders thuis liggen te creperen. En zelfs als je met de patiënt afspreekt, ik wil dat niet meer, volgens mij wil ik liever thuisblijven, dan gaat het toch nog vaak mis, óf omdat een familielid die patiënt alsnog instuurt óf omdat op dat moment de huisarts, de eigen huisarts, niet beschikbaar is, er een vervanger komt of het is op de huisartsenpost en die willen dan niet de verantwoordelijkheid nemen om dat eigenlijk al afgesproken beleid op dat moment uit te voeren. Dus wel of niet ziekenhuisopnames wordt wel besproken, ook wel echt met een afspraak daarover, maar dat gaat niet altijd goed. De plaats van zorg en sterven, ja, dat sluit een beetje aan bij de vorige, ziekenhuisopnames. Ik denk dat veel patiënten het liefste thuis zouden willen sterven, totdat ze gaan sterven, want dan worden ze onzeker of dan wordt de omgeving onzeker of de huisarts komt er niet meer uit. Dus er sterven nu meer mensen in het ziekenhuis dan je graag zou willen, zelfs als je daar eigenlijk afspraken over gemaakt had.

**I: Daar geldt eigenlijk hetzelfde een beetje als wat u net aangaf.**

R: Precies. Crisissituaties. “Wie moet u bellen als u Spaans benauwd bent of als het helemaal mis is?” Daar geven we steeds vaker kaartjes voor mee, met het telefoonnummer van de zorgverlener of van de contactpersoon. Dus dat doen we wel iets vaker. Meestal is het toch de huisarts, want je weet nooit helemaal zeker of het echt alleen maar die aandoening is. Je kunt niet door de telefoon inschatten, moet het ingestuurd worden of kan het ook thuis? Dus vaak is dat toch een kwestie van op de huisarts terugvallen. Wettelijke vertegenwoordiging in een situatie van acute verslechtering. Er staat meestal een contactpersoon in de status, een dochter, een zoon of een partner, dus dat staat geregistreerd in het dossier. Informatie is niet altijd actueel. Er ontbreken vaak 06-nummers. Een enkele keer is een contactpersoon gewoon overleden, maar die staat er dan nog wel. Maar dat is iets anders dan de wettelijke vertegenwoordiger. De wettelijke vertegenwoordiger is, we hebben zes familieleden en wie is nou eigenlijk degene die de beslissingen neemt, die ook thuis de beslissingen neemt, die ook de afhandeling verzorgt?

**I: Ja, dus dat hoeft niet per se de contactpersoon te zijn.**

R: Dat hoeft niet per se de contactpersoon te zijn. Wilsonbekwaamheid en levenseindebeslissingen, eigenlijk is dat het eerste deel van onze discussie geweest samen. Die levenseindebeslissingen en de gezamenlijkheid daarvan, daar ontbreekt het echt een beetje aan. Minder in de oncologie, want daar zal het dus echt wel gebeuren, omdat je daar toch wel een soort kantelmoment in de casus hebt van, nou hebben we echt niks meer. Bij chronische aandoeningen, die veel geleidelijker achteruitgaan, is dat minder duidelijk. Wilsonbekwaamheid vind ik een lastige. Wanneer zijn gezondheidsvaardigheden zo beperkt dat je eigenlijk over wilsonbekwaam zou moeten praten? Ik heb patiënten, die zijn zo ontzettend dom dat ze eigenlijk niet in staat zijn om hun eigen beslissingen te nemen over van alles en nog wat, over euthanasie, want dat is dan een hele concrete, maar ook over wel of niet naar het ziekenhuis komen of over therapietrouw, alle dingen die we met elkaar besproken hebben. Sommige mensen zijn zo onnozel dat ze eigenlijk wilsonbekwaam zijn. Maar dat is iets anders dan in wettelijke zin wilsonbekwaam. Ik zeg het niet goed, bij sommige mensen lukt het mij zo slecht om de patiënt mee te krijgen in waar we eigenlijk naartoe zouden willen… Dat kan dus ook mijn eigen tekortkoming zijn. Dan denk ik maar, die patiënt moet simpel zijn, want ik ben het niet. Maar eigenlijk is het gewoon, het lukt ons niet om een fatsoenlijk gesprek te voeren. Dat is dus deels mijn onvermogen, maar waarschijnlijk ook wel voor een deel – tenminste, dat hoop ik dan maar – is het dat de patiënt gewoon te simpel is. Ja?

**I: Ja. Het laatste thema gaat over beperkte gezondheidsvaardigheden. Heeft u weleens gehoord van die term?**

R: Ja. Ik ben … (functie binnen alliantie) van de LAN, de Long Alliantie Nederland. We hebben vorig jaar een presentatie gehad van iemand van Pharos. Een fantastische presentatie. Die heb ik opgevraagd, staat nog op mijn computer. Die heb ik nog eens een keer teruggekeken. Over de aantallen, over de omvang van het probleem, maar ook over hoe je het zou moeten tackelen, wat je ermee zou moeten. Ja, ik heb daarvan gehoord.

**48.31**

**I: Hoe vaak heeft u zelf te maken met patiënten met beperkte gezondheidsvaardigheden?**

R: Dagelijks.

**I: Dagelijks.**

R: Ja.

**I: Vaak.**

R: Vaak. Als je het doortrekt naar niet in staat zijn om aanbevelingen op te volgen – dat is iets anders dan niet genegen zijn om aanbevelingen op te volgen – dan is het bij meer dan de helft van de patiënten het geval. Ik vind niet kunnen stoppen met roken… Roken is in mijn vak zo belangrijk dat niet kunnen stoppen met roken of dat niet willen bij mij bijna per definitie een beperkte gezondheidsvaardigheid is. Nou, kom er maar eens bijzitten. Dat heb je gedaan een middag. Roken is natuurlijk een dramatisch probleem in mijn praktische werk. Ik vind niet kunnen stoppen met roken of niet stoppen met roken bijna per definitie een beperkte gezondheidsvaardigheid. Maar ook niet over je ziekte kunnen praten of niet over jezelf kunnen praten of niet over je toekomst kunnen praten. Nou, dat ligt dus voor een deel aan mij, aan een gebrek aan tijd en een gebrek aan skills, maar het ligt ook wel gewoon bij de patiënt. “Ik kan dat niet, durf dat niet, wil dat niet.” Dat zijn ook beperkte gezondheidsvaardigheden. In die zin zit er bij iedere patiënt een grens aan de gezondheidsvaardigheden. Niet één is er perfect als het gaat om gezondheidsvaardigheden. Dan heb ik dus per definitie alleen maar patiënten met beperkte gezondheidsvaardigheden. Nou, natuurlijk heb ik ook patiënten die geweldig hun best doen om goed voor zichzelf te zorgen en om de aanbevelingen te volgen, maar dat is de minderheid.

**I: Mijn volgende vraag zou zijn is, hoe herkent u patiënten met beperkte gezondheidsvaardigheden? U geeft al aan, hoe op die stoppen-met-rokenvraag wordt gereageerd.**

R: De manier waarop ze de kamer binnenlopen. Dat kun je uitstralen. Misschien wel de oogopslag, misschien wel de manier waarop ze gaan zitten in de spreekkamer. Als je in de spreekkamer komt zitten met een eagere blik in je ogen in de zin van, wij gaan dit consult eens even optimaal benutten… Dat is natuurlijk al heel anders dan wanneer je de kamer binnen schuifelt, achterover ploft en uitstraalt, doc., zeg het maar. Dat gaat al betrekkelijk snel.

**I: Dus veel is uitstraling.**

R: Ja. Ja, heel wat. Mijn eerste vraag is, hoe gaat het met u? Nou, als het eerste antwoord “goed” is, terwijl ik zie dat het niet deugt, dan hebben wij al een beperkte gezondheidsvaardigheid. Maar er zijn ook mensen die zeggen, het gaat helemaal niet goed. Dan vraag je even door en dan is het niet veel anders dan de vorige keer en is het eerder een depressie dan een longprobleem of hebben ze ruzie met hun partner, weet ik veel. Dus volgens mij kom je daar betrekkelijk snel achter. Hoe langer je met elkaar praat, hoe vaker dat bevestigd wordt.

**I: Dus als je mensen beter leert kennen, dan…**

R: Dan weet je ook… Ik heb één patiënt gehad, die is nu overleden, die bijna elke week kwam, iedere week met hetzelfde verhaal. Ik had daar iedere week hetzelfde antwoord op, ik bereikte daar helemaal niks mee. De patiënt ging tevreden weg, want die had even met mij gepraat en na een week was hij helemaal weer gewoon met datzelfde verhaal terug bij af. Dan denk ik maar eventjes, dat ligt aan de patiënt. De eerlijkheid gebiedt te zeggen dat dat natuurlijk ook wel aan mij kan liggen, maar als je helemaal niet oppikt wat ik zeg of wat ik uitleg of wat ik probeer te bereiken, dan mag je denk ik wel spreken van beperkte gezondheidsvaardigheden of van ernstige beperkingen bij de zorgverlener. Dat kan ook.

**52.34**

**I: In het algemeen, ervaart u barrières in de zorg aan patiënten met dit soort vaardigheden?**

R: Ja, natuurlijk. Alleen al het feit dat ik niet bereik wat ik zou willen bereiken. Ik bedoel, ik lijd daar zelf ook onder. Je krijgt er toch een beetje een fijn gevoel van als het lukt wat je bij een patiënt probeert te bereiken. Als het niet lukt heb ik daar ook last van. Dus ja, dat ervaar ik. Bij veel mensen denk ik, als je nou zou kunnen doen wat ik denk dat goed voor je is, dan zouden we samen een heel eind verder komen. Dan hebben we het over dat stoppen met roken gehad of over therapietrouw, medicamenten goed gebruiken of snappen wat ik zeg, waardoor je een beetje vertrouwen krijgt of een beetje angst wegneemt of een beetje zelfredzaamheid creëert. Nou, dat gebeurt natuurlijk heel vaak niet. Nou, ik zou niet zeggen dat je dan trekt aan een dood paard, want dat klinkt een beetje onaardig, maar het gebeurt natuurlijk nogal eens dat we niet bereiken wat we zouden willen bereiken.

**I: Wat zou u nodig hebben om die zorg dan beter te kunnen laten aansluiten op dat soort patiënten?**

R: Ja, ik doe aan externe attributie, dus ik leg het even niet bij mezelf, die oplossing. Meer tijd zou helpen. Een familielid dat even meekomt zou echt helpen. Of een tool om een patiënt zich beter te laten voorbereiden op het consult. Dat heb ik daarstraks al even genoemd.

**I: Dat gaf u ook aan, ja.**

R: Maar het gebeurt niet, dat die patiënt denkt, ik ga morgen naar die dokter toe en volgens mij moet ik het met de dokter gaan hebben over dat ik de trap niet meer op kom of dat ik zo graag nog eens op vakantie zou willen of dat ik eigenlijk Spaans benauwd ben en dat ik bang ben dat dat alleen nog maar erger wordt. Dus de manier waarop een patiënt zich voorbereidt op het consult kan enorm van invloed zijn op de opbrengst van dat consult. Als een patiënt helemaal niet nagedacht heeft over, wie ben ik nou eigenlijk, waar ben ik nou eigenlijk, wat wil ik nou eigenlijk, dan is het consult veel te kort om daar echt iets met elkaar in te bereiken.

**I: Wat u bedoelt is dat u dan de eerste helft van het consult al bezig komt om op die plek te komen en dan is de tijd om.**

R: Ja, om de hulpvraag helder te krijgen. Als die al komt, want meestal is het niet een hulpvraag, maar een zorgaanbod en dat is niet per se hetzelfde. We hebben wel nagedacht over, stuur die patiënt een week van tevoren een A4’tje met een paar open vragen en een paar aankruisvragen. Nou, twintig procent van de mensen is functioneel analfabeet. Die zien een paar lettertjes staan, maar komen daar niet uit. Die schamen zich ervoor om daar een ander bij te betrekken. “Kijk eens even wat hier staat, want ik begrijp het niet.” Nou, dat is al een lastige. Maar bij sommige mensen ontbreekt ook gewoon het vermogen om echt introspectief te zijn. “Waar ben ik eigenlijk bang voor, wat zou ik over een jaar…” Schrijf in zo’n vragenlijst maar eens, bent u er over een jaar nog? Dat is een prachtige concrete vraag. Nou, ik zie het niet goed komen. Maar als ik jou zou vragen, ben je er over een jaar nog, dan zeg je, normaal gesproken natuurlijk wel, want ik mankeer niks en ik rijd redelijk rustig en ik rook niet en ik drink niet en ik doe geen gekke dingen. Dus jij kunt wel zeggen, volgens mij ben ik er over een jaar nog. Maar een vijfentachtigjarige met COPD… “Ja, dat weet ik eigenlijk niet, zeg het maar, doc.” Je hoeft mij niet te vragen of jij er over een jaar nog bent, maar zo’n patiënt zal zeggen, ja doc., dat weet ik niet, jij hebt ervoor doorgeleerd, zeg het maar. Maar eigenlijk zou je willen dat hij denkt, volgens mij is het echt binnenkort afgelopen. Er zijn mensen, die zie je voor je en dan denk je, nou, dat is over drie maanden wel gedaan. Maar dat ga je die patiënten niet zeggen. Dat zou je eigenlijk wel willen zeggen, maar op het moment dat ik zeg, volgens mij bent u over drie maanden dood, hebben wij wel even nodig om dat allemaal netjes te plooien.

**I: Dan ben je met acht minuten niet klaar, ben ik bang.**

R: In acht minuten wil dat natuurlijk niet. Ik kan vlot communiceren. Het is niet zo dat het mijn eigen onhandigheid is. Ik kan betrekkelijk snel to the point komen, maar zo’n patiënt heeft toch even nodig. “Wat zegt hij nou tegen me? Zegt hij nog drie maanden? Zou hij echt denken dat ik over drie maanden dood ben?” Je hoort het kraken. Sommige mensen horen het niet eens. Die denken, laten we maar gauw wegwezen hier, want dat wordt toch niets meer. Terwijl je eigenlijk moet zeggen, hoe gaat het dan die laatste drie maanden? “Nou, geleidelijk aan achteruit en u komt de trap niet meer op en u kunt niet meer zelf eten en drinken en u wordt Spaans benauwd en dan geven we een beetje morfine en dan gaat u dood.” Je kunt het betrekkelijk snel uitleggen. Ik kan in drie zinnen vertellen hoe het gaat. Maar wil je daar een gezellig gesprek over voeren, dan is het natuurlijk niet een kwestie van drie zinnen en dertig seconden.

**I: Nee. Nee.**

**[57.50– einde opname]**

Z1long04

I: interviewer

R: respondent

**I: Oké. Ik heb er in ieder geval geen last van hoor. Had jij nog vragen vooraf?**

R: Nee hoor.

**I: Oké. Ik zeg steeds “je”. Is dat goed?**

R: Ja.

**I: Wat is jouw rol in de zorg voor patiënten in de palliatieve fase? Ik begin heel breed.**

R: Misschien moet ik toelichten dat ik enerzijds gewoon als algemeen longarts hier werk en dan heb ik een aandachtsgebied. Dat is thuisbeademing. In die beide gebieden heb ik er wel mee te maken. Dus in de thuisbeademing hebben we soms situaties waarbij patiënten in een palliatieve levensfase zitten. Dan geef je dus bijvoorbeeld adviezen over medische zorg et cetera. Als algemeen longarts heb ik dus ook af en toe patiënten op mijn spreekuur die door hun longaandoening ook in de palliatieve fase zitten. Dus dat is er zo nu en dan. Ja. Ja.

**01.11**

**I: Even kijken. Bij patiënten die jullie hebben in de palliatieve fase, is dan altijd één centrale zorgverlener aanwezig?**

R: Ik denk dat dat wel over het algemeen het streven is. Ik denk dat dat in de praktijk niet altijd zo is. Wat bijvoorbeeld bij patiënten van de thuisbeademing… Dat is meteen een beetje een ingewikkelde categorie in de zin van, patiënten krijgen van ons een beademingsapparaat en in feite leveren wij eigenlijk alleen maar de zorg rondom die beademing. Maar in de praktijk komt het er vaak op neer, doordat wij daardoor zo’n nauw contact hebben met de patiënt, dat de patiënt ons ook gaat zien als een soort centraal punt om dingen aan te vragen, terwijl daar eigenlijk de huisarts de leidraad zou moeten nemen of de neuroloog, als de patiënt een neurologische aandoening heeft. In de praktijk denk ik dat er wel naar wordt gestreefd, maar dat dat niet altijd zo is. Ja.

**I: Is het duidelijk voor de patiënt wie dat dan is altijd? Niet dus, als ik jou zo hoor.**

R: Nee, ik denk dat we dat bij de thuisbeademing wel vaak proberen te communiceren naar patiënten, zo van, wij doen nadrukkelijk die beademings-gerelateerde zorg en voor andere zaken… Of, uw hoofdbehandelaar verder is iemand anders. Dan ligt het er ook maar net aan hoe betrokken andere zorgverleners zijn hè? We hebben bijvoorbeeld patiënten met spierziekten als de ziekte van Duchenne of zo. Die hebben vaak helemaal geen neuroloog meer, omdat die vaak niet veel kan toevoegen. Als die een goede huisarts hebben, is de huisarts vaak de spil, maar soms is dat niet zo en dan neem je die rol toch weer over. Ja.

**I: Dus als ik jou goed begrijp en anders moet je me verbeteren, je hebt dus die thuisbeademingszorg, maar ook hier in het ziekenhuis. Kun jij over die rol nog iets meer vertellen, want die begreep ik nog niet helemaal.**

R: Ja goed, in het ziekenhuis heb ik natuurlijk soms patiënten met andere longaandoeningen, bijvoorbeeld een eindstadium COPD of toch een verdwaald iemand met longkanker die niet bij een oncologische longarts zit. Ja goh, ik denk eigenlijk stiekem, nu ik er zo over nadenk, dat het toch wel vaak zo is dat meerdere zorgverleners betrokken zijn met alle goede bedoelingen en dat het dan toch niet altijd heel duidelijk is, ook bij die algemene longziekten, wie dan eigenlijk de centrale figuur is die dat coördineert. Ja.

**I: Even over jouw rol in het ziekenhuis, hoe werk jij dan samen met andere zorgverleners binnen het ziekenhuis?**

R: Ja. Ja, dat is natuurlijk erg afhankelijk van de situatie hè? Bij longpatiënten in een palliatieve fase, soms kiezen wij ervoor om dat stukje zelf te coördineren, omdat wij ook wel wat kennis van en ervaring hebben met palliatieve zorg. Dan zijn wij degenen die dan af en toe bijvoorbeeld heel specifiek de hulp inroepen van een andere zorgverlener. En soms kiezen we er heel nadrukkelijk voor om het palliatieve team te betrekken. Eigenlijk doen we dat steeds meer. Het palliatieve team dat we hier hebben.

**I: Is dat ook hetzelfde als dat expertiseteam?**

R: Ja. Ja. Ja, precies. Ja. Ja, daar zitten natuurlijk weer verschillende disciplines in. Dus als je dat palliatieve team dan betrekt, dat is eigenlijk wel heel handig, want dan sturen zij dat ook mee aan. Ja. Maar als dat niet zo is, dan vragen wij anderen in consult bijvoorbeeld hè? Misschien hebben we een keer een geestelijk verzorgende nodig die ondersteuning moet bieden. Dan vragen wij dat, of zij betrokken zijn bijvoorbeeld. Ja.

**04.46**

**I: Hoe werken jullie samen met de huisarts? Wat je al aangaf voor de thuissituatie.**

R: Ja. Ja. Over het algemeen, als patiënten dusdanig serieuze gezondheidsproblemen hebben, dan is het streven wel om de huisarts altijd telefonisch en schriftelijk op de hoogte te stellen. Dat is in ieder geval mijn persoonlijke streven. Dus bijvoorbeeld een patiënt die in het ziekenhuis heeft gelegen en in een palliatieve fase zit, als die naar huis gaat, bellen we vaak wel de huisarts op en dan geven we vaak ook aan van, goh, wij sluiten hem af of wij spreken geen reguliere controles meer af en we hebben tegen de patiënt gezegd van, de huisarts is het eerste aanspreekpunt, neem alsjeblieft contact op als jullie toch van ons iets nodig hebben. Ja. En soms loopt dat een klein beetje parallel. Soms heb je mensen die in een palliatieve fase zitten en toch nog voor controles komen, waarbij de huisarts dan ook betrokken is. Idealiter communiceer je dan goed onderling.

**I: Dus dat is niet altijd zo het geval?**

R: Nou ja, goed. Dat is natuurlijk een beetje ook afhankelijk van de situatie, met welke personen je samenwerkt. Ja, dat is bij huisartsen ook heel wisselend. Niet “heel wisselend”, maar je hebt natuurlijk huisartsen die zich heel betrokken opstellen in bepaalde casussen en sommigen bij wie dat iets minder is. Ja, ik vind het vooral toch altijd heel bijdragend om gewoon even te bellen met een huisarts, zodat je elkaar echt eventjes kunt spreken. We kunnen wel een formele brief sturen, dat doen we natuurlijk ook altijd, maar ja, zo’n brief belandt ook ergens misschien op een grote stapel. Dus als ik het belangrijk vind dat de huisarts daar een grote rol in gaat spelen, dan bel ik eigenlijk altijd wel.

**I: Toch even een belletje.**

R: Ja.

**I: Je noemde even andere partijen. Ik weet niet meer wat je zei. Maatschappelijk werk, zei je dacht ik?**

R: De geestelijk verzorger zei ik.

**I: Oh ja, de geestelijk verzorger. Sorry. Maar zijn er behalve de zorgverleners ook dat soort partijen waar jullie dus gebruik van maken?**

R: Ja, weleens. Ik moet ook zeggen, dat is dan meer in de klinische setting en de verpleegkundigen van de afdelingen doen dat eigenlijk vaker dan wij. Die zien weleens dat patiënten bijvoorbeeld toch misschien enerzijds verlegen zitten om een praatje of echt met existentiële vraagstukken zitten en dan zeggen zij vaak tegen ons, vraag die geestelijk verzorger er eens bij, die hebben daar tijd voor, die hebben daar ook natuurlijk meer kennis en ervaring mee. Ja.

**07.14**

**I: Binnen de palliatieve zorg is aandacht van belang voor meerdere aspecten. Het fysieke, psychische, sociale en spirituele, wat je ook al aangaf. In hoeverre komen deze aspecten aan bod in jouw gesprekken met de patiënt?**

R: Nou, het spirituele eigenlijk niet. Het is niet dat ik dat niet zou willen, maar ik denk dat ik daar ook ergens een beetje misschien wel de levenservaring mis om daar… Ik zou niet goed weten hoe ik daar met mensen over zou moeten praten, laat ik het zo zeggen. Ik denk, het fysieke komt eigenlijk altijd wel aan bod. Dat is het makkelijkste hè? Het psychische ook wel. Ik probeer toch vaak mensen te vragen in hoeverre ze ermee zitten, of ze zich somber voelen. Dat soort dingen vraag ik wel na. Wat was die andere?

**I: Het sociale.**

R: Ja, sociaal ook wel hè?

**I: De omgeving.**

R: Dat je kijkt hoe de omgeving ermee omgaat en of er zorgproblemen zijn. Maar ik denk dat stukje… Je kent vast Sander de Hosson. Ken je die? Een longarts die op dit gebied ook heeft geschreven over palliatieve zorg. Die is heel vaak in de media.

**I: Ja, ik denk het wel. Ja.**

R: Die schreef ook dat artsen daar eigenlijk ook meer aandacht voor zouden moeten hebben. Dan denk ik van, ik ben het wel met hem eens, maar ik zou persoonlijk niet weten hoe ik dat zou moeten doen.

**I: Dan bedoel je het spirituele.**

R: Precies. Ik snap dat mensen met een soort van existentiële vragen zitten over het waarom en hoe. Daar voel ik mij niet bekwaam toe, om daarover te beginnen, want ik weet eigenlijk niet hoe ik dat aan zou moeten pakken. Ja. En het tijdsaspect speelt dan misschien ook wel een rol, want het is niet iets wat je even tussen neus en lippen door bespreekt, van, bent u bang om dood te gaan?

**I: Ja, dat is niet gewoon een afvinkvraagje inderdaad.**

R: Nee, precies.

**I: Jullie hebben denk ik tien minuten of zo? Wat is een reguliere…**

R: Ja, voor een controlepatiënt tien minuten. Voor thuisbeademing hebben we wel twintig minuten. Dat is iets meer.

**I: Ja, ietsjes meer. En die aspecten, je geeft aan die laatste minder, maar bijvoorbeeld het psychische en sociale, breng je die dan zelf ter sprake?**

R: Ja, ik denk dat ik dat over het algemeen… Ja goed, je probeert natuurlijk ook wel een beetje te polsen of in te schatten of het relevant is, maar zeker het sociale denk ik dat ik altijd wel even… Nou ja, hoewel, misschien niet altijd. Ja.

**I: Ja, zo vaak mogelijk even polsen in ieder geval.**

R: Ja. Ja.

**I: Doe je dat bij alle patiënten zo?**

R: Ik denk dat dat ook een beetje afhankelijk is van de ernst van de ziekte en het stadium van de ziekte. Maar als ze dus een ziekte hebben die al in een dusdanig ver stadium zit, waarbij mensen ook heel erg beperkt zijn, toevallig is het zo dat we bij de thuisbeademing bij elke nieuwe patiënt, elke nieuw verwezen patiënt, en soms ook verderop, vragenlijsten afnemen voor het consult.

**I: Vooraf.**

R: Ja. Dat zijn ook vragenlijsten over angst en depressie. Dat zijn vragenlijsten over de mantelzorg, over de zorgbelasting. Dus daar komt dat eigenlijk altijd wel aan bod heel nadrukkelijk. Bij de thuisbeademingspatiënten veel meer dan bij de andere patiënten, ook omdat dat meer gevolgen heeft ook voor onze interventie met thuisbeademing hè? Dat kan alleen maar in een bepaalde thuisomgeving. Dus daar hebben we daar wel heel nadrukkelijk aandacht voor. Ja.

**10.30**

**I: Dus dan leg je dat op die manier vast eigenlijk hè?**

R: Ja.

**I: Dat is dus in die thuissituatie, maar hoe gebeurt dat in die andere situatie, bijvoorbeeld als jij een consult voert of zo?**

R: Ja… Als ik het bijvoorbeeld heb over een eindstadium COPD, dat is natuurlijk ook wel iets. Ja, ik probeer toch vaak wel één vraag te stellen, bijvoorbeeld dat ik weleens zeg, goh, bent u nou heel somber omdat u zo weinig kan? Weet ik wat. Dat probeer ik wel in één zin te zeggen. En over dat sociale… Ja… Ik denk dat het streven van mij is om dat ter sprake te brengen, maar het gebeurt niet altijd.

**I: Maar die dingen, die leg je dan vervolgens vast in een zorgplan?**

R: Oh zo. Nee, ik maak daar gewoon een aantekening van, een notitie. Standaard als ik een nieuwe patiënt zie doe ik altijd een kopje “sociaal”. Dat kan zijn van, getrouwd, twee kinderen, werkt als dit en dat. Maar bij een ALS-patiënt bijvoorbeeld, die bij de thuisbeademing komt, schrijf ik wel nadrukkelijk op van, woont gelijkvloers, krijgt mantelzorg van echtgenote, thuiszorg wordt opgestart. Dat is natuurlijk ook wel iets heel praktisch hè, maar dat noteren we dan wel heel nadrukkelijk. Ja.

**I: De mate waarin je die aspecten ter sprake brengt, zitten daar ook verschillen in bij patiënten die bijvoorbeeld heel laag zijn opgeleid of die niet veel lijken te snappen?**

R: Ja, dan kom je waarschijnlijk wel een beetje uit bij het tijdsaspect, dat mensen bij wie je dan al moeite hebt misschien om het stukje medisch toe te lichten of uit te vragen… Als jou dat natuurlijk al vrij veel moeite kost, dan kan ik me inderdaad voorstellen dat je dat andere dan maar achterwege laat, omdat je daar gewoon niet meer aan toekomt. Ja. Maar niet per se omdat ze dat minder goed begrijpen of zo, maar meer dat waarschijnlijk het hele consult dan al meer tijd in beslag neemt en dat je dan misschien gaat kiezen wat je gaat bespreken, wat je dan het belangrijkste vindt.

**I: Ja, want je hebt immers ook maar een beperkte tijd natuurlijk.**

R: Ja. Ja.

**I: Even een volgend thema. Een goede communicatie is de basis voor de zorg, zodat de patiënt goed geïnformeerd is en er sprake kan zijn van gezamenlijke besluitvorming. Zijn er patiënten bij wie jij de communicatie moeilijker vindt?**

R: Ja, kijk, het is natuurlijk altijd je doel om bepaalde informatie over te brengen en soms heb je toch het idee dat die informatie niet goed overkomt. Dan weet je soms niet meer zo goed hoe je dat dan anders moet zeggen of formuleren om toch soms ingewikkelde informatie bij een patiënt begrijpelijk over te brengen. Dus ik denk dat je soms wel merkt dat je toch relatief moeilijke materie moet bespreken en dat het soms bijna niet te doen is om dat te vertalen naar een soort van leek, op een nog simpelere manier, dat is soms inderdaad wel lastig. Ik heb daar niet zo heel veel mee te maken, maar mensen uit een hele andere cultuur die dan ook nog de taal bijvoorbeeld minder goed machtig zijn, ja, dan heb ik wel soms het idee dat er wel wordt geknikt en “ja en amen” wordt gezegd, maar dat ze het waarschijnlijk niet echt kunnen reproduceren. Maar dat hebben we eigenlijk in … (naam stad) relatief weinig.

**I: Dat valt mee.**

R: Ja. Minder culturele diversiteit, zal ik maar zeggen. Ja.

**14.12**

**I: Wat doe jij dan om de communicatie te verbeteren?**

R: Ja, dan toch dingen zo simpel mogelijk uitleggen of er soms voor kiezen om dan bepaalde informatie weg te laten, denk ik, om het simpeler te maken. En soms denk ik ook, je hoeft ook niet altijd alle details, alle medische details met de patiënt te bespreken. Dat is natuurlijk wel een beetje de maatschappelijke tendens dat zeker ook jonge, misschien hoger opgeleide patiënten alles willen weten en wij als jonge dokters ook denken dat we de patiënt echt van elk detail bewust moeten maken, maar soms denk ik dat je ook weloverwogen kunt zeggen van, nou, ik versimpel het een beetje, dus ik vertel niet elk detail van wat ik op een scan zie, maar ik vat het een beetje samen, zodat het nog begrijpelijk is.

**I: Zou je daar een voorbeeld van kunnen geven misschien, uit de praktijk?**

R: Ja kijk, soms heb je mensen die hebben longkanker en die zitten in een traject waarin ze bijvoorbeeld geen behandeling meer krijgen, maar wel een scan ter controle. Stel, er zijn nieuwe afwijkingen te zien, waarbij je eigenlijk wel twijfelt of dat iets met die kanker te maken heeft, dan kun je in feite twee dingen doen. Je kunt zeggen, nou, die scan ziet er eigenlijk nog in orde uit, maar we controleren het voor de zekerheid over een paar maanden nog een keer. Of je kunt zeggen, ja, ik zie een paar afwijkingen, dat kan niks zijn, kan ook wel iets zijn, we maken een scan over drie maanden. Waar is de patiënt dan het meest blij mee? Dat is heel erg per patiënt verschillend. Dat voel je op een gegeven moment ook wel aan. Als je een iets vagere omschrijving geeft en de patiënt vindt dat prima en is er tevreden mee… Of je hebt dan een patiënt die gaat doorvragen. Dat is een voorbeeld waarbij je zegt van, nou, ik belast de patiënt niet met iets wat mogelijk in de toekomst een probleem gaat zijn, maar wat nu eigenlijk nog geen probleem is, wat moeilijk uit te leggen is, dus ik zeg, het is oké.

**I: Dat gebeurt eigenlijk heel erg intuïtief…**

R: Ja. Ja. Ja. Dat heeft dan ook wel te maken met of je kan inschatten of een patiënt dat kan begrijpen of dat hij daar misschien volledig van in paniek raakt. Maar dat is natuurlijk een beetje lastig, omdat tegenwoordig de tendens inderdaad is shared decision making. Iedereen moet alles…

**I: Ja…**

R: Maar eigenlijk is dat voor patiënten vaak helemaal niet zo fijn. Maar dat moet je wel goed aanvoelen natuurlijk, want de patiënt heeft er natuurlijk wel recht op om alles te weten. Sommige mensen willen wel elk detail weten en dat moeten ze dat ook krijgen.

**I: Hoe voel jij dat dan aan? Is dat op basis van hoe het gesprek gaat?**

R: Ja, ik denk dat je van tevoren al een bepaalde inschatting gemaakt hebt, toch een beetje op basis van intelligentie. Ik denk ook dat je tijdens een gesprek wel een beetje merkt wat patiënten nog kunnen begrijpen en wat niet. Als je dan al vrij snel merkt dat de informatie te ingewikkeld is of mensen dat niet meer goed kunnen onderscheiden, dan kan je de keuze maken om het iets anders in te kleden. Ik ga geen onwaarheden vertellen. Ik ga niet zeggen als er iets mis is dat er niks mis is, maar je kunt het natuurlijk wel op veel verschillende manieren zeggen. Ik denk dat het weglaten van bepaalde details die de patiënt toch niet kan begrijpen, dan een manier kan zijn.

**17.46**

**I: Behalve die manier, zijn er dan nog andere… Ik noem het nu even een trucje, maar dan doe ik het tekort. Maar zijn er nog andere dingen die je gebruikt?**

R: Goh, soms als ik voorzie dat het allemaal ingewikkeld is, dan laat ik weleens dubbele tijd inplannen, maar dat is eigenlijk maar heel zelden het geval. Ik denk dat dat uit een soort van financieel oogpunt ook niet heel wenselijk is, dat wij allerlei dubbele tijd gaan inplannen voor patiënten. Maar dat doe ik ook weleens een enkele keer, als ik weet van, ik moet allerlei moeilijke dingen bespreken, dat ik daar extra tijd voor inplan. Of mensen vragen om iemand mee te nemen. Dat kan ook wel helpen. Of als ik echt merk van, iemand is alleen en die snapt er helemaal niks van, dat is natuurlijk niet zo heel vaak zo, maar dat je dan weleens vraagt van, goh, zal ik u dochter ook nog even bellen om het te vertellen? Of op de afdeling is dat ook weleens, als mensen erg ziek zijn en het moeilijk begrijpen, dat ik ook weleens voorstel om de contactpersoon ook even zelf in te lichten. Mensen vinden dat ook vaak goed. Ja.

**I: In gesprekken, check jij dan ook weleens ooit of patiënten jou hebben begrepen?**

R: Ja, maar misschien niet zo heel nadrukkelijk. Wat ik wel soms doe is een keer daarna, bij een gesprek wat erop volgt, vragen van, goh, wat hebben we de vorige keer besproken, wat hebt u daarvan onthouden?

**I: Ah zo, dat je even weet waar je staat.**

R: Ja, dat je probeert toch de mensen te ontlokken hoe zij dat nou geïnterpreteerd hebben, want soms is dat toch wel anders dan dat je gedacht had. Ja, ik vraag wel heel vaak van, is het duidelijk voor u? Dat soort dingen. Maar ja, het is altijd een beetje de vraag of dat het helemaal dekt, maar dat vraag ik wel regelmatig. Ja. En ik vraag ook eigenlijk altijd standaard voordat mensen naar buiten lopen of ze nog vragen hebben of opmerkingen, dat soort dingen.

**I: In hoeverre is er volgens jou sprake van gezamenlijke besluitvorming met patiënten in de palliatieve fase?**

R: Ja, ik denk dat dat er wel heel nadrukkelijk is, maar je merkt gewoon toch een heel groot verschil. Je hebt gewoon verschillende typen patiënten. Ik denk over het algemeen dat je wel kunt stellen dat… Ja, ik noem het even… Welke term gebruiken jullie ook alweer?

**I: Gezondheidsvaardigheden.**

R: Ja, ik denk dat hoe meer gezondheidsvaardigheden patiënten hebben – en dat hangt natuurlijk toch wel vaak samen met opleidingsniveau en ook een soort van ervaring met bepaalde dingen – dat beter gaat dan wanneer patiënten hele beperkte gezondheidsvaardigheden hebben, omdat mensen het vaak gewoon niet kunnen overzien. Je kunt wel allerlei dingen samen willen beslissen, maar om een voorbeeld te noemen, we willen vaak ook met patiënten bespreken of ze nog gereanimeerd willen worden als ze een hartstilstand krijgen, of ze bijvoorbeeld nog op een Intensive Care opgenomen willen worden, maar mensen kunnen zich er helemaal geen voorstelling van maken hoe dat is. Zelfs voor ons als zorgverleners is dat soms moeilijk. Ja, idealiter zou je dat samen met een patiënt willen bespreken en dan samen daar een besluit over nemen, maar soms merk je toch dat je toch wel moet sturen, denk ik, omdat mensen gewoon niet kunnen overzien wat dat dan inhoudt, omdat dat te abstract is en mensen daar gewoon geen kijk op hebben. Maar er zijn natuurlijk patiënten die wel heel goed weten wat ze wel en niet willen, er heel goed over nadenken en goed geïnformeerd zijn. Maar ja, dat gaat wel makkelijker over het algemeen, vind ik, als patiënten dus wat meer zorgvaardigheden hebben en misschien wat intelligenter zijn. Grosso modo (ongeveer) hè? Er zijn natuurlijk altijd verschillen.

**21.34**

**I: De volgende vraag zou zijn, is dit voor alle patiënten hetzelfde? Nou, nee dus, als ik jou goed begrijp ☺.**

R: Nee, dat is wel echt heel duidelijk. En het is ook nog wel zo dat er ook wel heel erg duidelijk patiëntvoorkeuren naar voren komen. Je merkt toch bij een vrij grote groep van patiënten dat als je bijvoorbeeld zelf zegt van, er zijn meerdere mogelijkheden, we kunnen A, B of C kiezen, ze toch best wel vaak zeggen, dokter, zegt u maar wat het beste is. De publieke opinie is een beetje van, dat beslist de patiënt, wat het beste is. Toch hoor je nog heel vaak van patiënten terug, u hebt ervoor gestudeerd, zegt u het maar, wat zou u doen? Ja.

**I: Dus dat zou dan een situatie of een voorbeeld kunnen zijn waarin dat juist niet gebeurde.**

R: Ja.

**I: Kun je ook misschien een voorbeeld benoemen waarbij er heel duidelijk sprake was van gezamenlijke besluitvorming?**

R: Nou, wat een duidelijk voorbeeld is, dan haal ik weer even die thuisbeademing erbij. We hebben veel mensen met neurologische aandoeningen, bijvoorbeeld ALS. Dat is een progressieve spierziekte, waar je over het algemeen binnen vrij korte tijd aan overlijdt. Je overlijdt dan aan je longprobleem, doordat je longspieren niet meer werken. Dat kunnen we dan tijdelijk opvangen met een kapbeademing, met een kapje dat je heel makkelijk op en af kan zetten, wat mensen ook thuis kunnen gebruiken. Maar op een gegeven moment is dat niet meer voldoende en dan kun je nog kiezen voor invasieve beademing. Dat is dus via zo’n opening in je luchtpijp, waaraan dan echt continu een beademingsapparaat wordt aangesloten. Dan word je dus echt in leven gehouden door een machine. En dat moeten wij dus met die patiëntengroep bespreken, of ze dat wel of niet willen. Dat is echt een gezamenlijke beslissing. Wij hebben er wel een advies over. Ons advies is over het algemeen namelijk om dat niet te doen en daar hebben we ook bepaalde argumenten voor. Sommige patiënten zeggen dan, nou, inderdaad, dat wil ik niet. Of die zeggen al meteen, ja, daar heb ik over nagedacht, dat wil ik niet. En er zijn ook patiënten die dan zeggen, ja, maar ik wil dat toch wel, ondanks dat jullie er niet zo achter staan. Nou goed, dan gaan we er ook in mee. Maar dat is wel iets waarin we echt heel duidelijk samen die beslissing nemen. Ja.

**I: Dat is wel een mooi voorbeeld inderdaad.**

R: Dat is meteen een heel zwaarwegende natuurlijk. Ja, dat is ook niet iets wat je in vijf minuten bespreekt. Ja.

**23.56**

**I: Denk je dat het beter zou zijn als meer gezamenlijke besluitvorming zou plaatsvinden?**

R: Ja, ik vind dat best een lastige.

**I: Omdat je een paar keer aangaf, dat is een beetje de tendens nu.**

R: Ja, in theorie denk ik van wel. In theorie denk ik dat het inderdaad heel wenselijk zou zijn als je echt samen met je arts of andere zorgverlener een besluit kunt nemen, maar ik denk gewoon in de praktijk dat de materie gewoon echt soms te ingewikkeld is voor een patiënt om te overzien. Dus dat het dan heel moeilijk is voor de patiënt. Je wilt dat de patiënt een weloverwogen beslissing neemt, maar vaak is dat toch wel heel erg moeilijk voor een patiënt. Dan is het natuurlijk aan ons om dat zo goed mogelijk uit te leggen, maar desondanks denk ik dat het soms heel erg lastig is, omdat de patiënt het toch niet goed kan overzien en wij natuurlijk door een bepaalde ervaring wel beter weten wat bepaalde keuzes inhouden. Maar ja, goed, het blijft natuurlijk moeilijk te beslissen voor een ander. Dan kom je vaak bij het aspect, wat is kwaliteit van leven? Maar ja, ik vind… Ja, dat is misschien een rare vergelijking, maar ik ben bijvoorbeeld ook tegen referenda in de politiek, omdat ik zelf vind dat wij als leken op politiek gebied – de een weet er misschien wat meer van dan de ander – niet in staat zijn om dat soort beslissingen te nemen en te beseffen wat voor consequenties dat allemaal heeft, terwijl politici in mijn ogen daar de hele dag mee bezig zijn.

**I: Die kiezen wij daarvoor en die moeten dat dan… Bedoel je dat?**

R: Ja. Nou ja, ja, maar ik denk gewoon dat wij vaak niet goed in staat zijn om bepaalde beslissingen te nemen. Ik vind het ook moeilijk om bij een referendum “ja” of “nee” te kiezen, omdat ik vaak vind dat ik er dan toch te weinig inzicht in heb. Zo denk ik dat de patiënt vaak ook toch maar een beperkt inzicht kan hebben in die gezondheidssituatie, om een goede keuze te maken. Terwijl ik over het algemeen helemaal niet paternalistisch ben ingesteld, maar het is puur dat ik echt denk van… We horen soms ook terug… Nou ga ik weer even terug naar die thuisbeademingspatiënt die bijvoorbeeld wel heeft gekozen voor beademing met zo’n tracheostoma. Er zijn regelmatig patiënten die daarna toch wel zeggen, ik weet toch eigenlijk niet of ik dit wel had moeten doen. Als ze eenmaal aan die machine liggen, niks meer kunnen, maar wel in leven worden gehouden. Dat ze dan een beslissing moeten gaan nemen te stoppen met beademing of wat dan ook. Je ziet het ook weleens bij patiënten die heel ver gaan in oncologische behandelingen, dat dat soms zoveel ellende met zich meebrengt dat je dan toch weleens terughoort van, had ik dit allemaal wel moeten doen? Ja. Maar ik vind het best een hele lastige. In principe ben ik er heel erg voorstander van, maar ik zie er toch wel behoorlijk wat beperkingen in zitten.

**I: Dus als ik jou goed begrijp – en verbeter me als ik het verkeerd zeg – is er een heel groot kennisverschil. Het kennisverschil is eigenlijk bijna te groot om in sommige gevallen dat (gezamenlijke besluitvorming) te kunnen doen. Is dat wat je zegt eigenlijk?**

R: Ja. Ja. Ja. Ja.

**I: Ik vind dat een hartstikke mooie metafoor eigenlijk, met een referendum. Ik vind hem heel duidelijk.**

R: Dus ik stem blanco meestal.

**27.17**

**I: Ja, dat kan ook. Even kijken. Ik heb hier een aantal thema’s op kaartjes gezet. Even kijken hoor. Deze. Crisissituaties. Die. Het zijn eigenlijk belangrijke thema’s… Even kijken hoor. Ja, belangrijke thema’s in de zorg voor patiënten in de palliatieve fase. Zou jij met behulp van een rangordening in de kaartjes kunnen aangeven in hoeverre die ter sprake komen tijdens jouw consulten?**

R: Nou, ik denk dat ik deze eigenlijk al helemaal onderaan kan leggen.

**I: Levensbeschouwing en culturele achtergrond.**

R: Ja.

**I: Ik zeg dat even hardop. Dat vergat ik eerst weleens, maar dan…**

R: Even aan de kant, dan heb ik meer plek. Ik denk dat ik behandelafspraken en niet-behandelafspraken over het algemeen nadrukkelijk wel probeer te bespreken, dus dat dat hoog staat. En ziekenhuisopnames eigenlijk ook, dat we op een gegeven moment ook aangeven richting patiënt of huisarts van, u komt ook niet meer naar het ziekenhuis et cetera.

**I: Ja, wat jij ook aangaf, dat je op een gegeven moment afscheid neemt van elkaar. Toch?**

R: Ja, precies. Plaats van zorg en sterven ook wel. Dit is eigenlijk een hele goede, die wettelijke vertegenwoordiging in situaties van verslechtering. Daar heb ik het eigenlijk bijna nooit over. Ja, je gaat er misschien soms vanuit dat dat op een soort van natuurlijke wijze zich wijst, maar dat is natuurlijk niet altijd zo. Crisissituaties, daarvan denk ik, ja, dat zouden we eigenlijk ook beter kunnen bespreken. Dat doe ik ook niet zo. Wilsbekwaamheid en levenseindebeslissingen, ja, dat vind ik wel een beetje lastig, dat die samen op een kaartje staan, want ik vind dat wel…

**I: Die zouden eigenlijk losgekoppeld moeten zijn.**

R: Ja.

**I: Ja, dat zijn eigenlijk ook twee verschillende dingen.**

R: Ja, maar zo zou ik ze dan neerleggen.

**I: En bijvoorbeeld de laatste drie of de laatste vier, net wat je… Dus die komen bij jou eigenlijk bijna niet ter sprake.**

R: Nee. Dat zei ik natuurlijk in het begin ook al, die levensbeschouwing en culturele achtergrond…

**I: Ja, maar die worden dus door andere zorgverleners opgepakt of door andere partijen die daar over gaan?**

R: Misschien. Het kan ook zijn dat ze soms niet aan bod komen, denk ik, of dat ze niet worden opgepakt, dat niemand dat signaleert. Dat zou best goed kunnen. Ik ben er niet alert op in ieder geval. Ja. Ja goed, die wettelijke vertegenwoordiging in situaties van verslechtering, bij wet is er natuurlijk gewoon iets vastgelegd, dat dat in eerste instantie een partner is en daarna een kind et cetera, dus ik ga er dan eigenlijk wel van uit dat dat dan in de praktijk ook gebruikt wordt en dat is natuurlijk meestal ook zo, dus het is niet zo dat ik daar de patiënt nog eens aan herinner, wat misschien wel best goed zou zijn. En crisissituaties, dat vind ik eigenlijk wel een goede. Ik bespreek eigenlijk echt nadrukkelijk crisissituaties zelden of nooit, terwijl het wel vaak crisissituaties zijn, waardoor de patiënten soms ongewenst – in onze ogen ongewenst – toch bijvoorbeeld weer in een ziekenhuisbed terechtkomen, omdat er thuis bijvoorbeeld paniek is en men dan niet goed weet hoe dan te handelen. Dan wordt bijvoorbeeld 112 gebeld en dan komt de ziekenwagen, terwijl de patiënt misschien dan bijvoorbeeld met palliatieve medicatie ook in de thuissituatie geholpen zou kunnen worden. Dus dat is eigenlijk iets wat we misschien beter zouden moeten bespreken. Ja.

**I: Ja en die middelste, daarvan vond je eigenlijk dat die uit elkaar moest hè?**

R: Over wilsonbekwaamheid heb ik het eigenlijk ook niet zo vaak, terwijl dat natuurlijk wel relevant kan zijn. Maar levenseindebeslissingen, daar heb ik het soms wel over. Het is niet dat ik uitdrukkelijk vraag of mensen euthanasie willen, maar als dat op de een of andere manier ter sprake komt, dan haak ik daar zeker wel op in. Dus dat komt soms wel aan bod. En bij de beademing kan dat ook ter sprake komen hè, dat je ook aangeeft… Mensen vragen zich natuurlijk dan soms af van, hoe moet dat dan als ik beademing heb en ik ga dood of wil dood? Dat kun je dan ook heel praktisch bespreken. Ja.

**31.38**

**I: Ik had nog wat laatste vragen over beperkte gezondheidsvaardigheden. De term is reeds gevallen. Je hebt dus weleens gehoord van die term?**

R: Ja.

**I: Wat betekent het volgens jou?**

R: Ja, ik heb de term vooral gehoord via jullie onderzoek.

**I: Ja, dat horen wij heel vaak inderdaad ☺.**

R: Ik denk dat ik daaronder versta dat een patiënt door zijn soort van intrinsieke kenmerken moeilijk in staat is om beslissingen over zijn medische toestand te nemen of dat goed te overzien. Dat klinkt ingewikkeld.

**I: De definitie voor ons is, vaardigheden van mensen om informatie over gezondheid te krijgen, te begrijpen en toe te passen. Dat is eigenlijk wat jij ook zegt, toch? Dan gaat het over functioneel, echt lezen en schrijven, maar ook over interactief en communicatief, begrijpend lezen, maar ook kritisch, dus dat je ook informatie ordent en toepast en dat soort dingen. Hoe vaak heb jij te maken met patiënten die beperkte gezondheidsvaardigheden hebben?**

R: Ja, toch wel met enige regelmaat. Ja, ik vind het moeilijk om daar echt een getal op te zetten.

**I: Dus regelmatig.**

R: Echt regelmatig. Ik denk dat er zeker elk spreekuur wel één of meerdere patiënten bij zitten die toch wel hier aan voldoen. Ja.

**I: Hoe herken je die patiënten?**

R: Ja goh, over het algemeen denk ik dat dat snel kan opvallen aan de respons die je van de patiënt krijgt, dat je toch probeert in te schatten of iemand het begrijpt. Soms ook door vragen die je stelt, dat je merkt dat mensen daar helemaal niet goed antwoord op kunnen geven. Dus aan de manier waarop ze communiceren met jou.

**I: Op welke manier probeer je rekening met deze patiëntengroep te houden in de communicatie in het algemeen?**

R: Ja, toch door het wat simpeler te schetsen, of door wat eenvoudiger taalgebruik te gebruiken, hoewel ik me er zelf vaak op betrap dat ik… Ik denk vaak dat we het toch nog te ingewikkeld doen. Dan hoeft een patiënt nog niet eens beperkte gezondheidsvaardigheden te hebben. Ik denk over het algemeen dat wij als artsen toch vaak nog dingen die voor ons… Dan heeft het soms niet eens met termen te maken. Elke arts weet in theorie wel dat je natuurlijk lekentermen moet gebruiken, dat soort dingen, maar los daarvan denk ik dat wij soms toch… Ja, als je zelf jaar in jaar uit bepaalde verbanden trekt of je bepaalde dingen logisch vindt, dat dat op een gegeven moment voor een patiënt helemaal niet zo is, dat verliezen wij denk ik toch makkelijk uit het oog en dat is voor patiënten met beperkte gezondheidsvaardigheden natuurlijk extra lastig. Maar ik probeer daar dus wel een beetje voor te waken. Ik merk dat ook weleens bij de supervisie van anderen, van arts-assistenten bijvoorbeeld, ook die pas beginnen soms… Dan zit ik er naast. Dan leggen ze de patiënt bijvoorbeeld uit wat een patiënt heeft en waarom we de behandeling toepassen. Dat ik dan soms echt merk van, ja, maar dit kan de patiënt echt niet begrijpen. Dan kun je toch wel horen dat het echt nog te ingewikkeld is.

**35.02**

**I: Maar dat zit hem dan puur in de manier waarop het wordt uitgelegd?**

R: Ik denk dus enerzijds soms aan termen die gebruikt worden, maar ook of… Ja, hoe moet ik dat nou uitleggen?

**I: Misschien bedoel je die verbanden die je dan trekt.**

R: Ja, precies. Ik kan niet echt een heel goed voorbeeld bedenken, maar dat wij het bijvoorbeeld logisch vinden dat als je ademhaling faalt, je op de IC een bepaalde vorm van beademing kan krijgen. Dat is voor ons dan heel logisch. Wij weten wat er op een IC allemaal gebeurt. Maar als jij tegen de patiënt alleen maar zegt, goh, het gaat slechter met u, misschien moet u wel naar de IC, ja, dat zegt zo’n patiënt helemaal niks. Dan heb je misschien niet per se moeilijke taal gebruikt, maar dan overschat je toch de kennis die mensen denk ik hebben over het gezondheidssysteem. Ja.

**I: Ervaar je ook ooit barrières in de zorg aan patiënten die beperkte gezondheidsvaardigheden hebben?**

R: Ja. Ik denk wel dat het soms voor mensen met beperkte gezondheidsvaardigheden moeilijker is om hun klachten goed te uiten, dus dat dat een probleem kan zijn, dat ze met bepaalde dingen zitten maar het niet goed kunnen overbrengen, dat ze soms ook niet goed weten dat je daarmee bij iemand terecht kan of bij wie je daarmee terecht kan. Ik denk dat dat soort dingen wel nadelig kunnen werken.

**I: Dus dan is het voor jou een barrière dat ze eigenlijk niet goed kunnen teruggeven aan jou wat er aan de hand is.**

R: Ja, bijvoorbeeld. Ja. Ja, toevallig om een voorbeeld te geven, we hebben nu een patiënt op de afdeling met ook een eindstadium COPD. Het is niet zo’n hele intelligente patiënt. Ze heeft waarschijnlijk ook nog allerlei psychiatrische problematiek met persoonlijkheidsproblemen en een depressie. Ze heeft ook nog een verleden geloof ik met misbruik. Nou, dus heel zwaar belast.

**I: Oei, klinkt ingewikkeld…**

R: Die vrouw zegt eigenlijk al een tijdje, ik wil dood, of, ik wil euthanasie. Maar omdat zij dat zo moeilijk kan verwoorden kun je dat eigenlijk niet echt inwilligen, terwijl het misschien wel een hele reële wens is. Maar ja, om in aanmerking te kunnen komen voor euthanasie moet je natuurlijk wel goed kunnen verwoorden waarom je ondraaglijk lijdt, dat het een weloverwogen keuze is. Maar als jij natuurlijk maar zo beperkt kunt communiceren, dan is het heel moeilijk om daarop in te gaan, want dan twijfel je als zorgverlener natuurlijk van, ja, is dat wel echt weloverwogen? Ja, dan kan het zo zijn dat als dat een intelligente mevrouw was, dat die misschien wel - ja, dat is een heel extreem voorbeeld natuurlijk - euthanasie zou kunnen krijgen, maar dat dat bij deze patiënt niet lukt, terwijl die wens misschien net zo sterk is en net zo reëel is. Maar als je het niet kunt verwoorden is het toch lastig. Ja.

**37.55**

**I: Wat zou jij nodig hebben om zorg beter aan te laten sluiten bij patiënten met beperkte gezondheidsvaardigheden?**

R: Dan heb je het over een patiëntencategorie die vaak misschien zwakbegaafd is of een lichte handicap heeft, die hebben soms toch een begeleider. Dat kan een externe begeleider zijn. Ik merk wel dat dat heel erg kan helpen, dat patiënten een begeleider… Dat kan natuurlijk ook een familielid zijn of echt een begeleider van extern, die meehelpt om wat gezegd wordt te vertalen, zodat de patiënt dat ook kan begrijpen. Dus dat kan denk ik meehelpen. Maar dat zou misschien ook bij wijze van spreken een verpleegkundige op je eigen poli kunnen zijn, die erbij zit en die bijvoorbeeld daarna nog even een paar minuutjes met de patiënt kan gaan zitten om even te verifiëren of alles duidelijk was. Dat is natuurlijk mooie toekomstmuziek, wat natuurlijk in de praktijk waarschijnlijk moeilijk te realiseren is, maar ik denk een extra persoon die mee kan helpen en die mee kan luisterne en die nog eens kan verifiëren, ik denk dat dat wel zou kunnen helpen. En meer tijd.

**I: Ja, die is heel praktisch inderdaad.**

R: Ja. En misschien ook wel meer scholing voor ons, dat er bepaalde tips zijn of dat je eens een keer inderdaad gespiegeld wordt, van, je denkt wel dat je het simpel vertelt, maar dat is helemaal niet zo. Dat je daar zelf in wordt opgeleid of getraind, dat kan vast ook helpen.

**I: Wie zou dat dan moeten doen, dat spiegelen?**

R: Een communicatiespecialist.

**I: Of bijvoorbeeld jouw collega’s of zo? Zoiets.**

R: Ja, dat zou ook kunnen. Ja. Ja. Dat zou sowieso goed zijn. Daar zijn er wel vaker plannen voor, maar wij doen het hier heel concreet nog niet. We hebben het er weleens over gehad, van, het zou goed best zijn als we af en toe nog eens een keer een consult opnemen en eens even kijken wat er goed aan gaat en wat er misschien minder goed aan gaat. Ja.

**I: Ja, dat waren mijn vragen. Heb jij daar nog iets aan toe te voegen of heb je daar nog vragen over?**

R: Nee.

**I: Oké.**

**[40.04 – einde opname]**

Z1long05

I = interviewer

R = respondent

I: Ja, dank u wel. Even kijken hoor, ik heb aan het einde ook nog even deze korte vragenlijst voor u. En dan, ja, dan zijn we echt klaar.

R: Ja.

I: Maar goed, het interview. Allereerst fijn dat u tijd heeft en mee wilt doen. Heel fijn. En normaal zou ik bijvoorbeeld zorgverleners spreken en dan zou ik het graag gaan hebben over, hè, over uw rol in de palliatieve zorg. Over communiceren en informeren en gezamenlijke besluitvorming. Maar ook over beperkte gezondheidsvaardigheden. Maar u bent natuurlijk meer dan zorgverlener alleen. Dus ik dacht misschien, maar dat is, ik laat dat vaak even bij de geïnterviewde, is het misschien ook interessant om wat meer over het ziekenhuis / de organisatie van het ziekenhuis te weten.

R: Ja, ik zal kijken, hoever wij daarmee komen. Want het ziekenhuis is heel groot hoor.

I: Maar bijvoorbeeld ook vanuit uw rol in het expertisecentrum. Nou heb ik natuurlijk A. ook over gesproken.

R: Ja.

I: Dus, misschien is het wat als ik hier gewoon mee begin en ik en, ja, de tijd die ik over heb, dan kunnen we misschien dan terug,

R: Terug, ja.

I: en dan misschien nog ook die andere zaken.

R: Ja, is goed. Laten we maar even kijken hoever we komen.

I: Oké, nou, dat is fijn. Even kijken. Mijn eerste vraag is, is er beleid binnen uw ziekenhuis om de zorg toegankelijker te maken voor patiënten met beperkte gezondheidsvaardigheden?

R: Ha, als het er al is dan weet ik niet dat er een officieel beleid is.

I: Oké. Deze had ik al verwacht want dat zei A. ook precies zo.

R: Ja.

I: Ja, oké.

R: Nee.

I: Oké.

R: Nee, dus ik weet het, nee.

I: Oké, en ja, toen heeft A. gezegd van, joh, je moet even hier naar de, weer terug naar de eerste verdieping,

R: Ja.

I: van het ziekenhuis en daar heb je patiëntenvoorlichting. Daar ben ik ook al geweest,

R: Ja.

I: maar die hadden inderdaad ook hetzelfde antwoord.

R: Hetzelfde antwoord.

I: Ze waren er wel mee bezig dus ik weet niet hoe dat.

R: Nou, ik heb in ieder geval, er is niks gecommuniceerd of, en ik hou het redelijk in de gaten.

I: Oké.

R: Voor zover ik weet niet, nee.

I: Oké. Ja, mijn volgende vraag zou dan zijn, worden er binnen uw organisatie methodieken/instrumenten gebruikt om zorg toegankelijk te maken voor mensen met beperkte gezondheidsvaardigheden?

R: Nee, nee, niet dat ik weet. Tenzij jij het ook hebt, maar dat is niet echt beperkte gezondheidsvaardigheden, meer over communicatieproblemen qua taal, daar wordt wel actief, is net iets anders maar wordt wel actief geadviseerd om tolken in te zetten maar, ja, dat is net weer iets anders. Dat is wel om de communicatie natuurlijk te verbeteren.

I: Ja, oké, ja. Maar wat zou dan volgens u belangrijk zijn voor uw ziekenhuis om die zorg, mensen met beperkte gezondheidsvaardigheden, toegankelijker te maken?

R: Heel veel bewustwording. Want we weten al, dat ook mensen zonder beperkte gezondheidsvaardigheden nog geen 10% van een consult onthouden. Ik weet niet wat, het precies is voor mensen beperkte gezondheidsvaardigheden. Maar dat zal ongetwijfeld nog minder zijn. Dus ik denk bewustwording bij hulpverleners dat al hun goede bedoelingen in ernstig beperkte mate aankomen.

I: Ja.

R: Dat zou wel heel goed zijn, denk ik.

I: Oké. Ja, en welke mogelijkheden ziet u bijvoorbeeld? Dus aanvullend aan bewustwording?

R: Hoe je dat dan zou moeten doen? Ik denk dat je, dat het meest aansprekende is werken met voorbeelden. Wij hebben hier verschillende, onder andere hele goede tussen de middag besprekingen waarbij casuïstiek ter sprake komt, waar meerdere mensen bij betrokken zijn, al dan niet met fouten. En daar zou je bijvoorbeeld eens een keer kunnen laten zien, misschien zelfs aan de hand van één van de interviews die jullie hebben gehouden, van, hoeveel komt er nou aan bij iemand? Of hoe worden bepaalde informatie, hoe wordt dat door een patiënt gehoord? En, ja, je zult, ik denk met voorbeelden werken dat dat het meest binnenkomt.

I: Oké.

R: Omdat, weer een nieuwe folder maken gaat ‘m niet worden.

I: Nee, precies.

R: Nee.

I: Dus met voorbeelden om bij de zorgverlener binnen te komen?

R: Ja, exact.

I: Oké. Ja, wat, en welke barrière ziet u op organisatieniveau?

R: Tijd.

I: Oké, ja. Die had ik eerlijk gezegd ook al verwacht.

R: Ja, dat, dit kost gewoon tijd.

I: Ja.

R: En als je bij wijze van spreken mensen met beperkte gezondheidsvaardigheden coaches mee zou nemen of vertalers of hoe je het dan ook zou willen noemen, ja, dat kost gewoon tijd.

I: Ja.

R: En dat is het grote probleem.

I: Oké. Oké. En dus tijd is een barrière. En wie zou daar volgens u een rol in moeten spelen? Dus u heeft als barrière tijd, wie zou dan die rol, die barrière moeten wegnemen?

R: Ja, dat is niet zo makkelijk natuurlijk.

I: Zou dat bijvoorbeeld een rol zijn voor uw expertisecentrum?

R: Wie moet dat doen? Dat weet ik niet zo goed want dat ligt er een beetje aan hoe je het gaat… Kijk, zo gauw iemand bij een dokter zit is die dokter, moet zijn tijd afmaken. Dan zou je al een vertaling, iemand mee moeten nemen, een coach die daarna een nagesprek doet, een verpleegkundige, een casemanager, die je dan mee gaat geven. Dus daar zou een oplossingen kunnen zitten misschien.

I: Oké.

R: En of dat nou voor specifiek voor het expertisecentrum is, dat zou kunnen, onze verpleegkundigen die meegaan.

I: Ja. Oké.

R: Dat ligt een beetje aan het gesprek natuurlijk. Maar als het puur, een oncologisch gesprek is waarbij uitgelegd moet worden dat de in opzet curatieve behandeling niet meer kan dan heb ik liever een oncologie verpleegkundige.

I: Oké.

R: Maar ik denk dat de verpleegkundige beroepsgroep hier wel heel goed voor zou zijn.

I: Ja, oké. Ja, dat was eigenlijk mijn volgende vraag inderdaad, op welk niveau zou dat in het ziekenhuis dan zichtbaar moeten zijn? Even kijken, en zou u daar bijvoorbeeld specifiek beleid op willen?

R: Ik weet niet goed hoe groot het probleem is.

I: Ja.

R: Dus daar zal het wel vanaf hangen. Als het een groot probleem is dan moet je dat zeker maar ik weet niet precies hoe groot het probleem is.

I: Ja, en dan bedoelt u dit probleem met beperkte gezondheidsvaardigheden,

R: Ja.

I: in het ziekenhuis? Oké.

R: Ja. Hoeveel mensen hier echt behoefte en dus straks ook profijt van zouden hebben.

I: Ja.

R: Of je daar specifiek beleid op moet gaan lopen maken.

I: Ja, oké.

R: Want wat ik net al zei, hè, van alle patiënten is, komt maar 10% aan. Dan zou het op een hele andere manier en dan kun je niet met coaches gaan werken want dan is dat veel te arbeidsintensief. Dus, ja hoe groot is dit probleem?

I: Ja, dat is wel. Ja, oh, zo’n patiënten coach die erbij zit?

R: Ja, bijvoorbeeld, ja.

I: Oké, ja. Oké. Is het misschien wat als we nu dan verschuiven naar uw rol als zorgverlener, in de palliatieve zorg?

R: Dat is goed.

I: Oké. En wat is uw rol in de zorg voor patiënten in de palliatieve fase?

R: Ha.

I: Ik denk, laat ik ‘m breed neerleggen.

R: Laat ik breed beginnen. Ik ben gewoon, vanaf het begin betrokken bij de opzetten van de palliatieve zorg hier in … (naam stad ziekenhuis). Daarin hebben we, iedereen in palliatieve zorg een soort handjes op rug rol. Dat wil zeggen, een adviserende rol, naar alle andere professionele hulpverleners die te maken krijgen met patiënten in de palliatieve fase. Zowel binnen als buiten het ziekenhuis. Dus je kunt denken aan artsen, verpleegkundigen maar ook maatschappelijk werk, psychologen. Iedereen mag ons bellen voor advies. En wij houden het dan ook adviserend. En dat advies kan puur telefonisch maar we kunnen ook bedside consulten doen. Waarbij we al dan niet samen met de primaire hulpverlener naar een patiënt toegaan.

I: Oké. Dus de centrale zorgverlener die is, dat zijn jullie eigenlijk meestal niet? Jullie zijn adviserend?

R: Nee, als palliatieve zorg zijn we dat zeker, zijn we dat meestal niet, zijn wij dat nooit niet.

I: Oké.

R: Nee, en mijn andere pet is oncologische pijnbestrijding, daar ben ik behandelaar. Maar in de palliatieve zorg zijn we eigenlijk alleen maar adviserend. Met name omdat wij ook de zorg en de regie in één hand willen houden. Die ligt dan of bij de oncologische patiënt, bij de oncoloog. Of op een gegeven moment bij de huisarts.

I: Ja, en u wilt dat dat blijft?

R: Ja.

I: Dat dat duidelijk is voor de patiënt.

R: Dat dat heel duidelijk is voor de patiënt, ja.

I: Oké. En is het, denkt u, ook duidelijk voor de patiënt wie dan altijd de centrale zorgverlener is?

R: Dat doe, daar doen we heel erg ons best voor in dit ziekenhuis omdat heel duidelijk te maken. Er zijn ook briefjes, in ieder geval bij het oncologie centrum, waar op staat, wie is uw hoofdbehandelaar?

I: Oh zo, ja.

R: Dus bij wie moeten mensen terecht? En soms staat een hoofdbehandelaar en telefoonnummer van bijvoorbeeld verpleegkundig specialist. Wij proberen heel erg hard om dat heel duidelijk te hebben, ja.

I: Ja. En u gaf net al aan, hè, dat u dus een adviserende rol in het ziekenhuis heeft. Maar hoe werkt u bijvoorbeeld samen met de huisarts?

R: Huisartsen kunnen ons bellen 24/7.

I: Oké.

R: En met, ja, met vragen, whatever, hé, dat mag puur op symptomen zijn maar het mag ook organisatorisch zijn, het mag even sparren zijn, het mag. En dat sparren gaat dan meestal over palliatieve sedatie.

I: Oké.

R: Het mag zijn om even stoom af te blazen, nou, whatever.

I: Ja, oké.

R: Hoe wij iemand ook maar kunnen helpen, ja.

I: Ja, eigenlijk heel breed.

R: Heel breed, ja, het is heel breed. En we proberen ook echt wel op alle vier de dimensies van die palliatieve zorg te zitten. Dat gaat telefonisch wat lastiger dan bedside natuurlijk.

I: Ja.

R: Want telefonisch is er vaak een vraag.

I: Ja.

R: Iemand heeft pijn, wat moet ik doen, hé, maar aan bed is dat veel makkelijker, ja.

I: Ja. Nu ga ik even iets invullen maar dan bedoelt u bijvoorbeeld het spirituele aspect?

R: Ja.

I: Omdat via de telefoon te bespreken is misschien wat lastig, bedoelt u dat misschien?

R: Dat, ja, het is lastiger.

I: Hoe bedoelt u dat?

R: En het is, als iemand belt, belt hij met een specifieke, vaak met een specifieke vraag waar antwoord op verwacht wordt. En als je, dus een, als huisarts, als die een, om die als voorbeeld te nemen, een probleem heeft met een pijn probleem dan zit je er niet op te wachten dat zo’n consulent ook nog naar het hele sociale systeem vraagt. Omdat je het gevoel hebt dat je dat wel in grief hebt.

I: Oh zo.

R: Je wilt hulp hebben op het punt waar je zelf even tekortschiet of waar je het niet goed weet.

I: Ja, oké.

R: Ja.

I: Oké. En werkt u verder bijvoorbeeld nog samen met, of jullie, hè, jullie advies, sorry, jullie expertisecentrum, ook met maatschappelijk werk?

R: Ja.

I: Oké.

R: Wij hebben 0.1 (fte) geloof ik, een maatschappelijk werkende erbij, in het team zitten. En ook een geestelijk verzorger.

I: Oké.

R: Wij hebben wekelijks natuurlijk onze MDO’s en daar zitten zij allemaal bij. Daar zit ook de eerstelijns bij. Dus dat is veel uitgebreider.

I: Oké. Dus jullie hebben binnen jullie expertisecentrum ook een MDO?

R: Ja, één keer in de week.

I: Oké.

R: Daar worden alle patiënten besproken over wie er een vraag is gesteld in de afgelopen week.

I: Ah zo, dus over wie er advies gevraagd is die worden binnen het, MDO besproken? Oké.

R: Ja, en dat is echt multidisciplinair.

I: Oké. Oké. Mag ik wat vragen over communiceren, informeren en gezamenlijke besluitvorming? Want, even kijken hoor, ja, want ik had het ook gelezen in uw, hier in dit script. Het fysieke, psychische sociale, en spirituele welzijn. En in hoeverre komen deze verschillende aspecten aan bod in uw gesprekken met de patiënt?

R: Ik hoop bijna altijd.

I: Oké.

R: Want het is altijd lastig om jezelf, om dat van jezelf te zeggen.

I: Ja.

R: Maar, ja, dat is nou juist een onderdeel van de palliatieve zorg dat je veel breder kijkt dan puur dat stukje lichamelijk. Je zou ook niks kunnen oplossen, bijna niks kunnen oplossen, als je niet naar de vier dimensies zou kijken. Ook een pijnprobleem is bijna nooit een puur fysiek probleem. En er zit de angst, en de boosheid en het verdriet, en alles zit daar omheen. Als je dat niet bespreekbaar maakt dan gaat het, het gaat ‘m niet worden. Patiënten zijn wel eens verbaasd hoor, dat ik daar naar vraag. Je moet er wel naar vragen ook, mensen komen er niet zelf mee. Mensen komen er dus niet zelf mee.

I: Oké, dus u moet het echt zelf ter sprake brengen bij patiënten?

R: Ja. Omdat mensen onbewust kiezen wat ze vertellen aan iemand. En dat is bijvoorbeeld ook waarom oncologen altijd denken dat er nauwelijks pijn is omdat de patiënt dat niet tegen een oncoloog zegt dat hij pijn heeft. Want dan krijg ik misschien wel geen chemo of dan…

I: Want dan krijgen ze misschien geen chemo of, ja. Dat las ik, ja.

R: …krijg ik dit, ja, precies. Dus patiënten zijn ook heel verbaasd als ze bij de pijndokter komen, en zeker als ik mijn pijn-pet op heb, dat ik ook ga vragen hoe het overdag gaat. En hoe zij zich, ja, wat voor hen zin heeft, wat het leven zin geeft.

I: Ja.

R: Dan moeten ze even, vaak zie je mensen heel erg schrikken van, oh. Maar dan, ja, het ligt er een beetje aan hoe open jouw houding is en hoe de patiënt is maar dan komt dat vaak wel. En dan heeft het alleen maar meerwaarde.

I: Ja, oké. Want dat wilde ik inderdaad vragen, is dat bij alle patiënten zo dat u dat zelf ter sprake brengt?

R: Ja.

I: Oké. En is dat bijvoorbeeld ook bij mensen die laagopgeleid zijn? Is dat hetzelfde?

R: Ja, dat is hetzelfde.

I: Oké.

R: Dan is het misschien nog makkelijker.

I: Oké. Waarom?

R: Omdat die makkelijker praten vaak. We, ja, dat is een beetje, dat geldt natuurlijk lang niet voor iedereen. Maar ik heb het idee dat hoger opgeleiden meer voor zichzelf, meer voor zichzelf houden. En bij lager opgeleiden makkelijker is om het erover te hebben van, goh, wat maakt dat voor jou nou waard? Wat doe je de hele dag? Ja, dat dat, eenvoudiger is.

I: Oké. Oké. Want u gaf net ook aan van, hè, het is een beetje afhankelijk van wie de persoon is.

R: Ja.

I: En of hoe open de houding is. Maar hoe werkt dat dan? Is dat een inschatting die u maakt, of?

R: Nee, dat is de reactie die je krijgt. De vraag stel ik altijd.

I: Oké.

R: En het ligt ook aan de begeleider natuurlijk, hè, die ernaast zit.

I: Ja, oké.

R: En hoeveel er ingevuld wordt voor een patiënt. Want dat kan ook wel eens heel lastig zijn.

I: Oké. Kunt u daar iets meer over zeggen hoe het ingevuld wordt voor? Dus hoe een patiënt?

R: Dat als een patiënt die zelf minder vaardigheden heeft, iemand bij zich heeft, zijn broer, begeleider, die te beschermend is die gaat alles lopen in, die gaat lopen invullen voor een patiënt, “nee, het gaat wel goed met hem”.

I: Oh zo, op die manier.

R: Zo, bedoel je, nou ja, precies.

I: Oké. Oké.

R: Dat je de patiënt zelf niet te spreken krijgt, zeg maar.

I: Ja, ja. Oké, ja. Oh zo. Want ik dacht dat u bedoelde dat bijvoorbeeld zo’n behandeling, dat iemand bijvoorbeeld al heel lang bij een oncoloog loopt, zeg maar.

R: Ja.

I: En dat diegene eigenlijk al, ook al een soort van ‘geframed’ is op behandelen, omdat de betreffende oncoloog daar toevallig zo in staat. En dan komt iemand misschien bij een palliatief adviseur en dan komen ineens hele andere dingen ter sprake.

R: Nou, dat is wel, waar soms mensen enorm, ja, even raar van staan te kijken. Eigenlijk krijg je altijd wel antwoord. En je merkt gelijk aan iemand van, oké, tot hier en niet verder. Of ik mag wel doorvragen, ja, dat heb je natuurlijk wel gelijk door.

I: Oké. En die aspecten, hè, waar we het net ook over hadden, fysiek, psychisch, sociaal, spiritueel. Worden die vastgelegd in een individueel zorgplan van de patiënt of?

R: Nou, nog onvoldoende. Dat zou natuurlijk allemaal terug te lezen moeten zijn.

I: Oké.

R: Maar als ik naar mezelf kijk dan zou dat wel iets netter kunnen.

I: Oh zo.

R: We proberen het bij de palliatieve verslaggeving, proberen we het wel, beter dan in oncologische pijn verslaglegging.

I: Oké.

R: Bij de, als wij het voor puur palliatief, ja, hoe noem je dat? Een geoormerkt consult doen dan zijn wij daar iets braver in. Dan proberen we daar wel meer van op te schrijven.

I: Dus dan is er echt een individueel zorgplan voor die patiënt, of is dat teveel gezegd?

R: Nee, het is meer onze eigen verslaglegging over een consult waarin wel alle aspecten naar voren komen maar een patiënt, als je een individueel zorgplan hebt dan zie ik iets voor me wat bij de patiënt is. Dus we moeten kijken of we het over hetzelfde zorgplan hebben.

I: Oh zo, ja.

R: Ik zie met een individueel zorgplan, zoals het in het kader ook beschreven wordt, een plan zoals dat bij de, met de patiënt meereist en waar de zorgverleners hun steentje aan bijdragen. En wat wij doen is onze eigen verslaglegging, weliswaar op de vier dimensies, maar dat is niet iets wat met de patiënt meegaat.

I: Oké. Ja. Dan bedoelde ik denk ik die laatste.

R: Iets wat met de patiënt meegaan?

I: Ja, dat denk ik wel.

R: Nee, dat hebben we niet.

I: Oké. Zijn er patiënten waarbij u de communicatie moeilijker vindt?

R: Die zijn er natuurlijk altijd. Maar jij wil weten of er een bepaalde groep is natuurlijk, een, of ik mensen kan framen. Ik denk, even denken hoor, nee, ik denk niet dat ik bepaalde groep kan veralgemeniseren waarvan ik zeg van, nou, als die binnenkomen, dat vind ik toch wel heel lastig.

I: Oké. En zijn er dan mogelijk momenten dat u uw communicatie bewust aanpast aan de situatie?

R: Dat je doe je natuurlijk ook altijd.

I: Oké. En verschilt dat dan misschien bij verschillenden, ja, groepen patiënten?

R: Ook daar, mijn, ik denk mijn inschatting van hoger en lager opgeleid, ik vraag altijd, redelijk snel in een consult wat iemand voor een werk doet of werk heeft gedaan.

I: Ja.

R: Waardoor je een beetje een inschatting kan maken.

I: Ja.

R: Je hoort ook vaak heel snel of mensen in het medische vak zitten, medische kinderen, nichtjes, neefjes hebben. Vaak laten mensen dat ook al heel snel weten, daar kun je ook je taalgebruik wat op aanpassen. Of ik praat tweetalig. Als één van de twee wel medisch geschoold is en de ander niet dan probeer ik altijd medische plus vertaalde term, medische term plus vertaal term, zodat je iedereen toch een beetje op zijn niveau aanspreekt. En dat werkt het beste want dan krijg je vertrouwen.

I: Oké, ja. Oké, dus dan past u uw communicatie op het uw ingeschatte niveau van de patiënt aan?

R: Ja.

I: Als ik u goed begrijp.

R: Ja, ja, dat probeer ik in ieder geval wel.

I: Oké, oké. En checkt u weleens bij uw patiënten of zij u hebben begrepen?

R: Ja.

I: Oké.

R: Ik vraag altijd terug van, kunt u nog eens herhalen hoe wij dat gezegd hebben? Of, snapt u hoe u de medicatie nu moet nemen, of terugnemen? Of ja, ik probeer dat wel te checken, ja.

I: Oké.

R: Ja. En als ik denk dat dat het echt niet aangekomen is dan schrijf ik bijvoorbeeld, de medicatie schrijf ik op. Dat doe ik ook lang niet bij iedereen.

I: Oké.

R: Als ik soms mijn twijfels heb van, jongens, is dit wel helemaal aangekomen? Dan schrijf ik het even uit, ja.

I: Oké. En zijn er nog andere hulpmiddelen? Bijvoorbeeld zoals het opschrijven bijvoorbeeld, die u gebruikt?

R: Nee, ik vrees van niet.

I: Nee, geen filmpjes of visueel voorlichtingsmateriaal?

R: Nee. En ik heb ook nog zelden meegemaakt dat mensen zelf willen opnemen.

I: Oh zo.

R: Wel eens een keer.

I: Oh ja.

R: Ja, dat is natuurlijk, dat zouden patiënten zelf kunnen doen.

I: Ja, ja.

R: Maar dat, in mijn optiek komt dat heel weinig voor. Tenminste bij mij komt dat heel weinig voor.

I: Ja, ja. Even kijken hoor. In hoeverre is er volgens u sprake van een gezamenlijke besluitvorming met patiënten in de palliatieve fase?

R: Als het ergens moet dan hoop ik toch dat het in de palliatieve fase is.

I: Ja, zeg dat wel.

R: Desalniettemin blijft dat hele gezamenlijke besluitvorming natuurlijk een super moeilijk iets. Want een patiënt moet volledig op de hoogte zijn.

I: Zeker.

R: En hoe kan een patiënt volledig op de hoogte zijn? Dat zal ik of mijn collega’s ‘m toch moeten vertellen. Dat zou zo maar ook gekleurde informatie kunnen zijn.

I: Hoe bedoelt u met gekleurde informatie?

R: Als ik er ernstig van overtuigd ben dat iets goed is voor de patiënt, dan zal ik dat anders brengen dan als ik denk dat het misschien niet zo goed is voor een patiënt.

I: Oh zo.

R: Daar zit altijd, je eigen mening zit daarin. Hoe je het ook wendt of keert. Dat kun je wel proberen omdat niet te hebben maar dat is natuurlijk niet zo. Lang niet iedereen zit erop te wachten om een keuze te hebben. Doet u maar wat u goed vindt dokter. Het is soms, denk ik, ook niet eerlijk om mensen de, ja, de keus te geven klinkt misschien stom. Een voorbeeld uit mijn eigen kring, mijn moeder moest voor haar ernstig geestelijk en lichamelijk gehandicapte broer beslissen of er doorgegaan moest worden met sondevoeding, ‘ja’ of ‘nee’. En zij heeft mij toen wel gebeld. Ik zeg, stoppen want dat had geen bijdragende functie meer. Maar mijn moeder heeft dat moeten beslissen. Ze heeft nog steeds het gevoel dat zij haar broer, ja, niet vermoord heeft, zeg maar, maar zo. Je moet, denk ik, heel erg oppassen om mensen, ze moeten natuurlijk wel zelf meebeslissen. Maar ze hoeven op een gegeven moment ook niet in hun eentje de verantwoordelijkheid te dragen voor beslissingen waarvan zij het gevoel hebben dat ze leven of dood beslissingen nemen. Dus wel gezamenlijke besluitvorming maar zonder je terug te trekken en je verantwoordelijkheid niet te nemen.

I: Ja.

R: Dat vind ik wel erg belangrijk.

I: Maar dat is inderdaad wel een fraaie nuance op het besluitvormingsverhaal. Dat je dus inderdaad die verantwoordelijk wel houdt.

R: Ja.

I: En deelt in iedere geval.

R: Op zijn minst deelt.

I: Ja.

R: Ja, precies. En niet iemand opzadelt met beslissingen waar zij de rest van hun leven last van hebben.

I: Ja, ja. Zoals bijvoorbeeld uw moeder dan in dat geval.

R: Ja, ja, precies.

I: Ja.

R: Dat vind ik altijd, ik schrok daarvan dat zij daar zo’n last van had.

I: Ja, inderdaad.

R: Daar heb ik wel veel van geleerd.

I: Ja. Ja.

R: Ik denk, oké, neem je verantwoordelijkheid ook als zorgverlener.

I: Ja. Oké. En is dit voor alle patiënten hetzelfde, denkt u?

R: Nee, je hebt natuurlijk mensen die keihard de regie willen houden, die heel duidelijk weten wat ze willen. En je hebt mensen die echt geen idee hebben en doet u maar wat het beste is, dokter.

I: Oké.

R: En die zijn allebei moeilijk natuurlijk.

I: Ja.

R: De makkelijkste zitten er ergens tussenin. En gelukkig zitten de meesten er ergens tussenin dus dat scheelt.

I: Ja. Ja, en kunt u misschien een voorbeeld geven van een situatie waarin sprake was, of heel duidelijk sprake was van gezamenlijke besluitvorming? En een voorbeeld waar dat juist niet gebeurde.

R: Ik denk dat bij mij op de pijnpoli bijna altijd heel duidelijk wel gemeenschappelijke besluitvorming is. Want bijna iedereen komt binnen van, ik hou niet zo van pillen, dokter. Ik moet altijd praten als brugman om er opioïden eventueel in te krijgen. En waarom ik dat zou willen. En waarom ik eventueel Methadon zou willen, want dat is nog moeilijker dan die gewone Morfine. En daar is altijd, ja, ik leg uit, geef ook aan, luister, u bent de baas. U voelt uzelf. Wat gaan we doen? Gaan we proberen? Ja, ik denk dat dat altijd een gezamenlijke besluitvorming is.

I: Oké.

R: En helemaal niet, ja, dat komt eigenlijk zelden tot nooit voor.

I: Oké, nou ja, dat is toch eigenlijk wel positief dan toch?

R: Ja.

I: Ja.

R: Je moet ook niet de illusie hebben, want de patiënten doen toch niet wat jij zegt. Dus dat scheelt enorm.

I: Dat is alvast goed om vanuit te gaan, zeg maar. Denkt u dat het beter zou zijn als er meer gezamenlijke besluitvorming plaats zou vinden?

R: Ja, dat vind ik een beetje een rare open vraag. Want wat is meer, wat is minder? Hoeveel is er al? Er zal altijd gezamenlijke besluitvorming plaats moeten vinden want, ja, jij bent maar hulpverlener. Een patiënt is de baas over zijn eigen lijf dus, ja. Wij zijn eigenlijk altijd adviserend. Dat is de wet BIG ook, ik ben niet de baas, ik loop niet te zeggen, u moet dat doen. Dat kan ik ook helemaal niet.

I: Nee. Nee, oké. Dan laat, dan ga ik ‘m proberen anders te stellen.

R: Ja.

I: Als u vindt dat het beter zou kunnen, wat zou er dan voor nodig zijn? Wat zou kunnen helpen bijvoorbeeld?

R: Een betere, weet ik niet, ja, dat vind ik een lastige. Want waar? Dan moet ik eerst weten waar het scheef loopt.

I: Ja, hij is inderdaad erg open, dat ben ik met u eens.

R: Ja, precies, ja. Ja. Het begint bij, denk ik, toch de houding van de zorgverleners. Die open moeten staan voor het, voor de dialoog en niet voor het vertellen hoe het moet.

I: Dus het directieve, de directieve houding?

R: Ja, die moet los.

I: Ja, daar moeten we vanaf.

R: En daarbij moet je heel goed beseffen dat er meer wegen zijn naar Rome en dat sommige mensen echt hele andere ideeën hebben. En het zal ook heel erg afhangen van waar we het net over hadden, hoe sterk je zelf denkt dat iets goed of niet goed voor iemand is. Kijk, als iemand binnenkomt bij een oncoloog en die is ervan overtuigd dat hij geen chemo wil maar dat Canabis zijn kanker zal gaan genezen, dan ga je automatisch vanuit je kennis directief te werk, denk ik. Omdat je gewoon weet dat dat gewoon niet goed is voor iemand. Dus dit zal ook heel erg ook van de situatie afhangen.

I: Ja, ja.

R: Terwijl als er duidelijk twee of drie, open keuzes zijn die in, op medische gronden gelijkwaardig zijn, ja, dan is het natuurlijk veel makkelijker om gezamenlijke besluitvorming te nemen.

I: Ja, ja.

R: Dus, ja, de situatie maakt ook wel wat uit.

I: Ja, precies. Ja. Oké. Mijn laatste onderdeel, beperkte gezondheidsvaardigheden. Ik neem aan u heeft wel eens van die term heeft gehoord?

R: Ja.

I: En wat betekent het volgens u?

R: Dat mensen niet goed in staat zijn gezonde keuzes voor zichzelf te maken. Vaak vanuit misschien een niet weten. Sociale omstandigheden waar ongezond gedrag, leefwijze normaal is.

I: Oké. Ja. En hoe vaak heeft u te maken met patiënten met beperkte gezondheidsvaardigheden?

R: Ja, het ligt er een beetje aan wanneer, hoe beperkt iemand moet zijn maar ik denk, ik doe heel veel met hoofd-hals-kankerpatiënten.

I: Oké, volgens u, laten we uitgaan van uw definitie…

R: Ja.

I: Hoe vaak heeft u met?

R: Daar heb ik veel mee te maken.

I: Veel mee te maken?

R: Want ik doe veel pijnbehandelingen van mensen met een hoofd-hals tumoren. En daar zitten natuurlijk veel mensen bij die een uitgebreid rook c.q. alcohol verleden hebben.

I: Ah zo.

R: Ook veel mensen met een bijzonder levensverhaal zeg ik meestal.

I: Oké. Oké. En hoe herkent u die patiënten? Of is dat? Ja, dat heb ik denk ik eigenlijk al een beetje van u gehoord.

R: Ja.

I: Dat is een beetje de open houding en even kijken wat er komt, hè?

R: Ja.

I: Oké. Ja, mijn volgende vraag. Op welke manier probeert u rekening met hen te houden in uw mondelinge en/of schriftelijke communicatie?

R: Ja, ik denk, ik wilde zeggen, daar kan ik weinig nieuws over zeggen. Door het op een andere manier uit te leggen met makkelijkere woorden.

I: Ja, en ervaart u in de zorg aan deze patiënten ook barrières? Met deze groep, dus met deze mensen met beperkte gezondheidsvaardigheden, ervaart u daar ook barrières mee?

R: Niet in de spreekkamer. En dat verbaast mij zelf wel eens.

I: Oké.

R: Dat zijn mensen die je, waar je misschien op straat met een boog omheen zou lopen, of die je niet zou zien staan, maar in een spreekkamer is dat toch echt heel anders.

I: Oké.

R: Dan zijn die mensen, ja, het klinkt ook zo stom, volwaardiger of zoiets.

I: Oké. Ja. Ik zit even te denken, want ik, ja, dus u, in de spreekkamer zegt u van, nou, die mensen, ik noem het even, het is natuurlijk niet netjes maar, hè, dus de mensen volgens uw definitie met beperkte gezondheidsvaardigheden, die…

R: Dan zie ik die mensen staan, volledig.

I: Oké.

R: Als volledige mensen. Terwijl ze als ze op straat met een borrel op, dus als zwervers zouden lopen dan zou ik…

I: Dan loopt u er met een boog omheen.

R: Ja, dan is het volkomen anders.

I: Ja. Oké.

R: En dat vind ik, ja, dat vind ik gewoon zonde.

I: Maar waarom is dat dan? Of kunt u daar misschien iets over vertellen? Hoe komt dat dan?

R: Ja, dat weet ik niet goed hoe dat komt.

I: Oké. Want het verbaast uzelf geeft u aan…

R: Ja. Dan denk ik van, dan ben ik dus buiten te beperkt. Alsof je binnen, ja, opener staat voor ieder mens terwijl je buiten meer met je vooroordelen loopt en bij ons soort mensen blijft staan, zoiets.

I: Oké. Ja.

R: Ja.

I: Oké. Ik heb ten slotte nog één vraag.

R: Ja.

I: Ik heb hier een aantal kaartjes met daarop belangrijk thema's in de zorg voor patiënten in de palliatieve fase. Even kijken hoor. Deze ook nog. Die. En die. En kunt u met behulp van een rangordening in de kaartjes aangeven in hoeverre deze onderwerpen aan bod komen tijdens uw consulten?

R: Even kijken, in de rangorde, even kijken.

I: Ik, als u het goed vindt lees ik ze even hardop…

R: Ja, even kijken hoor, anders ga ik ze dadelijk…

I: Anders zet ik de band stop en dan weet ik niet meer wat er nou op tafel lag.

R: Oh ja, dan weet je niet meer wat, hoe en waarom. Nee, precies.

I: Daar kom je dan bij zo’n eerste interview achter, zeg maar.

R: Oh ja. Ja, precies. Oh ja, zij heeft met die kaartjes zitten spelen maar wat was het ook alweer? Wilsbekwaamheid. Goed, ik denk dat we beginnen met niet behandel afspraken, plaats van zorg en sterven, levensbeschouwing, wilsonbekwaamheid, ziekenhuis opnames, wettelijke vertegenwoordiging, crisis situaties.

I: Oké, dus van niet behandel afspraken tot aan crisis, dit komt eigenlijk altijd voor en dit bijna nooit in uw gesprekken met patiënten. Begrijp ik uw rangordeningen dan?

R: Ja, klopt. Klopt.

I: Oké.

R: Want ik zou niet weten waarom ik, dat is voor speciaal denk ik als iemand echt een kans heeft op een blow out, dan moet ik dat bespreken. Maar dat komt gelukkig heel weinig voor.

I: Oké.

R: En als je crisissituaties allemaal al gaat bespreken kun je ook heel veel angst veroorzaken, dus daar moet je even mee, echt voorzichtig mee zijn. Ja.

I: Oké. Ja. En worden bijvoorbeeld, als ik deze drie even een beetje isoleer, hè.

R: Ja.

I: Dus ziekenhuis opnames, wettelijke vertegenwoordiging en crisissituaties, mocht het niet ter sprake komen, worden die dan misschien door andere zorgverleners opgepakt bijvoorbeeld?

R: Ze liggen hier omdat ze bijna nooit voorkomen. Omdat ik ze meer, weet je wel?

I: Ja.

R: Wat ik net zei, een crisissituatie, ja, ik bedoel, meestal komt het niet voor.

I: Ja.

R: Of je zou moeten zeggen. Ja, ik vind ‘m lastig hoor.

I: Hoe bedoelt u?

R: Je kunt ook zeggen, heel veel mensen zijn bang om te stikken. En dat benoem ik dan weer wel. Is dat een crisissituatie? Want dat gaat namelijk niet gebeuren. Dus, ja.

I: Ja, die.

R: Ja, roept u maar wat dat is. En wettelijke vertegenwoordiging als, nou, dat bespreek ik eigenlijk niet van tevoren.

I: Ja, nee, maar oké, precies. Dus het komt gewoon weinig voor.

R: Ja.

I: Dat is het dan, ja.

R: En die ziekenhuisopnames, ik, als het goed is wordt dat met huisartsen besproken. Want dan krijg je de ellende in de weekenden. Eigen huisarts er niet, andere huisarts, er is een crisissituatie. Eigenlijk is er afgesproken, geen ziekenhuisopname. Maar ja, die tweede huisarts staat ook met zijn handen op de rug, dat is lastig. Dit vind ik iets voor in de eerstelijns te bespreken.

I: Oké. Dus die ligt aan die kant omdat het dan met de andere zorgverleners wordt opgepakt?

R: Ja, dat vind ik wel, ja.

I: Oké.

R: Ja? Dat was het?

I: Ja, dat is heel duidelijk. Ja, dat was ‘m, dank u wel.

R: Oké.

I: Heeft u nog wat toe te voegen aan het interview? Of heeft u nog wat te vragen? Dat kan natuurlijk ook het geval zijn.

R: Nee, ik geloof het niet.

I: Oké. Bedankt voor uw tijd!

Z2long01

I: interviewer

R: respondent

I: Ik wilde u graag wat algemene vragen stellen over uw rol als zorgverlener in de palliatieve zorg, over communicatie, over uw patiënten en ook misschien nog over de manier waarop het ziekenhuis georganiseerd is, maar dat moeten we even kijken of we daaraan toekomen. U vond de audio-opname goed. Heeft u tot zover vragen?

R: Nee.

I: Wat is uw rol in de zorg voor patiënten in de palliatieve fase?

R: Ja, in de palliatieve fase is met name begeleiden, comfortabel maken en ja, de juiste wegen te laten bewandelen de patiënten om aan zorg en ook zorgbehoefte te komen. Daar zit ik in de rol van een dokter dan in dit geval. Dan is het ook voor een deel medicamenteus, maar voor een deel is het met name de begeleiding en zorg eromheen. En dat proberen wij zo veel mogelijk in goeie banen te leiden. Zo moet je het zien.

I: Oké. Ja. Is er altijd één centrale zorgverlener aanwezig?

R: Ja, dat is eigenlijk de behandeld dokter, in dit geval ben ik. Ik coördineer, maar als we het over het palliatieve team hebben hè, dan zit ook de oncoloog, die zit erbij, pijnteam, en alles wat nodig, en een maatschappelijk medewerker en eventueel een psycholoog erbij. Het is gewoon een team eigenlijk voor de palliatieve zorg in het ziekenhuis, zo is ie geregeld. De dokter is, behandelend dokter met name, die is leading.

I: Oké.

En dat bent u ook?

R: Dat ben ik ook.

I: En is het duidelijk voor de patiënt wie dat?

R: Ja, tuurlijk ze hebben een contactpersoon dat is de behandelend dokter. En dat staat ook vast voor alle problemen ook op andere dingen dan komen ze bij ons terecht automatisch. En we hebben in het systeem ook wie eigenlijk aanspreekpunt is. Dat staat in een systeem. Dus al belt ie een andere afdeling, het aanspreekpunt staat gewoon in het systeem.

I: Oké, en u noemde het al het palliatieve team. Maar hoe werkt u binnen het ziekenhuis met de andere zorgverleners samen?

R: Wij hebben wekelijkse bijeenkomsten, dus voor de mensen die eigenlijk binnen het palliatief team werken en ook patiënten begeleiden. Hoe werkt het binnen het ziekenhuis? We hebben een onderling overleg een keer per week structureel. Als er dingen tussendoor zijn dan overleggen we. Zo is het eigenlijk in dit ziekenhuis geregeld. En ook als zij vragen hebben, zeg maar omgekeerd, dan kunnen ze dat ook bij ons vragen. De lijnen zijn kort.

I: Dus is dat dan een MDO?

R: Ja is een MDO een keer per week.

I: Oké. U noemde het palliatief team, daar bent u geen lid van bijvoorbeeld?

R: Jawel. Nee, kijk, als mijn patiënten besproken worden. Maar ik als dokter ben er alleen bij betrokken als mijn patiënten besproken worden, maar echte lid als dokter is de oncoloog. Dus ik ben eigenlijk een soort van, als mijn patiënten besproken worden dan zit ik ook in het palliatief, maar ik maak geen onderdeel uit van het palliatief team zoals jij bedoelt waarschijnlijk. Daar zit de oncoloog in, maatschappelijk medewerker, psycholoog en iemand van het pijnteam. Er zijn mensen die erin zitten dat is eigenlijk ons palliatief team. Maar als mijn patiënten besproken worden dan zit ik erbij als behandelend dokter.

I: Ja, oké, duidelijk. En hoe werkt u samen met de huisarts? Want we hebben het binnen het ziekenhuis wat besproken, maar…

R: Meestal is het met name als er dingen spelen dat de huisarts in ieder geval geïnformeerd wordt, dat is één. Dat doen wij meestal telefonisch alvast. En daarnaast worden eigenlijk ook de brieven naar de huisarts gestuurd van wat er afgesproken is, wat de stand van zaken is. En ik zit toevallig in het klankbordgroep van de huisarts, dus ja, ik heb juist kortere lijnen met de huisarts. Dus ik ken ze over het algemeen wel, maar het is met name dat wij ook, als we ze overdragen is dat naast dat er een brief naar de huisarts gaat dat wij ook even in ieder geval met de huisarts bellen om uit te leggen wat er aan de hand is, wat de status is en wat de afspraken zijn, want uiteindelijk nemen zij de begeleiding over. Daar is altijd telefonisch overleg vooraf. Dus kan per brief, maar nadat wij die patiënt gesproken hebben, het traject ingaan, bellen we met de huisarts en leggen we alles uit.

I: Oké, dus je belt altijd nog even na.

R: Ja, voorafgaand aan overdracht met name. Maar wij hebben altijd telefonisch contact.

I: En met bijvoorbeeld maatschappelijk werk?

R: Maatschappelijk werk, die wordt eigenlijk, als die, als palliatief ingeschaald wordt, komt ie vanzelf in beeld.

I: Oh die zit eigenlijk altijd binnen dat palliatieve team?

R: Ja, daarom. Dat gaat automatisch hier. Dan neemt de maatschappelijk werker het over en die gaat op zijn of haar gebied evaluatie doen en anders een begeleider. Dat gaat automatisch bij ons. Daar hoef ik niet eens voor te bellen.

I: Oké. Heeft u nog iets toe te voegen aan uw rol als zorgverlener in de palliatieve zorg?

R: Nee.

I: Communiceren, informeren en de gezamenlijke besluitvorming. Binnen de palliatieve zorg is aandacht van belang voor meerdere aspecten, het fysieke, psychische, sociale en spirituele welzijn van de patiënt. In hoeverre komen deze verschillende aspecten aan bod in gesprekken met uw patiënten?

R: Ja dat is meer van, je schat het een beetje in, van dat fysieke dat is dokter zijnde en ontdekken we wel, dat zien we wel. Dat psychische is met name wat ik zei ook hè als het in de palliatieve teamsfeer is, ja dan komt ie dus bij de medische psycholoog terecht. En dat sociale gedeelte, daar laat ik de maatschappelijk medewerker over het algemeen eigenlijk over gaan. En dat spirituele, dat is wel iets wat nieuw is voor ons met name. Ik doe ook onderzoek op het gebied van kwaliteit van leven, is een project wat ik ook met Japan doe. Dat is over kwaliteit van leven. Om te kijken van ja, hoe de beleving bij Aziatische mensen en Europese mensen is. Maar het spirituele welzijn, dat komt nu ook aan bod, maar dat is alleen in onderzoek verband.

I: Oké, maar in uw kamer, in uw consultkamer?

R: Dat gebeurt niet, nee. Maar misschien in studieverband dat er nu wel iets mee gaat gebeuren, maar de resultaten moeten nog komen. Van een beperkt aantal mensen heb ik wel al wat terug, maar voor de rest doe ik er niet veel mee, nee.

I: Oké. Dus wat u zegt met name het fysieke, dokter zijn, psychische en dan wordt het eigenlijk minder.

R: Ja.

I: Oké. En u had het over het inschatten. Je maakt eigenlijk een inschatting en dan begin je. Ik herhaal nu even wat u zegt hè, als ik iets verkeerds zeg moet u meteen verbeteren. Maar u had het over inschatten, hoe werkt dat dan? Kunt u daar iets meer over vertellen?

R: Nee maar dat is het fysieke, met name lichamelijke. Wat kan een patiënt, hoe ziet ie eruit, is ie vermoeid, kan ie nog lopen, hoe komt ie binnen. Dus dat soort zaken bedoel ik met het fysieke. Kijk als een patiënt, ja, op een brancard komt ja dan weet je van ja hoe ver die is en wat je opties zijn. Daar gelden allerlei regels voor. Dan kan je ook een beetje inschatten van wat is de behoefte, waar moet je aan denken en wat kan ik voor die patiënt gaan organiseren op basis van wat ik zie. Dus vandaar.

I: En maakt u bijvoorbeeld ook een inschatting op communicatie of een manier waarop u dan moet communiceren?

R: Nou over het algemeen, qua communicatie heb je, ja de populatie die ik over het algemeen zie is daar geen beperking of allerlei dingen die gebeuren. Ja, de communicatie, ik pas mijn communicatie niet aan aan de fysieke gesteldheid om het maar zo te noemen. Misschien wel de psychische gesteldheid, maar dan nog is het een beetje altijd inschatten van ja hoe ga je het gesprek in. Dus ja dat blijft een beetje lastig inschatten eerlijk gezegd. Maar over het algemeen is het ja, qua communicatie valt er niet veel te veranderen of aan te passen over het algemeen.

I: Oké. Dus u past eigenlijk uw communicatie niet of nauwelijks aan.

R: Nee hangt ervan af. Ja, communicatie in de zin van als het echt om communicatie gaat, ja, wat moet je aanpassen? Het is en blijft hetzelfde niveau over het algemeen. Tenminste dat is mijn inschatting. Dus ik weet niet waarom ik dat moet aanpassen als ik een patiënt heb die anders is.

I: Wat bedoelt u daarmee het is hetzelfde niveau?

R: Met communicatie van ja hoe richt je je communicatie en hoe communiceer je naar de patiënt toe. Dat is onafhankelijk van de fysieke gesteldheid over het algemeen toch?

I: Ja, dat is waar. Maar los van die fysieke gesteldheid, denk bijvoorbeeld aan patiënten die laagopgeleid zijn of niet zo heel veel lijken te snappen?

R: Ooh ja, nee tuurlijk, als je dat mee wilt nemen. Nee tuurlijk. Die inschatting moet je zeker maken. Als het een laaggeletterde is of iemand op een ander niveau werkt qua opleiding, naja dan ga je ook een keer je gesprek op aanpassen. Je moet gewoon begrijpelijk zijn en ook voor de patiënt te snappen zijn. Dan ga je daar ook niet op een hoger niveau communiceren. Dan pakt ie niks mee. Ja, tuurlijk je past je wel aan aan het opleidingsniveau. Ik bedoel er meer mee te zeggen qua, ik heb in de periferie gewerkt, daar moest je je communicatie wel aanpassen omdat mensen met taalbarrière of meerdere dingen, maar ook in deze populatie zie je mensen die ja, wij noemen het wat simpeler zijn dan doorsnee. Ja daar hou je wel rekening mee in je communicatie uiteraard en dan pas je je communicatie wel aan, dat zeker. Even voor de duidelijkheid.

I: Ja, ja precies. En hoe past u die communicatie dan aan?

R: Ja, je probeert in simpele woorden uit te leggen en ook even nog eens te verifiëren of het doorkomt. En dan stel je een vraag van wat begrijp je en of hij of zij het snapt wat er gezegd wordt en wat de consequenties zijn. Zo doe je dat, je controleert het telkens.

I: En bij dat, ik noem het even de simpelere patiënten. Komen daar eigenlijk diezelfde aspecten ook ter sprake? Dus sowieso het fysieke natuurlijk, maar

R: Ja, zeker. Het zijn dezelfde aspecten alleen in andere woorden, waarbij je jezelf een beetje aangepast aan het niveau van de patiënt. In dit geval intelligentieniveau dan.

I: Ja, precies. U gaf aan, je maakt die inschatting in het begin van het consult. Werkt dat bij die patiënten hetzelfde?

R: Nee, maar in een gesprek is ook altijd ook bij intellectuele patiënten, probeer je ook te controleren van ja, wat komt er door wat ik uitleg. En ik probeer ook in simpele woorden uit te leggen. Dat doe je bij elke patiënt eerlijk gezegd, om te kijken of het begrepen wordt. Dus ja, heeft niks met die fysieke gesteldheid. Ja als je een gesprek voert kom je er vanzelf achter wat het niveau van zo iemand is. En wat voor patiënt het ook is, je controleert wel telkens of het begrepen wordt en of de boodschap doorkomt. Dat kunnen twee dingen zijn. Dat kan ontkenning zijn of het kan ook te maken met het intelligentieniveau van de patiënt. Probeer het ook dan in simpele woorden uit te leggen. Zo gaat zo'n gesprek eigenlijk.

I: Dus eigenlijk gaandeweg het gesprek, als ik u mag herhalen, dan komt er eigenlijk, dan maakt u eigenlijk een inschatting van oké. En hoe controleert u of patiënten het hebben begrepen?

R: Na zo'n gesprek vraag ik ook of het begrepen is en of ze in simpele woorden kunnen uitleggen wat er verteld is. Dat is een manier om het te doen. Dat doe ik met name bij patiënten waar ik ook echt de indruk heb dat ze het niet snappen. En ik probeer ook in ieder geval te herhalen van wat ik zeg en ook nog eens te vragen of het begrepen is. En soms laat ik ze ook zelf vertellen of wat ik verteld heb of het doorgekomen is en wat zij ervan vastgehouden hebben. Zo controleer je, ja, zo hoeft niet bij iedere patiënt gelukkig, maar ja bij bepaalde patiënten doe je dat wel. Het is niet de doorsnee patiënt.

I: Die vier aspecten waar we het net over hadden, worden die ook vastgelegd in een individueel zorgplan?

R: Het is je dossiervoering en die is er sowieso. Ja, dan zie je ze met name en dat kan je er ook eventueel erin gooien, dat je de inschatting hebt dat iemand het moeilijk heeft, depressief is of wat dan ook, ja dat zet je wel erin, maar niet echt een zorgplan uitkomen.

I: Want werkt u met een individueel zorgplan?

R: Nee.

I: Oké.

R: Ja, maar individueel zorgplan, wat bedoel je ermee? Wij zien de patiënten, we zijn de artsen, wij zien de patiënten en het is afgemeten zorg, uiteraard, als je er dat mee bedoelt. Maar daarin worden die aspecten in meegenomen, maarja, kijk, als ik een inschatting heb van hier is iemand die psychische begeleiding nodig heeft, ja, dan verwijs ik die ook door. Dat soort dingen worden er wel in meegenomen.

I: Ja, precies. Wat ik bedoel is die aspecten, of je die dan ook na een consult ook vastlegt in een systeem of noteert.

R: In het dossier wel, wat je bevindingen zijn, dus dat doe je zeker. Maar het is niet dat we een structureel format hebben om dit allemaal te doen, dat doe je gewoon als dokter na je bevindingen die notities maken. Het is niet een format waarin je dit allemaal stopt. Dus dat doe je wel in je dossier en in je consultvoering.

I: Oké. Goede communicatie is de basis voor goede zorg zodat de patiënt goed geïnformeerd is dat er sprake kan zijn van gezamenlijke besluitvorming. Zijn er patiënten bij wie u de communicatie moeilijker vindt?

R: Ja, maar de moeilijkheidsgraad van de communicatie, waar ligt dat aan? Kijk je hebt soms patiënten waarbij, is de communicatie dan moeilijk als je het voor de communicatie doet? Nee maar kijk, als je iemand vertelt in ons vak dat het er allemaal slecht uitziet bij wijze van, op basis van de bevindingen, stel dat je een patiënt hebt waar je geen behandelopties hebt, maar die patiënt die volhard zich in dat ie in ieder geval een behandeling wil en ook niet wil snappen waarom de behandeling niet kan, ja dat vind ik wel lastige communicatie in de zin van, al probeer je het honderd keer uit te leggen, als ze dan niet proberen te snappen van ja, dat dit de regels zijn en dat er geen opties zijn. In dat geval zou ik zeggen, dat is wel iets wat lastig is. Maar wat je dan doet is alle informatie geven en een gesprek weer inplannen en nadien dat de patiënt er in ieder geval over kan nadenken en ook een beetje met de familie erover kan hebben en dan eens terugkomen om verder afspraken te maken. Dat is een categorie waarbij de communicatie over het algemeen stroef verloopt. Lastige communicatie ja, die bestaat eigenlijk niet zou ik zeggen.

I: Oké. Ja. Want mijn volgende vraag zou zijn, in welke opzicht is de communicatie dan moeilijker. Maar dat is dus als ik u goed begrijp, is dat dus eigenlijk om mensen te overtuigen van iets.

R: Ja of als er een ontkenningscomponent in zit, dan heb je een probleem. Dat zie je weleens, maar niet vaak gelukkig.

I: En wat u dus dan doet om de communicatie te verbeteren, is niet zozeer de communicatie zelf gaf u aan, maar dat u dan…

R: Je probeert dan wel in simpele woorden het dan nog eens uit te leggen.

I: Ja, precies dat u het dan herhaalt en ook op schrift meegeeft en al dat soort dingen, klopt dat?

R: Dat klopt. Dan geef je dat mee en dat ze erover kunnen gaan nadenken en dat we het daar dan weer over moeten hebben. Dus ja dat is een manier die ik eigenlijk toepas, weer een gesprek te voeren, in te plannen. Oké je kan die gesprekken oneindig maken terwijl het niet doorkomt, ja dan heb je een probleem.

I: Ja. En u gaf ook aan dat er eigenlijk geen communicatie is die lastig is. Kunt u daar iets meer over vertellen?

R: Het is met name, in dit geval is het voor ons namelijk een boodschap overbrengen. Ja hoe je het doet, of je links of rechts, maar je moet eigenlijk de boodschap proberen over te brengen. Ik heb tot nu toe geen patiënt waarbij het niet lukt om het maar zo te noemen. Soms doe je het in simpele woorden, soms doe je met een tweede gesprek of wat dan ook, maar je komt altijd uit. Dus ik zie er het probleem niet van in. Wat is de moeilijkheidsgraad van communicatie, dat is eigenlijk de tijd gunnen, herhalen en proberen in simpele woorden uit te leggen.

I: Ja, oké. Mijn volgende vraag heeft u eigenlijk ook al beantwoord denk ik. Zijn er momenten dat u bewust uw communicatie aanpast en hoe dan? Kunt u misschien een voorbeeld geven van wanneer u dat bijvoorbeeld gedaan heeft of moest doen?

R: Ja dat is eigenlijk wat ik net ook vertelde, van de communicatie aanpassen dat is het niet zozeer. Wat ik net zei, laten we nemen die simpele patiënt die een laag intelligentieniveau heeft, ja dan pas je jezelf daaraan, dan ga je proberen in simpele woorden uit te leggen. Dat is je aanpassing wat je doet hè. Kijk als een boodschap niet doorkomt, ja, dan probeer je nog eens een gesprek te voeren en, je, kan ook evengoed zijn dat ook met iemand erbij, familie die ze dan meenemen of kennissen of wat dan ook, dat je dat doet. Aanpassen is met name aanpassen aan het niveau van de patiënt. Dat is wat je doet. Maar het is niet dat je allerlei andere methoden gaat toepassen, dat doe ik niet. Het is en blijft communicatie die je naar elkaar toe hebt. Het kan ook evengoed zijn dat de patiënt dan volgende keer wel met iemand komt waarmee je dan ook de zaken bespreekt. Dus dat kan ook. Het is een optie. Taalbarrièremensen gebeuren dat ook sowieso dat ze dan met iemand moeten komen als je merkt dat het niet doorkomt of niet begrepen wordt. En dan vraag je of ze dan iemand meenemen.

I: Oké. Even denken, gebruikt u wel eens filmpjes of ander visueel voorlichtingsmateriaal in de communicatie?

R: Ja, dat zijn die informatiematerialen. Dat is met name papier, folders over het algemeen. We hebben een tijdje gebruikgemaakt van zo'n folder, zo'n videoboekvorm. Ik heb ‘m weggedaan, wat het was een pilot. Het is zo'n boek, maar het is een soort van video.

I: U vond het niet zo?

R: Nee, jawel, maar dat komt nog, dat komt wel. Dat was nog pilot.

I: Oh dat moet nog ontwikkeld worden.

R: Ja, dat moet nog ontwikkeld worden dat de mensen dat kunnen afluisteren. Is een soort van videogebeuren, is een communicatiemiddel. Informatie op video, maar dan kunnen patiënten ook zelf kiezen wat ze willen, welke vraag, welk antwoord.

I: Oké. In hoeverre is er volgens u sprake van gezamenlijke besluitvorming met patiënten in de palliatieve fase?

R: Kijk, de palliatieve fase is met name, het is behandelen of niet behandelen over het algemeen. Dat zijn twee keuzes die je voorgelegd worden. Er valt altijd een keuze te maken. En ik zeg ook, met name ja en nee en beiden zijn goed, maar het is met name dat je gezamenlijk moet kijken, van ja, wat is voor de patiënt het beste in dit geval en wat kan je sowieso. Kijk, als je niks kan, dan is het ook klaar. Ik bedoel, dan moet je ook proberen uit te leggen dat er geen mogelijkheden zijn. Als er mogelijkheden zijn dan heeft de patiënt eigenlijk de meeste zeggenschap van wat wilt ie. Dus uiteindelijk is eigenlijk het besluit aan de patiënt met ondersteuning, met medische onderbouwing om eigenlijk een besluit te nemen. Het is aan de patiënt om een besluit te nemen wat ie wil, over het algemeen. Als dokter kan ik van alles willen, maar als de patiënt niet wil, dan hoef ik dat niet te geven, dat is één, en omgekeerd kan het best zijn dat de patiënt alles wil, maar medisch gewoon niet kan. Het kan alle kanten op.

I: Bedoelt u dan met over het algemeen dus dat het eigenlijk altijd twee kanten is?

R: Ja het is altijd een gezamenlijk besluit, maar besluit van patiënt is met name doorslaggevend. Uiteindelijk is en blijft de patiënt de baas, vind ik.

I: Ja. En is dit voor alle patiënten hetzelfde?

R: Ja, is ook voor de andere patiëntencategorieën, is niet alleen voor de palliatieve vorm. Je probeert met je medische kennis een boodschap over te brengen en hopen dat het dan doorkomt en dat controleer je dan ook en op basis daarvan moet een patiënt proberen een besluit te nemen. En dat geldt voor elke patiëntencategorie over het algemeen. Ik kan niet zeggen ik wil dit of dat doen. De patiënt is eigenlijk de baas, maar je moet het wel kunnen onderbouwen. En soms wil de patiënt te veel en kan je ook niet veel, dan moet je ook proberen uit te leggen waarom dat niet kan. Je kan beide kanten op redeneren.

I: Oké. En denkt u dat het beter zou zijn als er meer gezamenlijke besluitvorming plaats zou vinden?

R: Ja, maar over het algemeen is het al gezamenlijke besluitvorming, toch? Zeg ik. De patiënt is met name die eigenlijk, ja kijk, gezamenlijke besluitvorming is met name als je meerdere keuzes hebt. Wij proberen die keuzes, in ieder geval bepaalde keuzes te onderbouwen, van dit en dat kan je, maar de patiënt zal op basis van de gegevens of de informatie die hij van jou heeft een besluit moeten nemen. Ja het besluit is uiteindelijk van de patiënt, maar met de informatie die je gegeven hebt. Dan is het wel gezamenlijk voor een stukje, maar voor een stukje is eigenlijk de patiënt die het bepaalt, vind ik.

I: Ja, oké. Wat u vindt is duidelijk. Maar vindt u ook dat het genoeg gebeurt?

R: Over het algemeen denk ik wel. Ik denk dat het wel gebeurt, bij iedere patiënt denk ik. Zo verloopt de communicatie, zo verloopt ook de besluitvorming, bij elke willekeurige arts.

I: Ja. Kunt u een voorbeeld geven van een situatie waarin er duidelijk sprake was van gezamenlijke besluitvorming?

R: Kijk, je hebt een patiënt die is wel wat oud, maar wel best vitaal, maar aan de andere kant heeft ie wel meerdere afwijkingen waarvan je denkt van, oké je kan twee dingen doen, of een grote operatie aan beide kanten met een verhoogd risico, of je kan kiezen voor een alternatief, van zullen we dit niet stereotactisch bestralen. Maar ja, beide opties die zijn er, beide opties zijn met mitsen en maren van die stereotactische bestraling, ja we hebben geen grote data en dan weet je de uitkomst niet, maar is wel een goed alternatief. Van die operatie weet je dat het risico’s inhoudt en ja bij grote risico's, ja, dan moet je de risico's voor- en nadelen afwegen en met elkaar bespreken van ja, dit zijn de opties en waar gaat zo'n beetje de voorkeur naar. In principe leid je erin, van dit zijn de opties, van de ene is het risico groter dan van die andere, maar het is wel een gezamenlijke besluitvorming in de zin van dat je gezamenlijk besluit wat eigenlijk het beste voor je is, maar nogmaals, het is en blijft een keuze van de patiënt, vind ik. Maar je moet wel even onderbouwen waarom je die ene een beter alternatief vindt dan die andere. Zo werkt het in de praktijk over het algemeen.

I: Ja, oké. Deze is dan denk ik lastiger, maar heeft u misschien ook een voorbeeld van een situatie waarin het juist niet het geval was?

R: Ik beslis nooit voor de patiënt.

I: Nee oké, dus eigenlijk niet.

R: Nee, geen situatie, de patiënt is de baas. Ik besluit niks voor de patiënt. De patiënt besluit uiteindelijk welke alternatieven hij neemt. Dus als er twee keuzes zijn of meerdere, dan is het toch aan de patiënt. Ik heb niks in te brengen, over het algemeen niet. Als dokter moet je niet iets gaan opleggen of iets willen doen wat jou het beste lijkt en wat de patiënt niet wil. Dat ga je niet doen, dat is niet van deze tijd.

I: Nee, precies, oké. Dus als u vindt dat het beter zou kunnen, dat gezamenlijk besluitvormen, ik weet niet of dat zo is, maar stel, u denkt dat zou misschien nog wel beter kunnen. Wat zou er dan voor nodig zijn denkt u? Ik moet hem misschien anders stellen. Denkt u dat het beter zou kunnen, die besluitvorming?

R: Ik denk dat je tot nu toe, wat ik net zei, je geeft die informatie, je bent als dokter een neutraal iemand en met medische kennis leg je bepaalde dingen voor. In dat kader moet eigenlijk de patiënt een besluit nemen, ja, wat valt daar te verbeteren? Ik denk niet veel, want je doet het nu al, van dat je alle relevante informatie aan de patiënt geeft en uiteindelijk de patiënt het voorlegt en de patiënt de keuze laat maken over het algemeen. Het stukje wat gezamenlijk is, is met name de informatievoorziening die je doet. Dat is het belangrijkste. Ja, dat doe je gezamenlijk. Maar ja, besluitvorming is aan de patiënt. Tenminste, zo zie ik dat nog steeds voor me. Is toch een beetje lastig om te zeggen van ja, wat valt er te verbeteren? Wat je moet doen eigenlijk is de informatievoorziening en de patiënt die besluit daarna wat ie wil.

I: Ja, die is heel helder, oké. Tot dusverre gezamenlijke besluitvorming. Ik heb hier een aantal kaartjes. Even kijken. Wettelijke vertegenwoordiging in de situatie van acute verslechtering, crisissituaties, wilsonbekwaamheid en levenseindebeslissingen, plaats van zorgen en sterven, levensbeschouwing en culturele achtergrond en niet-behandelafspraken. Kunt u met behulp van een rangordening in de kaartjes aangeven in hoeverre deze onderwerpen aan bod komen tijdens uw consulten?

R: Nou, in ieder geval, omdat ik oncologische dokter ben dan komt de niet-behandelafspraken met name over reanimeren en dergelijke altijd aan bod bij nieuwe patiënten. Als je een stadium 4 hebt, dan moet je ook die discussie niet gaan voeren als het slechter wordt van hé, we gaan niks doen. Je weet je kan van alles willen, je kan geen enkel intensivist in een ziekenhuis vinden die een patiënt gaat reanimeren die uitgezaaide ziekten heeft. Dus dat moet je wel even afspreken in ieder geval. Kijk als ie in de situatie komt in het ziekenhuis, dat er in ieder geval een zinloze ingreep is, als je iemand gaat reanimeren of beademen en dat het om die reden niet gedaan wordt, dus ja, over niet-behandelafspraken dat komt wel aan bod bij de nieuwe patiënt die eigenlijk de diagnose longkanker heeft in uitgezaaide vorm. En dan zal je dat wel bespreken. Ziekenhuisopnames, dat staat ook daaraan gekoppeld hè. Van ja, hoe en wat en wat er tijdens ziekenhuisopname gebeurt. Kijk, als een patiënt terminaal is, ja, dan is het over het algemeen in thuissituaties, ja dan is er eigenlijk een geen-returnbeleid. De huisartsen over het algemeen die kunnen het goed begeleiden. Ziekenhuisopname dat is daarbij niet noodzakelijk meer, dat was vroeger. Dit was het, plaats van zorg en sterven, ja, ik leg ze altijd uit, van dat er tegenwoordig heel veel mogelijkheden zijn. Hospice, dat is nog altijd iets dat kan. En dat er tegenwoordig ook een heleboel dingen in de thuissituatie kunnen met alle zorg eromheen en onder begeleiding van de huisarts. Ik heb er tot nu toe goeie ervaringen mee. We spreken met de huisarts en kijken wat de opties zijn en of ie ermee ingaat. Dan zijn we, plaats van zorg en sterven, dus dat zijn twee opties. Wij zijn poliklinisch, en als het vanuit de kliniek gebeurt, ja dan gaat het van hieruit ingezet worden, dan regelen wij dat vanuit de afdeling. Maar meestal zijn de patiënten in de thuissituatie en dan gaat dat via de huisarts. Maar je benadrukt wel dat er eigenlijk genoeg mogelijkheden zijn in de thuissituatie of vanuit een hospice. Dat is dan wel besproken. Ja, soms wilsonbekwaamheid daar hebben we bijna nooit mee te maken eerlijk gezegd. Maar voor levenseindebeslissing en dergelijke meestal, zie je wel, dan adviseren wij wel, van ja, de wensen om dat ook met de huisarts te bespreken. Dat benadrukken wij wel. De crisissituatie heb ik gelukkig niks mee te maken over het algemeen. Dus ik weet niet wat ik daar iets over moet bespreken. Ja over het vertegenwoordigen van acute situaties, kijk, over het algemeen staat het wel vast wat de patiënt wil en wat de wens is. En meestal zie je met name oncologische patiënten dat het goed gestructureerd is en dat ze dat in ieder geval besproken hebben met de huisarts bijvoorbeeld. Dus dat komt bij mij niet al te veel aan bod. Levensbeschouwing en culturele achtergrond, gelukkig hebben we qua culturele achtergrond, heb ik maar een paar patiënten met een andere culturele achtergrond. Wat de levensbeschouwing betreft, ja, dat is ook aan hun om dat in te vullen. Ik heb niet een populatie, ja, een paar patiënten met een andere culturele achtergrond. Dat is voor mij nu niet actueel. In het vorige ziekenhuis wel, toen was meer dan vijftig procent van een andere culturele achtergrond.

I: Oké. Zoals u in het begin ook al aangaf, een belangrijke rol voor de huisarts ook weer eigenlijk…

R: Die wordt ook altijd besproken ja.

I: Heel veel facetten.

R: Ja.

I: Oké. Laatste hoofdstuk, dat gaat over beperkte gezondheidsvaardigheden. Heeft u wel eens gehoord van het begrip beperkte gezondheidsvaardigheden?

R: Wat bedoel je ermee?

I: Dat zou eigenlijk meteen mijn vraag aan u zijn. Wat betekent het denkt u? Beperkte gezondheidsvaardigheden.

R: Is dat voor de patiënt?

I: Voor de patiënt ja. Dus een patiënt met beperkte gezondheidsvaardigheden, wat zou dat volgens u zijn?

R: Ja maar wat is gezondheidsvaardigheden, ja, in welke context zet je dat? Ik zou niks kunnen verzinnen.

I: Oké, nou, de definitie zoals wij hem nu met dit project hanteren is de vaardigheid van mensen om informatie over gezondheid te krijgen, te begrijpen en toe te passen en dat is zowel functioneel, dus dan heb je het echt over lezen en schrijven, interactief of communicatief, dus begrijpend lezen, hoofd- van bijzaken scheiden, vragen stellen en kritisch, dus het toepassen van informatie, ordenen, vooruitdenken, nou, dat soort dingen eigenlijk. Dus mensen met beperkte gezondheidsvaardigheden die zijn hierin beperkt, zal ik maar zeggen. Hoe vaak heeft u te maken met patiënten met beperkte gezondheidsvaardigheden?

R: Ja maar kijk, die informatievoorziening die krijgen ze sowieso hè. Dan kan je ook een ontkenningsfactor erbij hebben. Ja, dat begrijpen dat proberen we uit het gesprek zo veel mogelijk eruit te halen, te toetsen van ja, wordt het begrepen. Maar je hebt ook een stukje toe te passen wat je zegt. Je hebt ook een stukje die eigenlijk in de ontkenning zit en het niet wil begrijpen of niet naar willen leven. Dus laten we even kijken, oké, oncologie daar valt weinig te beleven, en ja toe te passen dat kunnen ze ook doen. Maar ja, bijvoorbeeld bij COPD, bij wijze van, ja, om die levensstijl aan te passen, ja, dat is eigenlijk begrijpen en toepassen, van ja wat kan ik er zelf aan doen en wat is mijn inbreng voor mijn gezondheid.

I: Oké. En hoe herkent u zo'n patiënten?

R: Ja, je merkt dat het telkens misgaat. Met name dat er een bepaalde factor in meespeelt. Dat merk je wel, dat haal je ook eruit. Je gaat je altijd afvragen, waarom gaat het mis, of als je opmerkt dat de patiënt in ieder geval in de ontkenningsfase zit, dat haal je ook eruit. Dan komt de boodschap niet door en ja, de dingen die je bespreekt, maar uit het gesprek kom je wel achter, van dat het niet doordringt of wat dan ook. Dat je dat over het algemeen wel opmerkt.

I: Oké. En bijvoorbeeld bij zo'n COPD-patiënt en dan met lifestyle, hoe gaat dat dan, of waar gaat het dan mis?

R: Bijvoorbeeld, ja, kijk, bij COPD, daar heb je voor een overgroot deel een eigen verantwoordelijkheid. Medicijn is niet de beste behandeling, stoppen met roken is de beste behandeling. Dat bespreek je en probeer je uit te leggen, maar die verantwoordelijkheid ligt wel bij de patiënt. En de vraag is van ja, dringt het door en snapt de patiënt de ernst ervan en gaat ie er iets mee doen. Dat kan je ook vragen bij het volgende gesprek van ja, hoe is het met roken. Heb je hulp nodig, dat kunnen we aanbieden, sinds kort weer, ook bij de huisarts.

I: Ja. Oké. En als u dan met zo'n, ik noem even een voorbeeld hè, maar u heeft dus die COPD-patiënt die nog steeds rookt, houdt u daar dan rekening mee in uw communicatie? Als u er bijvoorbeeld achter komt dat zo iemand nog steeds rookt?

R: Nee, de verantwoordelijkheid is aan de patiënt vind ik. Ik probeer telkens te benadrukken wat het nut ervan is. Ik bind er geen consequenties aan, het is een eigen verantwoordelijkheid. En ik leg ze ook uit dat het slechter kan gaan en dat dit de opties zijn, maar op een gegeven moment is er een niet omkeerbare situatie. Dan heb ik daar niks in te brengen, maar ik probeer ze wel bewust te maken. Maar ik ga mijn beleid niet aanpassen om te zeggen van oké, als je niet wil, dan houdt het hier op, ja, dan ben ik als dokter niet goed bezig. Ik probeer toch de patiënt zo veel mogelijk te begeleiden en in te begeleiden en te behandelen. Maar de behandeling moet je er niet van laten afhangen, maar je moet wel benadrukken naar de patiënt voor een stukje toch eigen verantwoordelijkheid heeft.

I: Oké, ja. Ervaart u barrières in de zorg aan patiënten met beperkte vaardigheden?

R: Nee. Ja, wat zou een barrière moeten zijn?

I: Ja, want hier kwamen we ook op bij het communiceren hè, of u barrières ervaart. Toen gaf u eigenlijk al vrij vlug aan van ja, communiceren daar heb ik eigenlijk geen barrières in.

R: Ja. Het heeft ook te maken met de patiëntenpopulatie die je hebt denk ik. Hier gaat het over het algemeen wel prima. Kijk, wat ik zei, als je zit in een ander ziekenhuis, in de periferie waar ik ook ooit gewerkt heb, ja, dan ervaar je wel barrières in de zin van de communicatie dat dingen niet doorkomen of ja, niet begrepen worden of wat dan ook, maar hier gelukkig niet, in de huidige situatie.

I: Oké. Dus u ervaart het eigenlijk niet zo, maar u heeft bijvoorbeeld ook geen, dus wat heeft u nodig om de zorg beter aan te laten sluiten op dat soort patiënten? U geeft eigenlijk aan van, ik heb niet echt barrières, maar zou er iets kunnen zijn waardoor de zorg beter aan zou kunnen sluiten bij specifiek die patiëntengroep?

R: Ja, wat heb je aan tools als dingen niet begrepen worden, dan moet je proberen toch meerdere malen gesprekken te voeren en dingen uit te leggen. Wat zou de zorg verbeteren? Ik kan het niet verzinnen eerlijk gezegd. Ik weet niet of je daarvoor tools hebt, ik denk het niet. Ik weet het niet. Nee, ik zou het niet kunnen zeggen.

I: Heeft u verder nog aanvullingen of vragen over het interview?

R: Nee. Het is een beetje lastig als je specifiek op de definities gaat letten en noem maar op, in de dagelijkse praktijk sta je er niet bij stil, denk ik.

I: En bedoelt u dan in bijvoorbeeld consultgesprekken?

R: Maar ook het beperkte gebeuren.

I: Bedoelt u beperkte gezondheidsvaardigheden?

R: Ja. Daar sta ik niet bij stil. Als je hem nu zo stelt, dan komt het wel aan bod, hoe dan ook, maar ja, niet in die vorm van dat je er gestructureerd mee bezig bent, dat weer niet.

I: Oké. Nou dank u wel.

Z2long02

I= interviewer

R= respondent

I: Dankjewel. Even kijken hoor. Het eerste thema gaat over jouw rol als zorgverlener in de palliatieve zorg aan patiënten. En wat is jouw rol in de zorg voor patiënten in de palliatieve fase? Oh, was trouwens alles duidelijk verder?

R: Ja hoor. Wat is mijn rol? Ik denk dat mensen die echt in de palliatieve fase zitten, in de zin van die echt in de stervensfase zitten, is onze rol heel klein eigenlijk. Die verwijzen wij vrijwel altijd terug naar de huisarts. Maar er zijn natuurlijk ook mensen die in een palliatieve fase zitten die nog actief behandeld worden. En daarin is onze rol met name die van, ja, behandelaar aan de ene kant maar aan de andere kant een soort van regievoerder ook. Hè, zorgen dat mensen met hun klachten op de juiste plek komen, bij de juiste behandelaar. En met name ook op tijd signaleren van problemen. Ja, dus regievoerder en een, hè, wat betreft klachten een signaalfunctie.

I: Oké. Dus zijn jullie voor patiënten meestal de centrale zorgverlener?

R: Ja. Zo lang zij bij ons echt actief onder behandeling zijn wel, ja.

I: Oké. En het is voor de patiënt ook altijd duidelijk wie dat dan ook is?

R: Vast niet.

I: Oh, vast niet?

R: Vast niet, nee.

I: Oké.

R: Nee.

I: Die vraag was eigenlijk een inkoppertje dacht ik, maar…

R: Ik zeg het wel, vaak tegen mensen, hè, van, nou, je mag altijd bellen. En bel dan naar poli en vraag dan naar mij. Dat gaat eigenlijk altijd wel goed. Of naar de verpleegkundige specialist van ons, van oncologie.

I: Ja.

R: Maar je merkt dat met name in diensttijd, dat mensen toch vaak niet weten bij welk loket zij terecht kunnen, ondanks dat dat wel in allerlei folders staat en dat het vaak wel besproken is maar dat blijft, zeker in een panieksituatie, dus in een situatie dat er iets gebeurt, blijft dat kennelijk onduidelijk.

I: Oké. Maar wat gebeurt er dan meestal?

R: Nou, dan bellen ze 1-1-2.

I: 1-1-2 En dan ziekenhuis en dan komen ze op de? Oh ja.

R: Ja, in het ziekenhuis. En dan worden zij ingeschreven voor de chirurg. En die ziet dan een gebroken been maar die kijkt niet naar het hele plaatje en dan, nou ja, dan gebeuren er, kunnen er allerlei hele rare dingen gebeuren. Terwijl dat, hè, als dat, als er direct naar ons gebeld was, was dat veel beter gestroomlijnd geweest. En dan hadden wij misschien gewoon de huisarts gestuurd.

I: Ja.

R: Of wij hadden pijnstillers voorgeschreven. Dus dat is, nee, ik denk niet dat dat dit altijd even duidelijk is, nee.

I: Oké, dus ik ga even voor mijzelf samenvatten. Jij moet mij onmiddellijk verbeteren als ik iets verkeerds zeg. Dus jij zei, dus behalve als een soort van regievoerder en hoofdbehandelaar heb je ook een soort, zeg je, een signalerende rol die heel belangrijk is. Ja, dus jij bent vaak ook centrale zorgverlener. En voor de patiënt is het niet altijd, meestal niet, duidelijk wie dat is. Hoe werken jullie verder samen met andere zorgverleners in het ziekenhuis?

R: Nou, wij werken heel nauw samen met andere zorgverleners in het ziekenhuis, met name met de radiotherapeuten natuurlijk. Zeker voor mensen die echt in een palliatieve fase zitten. Met de medisch psychologen. Met de mensen van het pijnteam. Ja, met de chirurgen voor deze groep mensen niet zo. Ja, en diëtisten, fysiotherapeuten, verpleegkundigen op de afdeling. Dus, ja, wij werken met heel veel verschillende disciplines samen.

I: Ja.

R: Maar met name voor deze groep mensen met name de radiotherapie.

I: Oké.

R: Daar werken we veel mee samen, ja.

I: Oké. En werken jullie bijvoorbeeld ook veel samen met de huisarts, met de eerstelijn?

R: Nou, ik denk dat dat, dat daar heel veel verbetering te halen is.

I: Oké.

R: En ik denk dat dat soms wel, soms gaat het heel goed. Maar er zijn ook patiënten waar wij helemaal eigenlijk geen contact hebben met de huisarts, om wat voor een reden dan ook. En dat is eigenlijk heel jammer, want als je dat beter zou doen dan zou je mensen waarschijnlijk veel meer uit het ziekenhuis kunnen houden. Maar dat blijkt in de praktijk toch lastig omdat, om daarin nauw met elkaar samen te werken. Maar natuurlijk werken wij samen met de huisartsen. Natuurlijk bellen huisartsen ons. Maar ik denk dat het met name, als het wat ingewikkelder is dat het zeker beter zou kunnen.

I: Oké.

R: Absoluut.

I: Oké. En mijn volgende vraag zou zijn of je ook samenwerkt met maatschappelijk werk, maar die noemde jij volgens mij al een beetje, hè…

R: Niet zo heel veel. Niet heel direct maatschappelijk werk.

I: Oké.

R: Nee. Wel als patiënten klinisch zijn opgenomen. Dan vragen wij hen er nog wel eens bij. Maar voor de poliklinische patiënten, alleen als daar echt een actieve vraag vanuit de patiënt komt, voor een probleem, dan wel maar.

I: Oké.

R: Ja. En er is ook geen structureel overleg of zo waar zij bij zitten.

I: Nee, precies. Want jullie hebben denk ik, neem ik aan, ook zo’n MDO natuurlijk of niet?

R: Nou, er zijn allerlei MDO’s.

I: Oh, oké.

R: En wij hebben op woensdagmiddag dan het grote oncologie MDO. En dat is, maar dat is met name gaat dat over mensen die niet in de palliatieve fase zijn, of over mensen die bestraald moeten worden. En dan hebben wij oncologie bespreking. En dat, daar bespreken wij alle patiënten met elkaar en ook met onze verpleegkundige specialisten.

I: Oké.

R: Dus daar gaat het meer over, ja, relatie en samenwerking met huisarts en dat soort dingen.

I: Ah, oké.

R: Ja, en dan zijn er op indicatie, zijn er allerlei MDO’s waar patiënten mee besproken kunnen worden. Maar in deze twee komt elke patiënt wel een keer ter sprake.

I: Ja, oké. En je noemde ook op een gegeven moment het pijnteam, hè, dacht ik. Maar hebben jullie ook een palliatief team?

R: Ja.

I: Werken jullie daar veel mee samen?

R: Ja. Maar ook met name bij patiënten die of opgenomen zijn geweest. En dat zij er tijdens een klinische opname bij betrokken zijn geraakt. Of als er hele ingewikkelde problemen spelen, dan wel, psychosociaal dan wel met ingewikkelde pijnproblemen, of iets wat wij niet goed onder controle krijgen.

I: Oké.

R: Maar het is niet zo dat structureel elke palliatieve patiënt bij het palliatieve team terecht komt. Helemaal niet zelfs.

I: Nee, oké.

R: En dat is denk ik ook niet nodig, voor iedereen. Maar ook daar zou wel meer gebruik van gemaakt kunnen worden, denk ik.

I: Ja, dus dat jullie eigenlijk meer samenwerken met dat team. Oké.

R: Ja.

I: Oké. Nou, de rol is mij best wel duidelijk eerlijk gezegd. Het volgende thema gaat over communiceren, informeren en gezamenlijke besluitvorming. En binnen de palliatieve zorg is aandacht van belang voor meerde aspecten. Dus natuurlijk uiteraard het fysieke maar ook het psychische, sociale en spirituele welzijn van de patiënt. En in hoeverre komen deze verschillende aspecten aan bod in die gesprekken met jouw patiënten?

R: Nou, hè, bij ons bespreken we, met name gaat het over fysieke problemen. Psychische klachten komen toch ook wel veel aan bod. Met name als mensen, ja, of toch wel echt, ja, echt depressieve klachten krijgen. En met name ook veel hebben wij het over slaapproblemen bijvoorbeeld. En piekeren, niet kunnen slapen, dat soort dingen. En sociaal al wat minder. Ik probeer het wel altijd te vragen: van, hoe zit de sociale situatie in elkaar? Is er een steun netwerk? Zijn er problemen? Maar, ja, daar is vaak eigenlijk geen ruimte voor. En ik denk ook niet dat mensen nou hun behandeld arts als eerste, ja, aanspreekpunt zien om hun, ja, sociale toestand te bespreken. Ja, en spiritueel dat, daar kan ik heel kort over zijn, daar heb ik het nooit over. Dan verwijs ik ze door naar een geestelijk verzorger of zo. Ik kan daar niks mee.

I: Oké. Ja. En jij gaf aan dat bij de sociale, bij het sociale aspect, dat er dan geen ruimte voor is. Wat bedoel je daarmee?

R: Nou dat is, ik denk dat erop gedoeld wordt, hè, of er sociale problemen zijn. Problemen in relaties met kinderen, thuis, financieel, dat soort, ja, dat is. Als dat speelt is dat vaak zo ingewikkeld, ingewikkelde materie, dat je helemaal daar eigenlijk gewoon geen tijd voor hebt. Je kan dan, je kan daar niet met mensen op in gaan. En de vraag is of dat onze rol ook is.

I: Jazeker, ja.

R: Dat weet ik niet. Er zijn wel eens mensen die dat wel bespreken. Die daar zelf mee komen. En dan maak ik daar ruimte voor. Maar wij zijn, dat klinkt heel hard natuurlijk, geen maatschappelijk werkers die allerlei problemen op kunnen lossen, helaas.

I: Nee, nee.

R: En dan komt dat, ja, toch vaak bij de huisarts of bij, die daar natuurlijk ook niet voor bedoeld is maar goed.

I: Ja.

R: Ja, dat is, ik denk dat je dat in een aflopende volgorde aan bod komt, ja.

I: Oké, ja. Oké, duidelijk. En breng jij zelf die aspecten ook ter sprake?

R: Nou, de fysiek, psychisch en sociaal wel maar spiritueel niet. En dat heeft natuurlijk heel erg te maken met hoe jij zelf in het leven staat en hoe jij zelf daar tegenaan kijkt en hoe belangrijk dat in jouw eigen leven is.

I: Ja.

R: Dan hoef je daar, ja, weet je, als jij zelf daar helemaal geen gevoel mee hebt, of bij hebt, is dat heel lastig omdat bij mensen ter sprake te brengen.

I: Ja.

R: Ja, ik heb daar, nee, ik doe dat niet actief.

I: Nee, oké.

R: Nee.

I: En is dat bij alle patiënten zo? Dus jij gaf aan, hè, die eerste drie, die breng je wel zelf ter sprake als ik jou goed heb begrepen.

R: Ja.

I: En is dat bij alle patiënten zo?

R: Nou, ik probeer wel als ik mensen voor het eerst, nou, niet bij alle patiënten maar, hè, mensen die in zo’n traject zitten, een palliatief traject. Als ik die mensen de eerste paar keer zie probeer ik wel echt een beetje in kaart te brengen van, nou, wat zijn dat voor een mensen? Hoe is hun omgeving? Hoe steekt dat in elkaar? Wat voor een problemen zijn er? Ja.

I: Oké. En is dat ook zo bij mensen die laag zijn opgeleid of niet zo heel veel lijken te snappen?

R: Ja, ik denk dat ik er dan extra moeite vaak voor doe.

I: Oké.

R: Omdat ik weet dat er, of tenminste dat weet ik niet want daar heb ik helemaal geen studies of zo over gelezen maar, in mijn niet zo hele lange ervaring, dat daar vaak meer problemen zijn.

I: Ah zo.

R: Ja, problemen om naar het ziekenhuis te komen omdat er geen geld is voor een taxi. Omdat er een zoon is die, weet ik veel, die veel drinkt die de patiënt niet kan brengen. Dat zij niet begrijpen wanneer ze welke pillen in moeten nemen. Dus dan, hè, dan. En zeker bij mensen die echt laag geletterd zijn, die bijvoorbeeld ook niet goed kunnen lezen of schrijven, probeer ik daar echt wel extra aandacht voor te hebben. Ja.

I: Oké. En dan bedoel je voor echt op het sociale aspect dus meer?

R: Ja.

I: En hoe vraag je daar dan naar of, hoe werkt dat dan? Kun je daar iets meer over vertellen?

R: Ja, dat is natuurlijk lastig want je bent afhankelijk van wat mensen jou vertellen. Maar vaak kun je al een hele goede indruk krijgen van, ja, hoe iemand in elkaar steekt als je weet wat voor een opleiding zij hebben gedaan en wat voor een werk zij hebben gedaan. En of zij een relatie hebben, of zij kinderen hebben en of zij daar contact mee hebben. Dat is, dat geeft al gelijk een hele boel informatie. En dat, ja, en hoe je verder met zo’n patiënt verder gaat is natuurlijk heel erg afhankelijk van, ja, hoe jij inschat dat mensen informatie kunnen begrijpen bijvoorbeeld. En hoe zo’n thuissituatie is. Want je kan wel tegen iemand zeggen, ja, je moet de komende drie weken elke dag naar het ziekenhuis komen voor bestraling. Maar als dat niet gaat, ja, dan zal je toch iets anders moeten verzinnen.

I: Ja, precies.

R: Of het vervoer moeten regelen.

I: Ja. Maar van zoiets zeg jij dan, dat is niet persé onze rol?

R: Nou, dat is lastig. Dat is heel ingewikkeld. Want aan de ene kant ben je wel, tenminste ik voel dat wel zo, verantwoordelijk om de beste zorg te verlenen aan zo’n patiënt. Aan de ander kant kan het natuurlijk niet zo zijn dat wij taxi’s gaan zitten bellen.

I: Nee.

R: Want, ja, en daar is geen tijd voor. En dat is ook niet mijn werk.

I: Nee.

R: Maar ja, ja, dat gebeurt wel eens, ja.

I: Ja.

R: Ja, je moet daar een soort van, toch een soort van modus in vinden. Of iemand zoeken die dat uit handen kan nemen voor een patiënt.

I: Oké. Ja.

R: Maar goed, je kunt niet de hele wereld beter maken.

I: Nee, precies. En even kijken hoor. En die aspecten, worden die allemaal vastgelegd in een? Sorry, ik moet eerst vragen, werken jullie met een individueel zorgplan?

R: Niet als zodanig dat daar een bepaald formulier voor is, nee. Maar wel in de zin natuurlijk dat er voor elke patiënt een individueel plan voor zorg gemaakt wordt.

I: Ja, precies.

R: Maar dat is niet iets wat een vastgelegd zorgpad of zo is. Of iets wat vastomlijnd is, nee.

I: Nee. Nee, oké, duidelijk. En die aspecten waar wij het net over hadden, hè. Worden die dan ook vastgelegd?

R: Nou ja, in principe hoor je alles wat je met een patiënt bespreekt in de status vast te leggen. Ja, nou ja, niet alles, nee, wel veel. Er wordt wel veel vastgelegd.

I: Oké.

R: Maar dat is niet een, ja, er wordt alleen vastgelegd wat ik in de computer intyp, zeg maar.

I: Nee, oké. Dus, ja, krijgen dan al die aspecten, dus behalve die spirituele, krijgen die evenveel aandacht in het vastleggen in de computer?

R: Nee, de fysieke aspecten staat, by far, voorop. Ja, dat is prominent aanwezig.

I: Ja.

R: Ja, dat is 70% tot 80% van de verslaglegging. En de rest komt er een beetje bij.

I: Ja.

R: En die balans kan wel een beetje verschuiven naarmate er meer problemen op het ene vlak zijn en minder problemen op het andere vlak, maar in principe zijn wij behandelaars van het lichaam, en niet van de geest.

I: Ja. Duidelijk. Oké. Ja, nou moet ik altijd, dit stukje dat moet ik dan altijd gaan voorlezen. Dat vind ik altijd heel awkward. Maar ik ga het toch proberen. “Goede communicatie is de basis voor goede zorg zodat de patiënt goed geïnformeerd is en er sprake kan zijn van gezamenlijke besluitvorming”. Zijn er patiënten bij wie jij de communicatie moeilijke vindt?

R: Ja, absoluut. Mensen die niet goed Nederlands spreken, waarbij er iemand tolkt waarvan jij niet weet of zij vertaalt wat jij zegt. Dat zijn vaak mensen uit andere culturen waarvan je, waarvan ik inmiddels wel weet dat het bij alle culturen geaccepteerd is om bijvoorbeeld te zeggen dat iemand kanker heeft. En als dat een soort, hè, als een vrouw met haar dochter komt en die dochter vertaalt wat ik zeg, en ik kan dat niet verstaan, ja, dan heb ik geen idee wat er bij die patiënt aankomt.

I: Ja.

R: En dat is heel lastig.

I: Ja, daar zit altijd wel een beetje ruis, bedoel je?

R: Ja, daar zit een enorme ruis.

I: Enorme ruis, ja.

R: Ja.

I: Sorry, ja.

R: Nou ja, dat weet je niet.

I: Ja.

R: Dan vind ik communicatie met patiënten moeilijk die het moeilijk vinden om uit te drukken wat zij vinden. Die dat niet goed onder woorden kunnen brengen. Of misschien zelf ook niet weten.

I: Ja.

R: Dat is een grote groep. En mensen die echt zo, hè, laag geletterd zijn, of uit een zo laag sociaaleconomisch milieu komen dat zij gewoon niet kunnen begrijpen wat je zegt.

I: Oké.

R: Dat helemaal niet kunnen overzien. Dat zijn met name drie categorieën patiënten die lastig zijn.

I: Ja. En in welk opzicht is de communicatie moeilijk dan bij deze mensen?

R: Nou ja, dat je. Kijk, je wilt natuurlijk graag overbrengen ten eerste wat, hè, wat het probleem is. Maar ook wat de keuzes zijn in een behandeling. En daarvoor is het heel belangrijk dat je weet wat mensen belangrijk vinden. Zeker als het om een palliatieve situatie gaat. Want ik kan wel zeggen, ja, chemotherapie staat in de richtlijn, moeten we doen. Maar als het een overlevingswinst geeft van drie maanden, en mensen hebben helemaal geen zin in chemotherapie, ja, dan moet je dat gewoon niet doen. En als dat, als die besluitvorming niet gezamenlijk genomen kan worden dan moet jij als dokter dus een beslissing nemen voor die patiënt. En dat is heel moeilijk.

I: Ja.

R: En, ja.

I: Ja, oké. En wat doe je om de communicatie daarin dan te verbeteren? Of om dat, ja.

R: Het ligt er een beetje aan wat het probleem is.

I: Ja.

R: Als het echt een taalprobleem is dan is het vaak wel zinnig om een keer een tolk telefoon in te schakelen. Al is het alleen maar om een keer te toetsen wat iemand al weet, wat er aangekomen is. Dat hoeft niet elke keer maar dat is vaak toch wel zinnig omdat te doen.

I: Ja.

R: Ja, en je moet een soort van modus vinden in wat, dat, hè, waarin de patiënt begrijpt wat jij zegt en je vragen zo formuleert dat mensen daar dus een zinnig antwoord op kunnen geven. En soms is het zo dat je een vraag dus zo moet formuleren dat mensen daar ja of nee op kunnen zeggen.

I: Ja.

R: Maar dat is lastig. Want je wilt ook niet iemand een antwoord in zijn mond leggen.

I: Nee, ja. Precies. En dus er zijn wel eens momenten dat jij bewust jouw communicatie eigenlijk aanpast.

R: Zeker ja.

I: Zou je daar een voorbeeld van kunnen geven misschien?

R: Nou, ik heb bijvoorbeeld, gister heb ik met een mevrouw, die functioneerde zelf op het niveau van een 12-jarig kind, gesproken over chemotherapie.

I: Oké.

R: En wat ik dan bijvoorbeeld doe is dat ik dan, hè, tien poppetjes teken en er drie rood maak, en dan zeggen van, nou, hè, dit is dus, zoveel mensen zijn er ziek. En zoveel mensen hebben er baat bij een behandeling bijvoorbeeld. Dat je probeert om het een beetje te visualiseren of zo of. Ja, om plaatjes te laten zien.

I: Ja.

R: Of om bijvoorbeeld een percentage te visualiseren voor iemand. Maar het kunnen ook hele kleine dingen zijn, hè. Soms helpt het al als je mensen met ‘je’ aanspreekt in plaats van u, zo.

I: Oké.

R: Omdat je dan beter contact kan maken. Of soms helpt het al om, weet ik veel, het even over het Feijenoord T-shirt te hebben om, hè, alleen maar even een soort van weerstand te breken tussen, ja, wat, zeg maar, tussen het bureau en de patiënt.

I: Ja.

R: Ja, ik denk dat je daar eigenlijk de hele dag mee bezig bent.

I: Ja, precies.

R: En hoe langer je mensen kent hoe makkelijker dat wordt natuurlijk.

I: Ja, want je gaf een aantal mooie voorbeelden vond ik. Dus dat je het naar een gesloten vraag toebrengt. En dat je het visualiseert. En dat je, ja, ik noem het altijd even, de afstand verkort. Dus dat je mensen met ‘je’ aanspreekt of dat je ze op hun interesses aanspreekt of op het Feijenoord T-shirt.

R: Ja.

I: Maar zijn er ook nog andere dingen die jij dan doet? Het zijn er veel hoor dus ik.

R: Ja, ongetwijfeld.

I: Ik ben gewoon nieuwsgierig. Oké.

R: Ongetwijfeld.

I: Ja.

R: Maar het is, het werkt ook de andere kant op, hè. Soms is het bijvoorbeeld dat je mensen hebt die heel hoogopgeleid zijn en dan print ik wel eens artikel uit de New England uit en dan, hè, dan zeg ik, ja, hier baseren wij het op. Lees het maar.

I: Ja, lees het zelf maar.

R: Lees het maar.

I: Ja.

R: En kom maar met vragen de volgende keer. En dat wil ook wel eens helpen. Omdat, ja, kijk, als dat het niveau is wat mensen hebben, wat mensen aankunnen, ja, dan is dat prima.

I: Ja.

R: En dat vinden, sommige mensen vinden dat vreselijk maar sommige mensen willen dat ook graag. En dan kan jij maar beter de literatuur aanleveren dan dat ze dat.

I: Zelf gaan doen en iets verkeerds opzoeken of zo, bijvoorbeeld.

R: Ja.

I: Oké. Ja.

R: Ik weet niet, er zijn vast nog heel veel andere manieren waarop wij dat doen maar.

I: Ja, en check jij bijvoorbeeld ook wel eens wat jouw patiënten hebben begrepen?

R: Ja, dat doe ik wel eens. Ik doe wel eens een soort van, hoe noem je dat ook alweer? Zo’n recall of zo.

I: Ja, ik noem ‘m even want het is een hele bekende. Ja, dat je vraagt of mensen.

R: Dat ik aan mensen vraag of zij kunnen samenvatten, wat wij besproken hebben. Mijn ervaring is dat mensen dat heel vervelend vinden.

I: Oké.

R: Dat zij dat als een soort toets situatie ervaren. Wat ik wel eigenlijk altijd doe is dat ik zelf samenvat wat wij besproken hebben. En dat ik ook vraag of dat ook is wat zij hebben besproken. Maar zo’n echte recall dat doe ik eigenlijk alleen als ik bijvoorbeeld een ingreep bespreek met hele ernstige complicaties. Om te toetsen of mensen dat echt hebben begrepen,

I: Ja.

R: wat de consequenties kunnen zijn.

I: Ja.

R: Maar ik merk, tenminste dat gevoel heb ik, dat mensen dat vaak wel als een soort van overhoring ervaren. En dat dat als niet als prettig wordt ervaren omdat te doen.

I: Ja.

R: Maar bijvoorbeeld chirurgen doen dat heel veel, hè?

I: Oké.

R: Ja, dat doe ik wel eens.

I: Oké.

R: Maar het is niet mijn favoriete strategie.

I: Nee, oké.

R: Nee.

I: Oké. In hoeverre is er volgens jou sprake van gezamenlijke besluitvorming bij patiënten in de palliatieve fase?

R: Nou, dat is heel ingewikkeld denk ik omdat je, kijk, echt gezamenlijke besluitvorming, zeg maar, eerlijke gezamenlijke besluitvorming is er eigenlijk nooit omdat jij als zorgverlener heel erg stuurt. En dat is niet persé expres.

I: Nee, precies maar als ik even mag, er iets aan mag toevoegen. Als je er even vanuit gaat dat inderdaad de verhoudingen eigenlijk al scheef zijn. Dus jij weet heel veel over longen en ziekten en degene die geholpen moet worden in principe niks. Dus als wij uitgaan van die balans.

R: Ja, ik denk dat er meestal wel sprake is van gezamenlijke besluitvorming. Er zijn ook mensen die dat niet willen. Die gewoon zeggen, zeg maar wat het beste is. En ik denk dat wij onbewust, dat weet ik wel zeker, dat je, je stuurt gewoon die besluitvorming. Ook omdat je, niet eens omdat je meer kennis hebt, maar ook omdat je zelf een idee hebt over hoe iets zou moeten verlopen. Niet omdat je dat persé die patiënt op wilt leggen, maar ja, je hebt nou eenmaal zelf ook, hè, een mening. En je staat ook zelf ook op een bepaalde manier in het leven en dat neem je gewoon mee in zo’n gesprek. En daar kan je ook niks aan doen, dat is denk ik ook niet erg.

I: Nee, precies.

R: Maar dat moet je je wel realiseren.

I: Ja.

R: Want ik weet bijvoorbeeld van mijzelf dat ik best wel terughoudend ben met zware behandelingen starten bij mensen die toch doodgaan. Maar dat is wel belangrijk dat jij je dat realiseert en dat er ook andere behandelaars zijn die dat heel anders doen.

I: Ja, die dat dan anders zien, oké.

R: Dat kan best als je patiënt bij de ene dokter dat hij met chemo naar buiten komt en bij de andere dokter met een recept morfine. En dat is, dan is er waarschijnlijk in beide gevallen sprake geweest van gezamenlijke besluitvorming maar dan heeft de zorgverlener toch gestuurd.

I: Ja.

R: Kennelijk.

I: Ja.

R: En dat is, dus ja de mening van de patiënt wordt altijd gevraagd en altijd betrokken maar ik denk dat wij veel meer sturen dan wij denken.

I: Oké.

R: Denk ik.

I: Oké. Ja, want denk jij dat het beter zou zijn als er meer gezamenlijke besluitvorming zou zijn?

R: Nou, dat is denk ik heel erg patiënt afhankelijk. Ik denk dat het over het algemeen beter kan.

I: Oké.

R: Het kan natuurlijk altijd beter. Maar ik denk dat het niet, het is niet altijd wat mensen willen, een eigen mening. Hè, hun eigen stem in hun behandeling. En dat is iets wat je in moet schatten. En dat is natuurlijk ook gezamenlijke besluitvorming maar, ja, het kan ongetwijfeld beter. Ja.

I: Ja, oké. Zou jij misschien? Of kun jij misschien een voorbeeld geven van een situatie waarin er heel duidelijk sprake was van zo’n gezamenlijke besluitvorming? En misschien een situatie waar dat juist niet zo was.

R: Nou, ik denk een goed voorbeeld van gezamenlijke besluitvorming is eigenlijk dat consult wat wij net hebben gezien, over dat darmonderzoek, hè.

I: Ja, precies.

R: Daar wordt heel duidelijk een gezamenlijk besluit genomen waarbij ik die mevrouw iets aanbiedt wat zij duidelijk niet wilt en daar uiteindelijk ook in meegaat.

I: Ja.

R: Terwijl haar wel duidelijk geschetst wordt wat zo’n onderzoek inhoudt en wat de voordelen ervan zouden kunnen zijn. En een voorbeeld van niet gezamenlijke besluitvorming, ik heb net een mevrouw gesproken die had longkanker, uitgezaaid, en die had al vijf lijnen chemotherapie gehad en die wilde graag nog een behandeling en daar hebben wij tegen gezegd, ja, dat gaan we gewoon niet doen. Omdat wij denken, of eigenlijk zeker weten, dat het je wel slechter maakt en dat dat niet gaat helpen. En in dat geval ben je heel, ja, dan heeft zij eigenlijk daar geen stem in. En ik denk ook dat je soms mensen tegen dat soort besluiten moet beschermen als dat, hè.

I: Ja.

R: Als je echt denkt dat het iemand niks gaat opleveren dat je daar toch heel streng in moet zijn.

I: Ja.

R: Maar ja, dat zijn wel, dat is wel ingewikkeld.

I: Ja, oké. Even denken hoor. Ja, ik zat nog te denken wat dat dan zou beteken voor de zorg voor de patiënt maar ook voor jou, zeg maar, in zo’n geval dat je dus eigenlijk geen, dus in het voorbeeld waar er juist geen sprake was van gezamenlijke besluitvorming. Ik zit even te denken hoor.

R: Nou ja, dat is voor beide partijen natuurlijk heel akelig want niemand gaat met een tevreden gevoel naar huis. Ik heb het gevoel dat ik die mevrouw een doodvonnis geef en die mevrouw heeft het gevoel dat ik haar opgeef.

I: Ja, terwijl het eigenlijk.

R: En dat is, je gaat beiden met een, hè, met de beste bedoelingen naar binnen en je komt beiden ontevreden naar buiten. En toch is dat op dat moment de beste beslissing.

I: Ja.

R: Maar dat is niet prettig.

I: Nee. Nee, precies.

R: Nee.

I: Oké.

R: Helaas.

I: Ja. Even kijken hoor hoe ik in de tijd zit. Oh ja. Ik heb nog een aantal vragen en ik heb hier nog een aantal kaartjes met belangrijke thema’s, crisissituaties, wilsonbekwaamheid en levenseinde beslissingen, wettelijke vertegenwoordig in de situatie van acute verslechtering, levensbeschouwing, culturele achtergrond, ziekenhuis opnames, niet behandel afspraken en plaats van zorg en het sterven. En kun je met behulp van een rangordening in de kaartjes aangeven in hoeverre deze onderwerpen aan bod komen tijdens jouw consulten? Dus dan zou je bijvoorbeeld het meest links het kaartje kunnen neerleggen van aspecten die heel vaak voorkomen.

R: Dat daarover gesproken wordt als dat?

I: Ja, dus bijvoorbeeld ziekenhuis opname, dus ik zeg maar even wat, hè,

R: Wel of niet of zo?

I: die bespreek je bijna altijd dus die gaan dan bijvoorbeeld op deze kant of naar boven of wat jij wilt. Oké, dus ik zeg even voor de audioband, aangeven wat je doet want anders dan.

R: Oké, boven liggen dan degene wat eigenlijk altijd besproken wordt.

I: Ja, dus ziekenhuis opnames en niet behandel afspraken.

R: Ja. Crisissituaties bespreek ik eigenlijk altijd. Die nooit. Ja, dit vind ik een moeilijke combinatie, wilsonbekwaamheid en levenseinde beslissingen. Levenseinde beslissingen bespreek ik relatief vaak maar niet in het geval van wilsonbekwaamheid. Dat komt ook niet zoveel voor.

I: Oké.

R: Ik denk van in plaats van zorg bij sterven, zo, denk ik. Ja.

I: Oké. Dus jij hebt bovenaan ziekenhuisopnames, niet behandel afspraken, crisissituaties, plaats van zorg en sterven, wilsonbekwaamheid en levenseinde beslissingen,

R: Ja.

I: levensbeschouwing, culturele achtergrond en de langste wettelijke vertegenwoordiging enzovoorts.

R: Ja.

I: Oké.

R: En dan gaat, dit over mensen die, zeg maar, die ik ken als wilsbekwaam, hè. Als mensen al komen en zij zijn al wilsonbekwaam, ja, dan wordt het natuurlijk wel besproken maar ik bespreek niet met mensen dat. Stel u voor, u wordt slechter, wie moet er dan beslissingen voor u nemen? Dat wordt denk ik bedoeld met dit kaartje.

I: Ja, ja.

R: Ja, nee, dat niet, nee.

I: Oké. En als je dan naar de onderste drie kaartjes kijkt. Worden die dan door andere zorgverleners wel opgepakt? Of bijvoorbeeld de laatste vier of laatste drie.

R: Nee, ik denk dat dat. Ik denk dat wij dat relatief slecht doen.

I: Oké.

R: En, want eigenlijk is het advanced care planning, hé, wat hier ligt. Alvast nadenken over wat als het slechter gaat.

I: Ja.

R: En ik denk dat wij als specialisten in het ziekenhuis zo bezig zijn met de ziekte behandelen,

I: Ja.

R: dat dit helemaal naar de achtergrond verdwijnt.

I: Oké.

R: En bijvoorbeeld als mensen bij het palliatieve team komen, die doen dit heel goed. Maar wij vaak te laat.

I: Oké.

R: Eigenlijk vaak pas als het echt slecht is.

I: En is dit ook wat jij bedoelde met, misschien ook het lijntje naar de huisarts?

R: Ja, dat denk ik wel.

I: Bijvoorbeeld.

R: Ja. Vaak heeft de huisarts dit soort zaken al besproken met de mensen,

I: Ja.

R: en dan weten wij daar helemaal niet van maar daar is vaak wel over gesproken maar niet met ons.

I: Oké. Ja, en wat vind jij daarvan? Is dat ook wat je zegt van, nou, dat is eigenlijk niet mijn rol? Dus het is prima. Of vind je dat dat wel beter kan?

R: Nou, in vind dat het.

I: Want dat kan ook natuurlijk.

R: Het is lastig want je, hè, zolang mensen bij ons komen zitten wij nog op de behandelstoel, dan worden mensen nog actief behandelt. Weliswaar niet met als doelstelling te genezen maar zij worden nog actief behandelt. En dat is een situatie waarin het heel moeilijk is om over einde levensbeslissing te spreken omdat je daarmee, ja, toch een soort van hopeloosheid bij die mensen neerlegt soms. Of mensen dat zo ervaren. Aan de andere kant is het wel echt onze plaats om dit te bespreken, want, hè, met name die niet behandel afspraken, crisissituaties, plaats van zorg en sterven. Als mensen midden in de nacht op de eerste hulp komen en dit is niet besproken worden ze dus opgenomen in het ziekenhuis.

I: Ja.

R: Want mensen komen met een hulpvraag. En die hulpvraag is, maak mij beter of geef mij pijnstilling. En, ja, dan worden mensen dus opgenomen en dat is niet nodig en niet altijd de beste beslissing, achteraf.

I: Ja.

R: En dat had voorkomen kunnen worden als daar eerder over nagedacht zou zijn geweest.

I: Ja, ja, precies. Maar vind jij het dan jullie rol? Ik zeg even jullie, hè, de longartsen, de rol van de longarts in dit geval omdat dan op te pakken?

R: Nou, ik denk dat als jij, deels wel.

I: Oké.

R: Zeker, hè, afspraken rondom wel of niet opnemen in het ziekenhuis, wel of niet reanimeren, wel of niet beademen. Hè, wat te doen bij acute benauwdheid? Kom je naar de eerste hulp? Moet je thuisblijven? Bel je de huisarts? Dat zijn echt wel dingen waar wij, ja, ik denk dat, ja, ik denk dat dit zijn dingen bij uitstek waarvoor je eigenlijk met de huisarts om de tafel zou moeten zitten.

I: Ja.

R: En waarvoor je misschien eigenlijk zelfs wel de huisarts naar het ziekenhuis zou moeten laten komen om gezamenlijk zo’n consult te doen. Want dit zijn allemaal dingen die vaak.

I: Dus dat jij samen met de huisarts,

R: Nou, bijvoorbeeld.

I: met de patiënt gaan zitten? Oh ja, ja.

R: Want, ja, wij weten vaak niet wat de huisarts heeft besproken maar andersom is dat ook zo.

I: Ja.

R: En dat zorgt ervoor dat er soms dingen gebeuren waarvan je achteraf denkt, ja, dat is echt niet goed geweest. En dat is gewoon zonde.

I: Ja, precies.

R: En of dat dan de huisarts moet zijn dat weet ik niet zo goed.

I: Ja.

R: Maar dit is dagelijks een struggle dit.

I: Oké, ja.

R: Ja, en dit is niet zo actueel maar dit wel.

I: Oké.

R: Dit eerste rijtje.

I: Ja, dus de eerste vier?

R: Ja.

I: Ja, oké.

R: Ja, daar is heel veel winst te behalen.

I: Oké. Nou, dat is goed om te weten. Ik had nog één? Of wilde jij hier nog iets over zeggen? Oké, sorry. Ik had nog één hoofdstukje, beperkte gezondheids-vaardigheden. Heb je wel eens gehoord van die term? Van het begrip.

R: Nee.

I: Oké. En wat betekent dit volgens jou?

R: Nou, ik denk dat het over mensen gaat die weinig inzicht, of weinig inzicht hebben in hun eigen gezondheid of die, denk ik, laag geletterd zijn waardoor zij misschien lager intelligent zijn waardoor zij eigenlijk niet goed beslissingen rondom hun eigen gezondheid kunnen begrijpen of kunnen overzien, denk ik.

I: Ja, oké. Ja, dat, de, het begrip wat wij hanteren is dat het de vaardigheid is om informatie over gezondheid te krijgen, te begrijpen en toe te passen. En dan gaat het over functioneel, dus echt leven en schrijven en zo, hè. Maar interactief, dus begrijpend lezen en hoofd van de bijzaken scheiden en dat soort dingen, enzovoorts. En kritisch. Dus dat je ook informatie toepast, ordent en vooruitdenkt en prioriteiten stelt. Hoe vaak heb jij te maken met patiënten met beperkte gezondheidsvaardigheden?

R: Dagelijks.

I: Dagelijks? Oké.

R: Ja. En dat is denk ik beetje een selectie, want onze patiënten zijn vaak uit een wat lagere sociaaleconomische klasse.

I: Ja, precies.

R: En dat komt omdat wij die selectie, heel veel mensen die gerookt hebben en dat, nou ja, dat is die groep mensen vaak.

I: Ja.

R: Maar ik denk dat het ook, dat komt voor in alle bevolkingsgroepen, denk ik, beperkte gezondheidsvaardigheden. En dat heeft ook niet persé met intelligentie te maken. Het kan ook te maken hebben, denk ik, met je kop in het zand steken, het niet willen horen, er niet over willen praten.

I: Ja.

R: Of niet jouw emoties kunnen uiten of weet ik veel wat. Maar ik denk dagelijks, meerdere keren per dag.

I: Oké. En hoe herken jij patiënten met die beperkte gezondheidsvaardigheden?

R: Ja, dat is lastig. Want soms is het heel duidelijk. Soms begrijpen mensen je duidelijk niet. Maar meestal is dat natuurlijk niet zo. Meestal voer je een gesprek en dan zeggen mensen ja en amen. Of ze zeggen, nee, ik wil dit, ik wil dat. Je toetst natuurlijk op geen enkele manier of mensen echt begrijpen of kunnen overzien wat jij hebt uitgelegd.

I: Ja.

R: Ja, tenzij je echt zo’n recall, of zo’n feedback iets.

I: Ja, waar wij het net over hadden.

R: Ja.

I: Maar dat, wat, je gaf ook aan, dat vinden mensen vaak helemaal niet leuk.

R: Nee, en dat zal ook wel komen omdat ze het vaak niet goed kunnen.

I: Ja, nou ja, ja, inderdaad.

R: Dus ja, dat is heel lastig omdat te signaleren, dat weet ik niet zo goed.

I: Oké.

R: Ik weet niet zo goed hoe je dat, zou moeten herkennen.

I: Het is eigenlijk een beetje een gevoel.,

R: Ja, het is een gevoel.

I: Een intuïtief, een intuïtief dingetje? Oké.

R: Ja.

I: Ja. En op welke probeer jij rekening met hen te houden in mondelinge of schriftelijke communicatie?

R: Nou ja, wat denk ik het allerbelangrijkst is is dat je je woordkeuze aanpast.

I: Die gaf jij in het begin ook al aan, dat klopt.

R: En dat je jouw toon ook aanpast. Dat is misschien nog wel belangrijker. Dat mensen zich niet.

I: Ja, dat je ‘je’ zegt bijvoorbeeld ook op een gegeven moment.

R: Ja, dat je ‘je’ zegt.

I: Is dat een voorbeeld?

R: Ja, dat is een voorbeeld.

I: Oké.

R: En, hè, je merkt ook, hè, soms dan zeg je, nou, u heeft dit en dit en dat. En dat is dan een heel ingewikkeld woord en dan zie je mensen al een beetje in paniek raken zo van, ik begrijp niet wat je zegt. En dat is denk ik iets wat je moet voorkomen.

I: Ja.

R: Zo kan je maar beter zo’n term gewoon helemaal niet gebruiken,

I: Nee.

R: of helemaal niet noemen omdat, dat kan een soort, ja, bijna een soort paniek soms triggeren. Dat je gewoon aan mensen ziet dat ze denken, oh, waar heeft zij het nou weer over?

I: Ja, precies.

R: Ja, en schriftelijk, ja, ik denk als je weet dat mensen niet goed kunnen lezen en schrijven, ja, dan moet je ze niet een folder meegeven. Dat is een beetje gemeen.

I: Ja.

R: En ik heb bijvoorbeeld ook een patiënt waarvan ik weet dat hij niet lezen, dan laat ik de secretaresse altijd bellen over afspraken.

I: Oh ja, ja.

R: Maar er zijn vast ook een heleboel mensen die ook niet goed kunnen lezen en schrijven waarvan ik het helemaal niet weet.

I: Ja, dat zou kunnen.

R: We zullen niet bij een eerste consult vragen, hoe goed kunt u eigenlijk lezen?

I: Ja, nee.

R: Je gaat er eigenlijk vanuit dat iedereen dat wel onder de knie heeft.

I: Ja, ja.

R: Maar dat is niet zo.

I: Ja. Oké. Dus als ik jou mag samenvatten, en nogmaals als ik iets verkeerds zeg dan moet je het even aangeven. Maar dat jij jouw eigen taalgebruik aanpast, simpel houdt, dat je moeilijke termen vermijdt.

R: Ja.

I: Dat is eigenlijk wat jij, hoe jij ook de communicatie aanpast, hè?

R: Ja, en wat ik probeer is korte zinnen te maken.

I: Oké.

R: En,

I: Oh ja, dat is ook een goede.

R: ja, soms als je merkt dat het echt moeilijk is voor mensen om, hè, om dingen te zeggen of te vragen, om echt die vragen gesloten te maken. Zoveel mogelijk naar een antwoord sturen.

I: Ja.

R: Maar dat is ook weer een gevaar natuurlijk want je legt daarmee mensen ook dingen in de mond die zij misschien niet willen zeggen, dus.

I: Ja. Oké. Ja. En ervaar je ook ooit barrières in de zorg aan patiënten met beperkte gezondheidsvaardigheden?

R: Nou, wat ik met name vaak merk, en ik weet niet of dat persé met beperkte gezondheidsvaardigheden te maken heeft, maar dat denk ik wel, is dat mensen in de palliatieve fase met beperkte gezondheidsvaardigheden vaak heel lang willen doorbehandelen. Dat die almaar willen behandelen,

I: Oké.

R: behandelen, behandelen. En dat zij niet op een gegeven moment, hè, kunnen zeggen van, nou, is het genoeg geweest. En de kansen zijn nu zo klein geworden,

I: Ja.

R: nu moeten wij ermee stoppen. En dat is wel een belangrijk probleem.

I: Oké. En hoe komt dat denk je? Dat het specifiek bij die mensen is dan.

R: Ik denk omdat zij die beslissingen niet kunnen overzien.

I: Ah zo. Ja.

R: Kijk, het is natuurlijk heel, het zijn allemaal kansberekeningen hè. Als wij chemotherapie geven is de kans dat u over vijf jaar leeft 2% groter dan als wij geen chemotherapie geven. En dat is voor ons al ingewikkeld.

I: Ja.

R: Laat staan als het over jezelf gaat. En laat staan als je niet eens begrijpt wat een procent is. Dan is het natuurlijk onmogelijk om daar iets van te vinden.

I: Ja.

R: En dan is dus de modus waarin mensen gaan is overleven. En dat betekent dus dat er behandelt moet worden. Kosten wat het kost.

I: Ja.

R: En ik heb daar hele, ik heb er echt enorme bezwaren tegen om mensen maar door te behandelen totdat zij uiteindelijk doodgaan.

I: Ja.

R: Dus dat levert wel eens struggles op.

I: Ja.

R: Problemen op.

I: Barrières.

R: Barrières op, absoluut, ja.

I: Oké. Ja, oké, ja.

R: Ja. Ja.

I: Oké.

R: En andere dingen is, hè, misschien dat mensen te laat aan de bel trekken als er problemen zijn omdat zij niet goed hebben begrepen wie zij dan moeten bellen en wanneer zij dat dan zouden moeten doen.

I: Ah zo.

R: Of een klacht niet herkennen. Hè, dat jij ze instrueert, als je oorsuizen krijgt moet je bellen. Maar niet hebben begrepen wat dan oorsuizen eigenlijk is.

I: Ja.

R: Ja, dat soort dingen. Nou, hele vreemde dingen maar ook hele rare dingen. Dat zij hun inhalatie medicatie opdrinken of zo, weet je wel. Dat het niet eens in je opkomt.

I: Ja, dat dat zou kunnen gebeuren.

R: Dat je denkt, hoe verzin je het? Maar dat gebeurt dus.

I: Ja, ja.

R: En dan hebben wij dat kennelijk niet goed uitgelegd. En de apotheek ook niet. En de patiënt was heel creatief. Maar dat zijn dus wel dingen die gebeuren.

I: Ja.

R: En dat is soms heel grappig en soms is dat echt heel dramatisch.

I: Ja, ja. Ja, precies.

R: Dus ja, dat is een probleem.

I: Oké.

R: Kan een probleem zijn.

I: Ja. En dus die barrières die je nu aangaf zijn eigenlijk vooral, liggen dan nu bij de patiënt. Maar ervaar jij ook ooit bij jouw collega's onderling barrières? Zijn er ook barrières tussen collega's onderling bijvoorbeeld?

R: Nou, tussen collega's onderling weet ik niet maar ik merk dat het voor mij wel, als mensen moeilijker, het moeilijker vinden om zaken te begrijpen, dat het voor mij een barrière is om moeilijke beslissingen te bespreken.

I: Oké.

R: Dat dat lastiger is om het over reanimeren te hebben. Lastiger is om het over stervensfase te hebben omdat dat dan, ah, dat weet ik niet, maar omdat dat voor mijn gevoel dan moeilijker is omdat voor die mensen, omdat te overzien.

I: Ja.

R: En omdat te kunnen plaatsen. En dat is denk ik iets wat meer bij mij ligt als zorgverlener dan bij de patiënt.

I: Ja.

R: Maar dat is wel een barrière die, ik vind het moeilijker om met, hoe noem je dat ook alweer? Mensen met een beperkte gezondheidsvaardigheden,

I: Ja.

R: ingewikkelde gesprekken te voeren.

I: Ja, oké.

R: Het is moeilijker, ja.

I: Ja. Oké.

R: Ja, het is een gezondheid, ja, ik weet het niet zo goed wat je daarmee bedoelt.

I: Nou, ik bedoel.

R: Want die hebben geen beperkte gezondheidsvaardigheden.

I: Nee, nee, sorry. Wat ik bedoel is bijvoorbeeld dat jij ziet om je heen, dat jij van een collega ziet dat hij met een bepaalde manier omgaat met dat soort patiënten en dat je daar bijvoorbeeld iets van wilt zeggen. Of dat je daar iets mee wilt. En dat je dat moeilijk vindt bijvoorbeeld.

R: Nou, het is, ja, wij zitten natuurlijk niet bij elkaar in de spreekkamers dus dat is lastiger. Maar het is wel soms zo dat, hè, dat er tijdens zo’n MDO gezegd wordt, nou, ik kom er niet uit met die patiënt. Ik kan daar, hè, er is veel, een soort van over en weer onvrede. Of er zijn barrières om zaken te bespreken. Of het gaat gewoon niet en dat die mensen dan overgenomen worden,

I: Oké.

R: door een andere dokter.

I: Oké, ja.

R: Om te kijken of het dan wel gaat. En soms gaat het dan wel.

I: Ja, dan is er toevallig wel een klik of zo.

R: Dan is er net een andere toon aangeslagen of dan begin je gewoon op een andere voet waardoor het wel gaat. Dus dat zijn wel de dingen die.

I: Ja, ja. Maar dat is eigenlijk heel positief toch dan? Of niet?

R: Ja, ja. Maar er zijn natuurlijk ook heel veel patiënten die wel, hè, waarmee geworsteld wordt wat helemaal niet aan de oppervlakte komt. Problemen die niemand weet, ja.

I: Oké. Ja, het is ook maar net hoe eerlijk die collega dan toevallig is op dat moment.

R: Ja, maar er wordt wel eens gezegd hoor, ik heb deze patiënt, ik kan er niks mee, wie wil ‘m overnemen? En is het, sommige mensen zijn er ook gewoon beter in dan andere mensen.

I: Oké.

R: Maar iedereen heeft zijn clubje van die mensen waar zij mee worstelen, ja.

I: Ja, het hoort er een beetje bij.

R: Ja.

I: Oké.

R: En vaak is het ook zo dat zij naar een andere dokter gaan en dat er precies natuurlijk hetzelfde probleem ontstaat.

I: Ja, precies. Oké.

R: Maar goed het ligt natuurlijk niet altijd aan de patiënt, het kan ook bij de dokter liggen.

I: Ja.

R: Of bij het ziekenhuis, of bij de omgeving, ja, weet ik veel, ja.

I: Ja, precies. Oké. Mijn laatste vraag. Wat zou jij nodig hebben om de zorg beter aan te laten sluiten op dat soort patiënten?

R: Nou, ik denk waar je, wat een enorme hulp zou zijn is als er wat meer een soort van visueel materiaal zou zijn,

I: Oké.

R: wat ik kan uitdelen.

I: Oké.

R: Gewoon plaatjes van longen met tumoren erin.

I: Want dat gebruiken jullie nu niet dan?

R: Nee, wij hebben dat niet echt.

I: Oké.

R: Dus ik probeer dan vaak dingen te tekenen of zo.

I: Ja.

R: Misschien bijvoorbeeld een filmpje waarin uitgelegd wordt wat longkanker is. Of dat soort dingen. Of hoe chemotherapie werkt. Dat zouden dingen zijn, hè, want wat wij natuurlijk vaak doen, wij bespreken iets en dan geven we folders mee zodat mensen het kunnen lezen thuis maar als dat te moeilijk is dan zou, bijvoorbeeld, denk ik, een filmpje of zo hele goede ondersteuning kunnen zijn.

I: Ja.

R: Dus dat is, dat merk ik wel dat ik daar vaak behoefte aan heb om iets mee te kunnen geven.

I: Ja.

R: Dus dat met name. Ja, en ik denk dat je, sommige dingen zullen altijd een worsteling blijven.

I: Ja, precies. Maar niet bijvoorbeeld tijd of zo? Meer tijd?

R: Ja, meer tijd kan ook wel helpen misschien. Nou heb ik wat meer tijd dan de meeste longartsen omdat ik nog in opleiding ben. Dus dat scheelt.

I: Oh ja.

R: En wat ik vaak al doe is als ik weet dat het veel tijd kost dat ik mensen als laatste op een spreekuur laat zetten. Dus dat ik dan kan uitlopen.

I: Oh ja, dat je de planning een beetje slim doet zodat je.

R: Ja.

I: Ja, oké.

R: Ja, dat je gewoon wat meer tijd hebt. Maar goed dat is voor mij nu nog makkelijk. Maar als je nog zelf supervisie moet doen en weet ik veel wat voor andere dingen nog allemaal, dan is dat natuurlijk lastiger.

I: Ja.

R: Maar ja, zeker. Tijd, natuurlijk.

I: Oké.

R: Zeker als je met een tolken telefoon moet doen, dat duurt zo lang. Dat is echt.

I: Ja.

R: Ja. Dan zou je wel een kwartiertje extra kunnen gebruiken.

I: Ja, ja, precies. Dat waren mijn vragen.

R: Nou, dan zijn wij volgens mij hartstikke mooi op tijd klaar.

I: Ja.

Z2radi01

I: interviewer

R: respondent

**I: Even kijken, mijn eerste vraag: wat is uw rol in de zorg voor patiënten in de palliatieve fase?**

R: Nou, mijn rol; ik ben dus radiotherapeut/oncoloog. Op de poli zie ik patiënten, palliatieve patiënten, die verwezen worden voor een palliatieve bestraling. Mijn rol is dan om eerst goed helder te krijgen wat de klachten zijn die de patiënt heeft. Als bestraling daarvoor kan helpen, om klachten te verminderen, dan leg ik daar alles over uit, de voor en de tegens, dus de effecten, maar ook de mogelijke bijwerkingen. Dan bespreken we samen of de patiënt dat wel of niet wil. Daarnaast zie ik het ook als mijn rol om gewoon überhaupt te kijken hoe het met de patiënt gaat, dus of ze voldoende voor zichzelf kunnen zorgen, hoe het met de mensen eromheen gaat, hoe pijnklachten bijvoorbeeld onder controle zijn of andere klachten. Dus daar denk ik ook weer mee.

**I: Hoe werk je met andere zorgverleners? Wat je aangaf, je kijkt dus ook naar de omgeving van de patiënt zelf, maar kijk je dan ook naar andere zorgverleners die om de patiënt heen werken?**

R: Ja, soms bel ik de verwijzer of ik bel de huisarts als ik tegen iets aanloop waarvan ik denk dat de huisarts daar wat mee kan of al mee bezig is, om het wat af te stemmen. Ik denk dat dat de voornaamste mensen zijn. En soms verwijs ik mensen hier naar de zorgpost als er bijvoorbeeld problemen zijn. Meer zo.

**01.46**

**I: Dus extern, zo noem ik het nu maar even, is het eigenlijk de huisarts?**

R: Ja.

**I: En hierbinnen is het af en toe dan dus naar de zorgpost.**

R: En de verwijzer. Ja.

**I: En de verwijzer, de verwijzer is?**

R: De verwijzer is degene die de patiënt naar ons heeft verwezen. Nou ja, dat wisselt natuurlijk nogal wie dat is. Soms verwijs ik ook wel naar het pijnteam, dus dan overleg je daarmee. En af en toe het palliatieve team.

**I: Ik vraag even door over dat externe. Dus je hebt de huisarts, maar werken jullie ook samen met maatschappelijk werk bijvoorbeeld?**

R: Nou, in het ziekenhuis verwijs ik patiënten af en toe wel door naar maatschappelijk werk, maar dat zijn meestal patiënten in de curatieve fase. Als er dat soort vraagstukken zijn, dan zou ik eerder verwijzen naar het palliatieve team, omdat die toch net weer breder kijken dan maatschappelijk werk en zij maatschappelijk werk eventueel weer kunnen betrekken.

**I: Maar hoe komt het dan dat dat meer bij die... Patiënten in de curatieve fase stuur je vaker door naar…**

R: Naar maatschappelijk werk en anders naar het palliatieve team, omdat het palliatieve team gewoon nog weer breder kijkt dan maatschappelijk werk zelf.

**I: Met breder bedoel je? Kun je daar iets meer over vertellen?**

R: Ook meer naar andere klachten. Die weten gewoon specifiek meer de dingen die kunnen spelen in de palliatieve fase, dus die kunnen daar ook meer naar vragen dan maatschappelijk werk, denk ik. Ja. En het palliatieve team weet ook heel veel van de dingen die maatschappelijk werk kan regelen.

**I: Dan gaat het, denk ik, misschien niet alleen over het fysieke, over het psychische, maar ook over het sociale en het spirituele welzijn van de patiënt?**

R: Ja. Ja. Ja.

**I: In hoeverre komen die vier aspecten, dus dat fysieke in ieder geval, het psychische ook, het sociale, wat je net ook al aangaf, de mensen daaromheen, en het spirituele welzijn, aan bod in gesprekken die je hebt met jouw patiënten?**

R: Ja. Nou ja, de meeste van de vier die je noemde komen wel aan bod. Het fysieke sowieso, want daarvoor komen ze hier. Maar dan kijk ik niet puur en alleen naar de klacht die ze hebben, maar ook wel wat breder. Dus, wat kunnen mensen nog, wat willen ze blijven doen? Hebben de bijwerkingen van bestraling daar bijvoorbeeld invloed op, want dan moet je meewegen of je dat wel of niet wil. Psychisch vraag ik eigenlijk ook altijd wel na hoe het op die manier met mensen gaat, of ze het kunnen dragen bijvoorbeeld en ook hoe de omgeving dat… Vaak is er een partner bij en dan vraag ik het daar ook wel aan. Het spirituele vraag ik af en toe, maar dat hangt er meer… Sommige mensen… Ik denk dat ik daar meer over vraag als mensen daar al zelf wat over aandragen, dus dat ik daar wat minder gauw uit mezelf over begin. Ja.

**04.55**

**I: Dat was inderdaad de volgende vraag, of je al die aspecten zelf ter sprake brengt. Die eerste twee lijken me natuurlijk vanzelfsprekend.**

R: Ja. Ja. Ja, nou, psychisch is misschien ook niet vanzelfsprekend. Nee. Maar ik vraag dat bij alle patiënten die ik zie, dus in die zin vind ik het vanzelfsprekend. Het sociale in feite ook. Je wilt toch weten of mensen voldoende netwerk hebben om alles op te vangen en of het lukt om op stap te gaan met mensen en nog de dingen te doen die ze graag willen. Maar spiritueel dus meer als mensen daar zelf over beginnen.

**I: Dus eigenlijk, in hoeverre die aspecten ter sprake komen bij patiënten verschilt per patiënt eigenlijk. Begrijp ik dat goed?**

R: Ja. Dus drie van de vier aspecten komt denk ik bij alle patiënten wel aan bod en het spirituele niet bij iedereen. Ja.

**I: Is er ook een verschil in het type patiënt, wat dat misschien verklaart of zijn er bepaalde patiënten bij wie jij denkt: daar komt het eerder ter sprake?**

R: Dat hangt er in die zin dus van af of mensen daar zelf mee komen. Sommige mensen beginnen bijvoorbeeld zelf over hun geloof. Dan vraag ik daar wat op door, maar ja, niet iedereen heeft het geloof meer natuurlijk, tegenwoordig. Dus dan is het voor mij weer wat vager hoe je daarover praat. Mensen praten daar dan ook minder over. Ja. Ja.

**I: Bijvoorbeeld mensen die laag zijn opgeleid of die het niet zo heel goed snappen; je geeft aan, dat fysieke, psychische en sociale komt vanzelfsprekend eigenlijk ter sprake, als ik je zo goed samenvat.**

R: Klopt.

**I: Dat spirituele is er eigenlijk een beetje als het vanuit de mensen zelf komt. Merk jij of dat bij mensen die laag zijn opgeleid wat meer ter sprake komt, of daar een verschil in zit?**

R: Dat ene aspect bedoel je?

**I: Ja, nou ja, in principe alle aspecten, of daar verschillen tussen zitten.**

R: Poeh, ik weet niet of de verschillen dan zitten in of ze hoog of laag opgeleid zijn. Ja, hoe mensen ermee omgaan… De een kan het accepteren, de ander wordt boos, de ander wordt verdrietig. Ik durf niet te zeggen of dat nou met opleidingsniveau te maken heeft. Dat weet ik niet. Nee. Dat spirituele wisselt ook. Ja…

**I: Dus er is niet echt een bepaalde groep patiënten aanwijsbaar bij wie dat eerder ter sprake zou komen of zo.**

R: Nee, dat denk ik niet. Nee.

**I: De afspraken die over dat fysieke, psychische, sociale en spirituele gemaakt worden, worden die ook vastgelegd in een individueel zorgplan voor de patiënt?**

R: Nou, ik leg het vast in mijn beleid. In HiX heb ik mijn consultvoering en onderaan staat mijn beleid. Daarin leg ik het vast.

**I: Dat snap ik niet helemaal. Wat bedoel je?**

R: Nou, kijk, in HiX heb ik gewoon mijn consultvoering, dus daar schrijf ik op wat iemand mij heeft verteld. Ik schrijf op wat ik heb gevonden bij lichamelijk onderzoek, wat er bij de beeldvorming is. Onderaan heb ik mijn stukje beleid, dus daarin noteer ik of iemand bestraald wordt, welke dosis, welk pijnstillers ik heb aangepast, met wie ik heb overlegd, wat er verder gebeurt. Dus dat leg ik daarin vast. Maar ik maak niet een apart individueel zorgplan. Nee. Dus het komt gewoon in het dossier van die patiënt onder het kopje “beleid”. Ja.

**09.16**

**I: Even denken. Ja, goede communicatie is de basis voor goede zorg, zodat de patiënt goed geïnformeerd is en er sprake kan zijn van gezamenlijke besluitvorming. Zijn er patiënten bij wie jij de communicatie moeilijker vindt?**

R: Nou, de taalbarrière kan natuurlijk een rol spelen, dus als mensen de Nederlandse taal niet zo goed machtig zijn. Dan heb je soms familie die vertaalt en soms een tolk, dus dan kun je dat wel weer opheffen. Ik denk patiënten die nog heel erg in hun boosheid zitten… Dat kan ook wel wat lastiger soms zijn, om hen te bereiken ook. Ja.

**I: Dus de communicatie is moeilijker… Ik zit even te denken. Het gaat eigenlijk alle kanten op, als ik het goed hoor. Het kan aan de taal liggen of het kan aan de gesteldheid van de patiënt op dat moment liggen. Zijn er vaak patiënten die bij jou komen…Die weten dan meestal al dat er iets moet gaan gebeuren, dat ze iets… Toch?**

R: Ja.

**I: Als ik jou goed begrijp.**

R: Ja.

**I: Ik zeg maar even wat. Door de oncoloog wordt verteld wat er aan de hand is. Die zegt: wij stellen voor om dit te gaan doen. Dan komen ze bij jou en dan leg jij hun uit wat dat eigenlijk precies is.**

R: Ja. Ja.

**I: Dat is eigenlijk hoe het werkt. Toch?**

R: Ja, klopt. Ja. Dat is het. Ja.

**I: Dus je hebt ook ooit mensen die heel boos zijn? Hebben die dan net dat nieuws gekregen en komen ze dan al direct bij jou? Is dat soms zo?**

R: Nee. Soms zit er natuurlijk een hele tijd tussen, tussen het moment dat ze de diagnose hebben gehoord en het moment dat ze bestraald worden. Daar kunnen weken, maanden of jaren tussen zitten. Maar iedereen gaat natuurlijk heel erg anders om met zijn ziekte. Ja, de een accepteert het en maakt er nog het beste van, de ander zit in zak en as en de ander wordt boos. Ja. Dus iedereen… Dus ik zie hen niet direct nadat de oncoloog hun heeft gesproken. Ze weten vaak al een tijd wat ze hebben. Maar iedereen heeft een heel ander mechanisme om daarmee om te gaan.

**I: Wat doe jij dan om de communicatie te verbeteren op zo’n moment. Je geeft aan: iedereen is eigenlijk anders, iedereen reageert er toch anders op, met hun eigen mechanismen. Wat is dan jouw mechanisme om daarmee om te gaan, om toch die communicatie goed te laten…**

R: Ja. Nou ja, benoemen bijvoorbeeld. En ernaar vragen. Ja, ik blijf zelf over het algemeen heel rustig en dat kan ook weer zijn weerslag hebben op mensen, dat ze er ook rustiger van worden. Ja, soms zit er bijvoorbeeld boosheid, dat mensen het gevoel hebben dat ze van alles moeten. Nou ja, dat kun je al heel gauw ontkrachten, dat er niks moet. Dus ja, dat wisselt, maar ik probeer wel te reageren op wat mensen zeggen of aangeven of non-verbaal aangeven. Ja. En uiteindelijk, door goede uitleg te geven die ze begrijpen en ook nauwlettend weer te volgen, dus dat ik na een week bijvoorbeeld weer bel of zo; dat kan ook als heel prettig ervaren worden. Ja, zodat je uiteindelijk toch een prettig gesprek hebt.

**13.05**

**I: Behalve wat je aangeeft, dus dat je dingen benoemt en dat je zelf ook heel rustig blijft, zijn er dan nog andere – strategieën is misschien een groot woord – dingen die je gebruikt om dat goed voor elkaar te krijgen?**

R: Nou ja, patiënten in het begin sowieso laten praten, dus ruimte geven. Het mag er zijn wat ze voelen. Ja, verder… Ik weet niet of ik daar vaste strategieën voor heb.

**I: Dat hoeft ook helemaal niet.**

R: Nee. Als een patiënt komt, ga ik niet eerst mijn verhaal vertellen, maar eerst vertellen patiënten en dan vraag ik hoe het gaat. Dan gaandeweg gaan we wel naar mijn stukje toe. Ja.

**I: Ja, precies. Dus als ik het goed begrijp komt de patiënt binnen, die krijgt heel veel ruimte en eigenlijk op dat moment, op basis van dat stuk in het consult, ga jij een beetje op je gevoel kijken hoe je het gaat aanpakken.**

R: Ja, ja, ja.

**I: Als ik het goed begrijp, toch?**

R: Ja, klopt. Ja.

**I: En zijn er dan ook specifieke momenten in zo’n consult waarop jij bewust de communicatie aanpast?**

R: Ja, ja, er zijn altijd wel momenten dat je juist stiltes laat vallen. Communicatie aanpassen; dat vind ik een beetje vaag.

**I: Het is vaag, maar bijvoorbeeld wat je zelf aangaf, dat je dat bijvoorbeeld benoemt. Dat vind ik wel een heel duidelijk voorbeeld. Zijn er dan ook momenten dat je dat… Is dat bijvoorbeeld het moment dat je zoiets hebt van: misschien krijgen mensen helemaal niet mee wat ik aan het vertellen ben. Dat zou zo’n moment kunnen zijn?**

R: Ja, dan zou ik wel kunnen zeggen van, nou, ik merk dat u nog erg boos bent, of, ik merk dat het u veel doet. Ja. Ja, dat gebeurt. Ja.

**I: Het gaat eigenlijk allemaal vanzelf.**

R: Ja, het is niet dat ik er heel erg over nadenk van tevoren, van, hoe pak ik dat aan.

**I: Kan jij misschien ook een voorbeeld geven? Bijvoorbeeld waarbij je één van die strategieën – ik noem het steeds strategieën, maar misschien is het een te groot woord – hebt gebruikt in het consult. Heb je daar een voorbeeld van misschien?**

R: En dan ook bij de palliatieve patiënten?

**I: Ja.**

R: Dan moet ik even terugdenken hoor, wie ik de laatste tijd heb besproken. Ja, ik weet niet of ik dat zo weer kan… Ik heb gisteren wel iemand gesproken in de palliatieve fase. Ja, ik kan me dat niet zo specifiek voor de geest halen.

**I: Nee?**

R: Nee. Nee. Nee.

**16.37**

**I: Check jij weleens bij patiënten wat ze hebben begrepen?**

R: Ja. Ik doe dat niet zozeer in de zin van dat ik vraag of ze het kunnen navertellen. Dat is eigenlijk de ultieme manier om zeker te weten dat ze het begrijpen. Dus op die manier doe ik het eigenlijk niet, terwijl ik wel weet dat dat de beste manier is. Maar ik vraag het meer. Ik vertel het sowieso heel rustig en tussendoor vraag ik: is alles duidelijk? Bijvoorbeeld als ik pijnmedicatie uitleg; ik schrijf het op en vaak bel ik een paar dagen daarna van: hoe gaat het nu met de pijnmedicatie? Dan vraag ik ook na hoe ze het hebben ingenomen. Dus dan doe ik meer op dat moment de controle.

**I: Dat is eigenlijk ook gewoon een check dus.**

R: Uiteindelijk wel. Ja. Dat moet je wel weten. Maar ik vraag niet heel specifiek of ze het nog kunnen herhalen. Nee.

**I: Die belcheck, zo noem ik het even, doe jij dat bij al jouw patiënten?**

R: Nee, niet bij iedereen. Maar met name bij het aanpassen van pijnmedicatie. Dan wil ik ook na een paar dagen weten: heeft het effect? Als het geen effect heeft pas ik het weer aan. Maar dan wil ik ook zeker weten dat ze het goed hebben ingenomen, zoals ik het heb uitgelegd. Vaak is er heel veel uitleg, dus dan is het wel handig om dat op dat moment nog te checken. Ja. Maar ik bel niet iedereen die ik een bestralingsbehandeling-uitleg heb gegeven. Maar met name bij verandering van medicatie bijvoorbeeld. Ja.

**I: Dat is dus ongeacht hoe goed of slecht de communicatie ging met die mensen.**

R: Nee, dat is ongeacht. Ja.

**I: Gebruik jij daar ook weleens visueel voorlichtingsmateriaal voor, filmpjes of iets dergelijks, in het consult? Maak je daar weleens gebruik van?**

R: Ja. Sowieso laat ik regelmatig de beeldvorming zien die van patiënten is gemaakt, dus de CT-scan bijvoorbeeld. Ik vraag altijd even of ze het al gezien hebben en of ze het willen zien. Als ze het willen zien, leg ik vaak even uit wat er afwijkend is en wat we kunnen bestralen. En als mensen willen, dan laat ik een foto zien van zo’n bestralingsruimte, hoe dat eruitziet. Soms teken ik wat, hoe we gaan bestralen. Dat zijn de voornaamste visuele dingen die ik gebruik. Ja.

**I: In hoeverre is er volgens jou sprake van gezamenlijke besluitvorming met patiënten in de palliatieve fase?**

R: Nou, die is groot, denk ik.

**I: Ja?**

R: Ja. Tenminste, ik leg altijd uit aan patiënten dat ik kan vertellen wat we kunnen doen en dat ze zelf ook kunnen afwegen wat ze wel en niet willen, dat ik advies kan geven, maar dat ze uiteindelijk zelf een beslissing moeten/kunnen/mogen nemen. Ja. En ook bij pijnmedicatieaanpassingen zeg ik altijd: we kunnen het nu aanpassen, het mag ook volgende week. Daar geef ik vrij veel ruimte, want vaak komen mensen binnen van, ik moet bestraald worden, en dan zeg ik al vrij snel, u moet niks, maar ik ga uitleggen wat er kan en u kan beslissen of u dat wel of niet wil. Er moet niks. Nee. Nee. Ja.

**20.32**

**I: Is dat voor alle patiënten hetzelfde?**

R: Dat denk ik wel. Ja. Ook bij curatieve patiënten doe ik dat. Ja. Er is altijd een keuze.

**I: Ja, precies. Ja, zeker. Maar aanvullend daarop doel ik bijvoorbeeld ook op patiënten die heel moeilijk dingen begrijpen, of daar de gezamenlijke besluitvorming ook plaatsvindt, dus of die bij alle patiënten hetzelfde is.**

R: Nou ja, kijk, uiteindelijk denk ik dat wel. Ja. Ik denk het wel. Kijk, net als de oudere vrouw, de oudere vrouwelijke patiënt die hier komt, die wil soms het liefst dat ik beslis. Die zeggen dat wel. Nou ja, dan kan ik zeggen van, u kunt dit doen, maar uiteindelijk zeg ik, u moet dit wel willen. Dus in die zin laat ik wel altijd de patiënt zelf beslissen, maar is het soms wel moeilijker voor patiënten. Dat klopt. Maar ik ga niet… Ja, ik kan niet voor andere mensen beslissen. Nee.

**I: Nee, precies. Denk jij dat het beter zou zijn als er meer gezamenlijke besluitvorming zou plaatsvinden?**

R: Zeker.

**I: Ja?**

R: Ja. Dat is heel belangrijk. Hier in het ziekenhuis kan je al gauw het gevoel hebben dat je een ding wordt dat van alles moet ondergaan, waar van alles mee gebeurt en je hebt geen idee. Je wordt een patiënt en een ziekte en je bent dan in die zin niet meer jezelf en dat lijkt me niet zo goed. Dus ik denk dat dat heel belangrijk is. Ja. Ik denk ook dat dat ten goede komt aan therapietrouw bijvoorbeeld, dat mensen soms ook bijwerkingen bijvoorbeeld makkelijker kunnen accepteren, dat dat zo is, omdat je er bewust voor gekozen hebt en omdat je ook weet welke andere behandelmogelijkheden er zijn, want die zijn er natuurlijk altijd. De beste behandelmogelijkheid medisch gezien is niet altijd de beste voor de patiënt. Ja. Dus dat is heel belangrijk.

**I: Wat zou nodig zijn om dat te kunnen verbeteren?**

R: Tijd. Je hebt hier tijd in de spreekkamer nodig om het uit te leggen. Patiënten moeten de tijd krijgen om erover na te kunnen denken, dus dat je een paar dagen later of een week later terugbelt om te vragen wat ze willen, want sommige mensen kunnen het niet in één keer beslissen. Patiënten moeten natuurlijk voldoende informatie krijgen om zelf te kunnen beslissen. Ik denk dat dat de drie belangrijkste dingen zijn. Ja.

**I: Even kijken hoe we in de tijd zitten. Oké. Ik heb nog een aantal vragen over dit thema en ook nog een aantal vragen over beperkte gezondheidsvaardigheden. Als je het goed vindt ga ik die eerst even doen.**

R: Ja, is goed.

**I: Heb jij weleens gehoord van dat begrip “beperkte gezondheidsvaardigheden”?**

R: Nee.

**I: Wat denk je dat het inhoudt?**

R: Ik vermoed dat patiënten dan een verminderd inzicht hebben in gezondheid of factoren die daarop van invloed zijn. Dat zou ik denken.

**24.11**

**I: Ja, precies. Hoe vaak heb je te maken met patiënten met dat soort beperkte vaardigheden?**

R: Tja…

**I: Dus het zijn wat je zegt inderdaad vaardigheden om informatie over gezondheid te krijgen, te begrijpen en toe te passen. Die zijn dan verminderd. Dat is eigenlijk wat het is.**

R: Ja… Ik durf niet zo goed te zeggen hoe vaak dat is. Kijk, als je het dan over roken hebt; uiteindelijk heeft iedereen altijd overal wel een keer gehoord dat het ongezond is. Dus iedereen weet dat het slecht is, maar ze doen er niks mee. Ja, heb je dan beperkte gezondheidsvaardigheden? Ik denk het niet. Maar ja, ik weet het niet. Dus ik denk dat heel veel mensen wel meer weten dan dat ze ermee kunnen doen. Maar goed, dat zijn misschien ook wel vaardigheden, om er iets mee te kunnen doen, met wat je weet wat niet gezond is.

**I: Bijvoorbeeld de patiënten die jij terugbelt, dat staat dan eventjes los van de vaardigheden gaf je aan hè? Het is eigenlijk meer van: die hebben pijn, ik wil weten of het werkt.**

R: Ja. Ja. Ja.

**I: Maar je hebt dus nooit zoiets van, ik bel die mensen omdat ze het waarschijnlijk fout doen, omdat ze er niet zo goed in zijn of dat ze het niet zo goed snappen.**

R: Nee. Nee, ik bel hun meer omdat ik weet dat het zo vaak fout gaat en omdat mensen zoveel informatie krijgen dat ze het… Vaak hebben ze het al eerder een keer van iemand op een andere manier gehoord en dan leg ik het weer uit zoals het eigenlijk hoort, met die pijnmedicatie. Dus dan bel ik na of ze het wel echt begrepen hebben. Maar dat is ook voor een hoogopgeleide lastig, want die onthoudt ook niet alles wat ik in de spreekkamer zeg. Een laagopgeleide ook. Dus dat is denk ik voor iedereen weer gelijk. Ja. Nou ja en mensen echt met een verstandelijke beperking, die bel je nog weer vaker, maar dat is net weer anders. Ja.

**I: Mijn volgende vraag zou zijn: hoe zou je die bijvoorbeeld herkennen?**

R: De mensen met beperkte…

**I: Met beperkte gezondheidsvaardigheden.**

R: Ja, ik denk dat je hen kunt herkennen aan hoe ze bijvoorbeeld met pijnmedicatie dat nu al doen. Maar je weet nooit of dat komt door de patiënt of doordat ze het eerder niet goed uitgelegd hebben gekregen. Dus dat weet je dan niet. Hoe kun je er verder achter komen? Ik weet het niet goed.

**I: Nee?**

R: Nee.

**I: Maar in consulten zijn er ook geen manieren waarop jij rekening houdt met hoe jij communiceert… Ja, hoe zeg ik dat? Ik zit heel even na te denken hoor.**

R: Ja. Maar ja, uiteindelijk praat ik met iedereen in eenvoudige taal en bij iets slimmere mensen gebruik je misschien net een beetje meer moeilijke woorden, verder wat minder. Maar uiteindelijk, de meeste mensen die hier komen zijn leek op het gebied van medische zorg, dus dan heb je sowieso dat je lekentaal gebruikt. Misschien bij minder hoogopgeleide mensen dat je het nog simpeler brengt en het nog een keer duidelijk herhaalt.

**28.30**

**I: Ervaar jij barrières in de zorg aan patiënten die beperkte gezondheidsvaardigheden hebben?**

R: Ja, nee, ik weet het niet. Het blijft voor mij een beetje vaag ook hoor.

**I: Die term?**

R Ja.

**I: Ja, dat klopt.**

R: Ja, wat voor een patiënt moet ik dan voor ogen hebben, denk ik dan. Ze hebben allicht wat meer aandacht nodig, dat je vaker dingen controleert. Ja, dan doe je dat wat meer. Ja.

**I: Even kijken hoor. Dan heb ik ten slotte nog de volgende vraag: dit zijn allerlei belangrijke thema’s voor patiënten in de palliatieve fase. Kun je met behulp van een rangordening aangeven in hoeverre deze onderwerpen aan bod komen in jouw consulten?**

R: Tja… Dit is: behandelafspraken over wel of niet reanimeren?

**I: Ja.**

R: Nou… Dit kan ik bijna naast elkaar leggen. Dit komt nauwelijks aan bod in mijn consult en die een beetje.

**I: Dus ziekenhuisopnames, plaats van zorg en sterven – ik doe dit even voor de audio-opname, sorry hoor – crisissituaties, niet-behandelafspraken, wilsonbekwaamheid en levenseindebeslissingen en wettelijke vertegenwoordiging komen niet aan bod in de consulten.**

R: Nauwelijks.

**I: Nauwelijks. Sorry. En levensbeschouwing en culturele achtergrond, waar we in het begin ook even…**

R: Ja. Deels wel.

**I: Deels wel.**

R: Ja. Maar dit: nee.

**I: Waarom komen die nauwelijks aan bod?**

R: Omdat ik maar een heel klein spilletje ben in die hele zorg voor de patiënt. Ik kom even om het hoekje kijken en dan trek ik me deels ook weer terug uit de zorg voor de patiënt. Een patiënt heeft een hoofdbehandelaar – óf de huisarts óf de verwijzer – bij wie dit naar voren komt. Bij een eenmalig gesprek bijvoorbeeld, als het een eenmalige bestraling is of zo, dan komt dit niet allemaal aan bod. Nee.

**I: Daar wilde ik nog iets over vragen, over die hoofdbehandelaar. Dus de hoofdbehandelaar, in jouw context, is vaak de huisarts.**

R: Nou, nee, het hangt ervan af. Soms is het nog een specialist in het ziekenhuis. Op een gegeven moment wordt vaak de zorg overgedragen aan de huisarts en dan wordt die de hoofdbehandelaar van die patiënt. Ja. De kapitein op het schip, zoals ik het vaak tegen patiënten zeg. Ja. Ja, als ik een patiënt voor één keer zie, dan komt dit niet aan bod. Nee.

**32.34**

**I: Ja, hartstikke duidelijk. Ja, dat waren mijn vragen. Had jij zelf misschien nog vragen?**

R: Nee.

**I: Nee?**

R: Het is wel duidelijk zo.

**[34.45 – einde opname]**

Z2radi02

I: interviewer

R: respondent

**01.00**

**I: Dus ik ga je vragen stellen over jouw rol in de palliatieve zorg, over communicatie en over de patiënten. Mijn eerste vraag is altijd wat breed. Wat is jouw rol in de zorg voor patiënten in de palliatieve fase?**

R: Ja, wij zijn van de Radiotherapie wel heel veel betrokken ook bij palliatieve patiënten. We zien hen natuurlijk regelmatig terugkomen voor pijnbestrijding of andere ongemakken waar we ze met radiotherapie mee helpen. Dan zien we hen op dat moment wel gedurende echt maar een korte periode. Dus je bestraalt hen kort, enkele weken daarna volgt de follow-up en daarna gaan ze in principe weer terug naar de verwijzer, dan wel de specialist dan wel de huisarts die het grote beeld in de gaten houdt. We proberen ook wel zoveel mogelijk de patiënten bij dezelfde arts terug te laten komen, maar als mensen ook zo vaak behandeld moeten worden, dan zullen ze ook meerdere artsen bij ons zien. Ja.

**I: Jij bent radiotherapeut/oncoloog hè?**

R: Ja. Ja.

**I: Je geeft aan: ze zijn maar relatief kort bij jullie. Hoe werken jullie hierin samen met andere zorgverleners binnen het ziekenhuis?**

R: Nou ja, patiënten worden naar ons verwezen door de verwijzer. We zien bijvoorbeeld veel longpatiënten met metastasen, maar ook Kahler-patiënten. Van alles. Vanuit een multidisciplinair overleg in het ziekenhuis wordt een indicatie tot bestraling gesteld. Dan worden de patiënten doorverwezen. We zien hen altijd op korte termijn. We hebben daar strenge richtlijnen voor, binnen hoeveel dagen we patiënten gezien moeten hebben en hoeveel dagen ertussen moeten zitten voordat ze bestraald zijn, voordat ze de eerste bestraling gehad hebben, om hen zo kort mogelijk pijn te laten hebben, doordat je hen zo snel mogelijk helpt.

**I: Je gaf aan: vanuit de longafdeling.**

R: Bijvoorbeeld.

**I: Of vanuit de oncologieafdeling.**

R: Ja, ook.

**I: Dat multidisciplinaire overleg, dat is een wekelijks overleg waarschijnlijk?**

R: Vaak wel. Dat ligt er een beetje aan welke tumor of welk doelgebied het is. Wij als radiotherapeut werken natuurlijk heel breed. We hebben ieder zo’n twee, drie, soms wel vier doelgebieden die we doen. Ik doe zelf bijvoorbeeld longtumoren, hematologische tumoren, huidtumoren en een beetje neurostereo-taxie, dus alleen de bestralingen van het hoofd. Maar palliatie is iets wat we eigenlijk allemaal wel doen. Dus het is niet zo dat ik alleen maar patiënten met uitgezaaide longkanker zie. Dat kan ook bijvoorbeeld van de darm of wat dan ook zijn. Of dat ze een uitzaaiing in het bot hebben, dat maakt niet uit.

**I: Het maakt niet uit waar het in principe zit.**

R: Nee. Nee. Nee.

**I: Is er – binnen het ziekenhuis nog eventjes hè – in ieder geval een centrale zorgverlener voor de patiënt?**

R: Ja, in principe wel. Ja.

**04.23**

**I: Dat zijn jullie meestal niet, als ik jou goed begrijp. Klopt dat? Als ik iets verkeerd zeg moet je je meteen verbeteren hè?**

R: Nee. Zoals ik het begrijp is het meer de verwijzer. Het hoofdbehandelaar-schap, daar heb je het dan over hè?

**I: Ja. Dus dat zou dan eerder een oncoloog zijn of een longarts of zo.**

R: Ja. Ja. Ja. Maar ik zit even te denken of dat nou zo is. Ja, nee, bij palliatieve bestralingen zijn wij nooit de hoofdbehandelaar. Soms heb je wel bijvoorbeeld mensen die voor een curatieve bestraling komen, dat we dan gedurende een langere periode die patiënten bestralen, maar daar moet ik de regels ook even over nakijken. Maar volgens mij voor de palliatieve niet. Nee. Wij zijn nooit in the lead.

**I: Ik heb nog één vraag over dat MDO. Jullie hebben een eigen MDO met Radiotherapie of worden jullie overal bij gevraagd? Hoe werkt dat dan? Of jullie hebben met Radiotherapie een MDO en daar komt dan een oncoloog bijvoorbeeld bij zitten? Hoe werkt dat precies?**

R: Ja. Ja. Als ik bijvoorbeeld het Long-MDO even als voorbeeld neem; daar zitten longartsen bij, daar zitten chirurgen bij die aan longtumoren opereren, daar zitten radiotherapeuten bij, daar zit een patholoog bij, daar zit een radioloog bij. Daar worden alle patiënten besproken, of ze nou curatief of palliatief zijn. De palliatieve patiënten kunnen regelmatig wel in aanmerking komen voor chemotherapie of immuuntherapie. Of als ze pijn hebben of bijvoorbeeld bloed ophoesten of veel last hebben van het hoesten; daar kunnen we ook voor bestralen. Dus dat zijn allemaal indicaties die dan langskomen. Dus daar halen we ook wel palliatieve patiënten vandaan. We hebben ook wel specifieke Palliatie-MDO’s, maar die doe ik dan zelf niet. Daar weet Y. veel meer van dan ik.

**I: Oké. Ja, dat is een goede vraag dan.**

R: Een wervel-MDO bijvoorbeeld heb je ook.

**I: Sorry?**

R: Een wervel-MDO. Dat is meen ik één keer in de twee weken en dat gaat specifiek over patiënten met Kahler-haarden dan wel botmetastasen in de wervels, om die te bespreken. Ja.

**I: Dus als ik je goed begrijp hebben jullie ook een apart palliatief gedeelte. Jullie hebben geen Expertisecentrum Palliatieve Zorg of hebben jullie dat wel? Dat weet ik eigenlijk nog niet.**

R: Jawel.

**I: Ik zit even te denken; ik ben bijvoorbeeld ook in Maastricht geweest, in het MUMC. Daar hebben ze een… Hoe heet het daar? Een Expertisecentrum Palliatieve Zorg Maastricht. Volgens mij heet het gewoon zo. Hebben jullie dat hier ook?**

R: We hebben een palliatief team in ieder geval, waar we mensen naar kunnen verwijzen, die dan naar het brede plaatje kijken. Ik weet, wij werken ook in Delft en daar is er in ieder geval een Palliatie-MDO en volgens mij is dat er hier ook.

**I: Dat zal ik dan nog eens aan Y. vragen. Dat is een goede.**

R: Ja. Die weet precies wat er allemaal geregeld is voor de palliatieve patiënten, qua logistiek en qua MDO’s en dingen. Ja. Ja.

**07.34**

**I: Je noemde zelf ook al op een gegeven moment de huisarts. Hoe werken jullie daarmee samen?**

R: Nou, ik vind zelf de huisarts altijd heel belangrijk bij de palliatieve patiënt. Ik bel regelmatig met huisartsen. We worden ook regelmatig gebeld door huisartsen als patiënten bijvoorbeeld bijwerkingen ook hebben van de bestraling. Maar ook andersom worden weleens patiënten verwezen door huisartsen. Soms is het zo, dan heb ik bijvoorbeeld een patiënt bestraald en die heeft dan toch nog veel pijn. Dan zit je ermee te worstelen telefonisch. Of hij heeft nog veel last van bijwerkingen. Dan heb ik weleens gevraagd van: goh, huisarts, kan je misschien even bij die patiënt thuis langsgaan, want ik vertrouw het niet helemaal. Dat soort dingen. Dus ik vind de huisartsen heel waardevol daarin, zeker ook als mensen… Het is soms heel moeilijk met afbouwschema’s, vind ik altijd. Als je bestraald hebt op het hoofd bijvoorbeeld, de patiënt krijgt een dexamethasonschema en je moet dat gaan afbouwen… Hetzelfde geldt voor pijnmedicatie, je moet dat gaan afbouwen… Bij sommige mensen gaat dat gewoon heel makkelijk en die weten precies hoe of wat, maar soms merk je al dat mensen gewoon zo overladen zijn met alle informatie en dat ze niet precies… Dan heb je zo’n gevoel van: dit gaat niet goed komen met dat afbouwen thuis, dus ik schakel de huisarts even in of hij dat een beetje wil begeleiden of in de gaten wil houden. Ik probeer er ook altijd voor te zorgen dat wat dat betreft, voor de pijnmedicatie bijvoorbeeld, de patiënt één aanspreekpunt heeft. Niet dat hij in het weekend naar ons belt van, goh, ik heb meer pijn, mag ik meer medicijnen, en dan twee dagen daarna weer naar de huisarts. Het is handiger als gewoon…

**I: Eén centrale…**

R: Ja. Dat overleg ik dan wel regelmatig met de huisartsen. Ja. Ja.

**I: Behalve de huisarts, zijn er ook nog andere zorgverleners met wie jullie communiceren, met wie jullie in contact staan?**

R: Ja, heel veel.

**I: Maar bijvoorbeeld maatschappelijk werk of een pastoraal werker, zoiets?**

R: Die heb ik zelf nog niet in hoeven schakelen. Ik weet dat er wel mogelijkheden toe zijn. Ik doe het dan altijd via de zorgpost, die zet dat dan uit. Ja.

**I: Deel 2, dat gaat over communiceren, informeren en gezamenlijke besluitvorming. Binnen de palliatieve zorg is aandacht van belang voor meerdere aspecten, namelijk het fysieke, psychische, sociale en spirituele welzijn van de patiënt. In hoeverre komen deze verschillende aspecten aan bod in jouw gesprekken met de patiënt?**

R: Veel. Ja. Ja, wij hebben gelukkig ook relatief veel tijd voor de patiënt, een half uur. Een half uur tot soms wel drie kwartier. Ik begin ook eigenlijk altijd met de vraag van, hoe gaat het met u, omdat je dan gelijk hoort van: is de patiënt nerveus, zit het hem al tot hier, is hij heel zakelijk en gaat het alleen over zijn pijn of wil hij het ook over andere dingen hebben? Ik probeer ook altijd te kijken of er bijvoorbeeld dingen in de thuissituatie spelen, of er genoeg steun is, of iemand alleen is, dat als hij meer last heeft of wat dan ook, dat er wel een beetje controle op is. Ja. Mijn ervaring is; als ik daarmee begin, dan komt dat al….

**I: Dan komt dat vanzelf goed.**

R: Ja.

**11.17**

**I: Breng jij ooit ook zelf die aspecten echt ter sprake?**

R: Welke aspecten bedoel je precies?

**I: Je zegt: ik begin eigenlijk met een open vraag, van: hoe gaat het met u? Op basis van wat er dan komt ga je door, toch?**

R: Ja. Ja.

**I: Maar bijvoorbeeld het fysieke; dat komt neem ik aan natuurlijk ter sprake.**

R: Ja, sowieso.

**I: Maar bijvoorbeeld het psychische of het sociale, wat je al aangaf, of het spirituele; ben je ook ooit echt zelf degene die daarover begint?**

R: Ja. Tenzij ik duidelijk merk dat de patiënt er niet over wil praten, maar ik probeer dat altijd wel. Ja. Ja. Ja. Ik heb ook altijd gewoon een kopje “sociale anamnese”. Dat staat er steevast. Ik vraag dan natuurlijk, bent u getrouwd, heeft u kinderen, maar ook, wat doet u een beetje op een dag, hoe vult u uw dag in. Op basis van wat de patiënt daarover zegt kun je al inhaken en weet je een beetje hoe het met de patiënt gaat. Ja.

**I: Dus eigenlijk is die aanleiding van het gesprek heel belangrijk voor jou.**

R: Ja. Ja.

**I: Is dat bij alle patiënten hetzelfde?**

R: Nee. Het is lastig onder woorden te brengen, maar ik denk dat je dat ook gewoon ziet aan de reacties van de patiënt of aan hoe hij überhaupt al binnen komt lopen. Loopt hij krom? Is hij een beetje bedroefd? Loopt hij rechtop en is het…

**I: “We gaan het even oplossen.”**

R: Ja, precies. En hoelang ze het al weten. Soms weten ze het net een paar dagen. Dan ben ik vaak ook wat beknopter. Dan ga ik niet al die patiënten nog bespreken, want dan zit het die patiënten echt nog tot hier. Dat bewaar ik dan voor een later moment en dan richt ik me puur en alleen op de pijn.

**I: En bij mensen die bijvoorbeeld laagopgeleid zijn, hoe werkt dat dan met die aspecten?**

R: Ja, dat is soms lastig. Alhoewel, die aspecten zijn dan misschien juist weer wat makkelijker dan het technische verhaal van de bestralingen uitleggen en wat er precies aan de hand is. Soms hebben mensen ook gewoon niet zo’n goed beeld van wat er nu aan de hand is of ze snappen het niet helemaal. Dan probeer ik het vaak met ondersteuning van beeldvorming bijvoorbeeld – tekenen helpt heel goed –uit te tekenen en op te schrijven. Dan maak ik gewoon een tekeningetje bijvoorbeeld van een bot of van de long, wat dan ook. Dan zet ik erbij: bijwerkingen tijdens de bestraling. Dat schrijf ik dan allemaal op. De lange termijn, dat schrijf ik ook allemaal op.

**I: Ja. Dan kunnen ze dat mee naar huis nemen.**

R: Ja. Ja. Ja. En altijd laagdrempelig zeggen van: als het niet duidelijk is of u heeft vragen, gewoon bellen. Ik heb het gevoel dat dat wel helpt. Ja.

**14.20**

**I: Dus als ik jou goed begrijp is eigenlijk wat je dus nu omschrijft meer het fysieke. Dat is eigenlijk vrij lastig. Of “lastig”, dat is lastiger dan die andere aspecten bij die mensen. Begrijp ik jou dan goed?**

R: Ja. Ja. Ja. Het is best wel ingewikkeld. De uitleg van wat er precies allemaal gaat gebeuren met de bestraling, daar ben je best wel even mee bezig. Dat is ook een heel technisch verhaal met soms wat moeilijke termen erin. Ja. Soms probeer je dat dan een beetje met een soort van handen en voeten duidelijk te maken. Maar ja, de vraag hoe het met iemand gaat of hoe die zich voelt, daar kun je natuurlijk altijd wel…

**I: Ja. Die kun je aan iedereen stellen in principe.**

R: Ja.

**I: Klopt. Die aspecten, die vier, zijn die ook vastgelegd in een individueel zorgplan?**

R: Een individueel zorgplan, hoe bedoel je dat?

**I: Werken jullie überhaupt met een individueel zorgplan? Wat registreer je eigenlijk van zo’n gesprek bijvoorbeeld?**

R: Ik heb een standaardopmaak die ik er altijd in zet: gewoon de anamnese, medicatie, de voorgeschiedenis oncologisch en ook algemeen, de sociale anamnese, bepaalde contra-indicaties voor bestraling, de familieanamnese soms. Dan lichamelijk onderzoek, de conclusie en de uitleg. Dat is eigenlijk gewoon het vaste format wat ik altijd erin zet. We hebben wel de afspraak ook dat we bij het beleid erin zetten van: hoe oud is de patiënt, wat is zijn conditie, wat is er aan de hand en wat gaan we doen, wat is de dosis, hoe vaak? Dus dat je dat allemaal echt duidelijk hebt geregistreerd. En ook: geeft de patiënt toestemming, heb je de bijwerkingen uitgelegd? Dat vinken we allemaal af. En informed consent. Ja. Ik zet daar ook altijd de bijwerkingen – dat is ook de bedoeling dat we dat doen – in die ik heb besproken met de patiënt. Dat is ook voor mezelf om terug te lezen, van: dat heb ik besproken. Ja.

**I: Duidelijk. Even kijken hoor. Ik wilde nog wat vragen over communicatie. Goede communicatie is de basis voor goede zorg, zodat de patiënt goed geïnformeerd is en er sprake kan zijn van gezamenlijke besluitvorming. Zijn er patiënten bij wie jij de communicatie moeilijker vindt?**

R: Nou ja, soms heb je gewoon meerdere opties. Er zijn meerdere wegen die naar Rome leiden. Je kan wel bestralen, je kan niet bestralen, je kan een hogere dosis geven of soms een wat lagere dosis. Dat is niet altijd zwart-wit. Zeker bij palliatieve patiënten zit daar soms wat verschil in. Ook al heeft iemand misschien precies dezelfde aandoening als iemand anders dan nog kun je voor een andere behandeling kiezen, omdat die patiënt in een slechtere conditie is of eigenlijk niet zo vaak wil komen, niet zo vaak kan komen. Dat is allemaal van invloed. Ik bespreek dan ook met de patiënt van: dit is het voorstel, wat vindt u daarvan, dit is een andere mogelijkheid. Bijvoorbeeld: we kunnen deze bestraling geven, maar dan is de kans dat u er langer baat van heeft groter, maar u heeft tijdens de bestraling meer last van de bijwerkingen. Of we geven nu wat minder, dan heeft u eerder misschien resultaat en minder bijwerkingen, maar dan kan het best zijn dat u ook eerder weer opnieuw moet komen. Dat zijn soms afwegingen die je in overleg met de patiënt maakt. Ja.

**I: Dus als ik jou goed begrijp is het eigenlijk afhankelijk van de patiënt zelf hoe moeilijk of hoe de communicatie verloopt.**

R: Ja. De ene patiënt is daar heel duidelijk in, die zegt, ik wil dit of dat, en de andere patiënt zegt, zegt u het maar. Dat is ook moeilijker. Als ik als dokter moet zeggen, wat moet ik doen voor deze patiënt, dan probeer ik dat toch altijd om te buigen naar dat we toch echt gezamenlijk de beslissing nemen. Maar dat is wel lastig soms. Ja.

**18.56**

**I: Is er bijvoorbeeld een specifiek… Je gaf zelf al aan: de palliatieve patiënt is soms wat ingewikkelder. Zou je misschien ook nog een ander onderscheid kunnen maken van patiënten bij wie je dat echt moeilijk vindt, communiceren.**

R: Ja, een taalbarrière is soms lastig, als mensen niet goed Nederlands spreken. Om dan dat soort ingewikkelde beslissingen te nemen is lastig. Nee, ja. Ik stem het altijd gewoon een beetje af op de patiënt. Dat gaat automatisch. Ik denk er niet bij na. Bij sommige patiënten bij wie ik denk, hier moet ik niet te veel uitweiden of in details gaan treden, want dan volgen ze het niet meer, houd ik het gewoon beknopter, echt een structurele uitleg. Sommige mensen willen het echt meer over de details hebben, willen alles eromheen weten, nou ja, dan bespreken we dat. Ja.

**I: Ik vond het mooi wat je zei: er zijn meerdere wegen die naar Rome leiden. Wat je zei, dat mensen tegen jou zeggen, zegt u het maar, dat jij dan toch gaat buigen en gaat proberen toch tot een gezamenlijk besluit te komen. Zijn er nog meer technieken die je bijvoorbeeld gebruikt?**

R: Om tot een besluit te komen?

**I: Ja. Ik denk het wel. Ja.**

R: Tijd geven. Ik heb ook weleens gedaan; als patiënten toch heel erg opzien tegen bestraling of twijfelen of ze het wel willen… Dan staat er ook op die dag al allerlei voorbereiding van de bestraling gepland. Dan zeg ik: weet u wat, we zeggen het allemaal even af vandaag en u gaat er thuis nog even rustig over nadenken, gaat er met de familie over praten. Dan bel ik u over een paar dagen op, misschien dat u het dan wel weet of dat u nog een keer terugkomt voor meer informatie, dat kan ook. Een beetje op die manier. Dat helpt vaak ook al. Dan nemen ze even afstand, dan kunnen ze het bespreken, nog eens de voor- en tegens voor zichzelf op een rijtje zetten. Ja. Dus dat helpt ook wel vaak goed, vind ik. Ja.

**I: Check je ook weleens of patiënten jou hebben begrepen?**

R: Checken? Nee, ja, ja. Ik denk niet altijd, maar je kan het vaak ook wel zien als ze het niet helemaal begrijpen. Dan vat ik het nog een keer samen in echt steekwoorden. Ik vraag soms ook aan de patiënt van: weet u nog de bijwerkingen die ik heb verteld? Ja, dat ze dat even teruggeven. Dat gaat eigenlijk wel goed. Ja. Ja, vaak doe je ook heel veel gewoon op basis van lichaamstaal.

**I: Ja, op gevoel denk ik hè?**

R: Op gevoel, ja.

**I: Waar zie je aan dat een patiënt het niet begrijpt?**

R: Als ze als een malle zitten te schrijven, maar eigenlijk niet meer horen wat ik zeg.

**I: Ja, die is heel concreet, ja.**

R: Ja. En gewoon de blik in de ogen, dat ze een beetje voor zich uit gaan staren, dat je gewoon weet dat het er hier ingaat en daar er weer uit. Ja.

**I: Dat soort dingen.**

R: Dat soort dingen. Ja. Ja. Ja.

**22.26**

**I: In hoeverre is er volgens jou sprake van gezamenlijke besluitvorming met de patiënt in de palliatieve fase? Die is al een paar keer gevallen.**

R: Ja. Ja. Nou, als ik spreek voor de radiotherapie vind ik dat daar heel veel sprake van is. Het is best wel een paar keer zo geweest dat patiënten verwezen worden voor bestraling, door de verwijzer, en dat na overleg met de patiënt is besloten dat toch niet te doen. Alles gaat in overleg en ik vind ook, er is niks verplicht. Zeker in de palliatieve zorg moet je iets doen om de patiënten te helpen. Je kan hen niet beter maken, maar in ieder geval misschien van hun pijn af helpen. Ja, ik vind ook, dat moet altijd in overleg gaan. Ik vind ook zeker dat je de optie om niets te doen moet bespreken. Dat doe ik ook heel vaak. Dat vinden misschien sommige artsen ook nog wel moeilijk, om niks te doen, maar soms is niks doen beter dan wel iets doen en mensen uit hun eigen thuisomgeving halen, voor misschien een kort effect of misschien helemaal geen effect. Maar het is soms moeilijker om te bespreken, van, misschien is het beter om niks te doen, dan maar alles te doen. Dat gesprek ga ik ook wel aan. Dat ik denk: deze meneer gaat zo hard achteruit, ik kan hem nu wel op de uitstralingen in zijn hoofd bestralen, maar daar heeft hij geen baat meer van, want hij is binnen een maand overleden. Zo bespreek ik het, niet op die manier, maar dat denk ik dan en dan ga je toch anders het gesprek in. Ja.

**I: Is dat voor alle patiënten hetzelfde, die gezamenlijke besluitvorming in de palliatieve fase? Misschien moet ik hem anders stellen. Denk je dat voor alle patiënten die gezamenlijke besluitvorming in dezelfde mate plaatsvindt?**

R: Nou, nee, dat denk ik niet. Ik denk dat het voor sommige patiënten moeilijker is om de gevolgen van een bepaalde keuze te overzien. Dat heeft inderdaad ook met opleidingsniveau te maken. Los van het begrip van wat er gaat gebeuren, maar ook de toekomst, hoe die eruit gaat zien en alle ingewikkelde dingen die erbij komen kijken. Ik denk dat sommige patiënten dat gewoon niet goed kunnen overzien. Ik denk dat het niet voor iedereen heel makkelijk is. Nee.

**I: Denk je dat het beter zou zijn als er meer gezamenlijke besluitvorming zou plaatsvinden?**

R: Ik vind dat altijd goed. Ik vind niet meer dat het is: de dokter zegt het, dus we moeten dat doen. Ik denk dat de patiënt de baas is over wat er gaat gebeuren.

**I: Kun je misschien een voorbeeld geven van een situatie waarin geen gezamenlijke besluitvorming plaatsvond?**

R: Een voorbeeld van een situatie waarin geen gezamenlijke besluitvorming plaatsvindt. Ik heb het idee dat we dat hier gewoon doen.

**I: Dat is ook een antwoord, toch?**

R: Ja. De patiënt wordt naar ons verwezen naar aanleiding van het MDO en er wordt geadviseerd van: u heeft pijn, dus ik zou bestralen. Maar je kan hier altijd nog besluiten om daar in overleg met de patiënt van af te zien. Daarom hebben we ook dat informed consent, dat de patiënt echt achter de behandeling staat die we gaan doen. We zijn eigenlijk verplicht om dat ook te bespreken. We kunnen niet zomaar zeggen: u gaat bestraald worden. Nee. Ziet u het zitten, vraag ik vaak.

**I: Even kijken. Ik heb hier een aantal kaartjes. Belangrijke thema’s die met patiënten besproken kunnen worden. Even kijken hoor. Ik zal deze even hier neerleggen. Ja. Sorry. Belangrijke thema’s in de zorg voor patiënten in deze fase, in de palliatieve fase. Wettelijke vertegenwoordiging. Een situatie van acute verslechtering. Crisissituaties. Ziekenhuisopnames. Levensbeschouwing en culturele achtergrond. Wilsonbekwaamheid. Niet-behandelafspraken en plaats van zorg en sterven. Kun je misschien met behulp van een rangordening in de kaartjes aangeven in hoeverre die aan bod komen in de gesprekken die je hebt met patiënten?**

R: Hoe vaak ze voorkomen?

**I: Ja.**

R: Zoiets, denk ik.

**28.25**

**I: Dus welke komt het meest prominent, het vaakst, voor. Is dat deze of is dat die juist?**

R: Deze.

**I: Oké. Dus ziekenhuisopnames als eerste, crisissituaties, niet-behandelafspraken, levensbeschouwing en culturele achtergrond, wilsonbekwaamheid en levenseindebeslissingen, plaats van zorg en sterven en de wettelijke vertegenwoordiging in een situatie van een acute verslechtering. Dit is even voor de audioband, want dat vergeet ik weleens. Dan liggen de kaartjes heel mooi en dan weet ik het niet meer.**

R: Ik vind het lastig hoor. Het komt allemaal wel naar voren. Deze bijvoorbeeld – de plaats van zorg en sterven, wilsonbekwaamheid, levenseindebeslissingen – komen wel ter sprake, maar dat is vaak iets wat toch ook met de huisarts en met de hoofdbehandelaar eerder besproken wordt. Als ik de patiënt één keer zien voor een bestraling voor pijn, wat één bestraling is, dan ga ik niet… Ik bedoel, soms beginnen mensen er zelf over, maar dit ga ik dan niet uit mezelf ter sprake brengen. Wel dit bijvoorbeeld, niet-behandelafspraken.

**I: Wat je net ook aangaf.**

R: Ja. Ik heb weleens een hele matige man gehad. Ik moet wel weten als hij acuut op onze afdeling onderuitgaat, wat die meneer nog wel en niet wil. Wil hij nog gereanimeerd worden? Dat soort dingen. Daar let ik wel altijd heel erg op. Ja. Dit eigenlijk ook bijna nooit.

**I: Maar als ik jou goed begrijp zit het eigenlijk meer in die rol die jullie hebben, wat je in het begin ook aangaf. Toch?**

R: Ja. Ja. Ja. Ja.

**I: Het laatste onderdeel gaat over beperkte gezondheidsvaardigheden. Heb je weleens gehoord van dat begrip? Beperkte gezondheidsvaardigheden.**

R: Ja.

**I: Wat betekent het, denk je?**

R: Beperkte gezondheidsvaardigheden. Ik weet het niet precies. Het heeft in ieder geval ook met laaggeletterdheid te maken. [Onderbreking door wisseling van kamer]

**I: Ja. Beperkte gezondheidsvaardigheden. Jij wist niet wat het betekende. Laaggeletterdheid dacht je geloof ik hè?**

R: Ja.

**32.01**

**I: De definitie die wij hanteren – het is een lastige definitie hoor, vind ik – is: vaardigheden voor mensen om informatie over gezondheid te krijgen en te begrijpen en toe te passen. Dat zit hem dan in het functionele, dus wat jij zei, lezen en schrijven bijvoorbeeld, maar ook in het interactieve of communicatieve, en in het kritische, dus in het toepassen van informatie. Hoe vaak heb jij te maken met patiënten met beperkte gezondheidsvaardigheden?**

R: Ja, toch wel regelmatig. Oudere mensen die vergeetachtig zijn soms. Buitenlandse mensen. Ja, qua sociaaleconomische klasse ook wel, bij longkanker. Veel patiënten die veel gerookt hebben, die vaak toch uit een wat lager milieu komen. Ja. Dus regelmatig.

**I: Hoe herken je die patiënten?**

R: Ja, intuïtief.

**I: Ja?**

R: Ja, de manier waarop ze antwoorden op je vragen. Geven ze rechtstreeks antwoord op je vraag of geven ze eigenlijk geen antwoord. De woordkeuze die ze gebruiken. Sommige mensen… Afhankelijk van de sociale anamnese. Wat hebben ze altijd als beroep gedaan?

**I: Ja, die is heel concreet inderdaad, ja. Opleidingsniveau. Beroep ook. Ja. Ja.**

R: Precies. En aan de hand van de vragen die ze stellen. Gaat het wat dieper of is het echt heel basaal. Aan de hand daarvan. Ja.

**I: Ik heb er al een aantal gehoord hoor, maar op welke manier probeer jij dan rekening met hen te houden in de communicatie?**

R: Structureren, uitleg, vaste volgorde, herhaling, samenvatten, gebruik maken van visuele hulpmiddelen, dus de scan met de uitleg van wat er allemaal zichtbaar is en/of een tekening.

**I: Wat je zei hè, eigenlijk het tekenen van een long bijvoorbeeld.**

R: Ja. Ja. Herhaling dus ook, dat ze dat als het niet duidelijk is kunnen vragen. Familie. Soms is het te veel voor de patiënt en merk je dat hij het niet meer volgt, maar dan toets ik bij de familie: heeft u het wel begrepen, kunt u het nog een keer bespreken met meneer of mevrouw?

**I: Ervaar je barrières in de zorg aan patiënten met beperkte gezondheidsvaardigheden?**

R: Nou, voor sommige mensen zou je wat meer tijd willen hebben, maar eigenlijk vind ik dat dat bij ons… Ja, eigenlijk kan ik dat ook gewoon regelen. Als ik denk van, nou, deze patiënt heeft gewoon meer tijd nodig, dan boek ik daar gewoon meer tijd voor in mijn poli. Dat is eigenlijk nooit een probleem.

**I: Dus je hebt dat eigenlijk gewoon zelf een beetje in de hand.**

R: Ja.

**I: Wat denk je dat er nodig is om die zorg van die groep patiënten beter aan te… Nee, sorry, dat moet ik even anders stellen. Wat zou jij bijvoorbeeld nodig hebben om zorg beter aan te kunnen laten sluiten bij die groep patiënten, dus bij die groep patiënten met beperkte gezondheidsvaardigheden?**

R: Goede ondersteunende materialen. Wat ik bijvoorbeeld in Maastricht had, wat ik heel fijn vond; ik ben in Maastricht opgeleid. Daar hadden we van die papieren, die kon je afscheuren, met zo’n tekeningetje erop, waar het al op stond. Dan kun je er mooi een pijltje bij zetten. Je kan ook zelf tekenen. Dat kan natuurlijk ook, maar dat vond ik wel prettig. Filmpjes vind ik ook vaak… Die hebben we hier niet echt, van de behandeling. We hebben wel wat, maar niet waarin het heel basic per doelgebied wordt uitgelegd, met filmpjes van: dit is een CT-scan, die gaat er gebeuren. Ik denk dat dat ook wel informatief is. Misschien training van professionals.

**I: In?**

R: Communicatie.

**I: En dan gericht op het spreken of op het intuïtief herkennen van dingen bij patiënten?**

R: Bijvoorbeeld. Ja. Ja. Ik noem zomaar wat.

**I: Prima.**

R: Of had je andere suggesties?

**37.07**

**I: Nee hoor. Nee hoor. Nee. Heb je nog aanvullingen op het interview of zijn er nog dingen die je zou willen toevoegen aan het gesprek?**

R: Ik kan even niks bedenken.

**38.52 – einde opname**

Z2radi03

I: interviewer

R: respondent

**I: Ik begin het interview altijd breed, over jouw rol als zorgverlener in de palliatieve zorg voor patiënten. Je merkte voordat ik de audio opname inschakelde al terecht op dat niet iedereen hier palliatief is op deze afdeling (radiotherapie). Dat is een goede opmerking inderdaad. Maar wat is jouw rol in de zorg voor patiënten en hun naasten in de palliatieve fase?**

R: Nou, die is beperkter dan je zou denken, want de meeste mensen hebben hier een curatieve behandeling. De palliatieve behandeling is over de hele bank genomen korter, waardoor ze heel weinig bij de verpleegkundige hoeven te komen. Snap je?

**I: Wat bedoel je met korter?**

R: Ik noem maar iets, als je keelkanker hebt is de bestralingsperiode zeven weken. Dan komen ze zeven weken hier. Deze mevrouw die je net zag heeft een anuscarcinoom, curatief in principe, en die loopt zeven weken hier. Elke week begeleid ik hen, maar soms wel dagelijks op het eind. Als je palliatief bestraald wordt is het soms eenmalig, soms komen ze vijf keer voor de bestraling, soms vier keer, soms dertien keer. En dan is het maar helemaal de vraag of daar een verpleegkundig begeleider bij moet komen. Dat is even ter illustratie. Maar we hebben ook heel veel mensen die palliatief blijken te zijn tijdens de bestraling bijvoorbeeld, dat het helemaal niet goed gaat. We hebben ook heel veel glioblastomen. Dat zijn mensen met hersentumoren die niet meer beter worden, sowieso niet. Dat is eigenlijk ook een palliatieve behandeling. Die zien we ook wekelijks en soms dagelijks. Soms zien we tijdens de hele bestralingsbehandeling dat het helemaal niet goed gaat en dat het van curatief draait naar palliatief naar afscheid nemen, bij wijze van spreken. Dat het helemaal escaleert.

**I: Dan zie je eigenlijk het hele vervelende traject.**

R: Dat is heel naar. Dan begeleid je hen bij het feit dat het helemaal veranderd wordt. Het hele toekomstbeeld verandert. Wat we ook veel hebben is dat mensen zo gewend zijn om bij ons te komen in de curatieve fase, een paar maanden later blijkt dat ze uitzaaiingen hebben en dan komen ze weer even gedag zeggen of kletsen of worden ze palliatief bestraald. En dan komen ze wel bij ons, want dan kennen ze ons al. Er zijn heel veel mogelijkheden.

**I: Het is echt niet alleen palliatief. Dat is wat je eigenlijk zegt.**

R: Dat is eigenlijk wat ik zeg. Ja. Heel veel mensen komen in principe voor een curatieve behandeling, maar het loopt vaak anders dan je denkt. Dat is zo met kanker.

**I: Helaas.**

R: Helaas. Ja, precies. En soms verandert dan het hele uitgangspunt en melden wij de mensen aan bijvoorbeeld bij het palliatieve team. Dat doe ik veel, frequent. Nou ja, frequent is ook weer overdreven, maar wel regelmatig. Ja. Ja.

**I: Jij werkt op de zorgpost.**

R: Ja.

**I: Kun je misschien iets meer vertellen wat dat precies inhoudt?**

R: Dat is een verpleegkundige post. Wij zijn eigenlijk, vind ik soms weleens, de Eerste Hulp van de Radiotherapie. Dus alle mensen die zich niet lekker voelen, die niet goed gaan, komen sowieso bij ons. Dan doen wij een soort triage als het ware, van: hé, wat is er aan de hand? Kan het zijn van de bestraling, kan het zijn van de chemo? Hebben ze een griepvirus onder de leden, hebben ze een hartinfarct, hebben ze een herseninfarct? We krijgen alles binnen eigenlijk. Dan meten we alle vitale functies. We gaan natuurlijk sowieso de arts erbij halen, een behandelend arts en als die er niet is de consulent. Dan bespreken we het beleid. Ook weer regelmatig brengen we mensen naar de afdeling en worden ze opgenomen of we brengen hen naar de Spoedeisende Hulp, waar dan verder gekeken wordt wat er aan de hand is. Een enkel keertje, als het heel ernstig is, een hartinfarct, dat hebben we wel meegemaakt, maar ook een herseninfarct. Dan gaan we mee naar een scan. Dan proberen we in ieder geval niet weg te gaan bij de patiënt. Dat is wel heel leuk, want daardoor is het heel veelzijdig.

**I: Ja, dat kan ik me voorstellen.**

R: Ik werk met twee andere collega-oncologieverpleegkundigen en wij zorgen dat dat altijd bemand is. Dit zijn de ad-hoc dingen die ik nu noem en voor de rest hebben we standaardgesprekken met mensen. Bijvoorbeeld de KNO’ers, dat is een hele moeilijke groep. Tongkanker, keelkanker, een vreselijke aandoening.

**I: Ingrijpend. Ja.**

R: Ingrijpend, verminkend. Dat plannen we allemaal van tevoren in. Dan weten we: die patiënt komt, we hebben overleg gehad, hij wordt door een arts doorverwezen of door en andere afdeling. Ik ben dan gespecialiseerd in de gynaecologische kankers. Dat betekent dat ik die mensen dan begeleid, leefstijladviezen meegeef, aanspreek… We zijn eigenlijk een heel laagdrempelig aanspreekpunt voor alle mensen die bestraald worden. Daar komt het op neer. We hebben zo onze eigen specialisaties. Bijvoorbeeld, heel veel mensen, dertig tot vijfendertig patiënten per dag krijgen gecombineerd chemo met bestraling. Dat heet chemoradiatie. Dus dan krijgen ze op een andere afdeling de chemo of oraal en dan hier komen ze elke dag voor de bestraling. Die mensen zien we zo structureel. Ongeveer honderdvijftig tot tweehonderd mensen per dag worden bestraald en daarvan zijn er meestal dertig tot vijfendertig chemoradiatie-patiënten. Die zien we echt wel wekelijks allemaal. Dan prikken we bloed en we brengen infusen in en we verbinden heel veel wonden. Ook voor wondzorg komen ze bij ons. Ze leveren urine in, ontlasting, alle lab-dingen. En voor de rest psychische begeleiding. Dan komen ze om even hun hart te luchten, om even te vertellen dat het thuis niet langer gaat. We schakelen ook thuiszorg in als het nodig is, we bellen met huisartsen. Ja, een beetje een spin in het web. Ja.

**I: Ik wilde het net zeggen, ja. Dus eigenlijk staan jullie binnen het ziekenhuis in contact met iedereen, dus zowel met de artsen die jullie erbij betrekken als met de radiologen natuurlijk.**

R: De radiotherapeut hè? Dat is wat anders dan radiologen. De radiotherapeut. Maar ook met de oncoloog op de chemoafdeling. Daar hebben we één keer per week een vergadering mee.

**I: Is dat het MDO toevallig?**

R: Dat is een soort MDO, maar dan van… CRT heet dat. De CRT-bespreking, op donderdag.

**I: CRT. En dat betekent?**

R: CRT, chemoradiatie. Dus dan worden de chemoradiatie-patiënten besproken met de oncologen. Ik houd daar een lijst van bij, een digitale lijst, en die hebben zij dan ook. Dus dan zet ik alle nieuwe mensen erop en die worden dan daar besproken. Verder hebben we contact met diëtistes, maatschappelijk werk, psychologen, logopedisten, met andere poli’s, poli Gynaecologie, poli KNO, poli Oncologie, poli Hematologie. Echt hartstikke leuk. En niet te vergeten het palliatieve team, dat voor ons heel belangrijk is.

**08.20**

**I: Die noemde je ook al, ja. Dat is voor mij heel duidelijk. Dan wil ik graag naar het tweede onderdeel, over communiceren, informeren en gezamenlijke besluitvorming. Ik heb wel een aantal dingen gehoord hoor, maar binnen de palliatieve zorg is aandacht van belang voor meerdere aspecten natuurlijk, niet alleen het fysieke, maar ook het psychische, het sociale en het spirituele welzijn van de patiënt. Je noemde er al een paar. Dat mensen hier komen om hun hart te luchten bijvoorbeeld, zei je al hè?**

R: Ja.

**I: Ik vroeg me af: in hoeverre komen deze verschillende aspecten aan bod in gesprekken met patiënten?**

R: Dat zal afhankelijk zijn van de persoon van de verpleegkundige en de persoon van de patiënt. Als dat klikt, dan ga je veel sneller over een eventuele dood, als de ziekte toch terugkomt of als het slecht afloopt… Ja, als het klikt heb je daar sneller gesprekken over en dat gebeurt vaak, vind ik. Dat mensen toch hun angsten uiten, hun zorg over hoe het verder moet met de kinderen, ik noem maar iets, of mensen met een eigen bedrijf, die niet weten hoe het moet. Dus het is maar net hoe open je ervoor staat om die onderwerpen te bespreken.

**I: Dan bedoel je: hoe open de patiënt is om dat met jou te bespreken?**

R: Maar ook de hulpverlener. Ik vind dat fijn. Ik vind het fijn om daarover te praten. Dan tast ik af bij de patiënt of dat een onderwerp is. Dan is het aan de patiënt om daar gebruik van te maken of niet. Wat we vaak doen is, ik vind dat patiënten vaak heel open en eerlijk zijn, hele fijne goede gesprekken. Soms speelt daar heel veel en dan verwijs ik eigenlijk door naar maatschappelijk werk, maar dat willen ze dan niet, dan willen ze liever hier blijven. Dus voor hen voldoet het. Of ze komen weer terug, dat ze zeggen: nee, dat was toch niets. Maar ook dat is weer afhankelijk van of het klikt.

**I: Jij zegt: die klik Wat is dan een klik? Waar merk je dat aan? Is dat een gevoel?**

R: Ja.

**I: Intuïtie en gevoel misschien?**

R: Intuitie. Of je elkaar mag, denk ik. Of ze denken: dat is wel een zuster, bij haar wil ik wel wat kwijt. Ja, dat denk ik, dat je een hulpverlener vertrouwt. Ja. Ja.

**I: Ja, precies. Breng jij die aspecten ook zelf weleens ter sprake?**

R: Ja. Ja.

**I: Doe je dat bij alle patiënten?**

R: Nee. Nee.

**I: Dat is redelijk resoluut. Maar dan ben ik natuurlijk benieuwd bij welke patiënten dat dan niet gebeurt en bij welke wel.**

R: Nou, als mensen… Ik breng vaak die aspecten naar voren als ik al langer een band heb met mensen. Dus als je iemand hebt voor een hele lange behandeling, mensen worden heel ziek daarin, zijn heel kwetsbaar daarin. We zien ook veel naasten, want die komen natuurlijk heel vaak mee. Als mensen er blijk van geven dat ze daar wel… Hoe zeg je dat nou? Sommige mensen blijven heel erg afstandelijk.

**I: Ja, gesloten…**

R: Dan vind ik het lastiger om het ter sprake te brengen, maar ik probeer het soms wel. Ja. Dat gaat iets beter dan vroeger. Ik ben sinds een tijdje, al een paar jaar, ook gespecialiseerd in begeleiding van mensen met gynaecologische kanker, om seksualiteit weer te herstellen, bij wijze van spreken. Ik ben opgeleid door seksuologen, een half jaar. Nu loopt er weer een studie ook naar, waar ik aan meedoe, waar ik in participeer, een onderzoek. Sindsdien vind ik het iets makkelijker om bepaalde onderwerpen ter sprake te brengen, maar dat komt puur doordat je dan…

**I: Doordat je weet hoe je dat misschien…**

R: Ja, dat makkelijker brengt. Ja. Ja. Dat geldt eigenlijk als ik het zo bekijk voor seksualiteit, wat toch een precair onderwerp is, maar levenseinde of angst voor de dood, angst voor ziekte en diagnose is ook precair. Dat lijkt wel een beetje op elkaar.

**I: Ja. Hele moeilijke, ingewikkelde dingen.**

R: Ja. Ja. Ja. Als je elkaar wat langer kent, heb je ook eerder gesprekken daarover, heb ik het idee, heb ik het gevoel.

**I: Daar kan ik me iets bij voorstellen.**

R: Dat mensen je vertrouwen en denken: waar ik nou nog mee zit, hoe gaat dat dan als ik… Soms komen mensen concreet ook met die vraag: hoe is dat dan, doodgaan? Hoe moet ik dat voor me zien? Dat soort dingen. Maar dat zal je pas durven vragen als je denkt: daar komt misschien wel een antwoord op of er komt misschien helemaal geen antwoord op, maar dan kunnen we het erover hebben. Ja. Ja.

**13.15**

**I: Ja, precies. Die aspecten, is dat bijvoorbeeld bij mensen die laag zijn opgeleid of dingen moeilijk lijken te snappen ook zo of zit daar verschil in?**

R: Nee. Ik heb het idee dat het bij hoogopgeleiden moeilijker is, persoonlijk.

**I: Waarom denk je dat?**

R: Dat is wel een psychologische vraag natuurlijk, hoe dat komt. Ik heb het idee dat die wat meer moeite hebben om… Die willen iets in stand houden, heb ik soms het gevoel, dat zij grip hebben op de situatie. Mensen die laagopgeleid zijn geven het makkelijker uit handen, heb ik soms wel het gevoel, van: zuster, alsjeblieft. Die durven zich kwetsbaarder op te stellen en zijn meer een flapuit, heb ik het idee soms. Dat vind ik soms fijner, fijner om te kunnen troosten. Hoogopgeleiden hebben soms het idee dat ze hun status op moeten houden of iets in die geest of ze benaderen het intellectueel. Soms is het makkelijker juist… Maar goed, ik had het laatst juist helemaal niet hoor. Een vrouw was hoogopgeleid. Dat was een heel fijn mens, maar die had een hersentumor. Die kwam echt concreet en zei: ik wil het toch graag met je bespreken, allerlei hele kwetsbare dingen. Dat waren prachtige gesprekken en die heb ik toen ook doorverwezen naar de palliatieve zorg, wat ze ook heel fijn vond. Die heeft daar vervolgens weer gesprekken over gehad. Die was heel dankbaar dat het zo liep. Die ging nog lang niet dood – die ging wel dood hoor, maar dat kon nog wel een jaar duren – en die was nu nog goed, die had nu het idee van: ik heb alvast wat antwoorden of wat tips gekregen voor straks. Die was prima. Die was ook intellectueel, maar die kon het wel weer opengooien.

**I: Hartstikke fijn toch eigenlijk?**

R: Ja, heel fijn. Ja.

**I: Die aspecten… En dan houd ik erover op hoor ☺.**

R: Ja, nee, prima. Nee, dat maakt mij niet uit.

**I: Maar die aspecten die dan besproken worden met zo’n patiënt, worden die dan ook vastgelegd in een individueel zorgplan?**

R: Nou, een zorgplan zou ik het niet willen noemen, maar wel in het dossier, in het patiëntendossier. Dus in het elektronisch patiëntendossier leg ik vast wat ik besproken heb, dat ik hen heb doorverwezen. Bijvoorbeeld zo’n vrouw, even om daarbij te blijven, dat ik haar doorverwezen heb naar het palliatieve team. Het palliatieve team zet wel een zorgplan erin. Ik beschrijf eigenlijk mijn gesprek en mijn doorverwijzing. Niet tot in detail, want ik vind niet dat iedereen dat hoeft te lezen.

**I: Ja, precies. Maar de aspecten komen wel aan bod.**

R: Ja. Ja. Ja. Ja. Ja. En inderdaad, met toestemming van iemand. Iemand moet er wel klaar voor zijn om doorverwezen te worden naar het palliatieve team, want veel mensen schrikken van het woord hè?

**I: Ja, dat is spannend. Dat is een drempel denk ik hè?**

R: Dus soms probeer ik ook mijn best te doen om dat al in een vroeger stadium te noemen zonder dat ik hen doorverwijs. Weet je wel? Dat ik vertel: we hebben hier…

**I: Dan is de term al gevallen. Oh ja, ja.**

R: “We hebben hier een palliatief team. Dat wil niet zeggen dat u in een palliatieve fase zit of in een terminale fase.” Dan probeer ik het zo uit te leggen. “Maar dan weet u dat u daar terecht kan voor allerlei tips and tricks op het gebied van: stel dat u niet meer beter wordt.” Dan is het: oh, oké, maar dat heb ik nu nog niet nodig. “Nee, u heeft het nu nog niet nodig.” Maar dan is het wel…

**I: Maar dan weet je het alvast.**

R: Ja. Dan klinkt het minder hard. Ik zeg ook weleens aan het begin van de gesprekken… Als ik mensen zeven weken ga zien, dan weet ik zeker dat ze aan de morfine gaan en dan zeg ik het al bij het eerste gesprek. Dan schrikken ze. Dan zeggen ze: ja, maar ik ga toch niet dood? Morfine heeft vaak de associatie met sterven. Dan zeg ik, nee, maar ik noem het expres, want in uw geval is het puur pijnbestrijding en u moet niet schrikken als straks de arts zegt: het is nu beter dat u overgaat op morfine. “Oh, oké.” Dan is het taboe eraf en dan weten ze dat.

**I: Dat is wel een interessante truc, techniek.**

R: Tot nu toe bevalt dat beter dan dat mensen overvallen worden van: ik ga toch niet aan de morfine. De arts noemde morfine, is het dan al zover? Dat heb je toch nog wel hoor. Ja.

**17.40**

**I: Als ik iets dieper mag ingaan op de communicatie, goede communicatie is de basis van goede zorg, zodat de patiënt goed geïnformeerd is en er sprake kan zijn van gezamenlijke besluitvorming. Mijn vraag is of er patiënten zijn bij wie jij de communicatie moeilijker vindt.**

R: Ja, die zijn er zeker. Daar hebben we het net al even over gehad, de hele gesloten mensen die niet willen praten, die stug zijn, argwanend, die moeite hebben met het vertrouwen van mensen of die slechte ervaringen hebben, dan kan natuurlijk ook. Mensen die boos zijn vind ik ook moeilijk. Ik had pas een stel waarvan hij ook een hersentumor heeft. Die hersentumorpatiënten zijn überhaupt moeilijk. Die weten dat het eindig is. Die komen er alleen maar voor dat het tijdelijk beter gaat en soms red je het nog twee jaar, een jaar of twee jaar, maar je gaat dood, hoe jong je ook bent. Die partner was zo boos. Weet je wel? Die kwam binnen en die zat mij echt alsof ik een soort boosdoener was aan te kijken. Dat vind ik heel moeilijk. En zo’n patiënt, die patiënt was veel kwetsbaarder, maar die mocht niet kwetsbaar zijn, want zij zei: dat is helemaal nergens voor nodig. “Praten? Nee hoor, we hebben het prima samen, hè?” Oei, dat vind ik heel moeilijk.

**I: Ja, dat kan ik me voorstellen, ja.**

R: Dat vind ik heel moeilijk. En voor de rest vind ik het, nee, voor de rest niet. Het is eigenlijk afhankelijk van de houding van de patiënt. Dat vind ik moeilijk. Maar de ziekte of het stadium niet, mensen die bijna doodgaan niet. Dat vind ik allemaal niet moeilijk.

**I: Puur gericht op communicatie: in welk opzicht is de communicatie dan moeilijker?**

R: Nou, omdat communicatie voor mij is dat je elkaar vindt op een bepaald level. Bij mensen die ik gesloten vind, afhoudend, stug, boos kan ik het level niet vinden. Dus dan weet ik niet of ze mij begrijpen en ik weet niet of ik hen begrijp.

**I: Je zit niet met elkaar op één lijn. Als ik zoiets zeg en als het niet klopt, moet je dat laten weten hè?**

R: Dat is het. Nee, dat is het. Nee, dat is het. Ja. Ja.

**20.06**

**I: Wat doe je dan om dat toch te verbeteren of proberen dat te verbeteren?**

R: Ja, daar probeer ik natuurlijk gewoon mijn ervaring als verpleegkundige in te gooien. Alweer een jaar geleden, denk ik, had ik een echtpaar waarvan de man ook een hele vervelende houding had, vond ik. De patiënt zelf, met een gynaecologische kanker, had daar last van. Dat vind ik ook moeilijk, als twee mensen het samen lastig hebben. Die man was ook heel boos. In plaats van dat ik in mijn afweer ging zitten, wat ik graag dan doe – dat ik denk, die man negeer ik verder en ik praat vooral met haar – ging ik dat juist niet doen en ging ik me heel erg richten op die man. Ik verwoordde wat ik zag van, ik begrijp dat u heel boos bent, ik zie dat u heel boos bent, waar komt dat vandaan? Het lijkt me voor u ook ontzettend lastig. Ik ging hem extreem, bij wijze van spreken, honoreren en een plek geven. Nou, die man smolt als was op de grond en vanaf dat moment hadden we al die weken een prima relatie. Die vrouw is ook overleden uiteindelijk. Dat is tot het einde… Ik ben ook nog bij haar langs geweest toen ze op sterven lag. Eerst was ze curatief en toen werd ze dus palliatief. Toen is die man ook voor een nagesprek gekomen. Toen heeft die bij wijze van spreken in mijn armen gehuild. Zo kwetsbaar was hij geworden. Er was niet veel voor nodig hoor. Ik wil niet opscheppen over mijn techniek, maar het was wel wat ik moest doen blijkbaar. Dat vond ik wel fijn. Ik vond dat heel mooi om te zien.

**I: Ja, dat kan ik me voorstellen.**

R: Ja. Dus soms probeer je op je intuïtie dit soort communicatieve vaardigheden in te zetten om mensen te ontspannen.

**I: Je zegt: dan gebruik ik mijn ervaring als verpleegkundige. Maar denk je dan bijvoorbeeld ook aan de verpleegkundige die net begint?**

R: Nee, die zal dat misschien moeilijker vinden.

**I: Ja, maar zijn er dan bijvoorbeeld tips?**

R: Ja.

**I: Wat ik eruit opmaak is een hele belangrijke tip dat je op één lijn moet komen met die mensen en dan kun je pas eigenlijk…**

R: Ja. Ja.

**I: Even denken waar ik naartoe wil met mijn vraag. Zijn er nog meer technieken bijvoorbeeld? Je noemde het zelf een techniek en dat is het ook zeker.**

R: Ja, het is een gesprektechniek.

**I: Maar zijn er nog meer technieken die je dan gebruikt, bij mensen bij wie de communicatie niet lekker…**

R: Nou, wat ik eigenlijk net zei is wel een hele belangrijke: verwoorden wat je ziet. Verwoorden wat je ziet, van: ik merk dat u stil wordt, ik merk dat u wat angstig kijkt, wat speelt er op dit moment? Dat kan een communicatietechniek zijn. Het is ook gemeend hoor. Ik wil dat dan ook wel weten. Dat is denk ik belangrijk. Echte interesse. Dat is volgens mij regel nummer één. En dan inderdaad verwoorden wat je ziet. Verder soms ook het samenvatten van wat er wel besproken is. Dan denken mensen: oh ja, oh ja, we hebben het daarover gehad. Of terugkomen op de vorige keer. Wat ik soms ook vraag is: hoe was het gesprek met de arts? Zodat je een ingang… Je gaat ingangen zoeken als de communicatie niet goed gaat. Wat ik ook echt dagelijks doe is vragen: jeetje, hoe kwam u er dan achter dat u kanker had? Dat vinden mensen ook heel fijn.

**I: Ja, dan komt er heel veel context natuurlijk ook voor jou om op in te haken, denk ik hè?**

R: Precies. Ik heb dan meer verdieping in wie die patiënt is en wat eraan vooraf is gegaan, ook in dat stugge. Ook de meest stugge mens wil best vertellen van: op een dag ontdekte ik een knobbeltje en ik schrok me dood. Toen duurde het heel lang bij de huisarts. Dan komt er een heel verhaal en dan hoor ik al dat ze boos zijn op de huisarts. Dan krijg ik alweer informatie. Ja. Oprechte interesse is denk ik regel nummer één. Verwoorden wat je ziet. Naar de beleving vragen van de patiënten. En allebei honoreren. Of “allebei”… De patiënt honoreren sowieso, maar als er naasten bij zijn, is dat ook heel fijn. Ik heb zelf veel in mijn eigen wereld dood en ziekte ervaren in de familie, veel. Dan ging ik vaak mee naar de gesprekken met de hulpverleners, maar dan telde ik gewoon volledig niet mee. Terecht, want mijn broer of mijn schoonzus of weet ik het wie allemaal dood zijn gegaan, kreeg de aandacht. Terecht. Maar hoe fijn is het als iemand zegt: goh, het lijkt me heel wat om daarbij te zitten en dit allemaal te zien. Dat probeer ik heel erg bij de naasten die meegaan of een kind van tien dat erbij zit te doen, van: jeetje, is papa ziek, hoe vind je dat dan in het ziekenhuis? Ik denk: je kan het allemaal negeren, maar…

**I: Dat lijkt me voor de patiënt ook heel fijn. Dan heb je zo’n driehoek eigenlijk of zo.**

R: Ja, regel nummer zeven: honoreer de familie die er omheen zit. Ja, zeker. Ja. Ja.

**25.34**

**I: Je noemde net heel veel technieken. Ik weet niet zeker of ik hem heb gehoord, maar check jij ook weleens of patiënten hebben begrepen wat je hebt uitgelegd?**

R: Ja. Ja. Dat is wat ik probeer te zeggen met de samenvatting. Dan zeg ik waar we het over gehad hebben en dan check ik of dat klopt, of je daarin op één lijn zit. Misschien moet ik wat vaker nog vragen: zou u het in uw eigen woorden kunnen… Maar dat vind ik dan soms lastig om te vragen, want het kan een beetje controlerend klinken. Dat hoeft niet hoor, want ik weet zeker dat het juist goed is. Maar soms vind ik dat lastig, alsof je mensen overhoort. “Heb je het allemaal wel begrepen?” Maar wat ik weleens doe is: het is zoveel en het is ook zoveel jargon wat u hoort, ook van de artsen, wat u van mij dan weer hoort, wat u op het toestel dan weer hoort, op het bestralingstoestel. Zijn er dingen die niet duidelijk zijn voor u of heeft u vragen? Weet u waarom deze dingen allemaal gebeuren? Dan check ik weleens of ze daar nog dingen bij hebben. Maar het is heel belangrijk om te checken: hebben ze het allemaal begrepen? Ja. Ja.

**I: In hoeverre is er volgens jou sprake van gezamenlijke besluitvorming in de palliatieve fase?**

R: Ik vind dat nog wel voor verbetering vatbaar. Dat komt doordat…

**I: Dat was mijn volgende vraag.**

R: Ja. Daar mag nog wel meer aandacht aan besteed worden. Dan heb ik het niet alleen over mijn stukje, want vaak is dat wel duidelijk voor mensen. Zo ingrijpend is het niet wat ik doe. Een wond verzorgen is waar een patiënt ook wel behoefte aan heeft. Maar wat de artsen… Ik vind soms dat de artsen te veel zitten op de behandeling. Maar dat is heel erg individueel verschillend hoor. Maar soms denk ik weleens: zouden die mensen er echt achter staan, achter deze hele behandeling, of is het gewoon puur omdat iedereen ervan uitgaat dat ze het gewoon gaan doen. Vaak ook onder druk van de familie, heel veel familiedruk, vind ik.

**I: Oh ja, dat heb je ook nog natuurlijk hè? Ja.**

R: Ja. En dat de patiënt denkt: oh, niet weer die chemo. Zeker in de palliatieve fase: niet weer! Die weten al hoe ziek ze worden en dat de mensen zeggen: denk erom, je hebt vijf procent kans om toch aan de goede kant te komen. Doe het nou maar, je moet pakken wat je pakken kan. Dat hebben we heel veel hier. Ja. Tenminste, heel veel dat mensen voor die dilemma’s staan en dat ze uiteindelijk vaak toch kiezen voor behandeling. Ja. Ja. Ja.

**28.48**

**I: Als je nu kijkt naar de patiënten, is dat voor alle patiënten eigenlijk hetzelfde? Die mate van gezamenlijke besluitvorming. Jij zegt eigenlijk van, nou, het kan hier en daar nog wel wat beter, als ik jou goed begrijp. Is dat voor alle patiënten hetzelfde?**

R: Nou, kijk, als patiënten goed geïnformeerd zijn, zichzelf goed informeren ook, dan zijn ze een gelijkwaardigere partij voor de besluitvorming. Maar dat is vaak niet zo. Mensen weten daar niet zo heel veel van. Kijk, hoe ziek mensen worden kan je hun niet vertellen. Nu ben jij goed. Op het moment dat jij een griepje hebt en je bent aan het braken, ik noem maar iets, dan denk jij: jemig, wat is dat erg. Maar op het moment dat je je goed voelt weet je niet eens meer zo erg hoe ziek je dan bent. Dat geldt eigenlijk voor die chemo- en bestralingspatiënten ook. Hoeveel last je ervan gaat krijgen om elke dag naar zo’n ziekenhuis te gaan… Alles staat in het teken van de behandeling, van pijn, van ongemak, van misselijkheid, van braken, van obstipatie, diarree, noem het allemaal maar op, duizeligheid, intense vermoeidheid. Ga dat maar eens vertellen aan mensen. Hoe voelen mensen dat? Die zeggen: ja, dat zien we dan wel. Maar dan nemen ze een besluit… Natuurlijk ook op basis van wat ze dan vinden, van: dat zie ik dan wel en dat overleef ik wel. Maar je weet nooit helemaal waar je voor kiest. Dus dat is een ander aspect hoor, trouwens, dat heeft niks met…

**I: Ja, nee, maar dat is een interessant aspect inderdaad.**

R: Dat kan je nooit genoeg vertellen. Het geldt niet voor iedereen. Sommigen zijn heel goed op de hoogte en zeggen: ik wil die behandeling. Die snappen de artsen helemaal. Er zijn ook mensen aan wie de artsen nauwelijks kunnen duidelijk maken wat hun te wachten staat. Er zijn grote groepen KNO’ers bijvoorbeeld, mensen met keelkanker… Dat zijn vaak mensen die drinken, roken, verslaafd zijn, heroïne, dakloos, alcoholisten heel veel… Ja, in hoeverre die helemaal weten wat ze gaan doen weet ik niet. Dus het is ook weer afhankelijk van de situatie van de patiënt. En soms denk ik… Maar zo mag ik niet denken. Ethisch vind ik dat altijd een lastig iets hoor. Er zijn mensen bij wie je denkt: ik geef eigenlijk geen cent voor hen. Voor hen wel, maar ik geef geen cent voor de behandeling en voor de toekomst van die mensen. Als je al dakloos bent en je bent al zo, waarom ga je nog beginnen in godsnaam aan een behandeling. De artsen gaan toch behandelen. Halverwege haken ze af, die mensen, want ze trekken het niet. Dat weten wij dan, als verpleegkundige, van tevoren. Die artsen zeggen van: nee, behandelen gaan we. Dus daar zit nog een… Wanneer moet je zeggen: we gaan niet meer behandelen, u gaat fijn thuis… We zorgen dat u ergens ziek kunt zijn. Waarom ga je die mensen die dakloos zijn in godsnaam chemo en bestraling geven als ze dan zo ziek zijn, met zoveel ellende. Maar goed, met de artsen is dat nog een dingetje. Soms hebben we weleens een discussie daarover. Ja.

**I: Denk jij dat het beter zou zijn als er meer gezamenlijke besluitvorming zou zijn?**

R: Ja, dat denk ik wel. Ja, dat denk ik wel. Een heleboel mensen willen het niet hoor. Dat is de andere kant. Een heleboel patiënten willen het niet: ja, jij bent de dokter, zeg jij het maar. Die balen ervan als de dokter zegt: u kunt ook kiezen voor niet-behandelen of voor die behandeling, dat is wat minder zwaar. “Ja, hallo, doet u nou maar wat u denkt, u heeft ervoor geleerd.” Dus het wordt soms niet gewaardeerd, gezamenlijke besluitvorming. En niet alle artsen zijn daar ook voor gemaakt. Die zeggen ook liever van: u heeft die en die ziekte, dit stadium en u kunt het beste dat gaan doen. Maar ik moet zeggen: hier op de radiotherapie vind ik de dokters wel goed hoor. Ik heb ook op de beenmergtransplantatie gewerkt, heel lang, wat een hele goede afdeling is ook op zich hoor, maar die hematologen joh, die gaan echt… Totdat iemand gewoon bijna de laatste ademhaling uitblaast gaan ze nog steeds voor behandeling. Mensen hebben geen keuze hoor. Echt niet.

**I: Oh, jeetje.**

R: Ja, die gaan zo voor… “Ja, maar dan maakt hij de bocht.” Dan hebben ze het altijd over de bocht maken. Die bocht gaat meestal naar de dood, maar dan is het: het kan ook de andere kant opgaan, dus tot het laatst. Oh, daar hebben we heftige discussies mee gehad altijd. Ja. Nee, je moet geen leukemie krijgen. Maar je moet überhaupt niet ziek worden hoor.

**I: Laten we proberen van niet ☺.**

R: Ja, laten we dat maar proberen.

**33.43**

**I: Ik heb hier een aantal kaartjes en dat zijn belangrijke thema’s in de zorg voor patiënten in de palliatieve fase.**

R: Ja. Ja. Ja. Zeker.

**I: Deze ook nog. Deze ook nog. En ten slotte die. Kun je met behulp van een rangordening in de kaartjes aangeven welke het meeste in gesprekken met patiënten die jij hebt aan bod komen?**

R: Zo. Ja. Oh, ik zit veel te veel te praten.

**I: Nee, nee, nee, nee hoor.**

R: En dan de belangrijkste bovenaan?

**I: Ja, bijvoorbeeld.**

R: Voor mij? Aan mijn kant? Of moet ik het naar jou toe draaien?

**I: Zo is het prima hoor. Dan lees ik ze wel even voor zodat het duidelijk is op de audioband, anders weet ik het straks niet meer.**

R: Dit is een belangrijke, die komt soms niet zo aan bod. Dit hoort een beetje bij elkaar. Ja, dan denk ik die en die. Nou, misschien staat die nog wel wat hoger. Zo. Zo, denk ik.

**I: Oké. Dus niet-behandelafspraken bovenaan.**

R: Ja. En behandelafspraken hè? Je hebt het tussen aanhalingstekens staan. Dus gewoon überhaupt behandelafspraken of niet.

**I: Ja, behandelafspraken. Crisissituaties.**

R: Ja. Wat te doen als het uit de hand loopt allemaal.

**I: Ja, precies. Ziekenhuisopnames.**

R:“Hoe vaak moet ik naar een ziekenhuis?” Ja.

**I: Levensbeschouwing en culturele achtergrond. Wilsbekwaamheid en levenseindebeslissingen. Plaats van zorg en sterven. De wettelijke vertegenwoordiging in situaties van acute verslechtering. Dat is altijd een hele mond vol, die laatste hè?**

R: Ja. Ja, zeker. Ja. Ja.

**I: Als ik even deze drie mag pakken, deze natuurlijk ook hoor, maar deze komen dan het meeste aan bod in gesprekken met patiënten.**

R: Ja. Ja. Ja.

**I: Deze onderste vier, komen die dan bij andere zorgverleners aan bod?**

R: Nou, als mensen in het ziekenhuis liggen komt de dominee of de humanistische… Die komen altijd langs om erover te praten. Culturele achtergrond, dan heb je het bijvoorbeeld over allochtone mensen. Ja, die komt dan ook aan bod. Ja, deze moet misschien maar helemaal onderaan hoor.

**I: Ja, jij doelt op wilsonbekwaamheid en levenseindebeslissingen.**

R: Ja. Ja. Ja. Ja. Ja, daar hebben we het niet zo… Die wilsonbekwaamheid, daar heb ik het niet zo vaak over. Maar wel over levenseindebeslissingen, dat mensen met de huisarts willen afspreken van: dan en dan is het voor mij genoeg. Dit is belangrijk: in het hospice of thuis. En dit is, wie neemt het woord, natuurlijk, op het moment dat mensen aan het doodgaan zijn. Wie neemt de verantwoordelijkheid?

**37.06**

**I: Dat heb ik waarschijnlijk al wel gevraagd, maar hebben jullie vanuit de zorgpost ook contact met de huisarts?**

R: Ja. Soms wel. Ja. Ja. Ja. Ja.

**I: Volgens mij had ik dat nog niet gevraagd.**

R: Ja.

**I: Wel gevraagd hè?**

R: Nee, ik had zelf genoemd dat we ook de huisarts bellen.

**I: Oké. Ja, sorry.**

R: Maar vaak laten we dat wel over aan de artsen hoor. Contact met de huisarts is er weleens hoor, zeker, maar meestal vraag ik de artsen, de behandelaars hier, de radiotherapeuten, het met de huisarts te bespreken en ik bespreek het weer met de thuiszorg. Dat zijn de lijnen. Ja. Dus met de thuiszorg hebben we veel contact mee, regelmatig. Ja. Ja. Duidelijk?

**I: Ja. Ik had nog een laatste onderdeeltje en dat gaat over beperkte gezondheidsvaardigheden. Ik zie dat we bijna door de tijd heen zijn, dus ik ga even gas geven. Heb jij weleens gehoord van het begrip beperkte gezondheidsvaardigheden?**

R: Ja, daar heb ik weleens van gehoord. Ja, zeker.

**I: Wat betekent het volgens jou?**

R: Ik heb zelf het idee dat dat te maken heeft met hoe mensen voor zichzelf kunnen zorgen en hoe ze kunnen zorgen dat ze gezond blijven, preventief, maar ook als ze ziek zijn of als ze slechter van gezondheid worden, hoe ze daarmee om moeten gaan. Dus dan is het voor jezelf zorgen eigenlijk en inzicht hebben in hoe je voor jezelf moet zorgen, dat dat beperkt is bij sommige mensen.

**I: Die sluit heel mooi aan bij “onze definitie”: de vaardigheden van mensen om informatie over gezondheid te krijgen, te begrijpen en toe te passen. Dus eigenlijk gewoon precies wat jij zegt. Dan gaat het over functioneel, dus dan heb je het echt over lezen of schrijven, interactief of communicatief. Dus begrijpend lezen. Maar ook kritisch, dat je informatie toepast en ordent, dat je vooruitdenkt. Dus dat zijn mensen met beperkte gezondheidsvaardigheden. Hoe vaak heb jij te maken met mensen met beperkte gezondheidsvaardigheden?**

R: Wel heel regelmatig ook. Ja.

**I: Heel regelmatig ook.**

R: Ja. Net wat ik je net al vertelde: de junks, de alcoholisten, rokers, zware rokers, mensen heel simpel van geest, de psychiatrische patiënten die we ook hebben die kanker krijgen. Ja.

**39.29**

**I: Hoe herken je patiënten met beperkte gezondheidsvaardigheden? Je noemde er een aantal, die zijn heel concreet natuurlijk hè?**

R: Ja, die zijn concreet.

**I: Maar je hebt denk ik ook een heel groot gebied waar het helemaal niet…**

R: Een grijs gebied.

**I: Letterlijk en figuurlijk misschien.**

R: Ja, inderdaad. Dat merk je in de zorg. Op het moment dat je die mensen leert kennen en dat traject met hen ingaat… Zeven weken loop je mee met hen, als verpleegkundige dan. Dan merk je dat wat je het ene moment hebt gezegd de volgende dag niet begrepen blijkt te zijn. Zo merk je dat inderdaad. Je merkt dat ze thuis toch gekke dingen doen, eigenlijk de leefregels die je met hen bespreekt of de adviezen, de leefstijladviezen, die volgen ze niet op of ze snappen het niet. Ik check misschien te weinig of ze kunnen lezen of niet. Soms komen we daar ook achter, dat mensen toch analfabeet zijn, dus dat je hun folders meegeeft, maar niet checkt of ze wel kunnen lezen. Ja, dat gebeurt nog wel hoor. Ja.

**I: Op welke manier probeer jij dan in de communicatie hier rekening mee te houden, in misschien wel de mondelinge en schriftelijke communicatie?**

R: Nou ja, iets meer eenvoudige termen gebruiken. Je past eigenlijk je communicatie letterlijk verbaal aan. Gebruik maken van tekeningen. Dat doe ik soms nog weleens, dat ik voor hen teken hoe het zit. Ja, dat is ook weer intuïtief, ook een beetje afwachtend. Soms is het natuurlijk ook een taboe, dus dan gaan mensen niet vertellen dat ze niet kunnen lezen. Dat vind ik ook nog weleens lastig om te checken. Maar als in de loop van de tijd blijkt dat ze dingen niet begrijpen, dat ze dingen niet opvolgen, dan ga je op dat niveau zitten en probeer je meer uit te leggen of terug te vragen, van: wat heeft u begrepen van het gesprek gisteren, weet u nog wat u thuis zou kunnen doen daaraan als u heel erg misselijk bent of als u hele erge diarree heeft? Dus dat je dat probeert te checken bij hen. Ja.

**I: Ervaar je weleens barrières in de zorg aan patiënten met beperkte gezondheidsvaardigheden?**

R: Ja. Ja.

**I: Welke barrières?**

R: Nou ja, dat je niet het idee krijgt dat ze het snappen, wat je wilt en doet. Dat ze het niet overzien. Dat vind ik wel heel lastig. Je wilt goede zorg geven en eigenlijk heb je daarvoor… Nou ja, dat is binnen de Psychologie en Pedagogiek bekend, dat er slimme mensen zijn, die snappen wat je bedoelt, die gemotiveerd zijn om het na te volgen, die aantrekkelijk zijn. Niet die hele vieze stinkende… Die hebben we natuurlijk hè? We hebben nu pas… Nou, dat was trouwens helemaal geen domme vrouw. Die had een enorme tumor op haar rug. Dat was helemaal geen domme vrouw, maar die was echt zo weird. Wij zeiden elke dag: je moet het spoelen onder de douche. Die wond was echt zo’n oncologische zweer geworden. Zo groot. Stinken, stinken! Die ging niet onder de douche. Dat is niet fijn. Dat zijn barrières namelijk.

**I: Dat is een barrière, ja.**

R: En als iemand zo ruikt, dat je denkt: oh! En maar zeggen: die wond ruikt, het is goed als u elke dag doucht, elke dag een schone pleister gebruikt. Ze deed het gewoon niet. Dan had het hele weekend die etter… Ja, dat snap je dan niet. Ja, sorry, dit is even inside information hoor, maar dat snap je dan niet hè. Dan denk je: waar zit het hem dan? De arts kwam er ook niet tussen. Dan bespreek je dat met de arts, van: waarom blokkeert dat? Nou, dat was een slechte gezondheidsvaardigheid. Ja. Maar dat blijft een probleem. Welke techniek je er ook op loslaat, je moet gaan kijken: wat slaat aan, wat voor gedeelte snapt die mevrouw niet aan hygiëne en de invloed daarvan op de wondgenezing? Dan heb je met de arts overleg. Ja, die komt er dan ook niet doorheen. Dan denk je: dat is dan de eigen keuze van mensen. Ook dan moeten we het weer loslaten.

**44.09**

**I: Wat zou jij nodig hebben om zorg beter aan te kunnen laten sluiten op patiënten met beperkte vaardigheden, gezondheidsvaardigheden?**

R: Nou, wat fijn is soms, is dat we weleens bijscholing hebben, scholing, bijscholing, ook op dat gebied. Dat kan alles weer even levendig maken. Tips uitwisselen, van: hoe ga jij daar dan mee om? Tekenen voor mensen of een schema van, deze week en deze week, dan zet ik streepjes, of tekeningen van een wond, van, zo gaat het eruitzien, het wordt eerst zo groot en dan wordt het weer kleiner, of bij mensen met gynaecologische kanker, hoe het er dan van onder uitziet, want dat gaat dan ook heel raar eruitzien. Dat heb ik dan inderdaad als tip: weer een scholing een keer.

**I: Omdat je dan beter leert om dingen te gebruiken?**

R: Ja.

**I: Dat je dingen leert tekenen?**

R: Het tekenen zelf niet natuurlijk, maar bijvoorbeeld een tip: als je merkt dat mensen het niet snappen is visualiseren handig. Dat heb ik weleens geleerd van zo’n les over mensen met een beperking in “gezondheidsdingen”. Lessen. Met elkaar blijven delen. Lessen. Iemand die zich daarop heeft gestort, die daar deskundig in is, een psycholoog of een verpleegkundige of een arts. Dat maakt me niet uit, iemand die zich daarin heeft verdiept. Dan met elkaar. Elke verpleegkundige, elke arts loopt daartegenaan. Het is fijn om met elkaar dingen… Dan frist het weer op en denk je: oh ja, die mensen hebben soms andere methodes nodig, laten we die dan uitproberen. Ja. Dat is altijd fijn om te doen. Scholen, bijscholen.

**I: Ja, bijleren.**

R: Bijleren. En ook weer even om erbij stil te staan. Intuïtief kan je dingen goed doen, maar dat wil nog niet zeggen dat je dan ook… Je gaat op je intuïtie af, maar er zijn nog veel meer mogelijkheden natuurlijk die je dan niet weet.

**I: Ja. En heel veel anderen kunnen ervan leren, die nog niet die intuïtie hebben, want dat is het natuurlijk een beetje hè?**

R: Juist. Met elkaar. Ja. Ja. Ja. En ik zit 42 jaar in de verpleging. Ik merk dat ik steeds makkelijker vaardigheden, die zijn erin geslopen in 42 jaar. Dat is wel fijn. Het is een ander gevoel dan toen ik jong was. Toen had ik ook weer andere vaardigheden die ik misschien weer af heb geleerd hoor. Dus elke fase is leuk. Ik weet wel dat de patiënt, toen ik 18, 19 of 20 was, zo’n net beginnende verpleegkundige, leerling nog, dat mensen zeiden: oh, wat heerlijk, jij bent nog zo lekker spontaan, al die routiniers wil ik helemaal niet. Dat patiënten dat aangaven. Nu ben ik natuurlijk ook een routinier en dan hebben we ook weer die jonge mensen nodig die spontaan zijn, dus dat is altijd leuk.

**I: Nou ja, je bent toch nog wel spontaan.**

R: Jawel, ook dat nog wel hoor, heus wel. Maar dat is wel leuk. Ja.

**47.36**

**I: Heb je nog aanvullingen op dit interview?**

R: Nee. Nou, hooguit het onderscheid tussen palliatief… Vanuit het onderwerp is het palliatief, maar eigenlijk geldt dit hele verhaal voor iedereen hè?

**I: Dat zei je al meteen.**

R: Nee, maar ook niet alleen in mijn werk, maar überhaupt.

**I: Zeker. Zeker. Ja. Nee, daar ben ik het ook zeker wel mee eens.**

R: In de hele zorg. Ja. Ja. Ja.

**[48.47 – einde opname]**

Z3a01

I: interviewer

R: respondent

**I: Oké, fijn. Oh, dank je wel.**

R: Alsjeblieft.

**I: Je hebt het allemaal verder ingevuld. Heel fijn. Zijn er tot zover nog vragen?**

R: Nee hoor.

**I: Het eerste thema gaat over jouw rol als zorgverlener in de palliatieve zorg. Wat is jouw rol in de zorg?**

R: Ik ben verpleegkundig consulent. Wat wij proberen is de collega’s, artsen, verpleegkundigen, te ondersteunen in de manier waarop zij de zorg leveren. Zij moeten het werk doen, generalistische zorg. Nou ja, als het lastig wordt, als er vragen komen, dan komen wij meedenken. In principe zijn wij adviserend. Een enkele keer nemen we ook wel wat dingen over, als het gewoon echt lastig blijkt. *[Toevoeging respondent na terugkoppeling: Het gaat hier om het “proactief zorgplan”, in de volksmond ‘palliatieve overdracht’ genoemd. Ik heb het ongetwijfeld zo gezegd, maar bovenstaande toevoeging maakt e.e.a. hopelijk begrijpelijker.]* Sommige overdrachten die worden geschreven over de continuïteit in de zorg, daar zouden we graag zien dat de afdelingsverpleegkundigen dat zelf doen, samen met de artsen, maar we weten dat daar geen tijd voor is. Liever goed dan slecht, dus dan doen we het liever zelf. Dat is iets wat we dan overnemen. Maar in principe adviseren we alleen.

**01.27**

**I: Oké. En is er altijd één centrale zorgverlener aanwezig?**

R: Hoe bedoel je?

**I: Dus voor de patiënt, vanuit het perspectief van de patiënt. Jij zegt: ik ben meer adviserend. Is er een centraal persoon waar die patiënt zich op richt?**

R: Centraal staat, los van dat de patiënt centraal staat, de hoofdbehandelaar. Die bepaalt het beleid en coördineert alles, onderhoudt contacten met bijvoorbeeld een huisarts. Of als een patiënt in de thuissituatie zit, is de huisarts de hoofdbehandelaar en die onderhoudt contacten met het ziekenhuis. Als je de centrale zorgverlener bedoelt, zoals die in het kwaliteitskader is geformuleerd, die hebben we niet. Het is niet zo dat de patiënt een vast aanspreekpunt heeft, ongeacht zijn verblijfplaats. Dat is wel een wens. Zo is het ook in het kwaliteitskader opgesteld, maar zo ver zijn we nog niet. We proberen wel… Er zijn een paar thuiszorgorganisaties hier in de regio, die heel veel zorg verlenen aan de patiënten van ons, die ook palliatief verpleegkundigen hebben. Die proberen we daar dan ook in te schakelen. Die zou je een beetje als zodanig kunnen zien, maar dat vraagt ook echt in de regio een afstemming op elkaar, van, hoe gaan we daarmee om?

**I: Ja, oké. Je gaf aan de huisarts. Kun je iets meer vertellen over hoe er met die huisarts wordt samengewerkt?**

R: Ons advies is eigenlijk altijd als een patiënt… Ook als wij er niet echt bij betrokken zijn, maar wij bijvoorbeeld in een multidisciplinair overleg aanwezig zijn en er wordt besproken dat de patiënt in een andere ziektefase is gekomen – dat kan zijn dat hij palliatief is geworden, maar dat er nog wel behandelingen zijn, maar het kan ook zijn dat iemand terminaal is geworden, dus nog maar een paar maanden hooguit te leven heeft – dan adviseren we: bespreek het met de huisarts, bel hem op. Het is niet alleen de brief die je stuurt, die best gelezen wordt. Wat wij zeker proberen is dat als we een soort overdracht schrijven voor een patiënt, we contact opnemen met de huisarts. De huisarts kent de patiënt beter, meestal, dan wij. “Hoe kijk je er daar tegenaan? Heb jij nog inzichten die voor ons relevant zijn?” Het afstemmen van adviezen en ideeën, zodat de huisarts daar ook weer mee verder kan. Vaak worden de vijf minuten dat je elkaar even aan de telefoon hebt ook gewaardeerd, want het is niet: het ziekenhuis dicteert aan de huisarts van, je moet dit of dat. Het is proberen om gezamenlijk te kijken, de patiënt in het oog nemend, van: wat is goed, waar kan het beter en hoe kunnen we elkaar helpen daarin?

**I: Ja, oké. Werkt u ook samen met maatschappelijk werk?**

R: Ja. We hebben het palliatief adviesteam in het ziekenhuis. W. en ik zijn de twee verpleegkundigen. Wij komen meestal als eerste bij de patiënt en er zitten vier artsen omheen. Je gaat maar met één arts praten. J. is ook echt kaderarts Palliatieve Zorg. Die benaderen we daardoor ook het meeste. Rondom die kern van zes mensen zit een hele schil met een geestelijk verzorgende, maatschappelijk werker, psycholoog, andere disciplines. Eigenlijk is het hele ziekenhuis de schil, maar er zijn een paar mensen die er gewoon vaker bij betrokken zijn. Die zijn in ieder geval bij de MDO’s aanwezig en bij ons eigen multidisciplinaire overleg. Alle patiënten die wij zien worden wekelijks besproken. Daar zit ook maatschappelijk werk bij. Daar zit niet altijd een psycholoog bij, maar dat is op dit moment nog een logistiek probleem. De verwachting is dat … alles beter maakt, het nieuwe ziekenhuis.

**05.59**

**I: Oké. Ja. Je hebt het over het palliatieve team en daar zit een schil omheen, zoals jij beschrijft. Is die schil daaromheen binnen jullie afdeling anders dan op andere afdelingen, of zijn er andere mensen uit die schil die jullie gebruiken in jullie palliatieve zorg dan bijvoorbeeld bij andere afdelingen?**

R: Wat bedoel je met andere afdelingen?

**I: Laat ik hem iets breder stellen: in de palliatieve zorg, denk jij dan dat jij andere mensen uit die schil nodig hebt dan dat je op andere afdelingen zou hebben in het ziekenhuis? Dus denk jij bijvoorbeeld dat jij meer met maatschappelijk werkers werkt dan op andere afdelingen of denk je dat jij meer gebruik maakt van pastorale hulp of zo dan op andere afdelingen?**

R: Ja, maar dat is meer omdat wij vertrouwder zijn met hen dan dat de noodzaak er meer zou zijn. Natuurlijk hebben mensen in de palliatieve fase, zeker als ze net slecht nieuws hebben gehad, vaak een heel diverse zorgvraag, die je vanuit verschillende disciplines kan invliegen. Soms is er ook nog wel onduidelijkheid van: goh, het is een kwetsbare patiënt die niet lang meer te leven heeft, maar wel al behoorlijk op leeftijd is. Moeten we nou de geriatrieverpleegkundige of de palliatief verpleegkundige inschakelen? Of kan je het met een maatschappelijk werker doen of zijn er juist zingevingsproblemen en hebben we een geestelijk verzorger of misschien wel… Er zijn ook wel raakvlakken vaak. De ene keer pikt die het op, de andere keer pikt een ander het op. We zitten hier in een klein ziekenhuis en het voordeel is gewoon, we kennen elkaar. We hebben elkaars telefoonnummer in het geheugen staan, dus we weten elkaar heel makkelijk te vinden, van: goh, denk even mee, is het nu handiger dat jullie gaan kijken? Daar zit niet echt ook een competentiestrijd, van: hij is vijfenzestig plus, dus hij is voor ons, of, hij gaat dood, dus hij is voor ons. Nee. Nee, ondertussen kennen we elkaar gelukkig goed genoeg om daar een goede weg in te zoeken. En andere afdelingen; wat je wel ziet is dat wij laagdrempelig tijdens een MDO, bijvoorbeeld bij de Chirurgie, zeggen van: goh, maar deze patiënt heeft net twee weken op de IC gelegen, heeft problemen, zou je daar niet maatschappelijk werk inschakelen, want we weten dat dat betere zorg is. Niks palliatiefs, maar op die manier proberen we elkaar ook wel te ondersteunen.

**I: Oké. Oké. Ja, lijkt me fijn die korte lijntjes. Hopelijk is dat in het nieuwe ziekenhuis alsnog zo het geval. Ja.**

R: Ja. Ja. Ik denk dat die lijnen alleen maar korter worden.

**I: Ja. Ja, fijn. Het tweede thema gaat wat meer over het consult zelf, over het communiceren, informeren en de gezamenlijke besluitvorming. Binnen de palliatieve zorg is er natuurlijk aandacht voor meerdere aspecten: het fysieke, psychische, sociale en spirituele welzijn van de patiënt. In hoeverre komen deze verschillende aspecten aan bod in de gesprekken met de patiënt?**

R: Dat is een lastige, want ze zijn alle vier even belangrijk. Misschien is spirituele zingeving de allerbelangrijkste, want heel veel dingen vallen daar overduidelijk onder. Maar het zijn de meest moeilijk te bespreken onderwerpen. We zijn een ziekenhuis, dus we zijn sowieso erg lichamelijk georiënteerd. Alle problemen die we zien zijn voor een heel groot gedeelte lichamelijk: pijn, benauwdheid, misselijkheid, een delier. Ja, sociale problemen, zorgen om kinderen of een overbelaste mantelzorger of iets dergelijks, daar hebben we wel aandacht voor, dat komen we ook wel vaak tegen, maar vaak is er juist in de thuissituatie meer aandacht voor. Dan is het tegen de huisarts, van: goh, wees attent, weet in ieder geval dat die mantelzorger overbelast dreigt te raken, kijk of je daar wat mee wil doen. Psychisch, met angst veel. Veel van onze patiënten zijn bijvoorbeeld bang om te stikken. Nou, daar ga je natuurlijk op in. Maar het zou wel wat gelijkwaardiger mogen. Het gewicht ligt vooral bij het lichamelijke. Daar maken we ons heel mooi van af door te zeggen van, wij doen maar een heel klein stukje van de palliatieve zorg, in het ziekenhuis. Thuiszorg, huisarts of verpleeghuis, hospice; daar gebeurt nog veel meer.

**11.48**

**I: Dus wat meer in de eerste lijn, als ik jou goed begrijp.**

R: Ja. Die overdracht waar ik het net over had, de palliatieve overdracht, is ook een momentbeschrijving. Ga daar alsjeblieft mee aan de haal. Verander naar nieuwe inzichten, nieuwe situaties die ontstaan, en schrap en voeg toe en verander waar je wil en waar je het nodig vindt. En iedereen heeft zijn eigen specialisme/specialiteiten en aandachtsveld of blikveld als je naar deze patiënt kijkt. Het is niet het kunstje van het ziekenhuis of: de verzorging ligt alleen bij de huisarts. Je moet het met elkaar gaan doen. Dat proberen we ook in de preventie juist, in het netwerk Palliatieve Zorg, steeds meer over het voetlicht te krijgen, dat we het met elkaar moeten doen. Dat klinkt allemaal heel mooi. Het is echt nog puzzelen en uitvogelen en onderzoeken hoe je elkaar daarin goed kunt vinden.

**I: Ja, oké. En in die gesprekken met patiënten bijvoorbeeld, brengen jullie dat soort aspecten ook zelf ter sprake?**

R: Ja, we proberen zoveel mogelijk zonder een agenda in het hoofd met de patiënt te praten.

**I: Wat bedoel je met “agenda in het hoofd”.**

R: Zo van: nou, ik moet die overdracht schrijven, dus ik moet even weten of de patiënt pijn heeft en benauwd is, of die angstig is. Hè? Het rijtje. Natuurlijk heb je dat in je hoofd zitten, maar ga eerst maar eens praten en sluit aan bij waar die patiënt mee komt. Vaak hoef je dan niet eens heel veel dingen erbij te vragen, want de patiënt komt zelf met heel veel dingen. Het is meer een kletspraatje dat je gaat houden met de patiënt, zoals je bij de buurman of buurvrouw op de koffie komt, dan dat het een heel formeel gesprek is. Daaruit haal je ook heel veel informatie.

**I: Oké. Dus uit het kletspraatje haal je eigenlijk al heel veel informatie.**

R: Ja. Ja.

**I: Ja, precies. En is dat bij alle patiënten hetzelfde?**

R: Nee, nee. De een staat daar wel voor open en de ander staat daar niet voor open. Ik weet niet of dat ook met de aard van de … (streek in Nederland) te maken heeft. Spreekwoordelijk wat stug en gesloten. Dat valt reuze mee hoor, maar wat je hier wel vaak ziet is: het is gewoon zo. We hebben ook een mevrouw die sinds twee weken, echt twee weken, weet dat ze uitgezaaide kanker heeft. Daarvoor heeft ze nooit klachten gehad. Ja, die heeft niet heel lang meer. Die is daar wonderbaarlijk nuchter over. Ja, ze heeft het allemaal nog niet op een rijtje en verwerkt. Hoe kan dat ook binnen twee weken? Maar ja, dit komt op haar pad en het is niet anders.

**I: Ja. Een bijzondere nuchterheid.**

R: Ja. Ja. En natuurlijk kom je ook hele emotionele mensen tegen. Nou ja, goed, probeer maar… Ik weet niet of ik dat mag zeggen. Wij hebben soms het gevoel dat we wat aanrommelen, alsof er niet een heel doordacht plan achter zit, heel professioneel, van: als dit, dan dat. Het is meer van: oké, kom maar op. Meedenken, meebewegen met de patiënt en kijken of je daarin aangrijpingspunten vindt. Ook omdat de patiënt dan pas… Daarin kun je ondersteunen, als hij zelf met dingen komt. Natuurlijk vragen we wel, maar als we ergens weerstand vinden, dan moet je je afvragen of je dat eenmalig of in die twee contacten die je hebt in een ziekenhuis moet proberen die weerstand te doorbreken of dat je dat aangeeft aan de huisarts, van: daar zit een weerstand, wees erop bedacht.

**16.27**

**I: Ja. Als ik mag inhaken op het aanrommelen; ik zie dat zelf niet zo overigens.**

R: Dank je.

**I: Maar bijvoorbeeld bij mensen die wat lager opgeleid zijn of die niet heel veel lijken te snappen bijvoorbeeld, worden dan die aspecten ook ter sprake gebracht of zijn er daar verschillen?**

R: Daar was ik best zenuwachtig voor.

**I: Waarom, wat bedoel je?**

R: Nou ja, ook omdat ik daar in elk geval geen vooropgezet plan heb. Ieder mens, ongeacht afkomst en opleiding en welke ziekte er ook speelt, heeft zijn eigen problemen en zijn eigen krachten. Daar probeer je een weg in te vinden. Als methode A van benaderen niet werkt, dan probeer je methode B. Juist het koffiedrinkgesprek maakt wel dat dat laagdrempelig genoeg is meestal, voor de meeste mensen, om er een gesprek over te voeren. Door te proberen mee te gaan in de informatie die de patiënt levert, kom je uiteindelijk ook op grote hoogten en filosofische inzichten soms. En soms is het gewoon heel praktisch. Ja.

**I: Dus als ik het mag samenvatten voor mijzelf, is het eigenlijk zo dat het geheel afhankelijk is van hoe de patiënt is en wat de wensen van een patiënt zijn. Als ik jou ook goed begrijp haal jij heel veel uit het – je noemt het kletspraatje, maar volgens mij is het juist heel belangrijk – kletspraatje vooraf, even een beetje laagdrempelig, dat je daar al heel veel uithaalt en dat je op basis daarvan bepaalt of je plan A –wat je net zelf zei – of plan B kiest. Is dat een beetje…**

R: Ja. Plan A en plan B vormen zich tijdens het gesprek wel. Maar ik ben me meestal niet zo bewust van de communicatievaardigheden of de gezondheidsvaardigheden van een patiënt. Daarom noem ik het soms ook wat aanrommelen. Ja, je kijkt naar de patiënt en je merkt het wel. Ja. Het voelt alsof er wat meer uit te halen zou zijn, maar misschien moet je ook zeggen: juist doordat we met verschillende lijnen zoveel samenwerken en wij de patiënt hier vaak maar één of twee keer echt zien; laat het over aan de mensen die de patiënt goed kennen en die daarmee verder kunnen gaan.

**I: Je doet wat je kunt op de momenten dat je zo’n patiënt tegenkomt.**

R: Ja. Je vraagt nogal wat hè, ongeacht wie het is. Mensen zijn kwetsbaar, want ze hebben heel slecht nieuws te horen gekregen. Wij komen daar als hulpverlener binnen en we vragen eigenlijk aan de patiënt alles behalve zijn pincode, alle emotionele dieptes en zwaktes en angsten. Daar zijn wij in geïnteresseerd.

**I: Dat is nogal wat.**

R: Ja.

**20.10**

**I: Ja, dat is het ook. Ja. Precies. Werken jullie met een individueel zorgplan?**

R: Ja. Wat we proberen is om als de patiënt naar huis gaat, als wij denken dat dat meerwaarde heeft, een plan op te stellen waarin we beschrijven: wat is de huidige situatie, wat zijn de bijzonderheden, wat doen we daaraan en wat zijn nou potentiële problemen bij deze patiënt? We kennen de ziekte, we kennen ondertussen een klein beetje de patiënt. Vanuit de ziekte weten we wat er kan gaan gebeuren, aan pijn, ascitesvocht, angst, heftige bloeding. Als je van tevoren weet dat iets kan gaan gebeuren, is het wel zo handig om daar nu alvast een plan van aanpak bij te hebben. Wat we heel vaak zien is dat de patiënt aangeeft van: ik wil thuis leven en uiteindelijk thuis sterven. We weten dat de interventies vooral zijn gericht op comfort en het beheersen van klachten. Aan de ziekte kunnen we vaak niet zo gek veel meer doen. Dus een ziekenhuisopname heeft weinig, misschien zelfs wel gewoon geen meerwaarde voor die patiënt. Die kan veel beter thuis blijven. Dat is ook de wens. Maar de problemen ontstaan om drie uur ’s nachts. Dan komt een vreemde huisarts bij die patiënt. Die heeft nog twee spoedjes waar hij tegelijkertijd moet zijn. Wat is er makkelijker dan insturen? Nou, dan moet je dus een alternatief hebben. Wat proberen we daarmee te doen? Als deze patiënt deze klachten heeft, dit hebben we al geprobeerd, dan kun je dat doen. Voorzichtig formuleren. Zodat in ieder geval de dag kan worden gehaald, de eigen huisarts bij die patiënt langs kan en verder beleid kan gaan afspreken, om te voorkomen dat er dingen gebeuren die die patiënt eigenlijk niet wilde.

**I: Ja, precies. Dan is het wel vastgelegd in ieder geval.**

R: Ja. Ja.

**I: Oké. Ik moet even kijken naar de tijd. Even kijken. Ja, zoals we al besproken hebben: een goede communicatie is de basis van goede zorg natuurlijk, zodat de patiënt goed geïnformeerd is en er sprake kan zijn van gezamenlijke besluitvorming. Zijn er patiënten bij wie jij de communicatie moeilijk vindt?**

R: Ja. Sommige mensen vinden het gewoon niet zo leuk om over de dood te praten of over ziekte. Ik weet niet of jullie bekend zijn met het fenomeen STEM, STerven op je Eigen Manier. Er zijn grofweg vijf manieren waarop Nederlanders omgaan met sterven, lijden, de dood. Ieder heeft zijn eigen sterke en zwakke punten. Bijvoorbeeld de ‘sociale’, het is iemand die zegt van: nou, dat praten over de dood, dat is niet leuk hoor, we gaan koffie drinken. Een karakteristiek voorbeeld uit één van die dingen. Dat is moeilijk. Sommige mensen lijken er niet over te willen praten en hebben hun eigen manier van benaderen nodig. Nou, je moet én accepteren dat mensen er in ieder geval op dat moment niet over willen praten én dat ze hun eigen strategie nodig hebben. Soms heb je die strategie nog niet gevonden.

**I: Oké. Dus als ik het goed begrijp is het wel afhankelijk van de patiënt hoe je je strategie eigenlijk dan aanpast.**

R: Ja. Ja, ik ben ook echt de professional, dus als het goed is, moet ik mij aanpassen aan de patiënt. STEM, één van de belangrijkste dingen die je daaruit leert, denk ik, is: tachtig procent van de mensen die je tegenkomt staat anders in sterven en dood dan jij.

**I: Ja. Dat is wel een mooie uitleg.**

R: Als je dat weet en je weet dat jij de professional bent en de patiënt niet, moet jij je dus aanpassen en kan je niet verwachten dat de patiënt zijn communicatie aanpast. Want ik wil wat bij die patiënt en niet andersom, in eerste instantie.

**24.52**

**I: Ja. Ja. En check jij dan weleens bij de patiënt of ze jou hebben begrepen?**

R: Ja. Dat zit ook in dat kletspraatje.

**I: Dat zit ook in dat beginmoment, ja.**

R: Nou ja, het klinkt als beginmoment, maar eigenlijk is het een uur lang koffie drinken. En natuurlijk vraag je heel gericht dingen. Als iemand moeite heeft om ergens mee om te gaan, dan klets je daar niet over. Dan heb je het daar ook echt over, maar…

**I: Oké. Ja. In hoeverre is er volgens jou sprake van gezamenlijke besluitvorming met patiënten in de palliatieve fase?**

R: Dat is een moeilijke, omdat de palliatieve fase ook hele verschillende betekenissen heeft. Formeel is het: er is geen genezing meer mogelijk, maar iemand kan van dagen tot maanden en soms wel jaren een levensverwachting hebben. Voor sommige artsen met name – daar zijn we afhankelijk van bij het in consult vragen – zijn het echt de laatste dagen: de patiënt gaat sterven, haal het PAT erbij, het palliatief adviesteam. Voor sommigen is het dan: er zijn geen ziektegerichte interventies meer mogelijk, het is nu puur symptoomgericht, dat is palliatief. Als er ziektegerichte interventies mogelijk zijn, proberen we zoveel mogelijk de zorg bij bijvoorbeeld de oncologieverpleegkundige te houden. Die kent die patiënt al heel erg goed. Maar als het puur om symptomen gaat, dan kunnen wij daar veel meer in. Oncologische behandelingen, ik heb er geen kaas van gegeten. Dus het shared decision… Het gaat wel van: goh, u heeft heel veel pijn, u heeft het heel benauwd, morfine zou een hele goede optie zijn, dat zou u daar echt bij kunnen helpen. Heel veel mensen hebben daar bezwaren tegen, ideeën bij. Ja, uiteindelijk bepaalt de patiënt of hij dat wil, waarbij wij wel proberen aan te geven waarom iets goed is en te kijken van: waar zit dan de weerstand? Ja. Maar ja, of dat nou echt shared decision making is… *[Aanvullend. We maken gebruik van het Utrecht Symptoom Dagboek (USD). Een van de items daarin is de mogelijkheid voor de patiënt om zelf aan te geven waar zijn/haar prioriteit van (be)handeling ligt. En in de methodiek van het palliatief redeneren zit telkens de terugkoppeling naar de patiënt of de conclusies en beleidsvoorstellen overeenstemmen met zijn/haar ideeën.]*

**I: Oké. En denk je dat het beter zou zijn als in grotere mate gezamenlijke besluitvorming plaats zou vinden?**

R: Ja. Maar ik denk al in een veel vroeger stadium, waar je het hebt over: als een arts zegt van, nou, goh, dit is de situatie, je kunt behandeling A kiezen, je kunt behandeling B kiezen en oh ja, je kunt ook niets doen. Als je het op die manier brengt, dan is er geen keuze meer, alleen tussen A en B, want niets doen… Als je dat op een hele andere manier brengt, aangeeft dat we hem blijven volgen, klachten draaglijk houden, op alle fronten proberen erbij te zijn en te helpen, is het een hele andere keuze geworden. Daar valt nog wel veel te winnen. Maar ik vrees dat dat overal in Nederland zo is.

**I: Ja. Dat denk ik ook, ja. Zou jij een voorbeeld kunnen noemen van een situatie waarbij er heel duidelijk sprake was van gezamenlijke besluitvorming? Je gaf eigenlijk net een voorbeeld, had ik het idee, waarbij dat juist niet echt het geval was, maar heb je ook een voorbeeld waar dat wel…**

R: Ja, ik weet niet of het echt onder gezamenlijke besluitvorming valt, maar wat wij heel vaak doen is tegen mensen in de laatste maanden van hun leven, als ze hier zijn, zeggen van: goh, u gaat nou naar huis toe, dan gaat het straks slechter, wat wilt u dan? Wilt u dan naar het ziekenhuis – en soms leg ik dan ook nog even voor de duidelijkheid uit: met de ambulance, op de Spoedeisende Hulp, onderzoeken en dan opgenomen worden – of zegt u: nee, dan wil ik thuis zijn en dan wil ik dat de huisarts het mij zo makkelijk mogelijk maakt. Nou, negenennegentig van de honderd mensen kiest voor die laatste optie. Dat is voor mijn gevoel het dichtste wat erbij komt, op dit moment, wat me zo te binnen schiet.

**29.29**

**I: Ja, oké. Wat zou er beter kunnen? Wat zou ervoor nodig zijn om die gezamenlijke besluitvorming te verbeteren, denk je? Je gaf net zelf aan van: nou, ik denk eigenlijk dat dat wel beter zou kunnen. Hoe zou dat dan kunnen?**

R: Ja, dat is een moeilijke vraag. Nou, het zijn gedachtespinsels een beetje, zo moet je het zien, want het is niet vastomlijnd.

**I: Graag.**

R: Dat we sowieso accepteren dat niets doen – en dat moet je nog beter verwoorden – een serieuze optie is voor mensen, naast de andere opties aan kuren en zoveelste lijn, chirurgische ingrepen, dat dat serieus is. Daarin moet je patiënten ondersteunen, dat er een serieuze andere optie is. Je zult als zorgverlener dat ook zelf zo moeten ervaren voordat je het kunt uitdragen, anders dan blijf je zeggen van: oh ja, we kunnen ook niks doen. In het kader van palliatieve zorg kan dat denk ik een belangrijke ondersteuning zijn voor shared decision making.

**I: Oké. Dus toch die; wat je als voorbeeld gaf, van, je kunt ook niks doen, dat je dat toch iets meer gewicht geeft of dat je die een beetje op gelijke waarde stelt. Vat ik jou dan zo goed samen?**

R: Ja. Ja. Dat mensen ook voelen dat het niets doen niet niets doen is, maar dat er meer aandacht is voor die hele mens, met die vier dimensies die je net ook behandelde.

**I: Ja, precies. Oké. Ik heb een aantal kaartjes. Daarop staat: wilsonbekwaamheid en levenseindebeslissingen. Ik heb er ook al een aantal gehoord in het gesprek. Behandelafspraken. Crisissituaties. Levensbeschouwing en culturele achtergrond. Ziekenhuisopname. En deze is heel lang: wettelijke vertegenwoordiging in een situatie van acute verslechtering. Nu aan jou de vraag of je een rangorde erin kan aanbrengen, in deze kaartjes, in hoeverre deze aan bod komen tijdens het consult, dus eigenlijk in hoeverre deze onderwerpen besproken worden in het consult.**

R: Prima. De plaats van zorgen en sterven is sowieso een hele belangrijke. Daaraan gekoppeld direct is ziekenhuisopname.

**I: Die noemde je daarstraks al hè? Ja.**

R: Ja. Omdat een ziekenhuisopname vaak de wens om thuis te sterven doorbreekt. Dat is direct ook gekoppeld aan crisissituaties, want daarvoor schrijven we nou juist dat plan, om dit te voorkomen. Wilsonbekwaamheid komt niet heel vaak aan bod. Levensbeschouwing en culturele achtergrond. Wij hebben hier een relatief homogene groepering qua culturele achtergrond. De Molukkers, om het zo maar even te noemen, dat is een groep die hier wel zit. Verder niet heel veel bijzondere culturele groepen. Vooral blanken. Als je het op die manier kan indelen.

**I: Ja. Jullie hebben vooral te maken met de nuchtere … (streek in Nederland).**

R: Ja. Waarbij die nuchterheid nogal ter discussie staat hoor. Wettelijke vertegenwoordiging. Ja, dat is eigenlijk meteen ook gekoppeld aan die wilsonbekwaamheid. De behandelafspraak, dat is in ieder geval iets wat ze vaak met de arts bespreken, waarbij we adviseren van: ga daarover praten. Standaard wordt het op de Spoedeisende Hulp gesteld, maar de meeste mensen hebben zoiets van: ik voel me hartstikke ziek, dus help me, hoezo ga je dan iets niet doen, natuurlijk moet je wat doen. Ik denk dat dit wel de belangrijkste taak van wat wij doen is. Dit zit er in adviserende zin wel bij. Hier hebben we wel aandacht voor, zeker als we het over euthanasie en sedatie hebben. Vaak is het wel duidelijk wie er in de naastengroep belangrijk zijn en beslissingen nemen. Vaak hebben mensen dat zelf wel aangewezen. Dan is het geen wettelijke vertegenwoordiging, maar dan is het de dochter die ook in de zorg werkt of de oudste zoon mag daar iets over zeggen. Zo ongeveer.

**35.24**

**I: Oké. Dus bovenaan de plaats van zorg en sterven, ziekenhuisopnames en crisissituaties. Eén trapje lager staat links de behandelafspraak. Dat is ook onderdeel van wat jullie…**

R: Ja, soms is het in een overleg met een arts van: goh, het is lastig. Nou ja, dan kunnen wij in ieder geval alvast een begin maken met het gesprek. Mensen moeten vaak ook tijd hebben om na te denken.

**I: Ja, oké. Eén trapje lager zitten wilsbekwaamheid, levenseindebeslissingen en wettelijke vertegenwoordiging in een situatie van acute verslechtering. En helemaal onderaan staat levensbeschouwing en culturele achtergrond en dat komt, als ik jou even mag samenvatten, eigenlijk door de locatie waar we zitten, eigenlijk door het homogene…**

R: Ja. En natuurlijk, want die vier dimensies met… Met name zingeving. Daar gaat dit ook over. Het lijkt misschien dat we dat niet belangrijk vinden, maar daar is wel aandacht voor, alleen minder expliciet.

**I: Ja. Ja, duidelijk. Het laatste thema. Dat gaat over de beperkte gezondheidsvaardigheden. Heb je weleens gehoord van die term, beperkte gezondheidsvaardigheden?**

R: Ja.

**I: En wat betekent dat volgens jou?**

R: Oh. Nou, dat is ook een moeilijke, want dat blijkt ook wel uit de manier waarop voor het onderzoek mensen worden geïncludeerd. Dat zijn eigenlijk afgeleiden, waarbij je je zelfs de vraag kunt stellen of ze ermee te maken hebben. Dat vind ik een hele lastige, om daar iets zinnigs over te zeggen. Er komen heel veel vragen bij me op. “Ik heb fors overgewicht.” Daar kun je ook van zeggen: dat is een beperkte gezondheidsvaardigheid. Je kunt het hebben over mensen die niet accepteren dat ze ziek zijn, die dat niet lijken te begrijpen of die gewoon door blijven roken. Ik vind dat heel erg lastig.

**37.44**

**I: Dat begrijp ik. Ik denk dat wij meer op de middelste categorie zitten. Dus het gaat bij ons meer om de vaardigheden van mensen om de informatie over gezondheid te krijgen, te begrijpen en toe te passen. Bijvoorbeeld, wat staat er nou op dat medicijnpotje? Het is eigenlijk een hele functionele definitie. Dat is eigenlijk de definitie die wij hanteren.**

R: Het probleem is denk ik, zeker bij levensbedreigende aandoeningen, dat de uitleg heel rationeel is, terwijl de reactie bij de mens, de patiënt, juist niet rationeel is, puur emotioneel vaak. En dan nog zit ik te denken, bijvoorbeeld ook met STEM in gedachten: iemand die het gewoon moeilijk vindt om over de dood en ziek zijn te praten, die zich daar gewoon niet fijn bij voelt en dat wegduwt, is dat een beperkte gezondheidsvaardigheid of is dat een coping mechanisme? Nee. Dat laat ik aan jullie over. Adviseer ons daar over een paar jaar maar over.

**I: Oké. Mijn volgende vraag zou zijn of jij zo’n patiënt zou herkennen in de praktijk en hoe vaak je daarmee te maken hebt.**

R: Nou ja, gezien de lagere sociaaleconomische status in deze regio zal ik er best vaak mee te maken hebben. Maar of het relevant is… Weet je? Je moet gewoon roeien met de riemen die je hebt. Het is uiteindelijk de patiënt, de mens, die al dan niet met mensen om zich heen ervoor kiest om een bepaalde behandeling wel of niet te doen. Ja. Daarin probeer je wel af te stemmen, maar hoe je dat… Nou ja, daar zit dan weer dat aanrommelen. Ik heb er niet zo één, twee, drie strategieën bij. Dat is ook één van de redenen waarom wij aan dit onderzoek meedoen. We vinden het wel heel belangrijk. Dat zeggen we in ons communicatieadvies, daar ga je met R. over praten. Maar we hebben geen idee hoe we dat dan precies moeten operationaliseren. Dat is gewoon lastig. We proberen handvatten te vinden.

**I: Oké. En ervaar jij dan weleens barrières in de zorg daardoor?**

R: Ja. Er zijn ook patiënten waar ik niks mee kan.

**I: Oké. En dat komt dan bijvoorbeeld door die lagere gezondheidsvaardigheden? Als we de definitie even pakken zo functioneel als ik hem beschrijf. Dat komt weleens voor?**

R: Ja. Ja. En soms moet je dan zeggen van: goh, collega, ga jij eens, misschien heb jij wel een ingang. En soms is het gewoon accepteren dat dat er niet is en dan is het terugverwijzen of bellen met de huisarts en zeggen, kort door de bocht, van: ik kan niks met die patiënt. Dat wordt meestal dan wel herkend. Dan moet je toch weer kijken van: oké… De huisarts dan, zeg ik, met het nodige gemak, van: oké, hoe gaan we dan toch goede zorg leveren? Soms is goede zorg heel paternalistisch, omdat de dokter het zegt. Maar of dat nou door die gezondheidsvaardigheden… Waarschijnlijk wel, maar het is tricky, omdat je daarmee wil een stigma, of een stempel op iemand drukt alsof die dom is, alsof die minderwaardig zou zijn. Gevoelsmatig roept dat bij mij dan even op. Dat bedoelen we niet.

**I: Nee. Nee, precies. Oké. Ja. Wat zou volgens jou nodig zijn om de zorg beter te laten aansluiten bij patiënten met beperkte gezondheidsvaardigheden?**

R: Tijd. Tijd nemen voor mensen, zodat je dat kletspraatje, dat koffiepraatje dat wij voeren met mensen ook kunt voeren. Alleen als jij als dokter bijvoorbeeld tien minuten per patiënt hebt op je polikliniek en je loopt al wat uit, dan heb je er geen aandacht voor en als je er wel aandacht voor hebt, dan loop je gigantisch uit en zijn andere patiënten er de dupe van. Ik denk tijd, maar die tijd moet je wel nuttig gebruiken door te luisteren, door af te stemmen.

**42.59**

**I: Ja. Dus eigenlijk vooral tijd, meer tijd voor dat soort patiënten. Oké. Duidelijk. Ja, de volgende vragen gaan eigenlijk over de ziekenhuisorganisatie. Die ga ik denk ik aan R. voorleggen…**

R: Ik kan er wel wat over zeggen. Ik denk dat J. daar ook wel veel over kan vertellen, de kaderarts Palliatieve Zorg.

**I: Oké, dat is een goede. Ik heb nu al gemerkt dat ik bij J. even wat gas moet geven bij de eerdere vragen. Heb je verder nog aanvullingen op dit interview, iets wat je zou willen toevoegen?**

R: Nee. Nou, we hebben een beschrijving gedaan van hoe wij mensen hebben geïncludeerd, of in ieder geval hoe wij tot het aanbod zijn gekomen waarvan W. heeft moeten zeggen “ja” of “nee”. Eén van de dingen die opvalt is dat de mens die je het meest gunt om hieraan mee te doen, omdat je denkt dat die echt het meeste tot de doelgroep behoort, de eerste is die huiverig is om mee te doen, ook vooral omdat ze bang zijn dat de relatie met een arts in gevaar komt, terwijl je wel uitlegt dat de arts zelf meedoet hieraan.

**I: Ja, daar gaat het om.**

R: Die bang zijn dat jij al weet wat de uitslag is van het onderzoek wat zij nog moeten ondergaan, wat in dat gesprek tussen arts en patiënt wordt medegedeeld, dus het tussenliggende onderzoek, dat jij dat al hebt en dat je hen daarom benadert. Of dat ze zich daar vreselijk zorgen om maken van: ik kom op de video. Hoe goed je dat ook probeert uit te leggen, dat is vreselijk moeilijk.

**I: Ja, maar we vragen ook nogal wat. Het is ook nogal een gesprek zeg, waar we even binnenkomen. Maar dat eerste aspect wat je aangaf, dat horen wij ook wel terug van W., die relatie met die arts, die hadden wij niet voorzien.**

R: Maar het zegt in ieder geval dat wij in onze uitleg, waar we mee bezig zijn en waarom we deze mensen benaderen, misschien toch nog wel weer andere woorden moeten gebruiken, andere manieren moeten vinden dan dat we tot op heden hebben gedaan.

**I: Ja. Jij zegt: kijk daar nog eens goed naar, naar dat inclusiepraatje.**

R: Nou, gevoelsmatig: ja, ja.

**I: Dat is een hele goede tip, dat moeten we zeker doen dan.**

R: Waarbij ik heel duidelijk het gevoel heb dat mensen mij niet hebben begrepen. Dat ligt dan weer aan mij, dat ik blijkbaar… Maar goed, dat ik dus op een andere manier… Ik wil wat van hen, dat ze meedoen, dat is waarom we hen vragen. Als zij om andere redenen dan voor mij valide redenen zijn, dat niet willen: prima, maar dan ben ik niet duidelijk genoeg geweest in de uitleg, omdat zij blijkbaar andere associaties krijgen.

**46.31**

**I: Ja, oké. Ja, duidelijk. En ook aan het interview zelf heb je niks toe te voegen hè?**

R: Nee.

**I: Nee. We gaan het uitwerken, dit interview. Dat krijg je dan, als je dat wilt, in transcriptvorm nog even terug, dus dan kun je er nog even doorheen lezen en dan kun je zeggen van: nou, dit bedoelde ik eigenlijk zo, hier wil ik eigenlijk meer dit en dat, zus en zo. Wat je net aangaf over die dr. J, dat hoeft er niet in, maar bijvoorbeeld hè. Ik weet niet of je dat wil?**

R: Ja, dat is wel handig, denk ik. Ik heb vaak mondeling meer woorden nodig dan als ik er een poosje over heb nagedacht en als ik het schriftelijk kan weergeven.

**I: Oh zo, oké. Nou, voor mij was het heel duidelijk.**

R: Oké. Nou, dat is fijn om te weten. Dank je.

**[48.14 – einde opname]**

Z3a03

I: interviewer

R: respondent

**I: Dank je wel. Even kijken. Ja, ik wil dus graag vragen gaan stellen en die gaan over jouw rol als zorgverlener in de palliatieve zorg, over communicatie, over patiënten zelf en over de manier waarop het ziekenhuis georganiseerd is. Nou, dat laatste stukje, over de organisatie van het ziekenhuis, is wat meer voor bijvoorbeeld R. die ik heb gesproken, dus die sla ik over. Het gaat dus eigenlijk voornamelijk over jouw rol in de palliatieve zorg, communicatie en de patiënt zelf.**

R: Oké.

**I: Zijn er tot dusverre nog vragen?**

R: Nee.

**I: Nee? Oké. Anders hoor ik dat graag natuurlijk.**

R: Ja. Ja.

**01.05**

**I: Jouw rol als zorgverlener in de palliatieve zorg: wat is jouw rol in de zorg voor patiënten in de palliatieve fase?**

R: Het is zo dat ik verpleegkundig specialist Oncologie ben, dus dat betekent dat als mensen een oncologische aandoening hebben en bij de oncoloog een diagnose hebben gekregen of bij de longarts, dat kan ook, of bij de hematoloog, ze daarna bij ons komen om nadere uitleg te krijgen over het behandelvoorstel dat is meegegeven door de medisch specialist. Dan is het onze rol om uit te leggen wat dat behandelvoorstel nou precies inhoudt, wat de werking zal zijn, wat de bijwerkingen zullen zijn en als ze orale medicatie moeten krijgen, hoe dat precies moet, maar ook vooral is het de bedoeling om in dat consult te achterhalen wie je eigenlijk voor je hebt. Wie is die patiënt in de context, hoe ziet die context eruit, hoe is de draagkracht?

**I: De draagkracht?**

R: De draagkracht van de patiënt en de sociale kaart om de patiënt. Hebben wij het idee dat er misschien toch nog aanvullende behandeling zou moeten komen op die standaardbehandeling of kunnen we ervan uitgaan dat de patiënt het allemaal goed begrijpt en dat hij dat zelf met zijn eigen netwerk kan uitvoeren, wat wij voorstellen. En ook wel om een beeld te krijgen van hoe die patiënt er zelf in staat, hoe die tegenover zijn palliatieve diagnose staat en tegenover het behandelvoorstel. Dat is wel een beetje een zoekproces, om dat goed af te stemmen met de medisch specialist. We werken met best wel veel medisch specialisten samen. Voorheen was dat anders. Toen werkten we met maar enkele medisch specialisten samen, zodat je heel gauw goed wist hoe de dokter dacht en dan kon je daar goed op aansluiten. We merken wel dat we dat nu weer een beetje opnieuw moeten uitvinden, met de dokters samen.

**I: Oké. Omdat er nu dus meerdere medisch specialisten zijn.**

R: Ja. Ja. Je ziet eigenlijk, zo is wel onze visie als groep, dat de medisch specialist meer focust op het medische deel, het technische stuk en dat wij meer focussen op de integratie van zowel het medische stuk als de verpleegkundige/de care-kant en wij dus ook de patiënt in zijn totaliteit wat meer meenemen. Nou, dat is soms even zoeken, hoe je dat doet en of je dat meteen in één gesprek altijd boven tafel kijkt. Dat zijn natuurlijk wel processen. Je hebt ook weleens dat je in het eerste gesprek denkt van: nou, ik geloof niet dat ik er nu al iets over mag zeggen van de patiënt. Dan probeer je dat op een later tijdstip in het proces te doen.

**I: Oké. Dus als ik het goed begrijp hebben ze eerst het gesprek met de arts en vervolgens met jou.**

R: Ja.

**I: Ik probeer voor mezelf samen te vatten wat je hebt gezegd. Jij gaat dan eigenlijk ook meer kijken naar de schil om de patiënt heen, naar de sociale kant enzovoort.**

R: Ja. Dat probeer ik te doen. Sommige artsen hebben dat ook al wel voor een deel gedaan, maar dat is niet altijd zo. Verder is het zo met die palliatieve trajecten dat de patiënten in het kader van die behandeling gecontroleerd worden. Dat gebeurt om en om, de ene keer bij de medisch specialist en de andere keer bij ons. Dus je ziet hen in een proces vaker. Het kan ook zo zijn dat als iemand een behandeling heeft afgesloten, hij een tijdlang pauze heeft van een behandeling en opnieuw weer moet starten; dat zijn eigenlijk de ijkpunten waarop je opnieuw dat gesprek zou moeten aangaan, van: goh, hoe staat u er op dit moment tegenover? Dat loopt soms goed en soms wat minder goed. Dat is heel wisselend. Een uitdaging.

**I: Ja. Ja, daar ga ik zo meteen even op doorvragen, als dat goed is.**

R: Ja.

**05.07**

**I: En is er altijd één centrale zorgverlener aanwezig voor zo’n patiënt?**

R: Een aanspreekpunt bedoel je?

**I: Ja.**

R: Ja. Ja. Die is er altijd. Ja.

**I: Oké. En in dit geval zou dat dan zijn? De arts?**

R: Nee, ik en mijn collega’s zijn eigenlijk het… We zijn met zijn drieën. Eén van ons is dan het aanspreekpunt. Dus als de patiënt vragen heeft, dan belt hij eerst ons. Dan proberen wij dat op te lossen. Dat lukt in veel gevallen. Als het niet lukt, dan kunnen wij de arts natuurlijk raadplegen. Soms moet iemand opgenomen worden omdat het niet goed gaat thuis. Of soms is met een uitleg iets goed te verhelpen. Soms moet je bijvoorbeeld een interventie doen, waardoor iemand toch gewoon thuis kan blijven en zijn vraag goed beantwoord is.

**I: Oké. Hoe werkt u samen met de huisarts?**

R: Als de patiënt onder de hoede komt van de huisarts, als je het hebt over palliatieve patiënten in de allerlaatste fase, als geen actieve behandeling meer plaatsvindt, dan wordt de zorg overgedragen aan de huisarts. Die wordt daarover dan geïnformeerd, over hoe ver we zijn gekomen, wat van de huisarts wordt verwacht. Dan overleg je dat en stem je dat af. Het is ook wel zo trouwens dat patiënten die een goede band hebben met hun huisarts tijdens die oncologische behandeling ook wel tussentijds ruggenspraak hebben met de huisarts.

**I: Oké. Dus eigenlijk is er vaak...**

R: Ja. Ook weleens bij calamiteiten. Meestal bellen de patiënten ons, maar een enkele keer ook de huisarts. En ook heeft de huisarts wel de rol om ook eens mee te denken. Er zijn ook patiënten die dat fijn vinden, om het behandelvoorstel dat ze hier hebben gekregen ook eens met de huisarts te bespreken, zo van: wat vindt mijn huisarts er eigenlijk van? Dat ze zo tot het besluit komen dat ze willen. Maar dat is niet bij iedere patiënt zo.

**I: Oké. En zijn er meer… Bijvoorbeeld de huisarts. Zijn er meer maatschappelijke… Werkt u bijvoorbeeld samen met maatschappelijk werk?**

R: Ja. Er is hier een multidisciplinair team. Dat bestaat voor een deel natuurlijk uit medici, maar er is ook wel een multidisciplinair paramedisch team, waar een psycholoog, een maatschappelijk werker en een geestelijk verzorger in participeren. Als wij inderdaad het idee hebben dat het van meerwaarde is, dan hebben we zeker [door elkaar, iemand neemt de telefoon aan en praat erdoorheen] of we overleggen met hen. Ja.

**I: Oké. En ik had nog een vraag: u beschreef net het team, dat jullie met zijn drieën zijn. Even voor de duidelijkheid, bedoel je dan de arts? Wat is de samenstelling van het team?**

R: Er zijn eigenlijk drie, nee, vier verpleegkundige oncologische teams, zou je kunnen zeggen. Dat zijn de collega’s van de Urologie, van de Colon-care, van de Mammacare en wij zijn van de Medische Oncologie. En wij zijn dan als Medische Oncologie met zijn drieën. Wij beschouwen onszelf als verpleegkundig specialisten en consulenten Oncologie als één team. We zien niet altijd dezelfde patiënten, maar hebben wel heel erg veel overlappende werkzaamheden en dingen die overeenkomen, waardoor je kan sparren over hoe je dingen aanpakt. Ja.

**08.35**

**I: Oké. Duidelijk. Ik wilde graag naar thema twee. Dat gaat over communiceren, informeren en gezamenlijke besluitvorming. Binnen de palliatieve zorg is aandacht van belang voor meerdere aspecten: het fysieke, psychische, sociale en spirituele welzijn van de patiënt. Ik vroeg mij af in hoeverre deze verschillende aspecten aan bod komen in uw gesprekken met de patiënt.**

R: Ik denk, als je dat zo opnoemt, dat het spirituele aspect het minste aan bod komt en al het andere wel. Dat wil niet zeggen dat het niet aan bod komt, maar dat is even zoeken, naar: wie heb je voor je en op welke manier willen mensen daar op ingaan? We proberen dat wel, niet zozeer heel hoogdravend, maar heel praktisch in de zin van: goh, wat betekent het voor u om deze diagnose te hebben, wat is voor u de drijfveer om aan deze behandeling te beginnen, wat is belangrijk voor u op dit moment? Dat soort vragen. Ja.

**I: Ja. Oké. Dus jullie gaan er eigenlijk algemeen in en afhankelijk van de patiënt komen die dingen ter sprake.**

R: Ja. Dat is ook afhankelijk van hoe mensen daar al over hebben nagedacht en ook afhankelijk van of en hoe mensen daarover op dat moment met ons willen spreken. Soms zijn mensen daar… Ik had vanochtend nog iemand die heel duidelijk zei… Die was net begonnen aan de volgende lijn in het palliatieve traject en die was heel duidelijk: daar wil ik het nu echt nog niet over hebben, ik heb het nog niet zo ver.

**I: Oké. Dus die dingen brengen jullie zelf wel ter sprake soms.**

R: Ja. Ja. Ja. Eigenlijk proberen we dat altijd te doen bij de start en ook bij de overgang van wat ik dan noem de ene behandellijn naar de andere behandellijn. Dat doen we de laatste tijd steeds bewuster.

**I: Sorry. Bedoel je dan van bijvoorbeeld curatief naar palliatief, van die lijnen?**

R: Nee. Van palliatief lijn één naar palliatief lijn twee. Als je een behandeling hebt gehad, heb je daar soms een tijdlang rendement van. Dan kan het zo zijn dat de patiënt opnieuw een toename van ziekte heeft en dat je dan kiest voor een volgende behandellijn, die wel weer gaat werken. Althans, dat is dan de hoop en de verwachting. Dat zijn dan weer van die kruispunten waarop je dit wel weer ter sprake brengt.

**I: Ja. Dat is dan zo’n moment waarop je de patiënt weer spreekt.**

R: Ja. En tegelijkertijd moet ik er ook eerlijk bij zeggen dat dit wel een aandachtspunt is waarvan we ons bewust zijn dat het niet altijd zo gemakkelijk is en dat je als team, als groot team, zoals ik net heb geschetst…

**I: Ja. Alle verplegingen dus bij elkaar.**

R: Ja. Dat we wel bezig zijn om te kijken hoe we dit nog beter kunnen doen en ook eenduidig. Soms is dat best lastig. Hoe doe je dat precies? Daar zijn we wel over aan het nadenken en in gesprek. We hebben ook vorige week toevallig nog van J., de palliatieve collega’s, een cursus gehad om te horen ook van hen, van: wat is er allemaal mogelijk op dat vlak en hoe kunnen we elkaar daarin ook vinden qua ondersteuning? Ja. En we sparren er ook wel met elkaar over, zo van: ik heb het zo gedaan, hoe had jij dat nou gedaan? Ja.

**I: Oké. Die vier aspecten die ik net noemde, om die zelf ter sprake te brengen; gebeurt dat ook bij mensen die laag zijn opgeleid of die niet zo heel veel lijken te snappen? Hoe werkt dat dan?**

R: Ja, dat kan wel, maar dan moet je een andere taal spreken en een andere toon aanslaan. Het is zelfs soms zo dat mensen die laaggeletterd zijn in sociaal opzicht heel hecht zijn. Dat is niet altijd zo natuurlijk, maar dat vind ik in deze regio ook wel, dat het een vanzelfsprekendheid is dat je voor elkaar zorgt.

**I: Op die manier.**

R: Dat mensen er ook wel over hebben nagedacht hoe ze dat dan willen. Dat is dan op een ander level wel van belang om boven tafel te krijgen.

**12.52**

**I: Oké. Mijn volgende vraag zou zijn: in welke fase van het consult zou dat dan zijn?**

R: Dat is eigenlijk ontzettend verschillend. Soms mag je er niet aan komen, tenminste, dat voel je dan zo, of dat echt zo is… Soms probeer je dat echt te expliciteren. Vanmorgen mocht ik er echt niet aan komen. Dat is heel duidelijk aangegeven. Soms denk je: dat is nu niet het goede moment. Dan bederf je het hè? Dat blijkt ook wel uit onderzoek, dat als je de verkeerde timing hebt bij dit soort zaken, je het daarna misschien wel voor altijd hebt verbruid. Dus je moet dat wel onderzoeken. Dat proberen we wel te doen. Soms mislukt dat, maar meestal gaat dat goed. Dat je dan toch denkt van: vandaag niet, maar de volgende keer probeer ik het toch weer in gang te krijgen, om het hierover te hebben.

**I: Oké. En hoe vraagt u er dan naar?**

R: Dan kom ik erop terug. Dan zeg ik iets van: de volgende keer benoemde u dat u toch wel heel erg ziek bent geweest van de kuur. Wat kunnen we nu nog doen om dat aan te passen? Hoe staat u er nu tegenover? Dat hangt een beetje af van de situatie, hoe ik dat dan probeer te brengen. Ja. Dus dat is best een uitdaging. En het shared decision making, wat je net even noemde, vind ik ook wel een uitdaging. We proberen dat ook wel op een hoger niveau in de organisatie nog wat beter op de kaart te krijgen door ook heel goed te kijken naar ethische besluitvorming. Kunnen we daar nog dingen in verbeteren en kunnen we daar ook met elkaar de neuzen dezelfde kant op krijgen? Zien we onszelf wel als een team? De dokter en wij, zijn wij wel een gesloten cirkel? Waar de een begonnen is, zou de ander mooi verder kunnen gaan als je elkaars informatie gewoon goed kan lezen, dat die cirkel rondkomt. Je ziet wel dat dat steeds meer ontstaat, dat wij er bewust aan werken om die cirkel goed rond te krijgen.

**I: Kun je daar iets meer over vertellen? Wat bedoel je dan precies?**

R: We hebben via het palliatieve adviesteam aan de ethische commissie een voorstel voorgelegd om in de organisatie een pilot te gaan doen over hoe je achteraf kunt zien of je besluitvorming wel op de goede manier is gedaan. Met behulp van een instrument wat dan Ethiek Light heet, wat dan niet helemaal gaat volgens het echte ethische gesprek... Ik ben nou even het woord kwijt. Het morele beraad, daar zat ik naar te zoeken. Dat is best een groot instrument, waarin je ook echt een formele gespreksleider nodig hebt die alle kanten van de medaille kent en goed is ingevoerd in dat soort gespreksvoering. Die heb je niet altijd, zeker niet ’s avonds, ’s nachts of in het weekend. Soms komen dan wel die vraagstukken op. Dus eigenlijk zouden we het heel mooi vinden als in deze organisatie alle afdelingen die met dit soort dingen te maken hebben zelf in staat zouden zijn met een aantal korte vragen de casus goed door te lopen, om te kunnen achterhalen van: goh, is hier nu aan alle zorgvuldigheidscriteria voldaan? Dat is bij de ethische commissie goed ontvangen. Nu is de vervolgstap dat we in dat palliatieve adviesteam daar een beetje mee aan het oefenen zijn om te kijken van: hoe doe je dat dan in teams? Als we in … zitten (locatie ziekenhuis), het is straks eerst een groot project om daar goed te komen en te landen, dan zullen we daarmee verder gaan. Dan kijken we of we een team kunnen vinden dat zin heeft om dat als pilot te doen. Ja. Dus het is wel een onderwerp wat leeft, in ieder geval in de verpleegkundige beroepsgroep. Ja. Om te kijken hoe we hier niet alleen individueel, maar ook als groep, beter in kunnen worden.

**17.11**

**I: Ja. Ja. Want in het algemeen gezien, die gezamenlijke besluitvorming, in hoeverre is er daar sprake van bij patiënten in de palliatieve fase?**

R: Ik denk dat dat iets is wat groeiend is. Ik denk dat de bewustwording van alle behandelaars steeds groter wordt op dit gebied, dat het een must is. Ik denk dat het voor sommigen even zoeken is, van: hoe doe je dat dan? Dat gebeurt ook wisselend. Als je het hebt over gezamenlijke besluitvorming, dan hoor je ook eigenlijk vooral naar die context te kijken. Dan hoor de je de behandelmogelijkheden die je op de plank hebt liggen voor de patiënt al een beetje in die context te plaatsen, door samen met die patiënt te kijken van: wat zou in uw situatie, op dit moment, in uw omstandigheden, met de wensen die u hebt voor de toekomst, het beste zijn om te doen? Dan heb je misschien de keuze uit drie behandelopties, maar dan moet je ook de optie niets doen, dat wil zeggen niet actief iets doen, meenemen, waarbij je het begrip best supportive care zou moeten uitleggen, dat je wel de beste ondersteunende zorg geeft. Het is niet zo dat we dan helemaal niets meer doen voor iemand, maar dat we wel de ondersteunende zorg bieden om alle symptomen die er zijn te bestrijden en ook te zorgen dat iemand van dat moment tot het allerlaatst een goed traject krijgt. Samen met de huisarts, daar kom je dan gauw op. En samen met de palliatieve verpleegkundigen in de thuissituatie. Daar kan zeker nog wel iets in verbeterd worden, denk ik. Maar ook dat is iets wat we met elkaar moeten oppakken, wat een groeimodel zal zijn, zoals dat in heel Nederland nu heel erg actueel is. Ja.

**I: Ja. Oké. En is dat voor alle patiënten hetzelfde?**

R: Wat bedoel je daarmee?

**I: Of die gezamenlijke besluitvorming bij die patiënt in de palliatieve fase voor alle patiënten** **hetzelfde is.**

R: Je bedoelt de wijze waarop zij willen participeren in die besluitvorming?

**I: Ja.**

R: Nee, dat is heel verschillend. Sommige mensen willen heel actief meedenken en willen wel op basis van de informatie die ze krijgen zelf die keuze maken. Er zijn ook mensen die zoiets hebben van: nou, dokter of zuster, zegt u het maar, help me daarbij. En er zijn mensen die samen met de dokter in gesprek willen gaan over wat ik net aangaf, van: wat hebt u in de aanbieding en wat gaat me dat kosten aan bijwerkingen en belasting? Hoe kijk ik daar dan tegenaan? Dan is die context belangrijk, om extra te vragen naar: welke doelen hebt u nog? Als iemand bijvoorbeeld piano speelt, moet je misschien geen chemotherapie gaan voorstellen waarvan je weet dat dat zenuwschade gaat opleveren, bijvoorbeeld. Als iemand bijvoorbeeld zijn werk zo lang mogelijk wil doen… Je moet dan heel goed kijken ook naar andere ziekten die mensen hebben. Als iemand cardiaal belast is of diabetes heeft of een longziekte heeft, zeg het allemaal maar, die algehele conditie; die weging probeer je dan te maken. Ik zie wel dat dat bij sommige dokters echt wel die kant op gaat en dat het bij andere misschien minder aan bod komt. Ook bij onszelf is dat soms nog een zoekproces. Het gebeurt ook weleens dat iemand een behandelvoorstel heeft gekregen van de dokter en dat hij vervolgens bij ons komt, dat toch nog een nuance wordt aangebracht, omdat hier dan ineens toch andere dingen naar voren komen in het gesprek. Het kan zijn dat het toch een aangepaste dosering wordt, dat het toch nog net even een andere chemotherapie wordt die net even lichter is. Nou ja, dat je toch net even een andere keuze maakt.

**21.07**

**I: Oké. Zou je een voorbeeld kunnen geven van een situatie waarin de gezamenlijke besluitvorming heel duidelijk was of een voorbeeld waarbij dat juist niet het geval is?**

R: Nou ja, onlangs nog was er een meneer die longkanker heeft, daarvan ook niet meer beter zal worden, palliatieve behandeling, die één palliatieve behandellijn heeft gehad, die vervolgens tijdens die behandeling twee keer een infarct krijgt, niet gerelateerd aan de chemotherapie, maar parallel daaraan is dat wel zo. Hij gaat dan naar het … (naam universitair ziekenhuis), er wordt een behandeling ingezet en dan blijkt eigenlijk dat ze die behandeling niet helemaal optimaal hebben kunnen doen. Nu is er sprake van progressie. Hij staat voor de keuze opnieuw die chemotherapie weer te moeten krijgen. Hij heeft het gevoel dat dat met grote haast moet en komt dan hier in het gesprek, bij mijn collega in dit geval, die aan hem uitlegt: die haast is er niet, dat kan best nog twee weken wachten, bij wijze van spreken. Dus we kunnen rustig kijken: wat zijn hiervan de voor en de tegens en wat zijn de angsten? Nou, de angst is natuurlijk: kan ik dat qua hartconditie wel hebben? Dus hebben ze besloten om eerst, voorafgaand aan de start van de kuur, toch nog een consult bij de cardioloog te hebben om die vragen goed beantwoord te krijgen, waarbij uiteindelijk de conclusie van de cardioloog was: ik beoordeel u nu opnieuw, zoals het er nu voorstaat – het was enige tijd geleden, er zat een half jaar tussen – denk ik dat het veilig kan. Dat heeft die meneer voldoende gerustgesteld om er goed in te gaan, waarbij hij ook duidelijk aangaf wat voor hem van belang was nog in het leven. Dat was dat hij graag elke zaterdag met zijn kleinzoon naar het voetbalveld gaat. Dat was voor hem cruciaal. Als dat niet meer zou kunnen, dus als zijn conditie dat niet meer zou toelaten voor langere tijd, dan zou hij willen stoppen. Dat is dan mooi, als je dat boven tafel krijgt. Dan heb je toch gezamenlijk iets van: dat gaan we dan wel doen. Dan moet je heel goed monitoren hoe dat gaat.

**I: Ja. En een voorbeeld van waar dat juist niet gebeurde? Dat is denk ik wel een lastige hoor.**

R: Ja, dat is best heel lastig. We komen weleens tegen dat, omdat er haast is bij bepaalde oncologische aandoeningen… Dat is meestal zo bij het kleincellige longcarcinoom, dat zijn vaak zieke mensen, waarbij je gisteren eigenlijk al had moeten beginnen met die chemo, als die diagnose eenmaal is gesteld. Als je een week wacht, kan het zo vergevorderd zijn dat mensen erg ziek zijn en het niet goed meer lukt. Mensen knappen daarvan vaak ook heel erg op of niet, want ze kunnen ook onderweg heel veel bijwerkingen krijgen. Dan is het een beetje erop of eronder. Dan is het niet altijd in alle gevallen gelukt, denk ik, om dat heel goed over te brengen. Mensen hebben weleens het idee, als ze aan zo’n behandeling beginnen, van: ik heb geen keus. Een keus is er altijd, maar dat is een hele andere mindset natuurlijk. Als je niet meer actief behandelt, betekent het dat je zult gaan overlijden binnen afzienbare tijd. Sommige mensen zijn heel erg op de behandeling gefocust. Dan is het heel lastig om ineens die andere kant te laten zien, want soms zijn mensen daar nog niet aan toe. Dus je moet ook heel goed kijken: waar zit de patiënt in dat proces en hoe kan ik daar wel of niet aan komen?

**25.02**

**I: Ja. En wat zou helpen om dat voor patiënten duidelijker te maken?**

R: Duidelijkheid en eerlijkheid, denk ik, al vanaf het allereerste begin. En voor de hulpverlener denk ik dat je jezelf, als dat kan, rust geeft om die patiënt in zijn geheel goed te zien. Eigenlijk een soort time-out, wat in Nederland nu natuurlijk ook veel gebeurt in allerlei behandeltrajecten, dat als je een patiënt voorlichting hebt gegeven, je een soort time-out en bedenktijd inlast, die van nut kan zijn, zodat de patiënt vragen kan bedenken voor zichzelf, samen met de mensen die om hem heen staan, waardoor je in een tweede gesprek pas een besluit neemt. Dat je zoiets structureel inbouwt, wat je eigenlijk ook altijd doet bij trials. Dat zijn de officiële onderzoeken waaraan mensen meedoen. Dan is dat verplicht. Je mag niet vandaag een trial aanbieden en vanmiddag al starten met de behandeling, daar hoort bedenktijd bij. Eigenlijk zou je dat, denk ik, bij dit soort zaken altijd moeten doen, heel bewust. Dat is ook iets wat je natuurlijk met elkaar multidisciplinair zou moeten afspreken, dat je dat als dat kan, uitzonderingen daar gelaten, als je echt gisteren had moeten beginnen, moet doen.

**I: Oké. Ik ga even een stapje terug. Ik ging zojuist even een stapje verder, want ik vond het interessant om op de gezamenlijke besluitvorming in te haken. Ik wil even heel kort terug naar die aspecten, dat fysieke, psychische, sociale en spirituele welzijn. In navolging daarvan: werken jullie ook met een individueel zorgplan, waar dat soort dingen in worden vastgelegd?**

R: Ja, als wij de patiënt hier voor het eerst zien voor zo’n behandelplan, dan maken we een brief. Die is herkenbaar in de computer, omdat die dan onder Oncologie wordt weggezet. Als het om een long-oncologische patiënt gaat, staan al onze andere brieven onder Longen, zodat je het vervolg kan zien, maar de basis is altijd een Oncologie-intake. Daarin wordt dat allemaal beschreven. Ook naarmate de gesprekken vorderen, in een traject bij de gewone controles; als zoiets dan naar voren komt of als ze dat actief bespreken, wordt dat er ook ingezet en kun je dat terugvinden.

**I: Oké. Dus eigenlijk worden alle afspraken rond die aspecten vastgelegd.**

R: Ja. Ja.

**I: Oké. Ja, goede communicatie is natuurlijk de basis van goede zorg, zodat de patiënten goed geïnformeerd zijn en ook gezamenlijke besluitvorming kan plaatsvinden, waar we het net ook al over hadden. Zijn er patiënten bij wie je de communicatie moeilijker vindt?**

R: Ja, dat zijn de patiënten bij wie je het gevoel hebt dat je geen ingang hebt.

**I: Oké. Ja, dat zei je daarstraks ook al. Ja.**

R: Ja. Of *nog* geen ingang hebt. Dat kan natuurlijk komen door de persoonlijke klik die er misschien nog niet is. Dat kan. Of doordat je echt even eerst moet zoeken naar: waar is die patiënt en wat is de voordeur, wat is de achterdeur, hoe kom ik erin? Dat heb je niet altijd in één keer voor elkaar. Dat is ook wel timing soms, opbouw in het proces.

**I: Oké, dus dat is eigenlijk puur op basis van het gevoel eigenlijk.**

R: Ja en ook wel expliciteren, dat je het vraagt aan mensen. Soms voel je dat je er echt niks mee mag doen. Dan probeer je het de volgende keer. Of via een collega, dat je het doorspeelt, dat die de volgende keer de patiënt ziet. Dat is het meest lastig. Dat is niet altijd eenduidig. Bij sommige mensen lukt het vrij snel, ben je vrij snel tot de kern gekomen, en bij anderen duurt dat langer.

**29.12**

**I: Oké. Zou jij misschien een voorbeeld kunnen geven van momenten waarop dat niet zo goed gaat?**

R: Nou, als je merkt dat een patiënt bijvoorbeeld heel erg in de behandelstand staat, zoals wij dat dan uitdrukken, en wij daar af en toe wel bedenkingen bij hebben, zo van, goh, is dat wel wijs, maar de patiënt heel erg op dat spoor zit, want zo komt hij hier al binnen, dan probeer je dat te nuanceren. Je wilt ook niet alle hoop uit handen geven, want iemand bedenkt dit ook ergens voor en als het niet ergens over zou gaan, had hij nooit dat behandelvoorstel gekregen, dus het zal ook wel iets opleveren, maar het heeft ook zijn prijs. Dat moet je iemand ook wel goed duidelijk maken. Nou ja, dat probeer je dan wel te doen, maar soms is het dan lastig om die optie van helemaal niets doen, althans, geen actieve behandeling geven, lastig. Dan zeggen mensen soms wel: ja, maar dat is echt geen optie, ik ga ervoor, ik ga door. Soms willen mensen heel lang doorgaan om allerlei redenen. We hebben nog niet zolang geleden een mevrouw gehad, zeventig plus, die een inwonende dochter had en een inwonende kleindochter, die voor die dochter en die kleindochter eigenlijk altijd heeft gezorgd. Eigenlijk was die dochter niet geheel zelfstandig. Zo’n systeem was dat geworden. Die heeft heel lang gewoon gedacht: ik kan niet zomaar weg, ik heb geen tijd om dood te gaan, ik moet hier blijven voor mijn dochter en mijn kleindochter. Die lapte zichzelf altijd op als je haar hier zag. Daar kwam je niet zomaar binnen. Pas toen het echt slecht ging is dat gelukt. Soms is dat echt zo. Tenminste, dat ervaren wij dan allemaal zo. Dan is de observatie niet op één collega gebaseerd, maar op meerdere, en als je dan opeens een ingang hebt; toen was er ineens een moment dat we wel het maatschappelijk werk erbij konden halen, we ervoor konden zorgen dat de dingen werden geregeld en dat zij ook wel rust kreeg om dat laatste stuk te begaan. En een andere mevrouw die goed was opgeleid en die heel helder had wat haar te wachten stond, gaf ook altijd aan: ik weet heel goed hoe het in elkaar steekt, maar ik wil nu nog niet stoppen. Ik wil nog echt doorgaan tot ik het gevoel heb dat ik niet meer kan en dan moeten maatregelen worden genomen. Nou, dat bleek ook, maar dan moet je ineens daar heel snel op inspelen en ook snel handelen, want het mocht eerder niet. En bij andere mensen kun je dat heel goed parallel aan elkaar doen. Die beginnen met een palliatieve behandeling, maar die kunnen ook heel goed parallel daaraan nadenken hoe ze dat laatste stuk willen doen en dat vastleggen, in een la leggen en die la kan open als het zo ver is.

**I: Ja, precies. Je hebt het een paar keer gehad over binnenkomen en je gebruikt nu de metafoor “de la open”, maar hoe herken je dat moment? Is dat een gevoelskwestie of niet?**

R: Nou, bijvoorbeeld, die ene mevrouw die ik net benoemde, die belde mij en die zei: het gaat nu echt niet goed, mijn buik is weer dik – die mevrouw had heel veel vocht in de buik – ik denk dat er weer iets aan moet gebeuren. Nou, dan luister je goed en dan zeg je: nou, dat is heel naar en vervelend. Ik denk dat het heel goed is dat we nu een gesprek hebben op hele korte termijn om te kijken of dit nu nog, op dit moment, realistisch is en hoe het er nu echt voorstaat. Ze zei: ja, dat denk ik ook, want ik denk dat het echt niet goed gaat.

**I: Oké en dat is dan het moment…**

R: Dat is dan het moment. Dan wordt het bevestigd. Je probeert het vaak even af te tasten. Dat had ik vaak gedaan bij haar. Nu zei ze: ja, dit is het moment. De volgende dag is ze geweest bij een collega en die heeft dat goed opgepakt. Ja. Ja. Dus het is wel zo dat we het vaak noemen en dat als het moment er dan is, dat het dan ineens mag en dan kun je doorpakken. Ja. Maar bij anderen loopt het heel mooi al vanaf het begin parallel hoor. Dat heb je ook wel met grote regelmaat. Ja.

**33.32**

**I: Ja. Oké. En is dit anders bij mensen die bijvoorbeeld laagopgeleid zijn of die een migratieachtergrond hebben bijvoorbeeld, die communicatie?**

R: Die hebben we eigenlijk niet zo heel veel hè, mensen met een migratieachtergrond.

**I: Ja, maar ook mensen die bijvoorbeeld laaggeletterd zijn of die moeilijk dingen begrijpen, is daar de communicatie anders?**

R: Dan moet je echt op een ander niveau met hen spreken, een andere taal.

**I: Dat gaf je al aan, ja.**

R: Soms chronisch, om het zo maar te zeggen, als dat kan. Als ze maar gewoon goed begrijpen voor welke diagnose ze hier zitten en dat als de ene behandeling niet meer werkt de volgende eventueel beschikbaar is, maar dat dat elke keer een keuze is. Dat kun je dan op allerlei verschillende niveaus aan mensen duidelijk maken. Maar soms, wat ik eerder ook al zei, is het zo dat mensen die laaggeletterd zijn in dit soort zaken heel rigoureus zijn en heel duidelijk weten wat ze willen.

**I: Oké, dus eigenlijk juist…**

R: Dat valt me weleens op in deze regio. Ja. Sommigen hebben zoiets van: doe maar gewoon, daar heb ik al lang over nagedacht. Dan staat mijn dochter voor me klaar en de buurvrouw.

**I: Ja, ja. Wat je ook al zei, dat het vanzelfsprekend is dat iedereen…**

R: Ja. Ja. Ja. Maar je moet dat wel aftasten. Je moet dat natuurlijk wel expliciteren.

**I: Ja. Oké. Voor mijn volgende vraag heb ik een aantal kaartjes. Daarop staat wilsonbekwaamheid en levenseindebeslissingen. Levensbeschouwing en culturele achtergrond. Even kijken, dit is een lange: wettelijke vertegenwoordiging in een situatie van acute verslechtering. Het past er nét op. Niet-behandelafspraken. Crisissituaties. Even kijken hoor. Ziekenhuisopnames. Plaats van zorg en sterven. Zou je met behulp van een rangorde in de kaartjes kunnen aangeven in hoeverre deze onderwerpen aan bod komen in een consultgesprek?**

R: Dan wil je graag de rangorde van wat altijd wordt besproken en wat minder vaak wordt besproken.

**I: Ja.**

R: Ja. Dit wordt vaak besproken.

**I: Niet-behandel… Ik zeg het even hardop hoor. Je ziet het niet op de audio.**

R: Ja. Ik weet niet nog niet precies wat ik… Dit wordt door de arts vaak besproken, de niet-behandelafspraken. De plaats van sterven proberen wij eigenlijk altijd wel met de patiënt te bespreken als ze een palliatieve indicatie hebben, ergens in het traject. Dat geldt ook op een gegeven moment voor ziekenhuisopnames: wil je dat nog wel? Crisissituaties, dat bespreken we eigenlijk ook altijd, ook wel in een vroeg stadium. Crisissituaties worden eigenlijk altijd benoemd in de zin van: wie moet je bellen als er een crisis is, hoe zijn we dan bereikbaar en hoe kun je dan daarover met ons communiceren? Levensbeschouwing en culturele achtergrond. Ik denk wel dat het veel met elkaar te maken heeft.

**I: Je bedoelt dit thema en de overige thema’s…**

R: Nee, deze twee. Culturele achtergrond in relatie tot levensbeschouwing. Ja, je probeert er eigenlijk wel achter te komen hoe mensen in het leven staan. En als ze bijvoorbeeld geloven, op welke manier geloof dan voor hen belangrijk is. En als ze niet geloven welke andere levensovertuiging… Hoe staat iemand in het leven? Het is wel iets wat we toch met grote regelmaat wel bespreken in onze consulten. Dat moet hierbij, denk ik. Wettelijke vertegenwoordiging in situaties van acute verslechtering. Als er situaties zijn waarbij mensen niet zichzelf kunnen vertegenwoordiger wordt het zeker... Wilsonbekwaamheid en levenseindebeslissingen. Ja, ook in situaties waarin ze dat niet zelf kunnen zal dat wel benoemd worden en is het duidelijk wie dat dan voor mensen beslist.

**37.58**

**I: Oké. Dus eigenlijk komen alle thema’s aan bod.**

R: Ja, volgens mij wel.

**I: En er zijn er een aantal die wat meer…**

R: Nou, dit komt natuurlijk niet altijd voor hè? Het is maar een kleine groep waar dat van toepassing is.

**I: Afhankelijk van de situatie.**

R: Ja. Ja. En dit komt aan bod als je met elkaar het idee hebt, samen met de patiënt, dat een actieve behandeling van onze kant niet meer op zijn plaats is. Maar anders is een ziekenhuisopname wel op zijn plaats, als je nog actief aan het behandelen bent, want de complicaties die wij dan veroorzaakt hebben, moet je ook weer oplossen. En als de patiënt naar de huisarts gaat, dan wordt vastgelegd of iemand nog ziekenhuisopnames wil. Als dat nog wel zo is, bijvoorbeeld voor een bloedtransfusie, ik noem maar iets, maar dan weer direct naar huis, dat wordt dan vastgelegd.

**I: Oké. Nou ja, duidelijk. Ik had nog één thema waar ik even snel doorheen ga. We zitten alweer bijna op drie kwartier. Dat gaat eigenlijk over beperkte gezondheidsvaardigheden. Heb je weleens gehoord van die term, gezondheidsvaardigheden?**

R: Nu in het kader van het onderzoek wel en anders had ik het misschien zelf net even anders genoemd.

**I: Ja, oké. Want wat betekent het volgens jou?**

R: Het betekent volgens mij dat het mensen zijn die niet altijd alles goed begrijpen, daardoor niet altijd goed weten hoe ze er eigenlijk voor staan qua gezondheidssituatie, daardoor niet altijd de juiste verwachtingen hebben van wat we nog allemaal wel en niet voor hen zouden kunnen doen en daardoor niet altijd de therapie op de juiste wijze kunnen uitvoeren, omdat ze het niet goed hebben begrepen.

**I: Oké. Dat past heel goed bij onze term, denk ik.**

R: Ja. En soms misschien ook wel mensen die er niet goed mee overweg kunnen, die er niet mee om kunnen gaan, die in de ontkennende fase zitten. Dat zou je ook een beperkte gezondheidsvaardigheid kunnen noemen. Die niet realistisch naar hun situatie kunnen kijken, ook al hebben ze het intelligentieniveau wel.

**I: Ja, oké. En hoe vaak heb je te maken met patiënten met dit soort beperkte vaardigheden?**

R: Toch wel met enige regelmaat. Dat we ook wel aanvullende interventies doen, bijvoorbeeld met de thuiszorg, om medicatie uit te laten zetten door de thuiszorg, omdat we denken dat het helemaal niet goed gaat komen. Dat je daarbij natuurlijk ogen kan hebben thuis, extra, die meekijken. En soms met formulieren, behandelschema’s extra goed op papier zetten met afstreeplijstjes, zodat mensen kruisjes kunnen zetten dat ze het ook gedaan hebben. Er is natuurlijk ook wel veel patiënten informatie die daarin wel voorziet. Of een mantelzorger die extra ingezet wordt. En soms zijn mensen ook overbelast, ook de mantelzorgers, dus dan probeer je op tijd te signaleren dat er misschien toch extra thuiszorg bij moet.

**41.18**

**I: Ja. En hoe herken je patiënten met dat soort vaardigheden?**

R: Doordat mensen vaker dezelfde vraag stellen. Dan weet je daardoor dat het niet begrepen is. Soms ook, als ze voor de tweede keer komen, dat je ziet dat ze niet goed hebben nageleefd wat je had uitgelegd. Dan denk je: oh, dat is dus niet goed gegaan. Soms voel je het aan alles, direct. Ja, eigenlijk aan hoe mensen reageren, hoe ze kijken, hoe ze dingen kunnen teruggeven. Je probeert ook te checken van: heeft hij het nou goed begrepen? Kunt u het in uw eigen woorden nog een keer vertellen? Als je daaraan ziet, het lukt niet… Of als je bijvoorbeeld merkt dat mensen toch wel overbelast zijn of dat de mantelzorger overbelast is, dan kan dat ook een reden zijn om de thuiszorg te vragen te helpen.

**I: Oké. Want behalve zo’n teach-back, dat je zegt van, goh, probeer eens in uw eigen woorden uit te leggen… Zijn er nog andere manieren waarop je rekening probeert te houden met communiceren?**

R: Je probeert eigenlijk altijd aan te sluiten bij de taal van die patiënt, voor zover het lukt, daar snel een beeld van probeert te krijgen. Ook wel door externe hulp te opperen. Als mensen dan zeggen, ja, heel graag, dan weet je ook dat je goed zit.

**I: Ja, precies. Dan is het duidelijk, ja.**

R: Ja. Ja. Dat ze zich daarin gesteund voelen. Of dat ze zeggen: ik snap er helemaal niks van, ik zie door de bomen het bos niet meer. Het is ook altijd zo dat na onze uitleg de collega’s van de chemokamer die de infusen toedienen ook nog extra uitleg geven, dus daar komt ook wel een signaal vandaan, van: ze hebben het toch niet helemaal goed begrepen. Dus als je mensen verdwaasd naar die papieren ziet kijken die je hebt gegeven, dan zie je wel van: dit komt zo niet goed.

**I: Ja. Ja, precies, dan heb je al een idee van…**

R: Ja. Of door wat ze zeggen. De hele non-verbale uitstraling. Dus je ziet het eigenlijk als het duidelijk is heel goed, maar de mensen die heel goed zijn in het verbloemen daarvan en die net doen alsof ze het allemaal wel begrijpen, die schieten er wel door, die kunnen erdoor schieten. Ja.

**I: Oké. En behalve dat je op een gegeven moment ziet, dat is niet goed gegaan met medicatie of zo, zijn er nog andere barrières in de zorg aan patiënten met dat soort vaardigheden?**

R: Jawel. Dat ze bijvoorbeeld niet bellen als ze dat wel hadden moeten doen, in een crisissituatie bijvoorbeeld, terwijl we hen daar wel goed over hadden geïnstrueerd. Dan zeggen ze hier in … (streek in Nederland), dat stond op het briefje, ik kon koorts krijgen, dus nou, dat moet ik dan accepteren. We zijn bikkels, we gaan gewoon door. In zo’n situatie moet je juist bellen. Dus als je ziet dat iemand doodziek via de huisarts binnenkomt, terwijl we hen anders hadden geïnstrueerd, dan denk je: hé, dit is niet goed. Of als je ziet dat mensen slecht voor zichzelf zorgen. Dat zijn allerlei dingen waaruit je het soms kunt afleiden.

**44.41**

**I: Oké. En wat zou er bijvoorbeeld nodig zijn of kunnen zijn om dat te kunnen verbeteren, de zorg aan patiënten met dat soort vaardigheden?**

R: Nou, op het moment dat je dat merkt direct extra hulp inzetten, heel laagdrempelig en vroegtijdig. We bellen eigenlijk ook altijd de mensen, sowieso. Als het iedere keer wel goed gaat en je merkt ook aan de feedback van de patiënt dat hij het goed kan handelen, dan kun je dat weer wat afbouwen. Maar als je denkt van, hé, ik merk aan alles dat deze patiënt het niet goed heeft begrepen, dat hij veel zieker is, dat je het niet vertrouwt, dan laat je iemand komen en dan heb je toch vrij snel in de gaten wie een zorgenkind is, zoals wij dat dan noemen, en wie niet. De zorgenkinderen bel je vaak. Zo proberen we dat over het algemeen te achterhalen. En soms moet je zorgen dat er in de thuissituatie versterking komt door thuiszorg in te zetten bijvoorbeeld. Ja. Zodat je daar extra ogen hebt. Of via de praktijkverpleegkundige van de huisarts of via de huisarts. Dus dat je op die manier een samenwerkingsverband zoekt. Laatst nog, bij jou (wijst naar collega in de kamer), een psychiatrische patiënt die in die zin kwetsbaar is dat er dan contact gezocht wordt met de psychiatrische verpleegkundige die hem op dat punt begeleidt, want zo iemand kan eventueel ook weer problemen krijgen door de medicijnen die wij verstrekken of door de druk van zo’n behandeling die wij geven. Dan ligt meteen al de basis bij de intake, zodat ze daar ook weten: dit gaat bij jullie patiënt gebeuren. Kijk van jullie kant mee, wij kijken mee. We zoeken elkaar op als het nodig is. Ja. Ja, dus zo proberen we het over het algemeen te doen.

**I: Oké. Nou, duidelijk. Ik wilde nog op één ding inhaken. In ongeveer het begin van ons gesprek gaf je aan dat de communicatie soms goed ging, maar dat het ook soms niet goed ging. Het ging in eerste instantie over jouw rol in de zorg voor patiënten in de palliatieve fase. Toen ging het op een gegeven moment over de aanwezigheid van één centrale zorgverlener, dat die er dus is, en dat met meerdere zorgverleners in het team wordt samengewerkt, dat ook samen wordt gewerkt met de huisarts, met maatschappelijk werk. Volgens mij had het te maken met die samenwerking met die partijen, waarbij dus de patiënt centraal staat, dat dat eigenlijk bijna altijd goed gaat, maar dat het soms ook niet goed gaat. Volgens mij was dat de context.**

R: Je kunt weleens iets missen, signalen missen. Als mensen niet duidelijk zijn over dat er toch thuiszorg moet zijn, dat het niet goed lukt, als mensen dat maskeren en we hebben het zelf op dat moment niet in de gaten, dan kan zoiets erdoor glippen, dat je dat niet signaleert, waardoor je het ook niet oppakt. Dan denk je dat je duidelijk bent geweest in je uitleg en ook in alles wat je hebt besproken, maar dan blijkt dat toch niet altijd zo te zijn. Dat kan weleens gebeuren. Ja.

**I: Ja. Ja, op die manier. Heb jij nog aanvullingen op het interview, opmerkingen, toevoegingen?**

R: Nee, ik denk dat wij hier goed op weg zijn. Ik denk dat we er nog niet zijn, dat er ook nog wel wat dingen te verbeteren vallen. Vooral als je het hebt over die hele groep van verpleegkundigen die dit werk doen, om de neuzen daarin dezelfde kant op te krijgen, maar ook nog richting het multidisciplinaire team, dat we een mooi gestroomlijnd geheel krijgen. Daar zijn we mee aan het werk, samen ook met het palliatieve adviesteam, de collega’s daar. Dat is wel een mooie uitdaging. Ja.

**49.14**

**I: Ja. Oké. Oké. Nou, hartstikke duidelijk.**

**[50.46 – einde opname]**

Z3a04

I: interviewer

R: respondent

**I: Zijn er tot zover vragen?**

R: Nee hoor. Ik zou zeggen, ga je gang.

**00.30**

**I: Oké. Allereerst wilde ik het graag hebben over uw rol als zorgverlener in de palliatieve zorg aan patiënten. Wat is uw rol?**

R: Dat is een hele goede vraag.

**I: Ja. Laten we breed beginnen.**

R: Ja. Mijn basisrol is dat ik longarts ben, al achttien, negentien jaar in dit ziekenhuis. In je werk als longarts kom je natuurlijk steeds meer mensen tegen die in het eindstadium van hun leven zitten en langzamerhand palliatief worden. Dat is een stukje zorg waar we eigenlijk heel weinig aandacht aan schenken. Dat is heel belangrijk, omdat we daar uiteindelijk allemaal mee te maken krijgen, of je nou wel of niet gezond bent. Wat je dan palliatief noemt, is dat mensen langzamerhand een eindpunt van hun leven in zicht zien en dat het toch belangrijk is dat dat stukje leven wat je nog te leven hebt in goede kwaliteit gaat gebeuren, zo goed mogelijk. Dat kan je eigenlijk alleen maar doen door echt daar bewust mee bezig te zijn, met dat palliatieve fase. Dat is ook de reden waarom ik vorig jaar of twee jaar geleden een opleiding heb gevolgd, apart daarvoor. Dan ben je kaderarts Palliatieve Zorg. Er zijn steeds meer mensen in Nederland die die opleiding volgen, huisartsen, maar ook langzamerhand steeds meer medisch specialisten. Om in ieder geval tools en handvatten te krijgen om op verschillende onderdelen van die palliatieve zorg in te gaan. Nou, daar haal je voldoening uit. Voor de patiënt is het natuurlijk het belangrijkste. Dat merk je. Ik heb vanmiddag ook twee patiënten gezien. Daar besteed je dan een uur aan. Dan praat je over allerlei dingen. Het feit dat mensen op een andere manier hun verhaal kwijt kunnen, met meer rust en tijd, is voor hen al heel erg belangrijk. Dat geeft gewoon een gevoel van: ik voel me daardoor al wel beter, ik kan mijn ei kwijt. Nou ja, dat is een beetje de rol. Hoe kun je die palliatieve zorg verbeteren, uitbreiden? Door te gaan verspreiden in het ziekenhuis. Dat is ook de reden waarom we nu sinds gisteren een vakgroep – misschien heeft J. dat ook wel verteld – Palliatieve Zorg in het leven hebben geroepen. Daarvoor hebben natuurlijk een aantal voorbereidingen plaatsgevonden. Dat gaan we nu ook uitdragen in het ziekenhuis, zodat niet alleen wij de palliatieve fase, de markeringsfase kennen voor patiënten, maar ook andere dokters, dat je niet eindeloos doorgaat. Dus het doel is eigenlijk met zo min mogelijk middelen mensen toch een goede kwaliteit van leven kunnen geven, dat je niet eindeloos doorgaat met behandelingen. Dat zijn allemaal aspecten waar je op moet letten. Het is vaak veel belangrijker om iets niet te doen dan wel te doen in de zorg. Als je hier als patiënt komt, hebben dokters altijd het gevoel iets te moeten doen. Dat is juist heel goed, om bewust voor jezelf daarmee bezig te zijn, van: kan ik ook niet over andere dingen praten, bijvoorbeeld over het behandelbeleid? Wat kun je nou doen als je omvalt? Dat soort dingen meer. Dat kun je niet zomaar met mensen gaan bespreken, maar wel als je daar tijd voor neemt. Dat is wel belangrijk. Dus dat heeft allemaal met dat palliatieve werk te maken. Zo zie ik het dan. Dat doe je natuurlijk al een hele tijd, maar naarmate je zelf ook ouder wordt… Dat ga je misschien ook wel merken: je wordt natuurlijk ouder en op een gegeven moment ga je toch over dingen anders denken, zeker als je in de gezondheidszorg zit. Zo gaat dat.

**04.06**

**I: Ja. Oké. Ik hoorde al wat over tijd, over meer tijd. U gaf ook aan: tools en handvatten. Kunt u wat voorbeelden noemen?**

R: Die je kunt gebruiken om… Nou ja, elke ziekte, elke chronische ziekte heeft zijn eigen tools. Nou ben ik natuurlijk longarts, dus vanuit de longziekten weet je dat ook wat beter. Kijk, het is heel duidelijk, als mensen kanker hebben, dan is het makkelijker om dat te herkennen. Daar gaat het ook om, tools om te herkennen dat iemand palliatief wordt. Dat is belangrijk. Nou ja, we zien ook heel veel mensen met COPD in het eindstadium. Nou ja, je hebt een vraag over tools hè? Bedoel je nou tools voor het herkennen van een palliatieve fase bij mensen, van: hé, dit kun je gebruiken om erachter te komen dat iemand eigenlijk palliatief is?

**I: Nou, ik denk dat ik hem breder bedoelde. U gaf aan dat die palliatieve fase eigenlijk steeds meer aandacht krijgt en dat er steeds meer tools en handvatten komen om daarmee om te gaan in de praktijk.**

R: Ja.

**I: Toen dacht ik, dan ben ik wel nieuwsgierig naar deze tools, maar bijvoorbeeld ook in de herkenning, wanneer iemand palliatief wordt, dat zou ook kunnen hoor.**

R: Ja, precies. Tools kun je gebruiken voor de herkenning. Dat hangt van het ziektebeeld af inderdaad. Maar tools om daar energie in te stoppen; dat is gewoon door een polikliniek daarvan te maken, dus door een aparte polikliniek te creëren waar je dit soort mensen kan zien, met rust. Een andere tool/manier is om samenwerking op te zoeken met huisartsen of andere zorgverleners. Huisartsen, dat is natuurlijk een hele belangrijke groep. Die zijn een aanzienlijke tijd van hun werk ook bezig met palliatieve zorg, soms zonder dat ze het weten. Het niet weten en het dus ook niet kunnen markeren. Daar moet je ook de samenwerking in opzoeken. Een huisarts kun je ook helpen met het herkennen van bepaalde dingen. Huisartsen hebben het allemaal niet gemakkelijk. Dat zijn mensen die met weinig tools/middelen toch een bepaalde zorg moeten gaan leveren. Dat is voor ons natuurlijk vaak in het ziekenhuis wat makkelijker. Nou ja, tools, samenwerking, verpleeghuizen, andere ziekenhuizen, onderwijs – vooral heel belangrijk – geven aan arts-assistenten, coassistenten, verpleegkundigen. J. en W., de verpleegkundig consulenten doen dat ook heel veel. Ik doe dat iets minder, maar de bedoeling is ook om dat meer te gaan doen. Dus je moet het gewoon bekend maken. Als je nou een vakgroep wordt; dat is wat anders dan een commissie. We zijn hiervoor altijd een commissie geweest, maar als je een vakgroep wordt, dat zegt het woord al; je kent natuurlijk de vakgroep Cardiologie, Longziekten, Interne Geneeskunde. Die bestaan tientallen jaren. De vakgroep Palliatieve Ondersteunende Zorg bestaat nog niet. En nu gaat dat heel langzaam in ziekenhuizen ontstaan. Ik geloof dat in Nederland twee of drie ziekenhuizen dit ook al doen. Wij zijn de vierde die dat doen. Dat is meer om het body te geven, herkenbaarheid te geven, zodat een chirurg die altijd aan het snijden is, denkt van: hé, palliatief, oh, een vakgroep, wat is dat? Snap je? Dus om hen ook het idee te geven van: goh, waar ben ik nou mee bezig, kunnen dingen ook anders? Dus je probeert je echt constant te laten zien. Dat moet je natuurlijk ook wel netjes doen, want dokters zijn allemaal gericht op herstel van dingen, niet om ervoor te zorgen dat je bij iets wat niet meer hersteld kan worden toch een stukje kwaliteit en zinvolheid… Daar gaat het ook om. Kijk, jij bent natuurlijk jong, je bent bezig met onderzoek, dat is jouw zingeving. Je zinvolheid is om hiermee bezig te zijn. Iedereen is in zijn leven bezig met iets wat hem in leven houdt. Maar als je chronisch ziek bent en het einde van je leven kan naderen, dan wordt dat allemaal heel anders. Hoe kun je er nou voor zorgen dat die mensen toch een stukje levensgevoel kunnen krijgen van: hé, nou, daar doe ik het nog voor. Dat is best lastig. Daar zijn J. en W. bijvoorbeeld, verpleegkundigen, best wel sterk in. Die houden zich ook veel bezig met zingevingsvragen bijvoorbeeld. Dat is best lastig, om daarover te praten. Daar kan ik ook nog wat van leren. Ook al ben ik palliatief bezig, ik ben toch ook steeds geneigd om hulpmiddelen te zoeken om die aan patiënten te geven en te kijken of ze zich daar beter door voelen. Dat moet ook wel, daar ontkom je niet aan, maar je moet het ook in zijn geheel bekijken, gewoon aandacht schenken, wat je wel meent, dat je laagdrempelig bereikbaar bent als dingen nodig zijn. Bereikbaarheid. Dus we zijn bijvoorbeeld ook bezig met eHealth. Dat is ook een manier om zorg te geven op momenten buiten de polikliniek om, op gezette tijdstippen. Dat zijn allemaal gezette tijdstippen, maar je moet het daartussen ook kunnen leveren. Allemaal nieuwe dingen. Dat moeten we ook allemaal gaan bekijken. Ze staan allemaal op het lijstje. We hebben een document waar al die dingen in staan. Dat is wel leuk. Of “leuk”, het is uitdagend.

**09.14**

**I: Ja. Uitdagend, interessant. Om even terug te komen op uw rol als zorgverlener in die palliatieve zorg, is er altijd een centrale zorgverlener aanwezig voor een patiënt?**

R: Je bedoelt op palliatief gebied?

**I: Ja, bent u dat of is dat bijvoorbeeld M.?**

R: Ja, weet je? Dat heeft te maken met hoe het team is opgebouwd. Dan heb ik het even over overdag, van maandag tot en met vrijdag. We hebben een palliatief team, een palliatief adviesteam. Daar zitten gewoon vier dokters in. Daar zitten twee internisten in, daar zit een intensivist in en een longarts, dat ben ik dan. Wij zijn dan de vier dokters die betrokken worden bij palliatieve zaken waar W. en J., consulenten, palliatieve verpleegkundige consulenten, als eerste naartoe gaan. Die sonderen het probleem. Die worden ook in consult gevraagd. Zoals het nu gaat, in het ziekenhuis, worden zij in consult gevraagd. Zij bespreken dan de casus met ons. Wij hebben het van tevoren natuurlijk ook in de computer allemaal bekeken, foto’s ook en al die dingen meer. Dan worden we op de hoogte gebracht, op allerlei domeinen van de palliatieve zorg. Dan gaan wij ook zelf naar die patiënt toe daarna. Dat is wel heel apart. De patiënt ken je niet altijd, heel vaak niet. Dat vergt nog best wel tact, bescheidenheid om je toch langzamerhand… Mensen moeten niet meteen denken: oh, daar komt hij aan. Snap je? Dat is heel grappig. Ik moet zeggen, de meeste mensen vinden het best wel fijn dat iemand op een stoel bij hen kan zitten en over allerlei dingen praat, wat natuurlijk ook wel met hun ziekte te maken heeft, met alles wat eromheen draait. Het aparte van palliatief bezig zijn, is dat ik me in dit geval niet alleen met de Longziekten bezighoud, maar met het hele gebied hè, van de Gynaecologie tot de Interne. Dat betekent eigenlijk ook dat je daar eigenlijk ook wat meer van moet gaan weten. Dat betekent eigenlijk ook dat Palliatieve Geneeskunde, zo kun je het ook noemen, eigenlijk een vakgebied moet gaan worden in zijn algemeenheid. Dus je kunt internist worden of cardioloog of longarts, dat is een opleiding van zes jaar, maar eigenlijk moet Palliatieve Geneeskunde ook zoiets gaan worden, een opleiding van vier tot zes jaar, ik noem maar even iets. In de toekomst zou dat ook… Nou ja, aan de andere kant zijn er ook mensen – zo is het ook hoor – die zeggen: het is generalistische zorg, elke dokter moet dat kunnen, tot een bepaalde hoogte. Maar niet alle palliatieve problemen zijn goed op te lossen en daarom is het helemaal niet gek dat je daar ook dokters voor hebt of gespecialiseerde verpleegkundigen, die daar ook heel veel mee kunnen. M. die je net hebt gesproken is oncologieverpleegkundige, maar zij vindt op haar gebied ook heel veel van dit soort raakvlakken. Dus die mensen zijn ook belangrijk. Uiteindelijk doe je het ook met een team hè, met zijn allen. Het is best een grote vakgroep. Er zijn iets van tien, vijftien mensen die erbij betrokken zijn. Als er nou een internistisch probleem is, dan ga je natuurlijk ook meer een internist erbij vragen voor adviezen. Ik bedoel, ik weet ook niet alles van leukemie af, ik noem maar even iets, vooral in het eindstadium daarvan, wat je allemaal nog kan doen om mensen een goede kwaliteit van leven te geven, dus je doet het met zijn allen uiteindelijk.

**12.31**

**I: Ja. Oké. En in het kader van “met zijn allen”; behalve internisten, u noemde de huisarts al… Zijn er ook nog andere partijen die erbij betrokken worden, in de zorg voor zo’n patiënt?**

R: Ja, weet je? Wat heel belangrijk is, is natuurlijk de patiënt en zijn directe kring eromheen. De mantelzorger is ook ontzettend belangrijk. Dat kan een familielid zijn of vrienden. Die komen hier ook op onze polikliniek. Dan zie je ook dat die ook een behoorlijke tik van de molen krijgen. Die moeten zorgen, die maken zich ook zorgen, dus die horen ook aandacht te krijgen en steun te krijgen. Je moet vanuit de kern gaan. De patiënt is er een, de mantelzorgers daaromheen. Daaromheen heb je natuurlijk gelijk de huisarts die erbij betrokken is. Daaromheen is dat de thuiszorg. Dat is de werkelijkheid. De thuiszorg heeft heel veel te maken met palliatieve zorg. Die ziet die mensen thuis. Wij zien hen natuurlijk op het moment dat ze in het ziekenhuis zijn of op de poli komen. Vooral de thuiszorg; je hebt heel veel thuiszorgorganisaties. Elke thuiszorgorganisatie heeft zijn palliatieve knowhow. Iedereen doet het wel op een vergelijkbare manier, maar de ene partij doet dat weer anders of intensiever of professioneler dan de ander. Dus wat zou mooi zijn? Dat je al die partijtjes die je hebt – Buurtzorg, TSN en noem maar op – uiteindelijk allemaal onder één noemer zet, dat je eigenlijk één soort organisatie hebt van mensen die even goede zorg op dezelfde manier met dezelfde professionaliteit en kennis en kunde kunnen geven. Dat is eigenlijk de palliatieve zorg, met de huisarts als roerganger. Zo gebeurt het in Nederland. In Duitsland bijvoorbeeld gaat het weer anders. Daar heb je weer supergespecialiseerde verpleegkundigen die dat doen. Twee van de intensivisten van ons ziekenhuis werken ook een beetje in Duitsland. Daar hoor je dat die palliatieve zorg wat minder bij de huisarts ligt. Die laat dat meer over aan de gespecialiseerde verpleegkundigen, die zijn meer leading. Dat is anders daar. Dat heeft heel veel voordelen ook. Huisartsen hebben het ook zo druk met heel veel dingen. Die vliegen van hot naar her. Dus wat dat betreft zou het ook mooi zijn als je zoiets vergelijkbaars in Nederland zou kunnen krijgen. Maar goed, al die discussies zijn nu ook gaande, hoe je die zorg moet gaan leveren. Een belangrijke zorg, ik noem maar even iets, is de ambulance. Mensen bellen heel vaak 112 op als het thuis mis gaat, ook bij palliatieve patiënten, van wie ze weten, jongens, die wordt niet meer beter, die gaat dood; die bellen tien dagen voordat iemand waarschijnlijk zal overlijden toch nog de ambulance op en die halen zo iemand dan op. Dan wordt die weer naar het ziekenhuis gebracht en dat is niet goed, zoals dat gaat. Dus de hele communicatie daaromheen, daar zijn we ook heel druk mee bezig, om dat goed te krijgen. Dus ook ambulancemensen moeten ook betrokken zijn bij de zorg van een patiënt. Die moeten precies weten, als ze komen of een melding krijgen, van: hé, die patiënt is het, zo moeten we het aanpakken. De huisarts; jij hebt een huisarts, ik heb een huisarts. Die heeft natuurlijk niet altijd dienst. Er zijn ook andere huisartsen die dat overnemen en die moeten dat ook weten. Dat gaat ook niet altijd goed. Die weten soms ook dingen niet. Ik zou het niet weten. Die bellen ook 112 op, van: haal maar op. Dus heel die zorg, dat is natuurlijk heel breed, die moet in elkaar samenvallen. Je moet van elkaar weten wat je doet. Ja, dus de informatie ligt natuurlijk ook vooral bij de patiënt en die moet inzichtelijk zijn voor iedereen, maar ook voor de patiënt zelf en ook voor de familie. Alles, de brieven die we hebben, dit alles, moet allemaal inzichtelijk zijn. Dan weet je gewoon wat er met je is. Dat moet je natuurlijk op een hele goede manier uitleggen, dat mensen niet in paniek raken. Nou ja, zo moet je dat doen. Ja.

**I: Oké. Oké. En werken jullie ook met een zorgplan voor een patiënt?**

R: Ja, daar zal J. ook wel iets over gezegd hebben misschien. We werken in het ziekenhuis hier met een zogenaamd proactief zorgplan. Dat is ook bedacht door het UMCG. Ik vind dat ook een heel sterk plan. Kijk, het eenvoudigste plan is eigenlijk onze brief hè, om het zo te zeggen. Daar begint het vaak mee. Als dit het startpunt is; we maken een uitgebreide brief. Daar staan allemaal punten in waar je op moet letten: overweeg dit, overweeg dat, levensverwachting, wel of geen reanimatie. Als mensen van hier naar huis gaan… Niet voor iedereen, maar eigenlijk zou het voor iedereen moeten gebeuren die palliatief is: een zorgplan maken waar alle items van zorg worden benoemd, euthanasiewensen, noem maar op, van alles. Dat plan gaat mee met de patiënt. Die heeft dat bij zich. Dan is het idee dat andere zorgverleners die bij die patiënt komen ook in één oogopslag kunnen zien wat het probleem is, wat er aan de hand is. Alleen het plan is papier en er zijn heel veel papieren bij de patiënt. Er zijn heel veel andere plannen ook. Dan moet je ook weten dat dat in de praktijk niet altijd goed loopt. Daarom is het ook heel belangrijk om van de huisartsen te horen van: jongens, hoe gaat het met die plannen, wat vinden jullie ervan? Ik weet ook dat er zeker wel mensen zijn die dat prima vinden, dat dat er is, maar patiënten verstoppen plannen ook, dat soort dingen meer. Maar het is wel mooi dat je een startplan hebt en maakt. Het is van eminent belang, zo’n plan, dat dat verder uitgewerkt wordt voor de lange termijn, door artsen, door andere zorgverleners, thuiszorg of wat dan ook. Puur in interactie met wat de patiënt wil. Dat is constant belangrijk hè? Ik heb dat ook, ik vergeet dat soms ook. Je moet heel goed weten: wat wil een patiënt, wat weet hij, wat wil hij weten, wat wil hij? Dat is niet altijd makkelijk hoor, voor een patiënt, om dat te beantwoorden. Maar elke patiënt heeft het gevoel wat hij graag zou willen. Daar moet je heel goed naar luisteren eigenlijk. Dat is niet makkelijk altijd.

**18.37**

**I: Nee, want dat is dan eigenlijk een soort van gezamenlijke besluitvorming, waar je dan op komt.**

R: Ja. Precies.

**I: Even kijken hoor, dan ga ik even een stap verder. Dan is mijn volgende vraag in hoeverre er sprake is van gezamenlijke besluitvorming, met patiënten in de palliatieve fase?**

R: Ja, weet je, dat doen we wel. Kijk, een voorbeeld even van een longkankerpatiënt. Ik heb er gisteren ook eentje gehad, een vrij jonge vrouw met allemaal huidmetastasen. Ja, die is palliatief. Die wordt niet meer beter en die krijgt palliatieve behandeling met chemotherapie. Dus wat heel belangrijk is in die shared decision making is dat je de patiënt en de partner, of de familie die erbij is, volledig op de hoogte brengt er ook tijd voor neemt, over hoe de situatie is, wat de behandelingsmogelijkheden zijn en dat het ook belangrijk is eigenlijk, ook zodat mensen horen… Die vallen om van de informatie, dus je moet daar absoluut ruimte voor geven door hen nog een keer te laten komen, door hen te laten praten. Nou, je hebt net met M. gesproken. Zij is oncologieverpleegkundige. Zij zijn heel belangrijk in onze organisatie. Die geven ook adviezen daarin. Maar goed, uiteindelijk geef je de mogelijkheden van wat je kan doen: chemotherapie wel of niet gaan geven? Nou ja, ik hoor reacties waarbij er ook mensen zijn die zeggen: joh, dat hoeft voor mij niet. Heel veel mensen zoeken zelf dingen op, terwijl je zou zeggen van: goh, dat zou je eigenlijk best wel kunnen doen, als ik dat zo inschat. Maar sommige mensen hebben daar geen behoefte aan. Nou, een voorbeeld, dat hebben wij niet meegemaakt, dat heeft in het Medisch Contact gestaan: een dokter die slokdarmkanker kreeg en die behandeld is met chemo-/radiotherapie. De laatste stap was de slokdarm eruit halen. Nou, dat wou hij niet. Daar heeft hij heel veel over nagedacht, al die dingen meer. Prognostisch is het beter om dat te doen, dan leef je langer, maar de kwaliteit van leven kan een gigantische klap krijgen als je hem kwijtraakt en dat wilde hij niet. Nou, hij leeft nog steeds. Maar dat zijn keuzes. Ik zeg dat al jaren hoor, van: jongens, uitleggen wat er aan de hand is, wat zijn de mogelijkheden, wat kun je wel en niet doen? Gewoon de nadelen en voordelen benoemen, maar het ook niet zo serveren dat je het helemaal aan patiënten overlaat. Dat moet je ook weer niet doen. Ik probeer daar wel zelf ook een gevoelswaarde in te geven. Bij sommige patiënten zeg ik ook weleens: nou ja, als u mijn vader zou zijn, dan zou ik dat en dat gevoel hebben. Maar weet je? Het is een interactie. Nou ja, dan kan zo’n spreekuur best wel uitlopen, maar het is wel belangrijk dat het besluit goed genomen wordt. Er worden overigens ook onderzoeken gedaan – ik heb er zelf eigenlijk nooit patiënten naartoe gestuurd – met huisartsen. Dat heeft een naam. Het UMCG doet daaraan mee. Dat is namelijk als we zo’n slechtnieuwsgesprek hebben dat de patiënten daarmee naar de huisarts gaan en dat ze met de huisarts dan bespreken van: wat gaan we doen? Dan horen we van de patiënt en de huisarts… De huisarts speelt ook een hele belangrijke rol in: wat ga je doen? Dus de huisarts bellen we ook meteen op. Als ik tegen jou wat moest zeggen, bel ik gelijk de huisarts op. En brieven; ik maak brieven zelf, ik kan vrij snel typen, dus die brief gaat ook gelijk… De huisarts heeft hem gelijk al. Een druk op de knop en hij heeft hem. Dan bel je hem op en dan leg je dat uit. Dat is heel belangrijk. Mensen gaan…

**I: Die komen dan weer bij de huisarts en dan…**

R: Precies. Die moet dat weten. Die moet dat echt weten. Veel dokters doen dat ook hoor, op die manier. Niet allemaal, maar het is van eminent belang. Er zijn ook mensen, die kunnen vaak niet geloven wat er aan de hand is. Aan de andere kant hoor je ook vaak mensen zeggen: ik wist het wel. Mensen denken er toch over na. Mensen komen hier in deze streek niet zomaar, die komen niet zo gauw. Ze hebben vaak al lange tijd klachten. Mensen zijn hier best wel nuchter hoor, maar heel reëel.

**I: Ja, die heb ik vandaag ook al een paar keer gehoord. De nuchtere (streek in Nederland) ...**

R: Ja, precies. Dat zijn ze. Ja. Soms moet je het wel uit ze trekken hoor. Net ook weer, een COPD’er, eindstadium. J. heeft ermee gesproken, uitgebreid. Dan komt er niet zoveel uit. Die man sluit zich af. Dat is heel belangrijke informatie die ik van hem krijg.

**I: Je hoort van J. van: ik heb het echt geprobeerd, maar…**

R: Ja. “Die is afgesloten.” Ik zie die man. Ik heb hem een half jaar geleden ook gezien. Het gaat niet goed met die man. Hij is kortademig, ziet er anders uit. Hij rijdt nog auto, dat kan hij nog wel, maar dat gaat met moeite. Het is gelukt om de inhalatiemedicatie te veranderen. Fysiotherapie wilde hij eerst niet, wil hij nou wel. Ik heb een uur lang met hem zitten praten, met de familie erbij, een dochter, schoonzoon. Zijn been, zijn vaten zitten dicht, de bloedvaten van zijn benen. De chirurg zegt: dat gaan we niet opereren. Maar die man lijdt daaronder, die heeft er zoveel last van. “We gaan niet opereren omdat hij qua longen slecht is. Dat is een risico, zo’n operatie met zulke slechte longen. Maar ik zeg: als je dat nou heel goed met elkaar bespreekt, longarts, patiënt, chirurg, dan kunnen we een plan maken om te kijken of je toch behandeld kan worden. Zo moet je dat gewoon doen. Dan gaan die mensen toch anders denken en praten. Het is belangrijk om dat goed te verwoorden in de brief, ook naar de huisarts toe. Nou ja, een brief voor het ziekenhuis. De chirurg kan dat ook lezen, dat ik dat openlaat. Ik bedoel, er zijn genoeg mensen met ernstige COPD die iets aan hun blindedarm/darmen krijgen, ja, die moeten wel onder het mes. Heel velen overleven dat gewoon goed qua longen. Snap je? Dus niet alles hoeft meteen…

**I: Dat lijkt me ook een hele moeilijke inschatting natuurlijk.**

R: Ja, je moet dat inschatten op een bepaalde manier. Inderdaad, soms lukt het niet, maar bij een aantal weer wel. Maar daar moet je weer tijd voor hebben. Dat hebben we hier op de polikliniek, dat creëren we dan en dan zeg je: oké, hebben we toch wel iets kunnen bereiken. Dat lukt niet bij iedereen hoor, maar bij zo’n man die helemaal gesloten is lukt het dan toch om wat voor elkaar te krijgen. De familie er ook bij is belangrijk, een dochter die elke dag bij hem komt, die hem helpt. Nou ja, dat is een stukje shared decision making wat je doet.

**I: Ja, precies. Als ik voor mijzelf al vast een soort tussenconclusie zou mogen trekken is het dat tijd en aandacht in zo’n consult, niet alleen van u, maar ook van J., (verpleegkundige) en de communicatie tussen jullie allemaal eigenlijk maakt dat je echt concrete shared decision making kan voeren, als ik het goed begrijp.**

R: Klopt. Ja. Nee, daar heb je gelijk in.

**I: Oké. En is dat bij iedere patiënt hetzelfde?**

R: Nee, dat is per mens verschillend. Er zijn ook mensen bij wie je dat niet kan doen.

**25.31**

**I: Oké, wat voor mensen…**

R: Ja, weet je? Het is de minderheid hoor, maar er zijn een aantal mensen… Dat heeft ook weer met IQ te maken, maar daar gaat dit ook over. Hoe krijg je mensen… Sommige mensen kunnen ook zelfs een normaal IQ hebben, die willen daar gewoon niet over praten. Die geven dat ook aan. Die blokken dat af. Dat is best lastig.

**I: Dat kan ik me voorstellen.**

R: Een ander gesprek op een ander moment moet je dan voeren. Soms heb ik dat al gelijk in de gaten, dat ik dat nu niet moet bespreken. Dat weet ik al van tevoren, want dat wil niet.

**I: Oké. Is dat dan een gevoelskwestie?**

R: Dat heeft met intuïtie te maken. Of met “intuïtie”… In mijn presentatie heb ik al verschillende mensen laten horen; de kunst van het laten. Dat is een plaatje van Albert Einstein. Die zegt dat. We zijn allemaal bezig met rationeel denken. Alles is op basis van feiten, gegevens, formules. We leven in een datatijdperk, dat wordt steeds erger. Maar er is iets wat onderschat wordt, dat is de menselijke intuïtie, creativiteit. Dat. Dat heeft iedereen bij zich. Niet om mezelf of onszelf in de schijnwerpers te zetten, maar het is wel iets – dat heeft ook met ervaring te maken – wat je op een gegeven moment voelt en merkt. Je kunt er soms ook best wel een keer een misslag mee maken hoor, maar naarmate je vaker een bepaalde situatie hebt gezien denk je: goh, dat moet ik niet doen. Dat moet je op een andere manier doen op een ander moment, maar wel zodanig dat ik toch wel het gevoel heb van, ik heb toch wel redelijk wat bereikt. Bijvoorbeeld een behandelbeperking bespreken, niet reanimeren, dat lukt niet bij iedereen, om dat aan het begin te bespreken. Moet je je voorstellen dat je hoort dat je ongeneeslijk ziek bent en dat er gelijk verteld wordt: als je omvalt, laten we je mooi liggen. Snap je? Daar komt het op neer. Zo vertel je het niet, maar… Dan lukt het niet altijd om dat allemaal in één keer aan iemand te vertellen. Dan moet dat op een andere manier gebeuren. Dat is dan mooi, dan kun je ook de verpleegkundig specialist daar… Die zijn daar ook heel belangrijk voor. Die zijn aanvullend.

**I: Ja. Ja, dat had ik inderdaad begrepen.**

R: Ook al hebben we vaak de witte jas niet aan, we zijn toch dokters. Dat weet de patiënt ook. Al komt het alleen al door de naam. Nou, verpleegkundigen zijn heel belangrijk daarin, als steun.

**27.56**

**I: Ja. Kunt u een voorbeeld geven van een situatie waarin shared decision making niet goed loopt? Is dat dan wat u net zelf aangaf zo’n moment dat zo’n patiënt er gewoon echt niet over wil praten. Is dat zo’n moment? Of wat zou dan zo’n moment zijn dat dat lastig is, shared decision making?**

R: Ja, weet je, laatst heeft iets me toch wel moeite gekocht. Een dame van 82 jaar, compleet dissimuleren. Lachend binnenkomen, terwijl ze een grote longtumor heeft, terwijl haar hart het niet doet, ze COPD heeft. Die komt dan met een dochter en die had ook nog haar dochter bij zich. Die dochter; ik was er al voor gewaarschuwd, dat die alles voor haar moeder wil. Alles moet gebeuren bij moeder. Dat is lastig, want je probeert dingen in de realiteit uit te leggen, wat het betekent. Dat ging ook over wel of niet gaan beademen in acute situaties. Ja, dan heb je gewoon drie mensen voor je. Jij zit hier alleen. Dan moet je het niet zo gaan doorduwen, van: jongens, we gaan niet reanimeren. Dat wil dan niet. Dan maak je een deal. We hebben er ook van gemaakt: oké, zoals het nu is wordt het gewoon een reanimatieprocedure voor een korte periode, dat doen we. Ik denk dat als ik die mevrouw alleen had gehad, dat het anders was geweest. Die dochter had er een heel… Die keek constant met enorm grote ogen om zich heen, die was heel bang, ook bang om haar moeder te verliezen. Maar goed, die mevrouw is naar de cardioloog gegaan. Die was gewoon bezig met een sigaretje roken thuis, was gewoon langzaam dood aan het gaan. Die klaagde niet, maar die had een hele slechte hartfunctie, ernstig COPD, longkanker. Nou, die diagnose hier kwam er gewoon uit. Dat moet je hun dan vertellen. Dat kostte hen heel veel moeite om te begrijpen. Nou, uiteindelijk, om een lang verhaal kort te maken, ze is naar de cardioloog gegaan. Medicamenteus is het hart toch weer in een betere pompstand gezet. Zij bleek gelukkig niet gemetastaseerd te zijn, kon ook niet geopereerd worden en ze was ook te zwak om een diagnose te stellen. Dus puur op een CT… Als je dit ziet, zie je hier zo’n grote knol zitten. Ze krijgen ook een PET-scan in Groningen, dan kijk je of er uitzaaiingen zijn. Die zijn er niet. We gaan dat lokaal bestralen. Uiteindelijk is dat radicaal bestraald. Dat doe je om toch in opzet een stukje genezingskans te krijgen, terwijl die kans klein is hoor, bij dit soort ziektes ook. Tien procent kans. Die vrouw heb ik afgelopen maandag, eergisteren, gezien en die ziet er veel beter uit. Ze begon weer over het reanimatiebeleid te praten, weer, want daar hebben we heel veel over gesproken. Ik zei: nou, de situatie is veranderd. Ze is in een betere conditie, ze doet weer dingen. Het reanimatiebeleid staat gewoon nu op maximaal. Maar als het toch misgaat… Als het gebeurt in het ziekenhuis, intensivisten krijgen daarmee te maken. Als zij het verhaal lezen; de pompfunctie blijft natuurlijk heel matig, ze heeft een ernstig COPD, ze heeft kanker. Als zij iets acuuts krijgt, dan is de kans groot dat je op basis van medische gronden gewoon niet gaat intuberen, dat je dat niet gaat doen, dat je geen reanimatiepogingen gaat doen. Het ligt eraan hoe de situatie is, maar dat dan de intensivisten zeggen: jongens, dat heeft geen zin. Dan doe je dat op medische gronden. Dan kan de familie hoog of laag springen als ze willen, dat kan dan niet meer. Dan heb je daar je reden voor. Juridisch kun je dat doen, maar dan moet je natuurlijk wel heel goed de familie, hoe moeilijk het ook is voor hen, dat gaan uitleggen, hoe de situatie is. Dus je moet er niet ingaan om een soort strijd te winnen. Je moet er geen strijd van maken. Maar het is puur zoeken en kijken. Je bent constant mensen aan het scannen, voor jezelf, alsmaar aan het scannen. Hoe kijken ze? Wat vinden ze? Hoe reageren ze? Snappen ze het wel of snappen ze het niet? En je kunt je er ook een buil aan vallen. Ik heb ook een keer een paar jaar geleden een ouder echtpaar gehad. Nou, die snapten het allemaal, de behandelbeperking. Die gingen het daarna met hun dochter bespreken. Die waren in paniek. Dus daarna met dochter weer, weer uitgebreid met de dochter gesproken en dat is allemaal goed gekomen. Zo kan het ook hè? Alles wat je ziet hoeft niet echt zo te zijn. Daar kun je je ook in vergissen. Of “vergissen”, het was geen vergissing, maar die mensen gingen toch thuis nadenken. Die hadden het ook niet opgenomen op een bandje. Oudere mensen doen dat niet zo gauw. Maar dat was misschien wel handig geweest, om dat dan weer te horen. Maar die dochter, die toevallig in het ziekenhuis werkte… Maar dat is goed gegaan. Gewoon uitleggen en dat was prima. En dan leg je het nog een keer uit. Nou ja, elke patiënt is weer anders. Dat is ook zo. Dat blijkt ook wel uit de gesprekken die je dan voert met hen. Ja. Ja.

**33.18**

**I: En denkt u dat het beter zou zijn als er meer gezamenlijke besluitvorming zou plaatsvinden met de patiënt?**

R: Ja, ik denk dat het geleidelijk aan wel meer gebeurt. Ik kan natuurlijk niet in de keukens van andere dokters kijken, maar het kan meer gebeuren. Ik heb soms… Wij zien gewoon toch best wel vaak bij de internisten en ook bij mijn eigen collega’s; we zijn met zes longartsen. Alle zes doen we het op dezelfde manier. We zijn allemaal verschillend. Maar ik vind soms weleens dat een aantal dingen ver gaat. Dan kun je ook de vraag stellen… Dat vind ik niet altijd makkelijk, dat je ook naar je collega’s zegt van: moet je dat wel doen joh? Snap je? Dat is lastig. Dat vind ik soms lastiger dan dat aan een patiënt proberen uit te leggen. Bij bepaalde collega’s kun je het wel doen, maar niet bij allemaal. Een voorbeeld, dat had niks met kanker te maken. Een oude baas van drieënnegentig jaar, tweeënnegentig jaar, al zou hij negenentachtig zijn, die had een slaapapnoesyndroom. Een slaapapnoesyndroom, negenentachtig jaar. Die houdt zijn adem ’s nachts iets te veel in. Ja, die moest dan behandeld worden door een grote kap op zijn gezicht ’s nachts. Nou, toen moest ik dat… Ik moest er iets mee doen. Ik las dat. Nou, toen heb ik gewoon toch daar iets over gezegd tegen mijn collega. Nee, die man werd opgenomen voor een registratie nog een keer voor zijn slaapapnoe in (plaats ziekenhuis), maar die had klachten en daar waren wat dingen mee. Toen kreeg ik te horen wat er allemaal met die man gebeurt. Bijna 90 jaar, slaapapnoeregistratie, kijken of hij daarvoor ingesteld kan worden, voor de behandeling. Dan ben je zo oud, richting het eind van je leven. Die man was niet lekker en heel toevallig bleek hij op dat moment een hartinfarct te hebben. Dat was puur toeval. Maar ik heb uiteindelijk wel tegen mijn collega gezegd van: goh… Ik heb het via de mail gedaan, want ik zie hem niet altijd en dat is ook weer niet handig, via mails. Dat is niet goed.

**I: Dan mis je toch die…**

R: Precies. Heel voorzichtig, heel voorzichtig heb ik het uitgelegd. Nou, jongens, ik kreeg toch de shit over me heen.

**I: Is dat zo moeilijk tussen artsen?**

R: Ja. Ja, het ligt soms gevoelig. Ja. Het ligt soms gevoelig. En dat kan een belemmering zijn voor de palliatieve zorg. Dat is ook een uitdaging.

**I: Oké, dat zou mijn volgende vraag zijn inderdaad. Hoe zit dat dan in die…**

R: Dat kan een belemmering zijn.

**I: Aangezien u ook aangaf dat dat een nieuw iets is, dat dat misschien meer body moet krijgen in de ziekenhuisorganisatie.**

R: Ja. Dus je probeert door een vakgroep te maken dat dokters daar ook meer over kunnen nadenken, van: jongens, dat moeten we eigenlijk niet doen. Ik heb het al twee keer meegemaakt tijdens een weekenddienst hier, dat ik iemand heel erg benauwd op de interne afdeling zag liggen, gewoon zo benauwd. Daar had ik niks mee te maken. Toen heb ik wel de betrokken internist gebeld, van: goh, wat gebeurt daar, die man gaat niet goed, heel benauwd. Ja, die lag er voor een probleem met zijn darmen, had ook COPD erbij, emfyseem. Die betrokken internist vond dat ook, maar die deed daar eigenlijk zo min mogelijk aan, omdat het niet zijn patiënt was. Hij had wel dienst, hij moest er natuurlijk wel voor waken, maar de oorspronkelijke hoofdbehandelaar was een andere dokter en die is gewoon meer zo van: zo wil ik het en zo wil ik het niet. Die zag daar een enorme drempel in. Nou, dat heb ik met hem besproken. Ik zeg van: nou praat je met mij. Wij vinden dat dit zo niet kan. We hebben dingen allemaal beschreven, genoteerd. Hij was zo blij, want hij zat ermee in zijn dienst. Uiteindelijk hebben we die meneer morfine gegeven, zodat die benauwdheid wat verminderde. Hij is een paar dagen later overleden hoor, dat was ook helemaal logisch, maar zo zie je dat ook tussen internisten… Bij toeval kom je er dan achter dat daar een drempel is. Dat gebeurt in de praktijk… Dat gebeurt niet zo vaak, maar het gebeurt wel. Snap je? Het is heel belangrijk om te proberen de palliatieve zorg, de grenzen van handelen, gewoon toch bij iedereen … Nou ja, dat ze eruit zichzelf aan gaan denken. Dat is het doel. Dus wij zijn eigenlijk adviserend daarin. Ondersteunend, adviserend. Dus niet beschouwend. We doen zelf zo min mogelijk. Maar meer: aan deze dingen moet je denken, hier kan je aan denken, al moet je gewoon niks meer doen. Zo moet je dat doen. Of een verpleegkundige komt naar ons toe en zegt: B., die patiënt lijdt, die is van die en die dokter, dat kan niet. Dat gebeurt ook hè. Dat is een verpleegkundige die dat vraagt. Dat gebeurt niet zo vaak, maar ze mogen het doen. Dat is ook een beetje de bron. Een tijd geleden is hier een patiënt niet gereanimeerd en doodgegaan. Die verpleegkundige is ontslagen uit het ziekenhuis. Dat heeft zelfs in het Medisch Contact gestaan, landelijk. Een verpleegkundige weigerde een reanimatie, bij iemand die wel gereanimeerd moest worden. Die verpleegkundige had misschien wel gelijk. Snap je? Maar een verpleegkundige mag nooit zomaar een reanimatie niet doen terwijl het wel moet. Maar de verpleegkundige had wel gelijk. Wat eigenlijk verpleegkundigen ook kunnen doen is van tevoren – daar zijn wij ook voor – een discussie… Je kunt best ons erbij halen. Dan kunnen wij het gesprek met de verpleegkundige aangaan of met de betrokkene dokter. De verpleegkundige kan best wel verkeerd denken. Dat kan ook, dat die te weinig argumenten heeft voor zichzelf om zoiets te kunnen vinden. Zo’n verpleegkundige moet je dat ook uitleggen. Maar het kan ook zijn dat de dokter eigenlijk te ver gaat. Dus het kan soms heel erg gevoelig liggen. In dit geval was het een escalatie, wat zelfs de landelijke bladen heeft bereikt. Dat gebeurt. Maar dat heeft een grondslag gewoon van: hoe ver ga je in je behandeling? Iedereen heeft ermee te maken, ook verpleegkundigen. Die zien heel veel, veel meer dan wij eigenlijk. Dus die zijn heel belangrijk voor ons team gewoon, met elkaar.

**39.16**

**I: Ja, precies. Oké. Even tussendoor, in het kader van de tijd, wij hebben tot kwart over geloof ik hè?**

R: Ja, zo globaal.

**I: Ja, oké. Dan ga ik eventjes…**

R: Ik kan ook wat korter en krachtiger beantwoorden.

**I: Nou, nee, dat bedoel ik niet persé hoor. Dan ga ik denk ik eventjes over op de beperkte gezondheidsvaardigheden. Is dat een term waar u weleens van gehoord hebt?**

R: Ja, dat dat bij patiënten kan bestaan. Ja.

**I: Ja. En wat betekent dat volgens u?**

R: Volgens mij betekent dat dat mensen een verminderd innerlijk vermogen hebben om hun eigen gezondheid… Wat het betekent voor henzelf en voor hun omgeving, om dat goed in te kunnen schatten, de ernst van wat je kunt verwachten, voor nu en de toekomst. Dat ze doordat ze dat niet begrijpen geweldig in de problemen kunnen raken. Nou ja, dat ze slechter kunnen worden hadden ze kunnen bedenken, maar dat hebben ze niet begrepen. Op die manier kunnen ze in de problemen raken. Dus het is van belang dat je dat herkent, dat die mensen zo zijn, dat je dat op een naïevere manier en met tijd moet gaan uitleggen. En met modellen. Zoals je kunt zien, we hebben hier allerlei modellen. Ik ben een van de weinige dokters die met heel veel kleurtjes werkt, modellen van de long werkt, met olifanten werkt. Hier om in 3D uit te leggen hoe CT’s zitten. Dat blijkt ook, als je dingen uitlegt met voorbeelden, tekeningen, schetsen; wat tastbaar is onthouden ze beter. Voor mijn gevoel werkt dat ook beter, even los daarvan. Dat blijkt ook uit de praktijk, dat dat beter is. Ik heb een scheurkalender, geneeskundige scheurkalender en daar stond dat in. Onderwijs, dingen uitleggen. Tien procent onthoud je. Vijftien procent. En dan, op het laatst, gaat het langs je heen. Dus dat is wel belangrijk. Ja.

**41.10**

**I: Oké. En hoe herkent u patiënten met beperkte gezondheidsvaardigheden?**

R: Ja, dat is een goede vraag. Dat is een manier van praten, op welke manier ze de vragen beantwoorden. Ja, vaak ook hun onderlinge communicatie. Het vinden van de juiste woorden. De manier waarop ze gekleed zijn. De manier waarop ze verzorgd zijn. Dat komt op hetzelfde neer. Je weet het natuurlijk ook wel uit voorgaande medische informatie. De brief van de huisarts is van belang. Mensen roken intenser. Lifestyle, laat ik het zo zeggen. Jij en ik kunnen ook roken, maar we kunnen verder alles goed begrijpen, maar lifestyleproblemen zie je. Ja, zo kun je misschien wel meer dingen benoemen. Je zou Tokkies kunnen noemen, maar Tokkies kunnen best wel heel slim zijn. Daar zitten slimme bij. Dat zegt niet altijd alles. Als je op een gegeven moment vraagt van, goh, begrijpt u het, kunt u het nog eens uitleggen, dan… Dat soort dingen. Dat doe ik niet zo vaak hoor, want dat vind ik vaak een soort domheidstestvraag, maar goed. Zo haal je het eruit. Er zijn best wel wat mensen die daaronder vallen. Ja. En sommige mensen zijn analfabeet. Die kunnen dat heel goed bedekken.

**I: Die kunnen dat heel goed maskeren.**

R: Maskeren. Ja, precies.

**I: Ja, precies. En wat zou er volgens u nodig zijn of nodig kunnen zijn om zorg beter aan te kunnen laten sluiten bij mensen met beperkte vaardigheden? Je hebt kleurtjes, modellen en dat soort dingen.**

R: Ja, dat soort dingen. Toch wel wat tijd, want mensen zijn vaak ook angstiger. Tijd. Nou ja, dit. Wat je allemaal kan doen om dat goed op te vangen bedoel je hè, was je vraag?

**I: Ja.**

R: Je goed voorbereiden. Heel belangrijk is je poli heel goed voorbereiden en de patiënt holistisch zien. Als zo’n patiënt komt op mijn poli, dan gaat het niet alleen om de vraag van de huisarts. Meestal zijn mensen hier al bekend. Wat het probleem is, elke dokter heeft zijn eigen medische voorgeschiedenis hè? Een dermatoloog heeft zijn voorgeschiedenis, een oogarts, cardioloog, neuroloog, longarts. Vaak is dat allemaal versplinterd. Wat ik allemaal doe, voordat je komt weet ik eigenlijk alles van je, wat op schrift is, wat de huisarts heeft gesteld. Dus voorbereiding is belangrijk, want dan kun je dingen al zien. Nou ja, zo globaal, misschien mis ik nog wat. Maar zo kun je dat doen, denk ik. Ja.

**43.59**

**I: Ja. Oké. Dan wil ik graag nog één vraag stellen, als dat kan.**

R: Ik zag de papiertjes al liggen. Daar ben ik nooit zo goed in, in een intelligentietest.

**I: Oh nee, het is zeker geen intelligentietest hoor ☺. Even kijken. Ik heb hier een aantal aspecten. Wilsonbekwaamheid en levenseindebeslissingen. Ziekenhuisopnames. Het zijn eigenlijk thema’s die in consulten ter sprake kunnen komen. Crisissituaties. Niet-behandelafspraken. Deze is lang: wettelijke vertegenwoordiging in de situatie van acute verslechtering. Plaats van zorg en sterven. Levensbeschouwing en culturele achtergrond. Mijn vraag is of u met behulp van een rangorde zou kunnen aangeven in hoeverre bepaalde thema’s ter sprake komen in consulten, in uw consulten.**

R: Even kijken hoor. Wat heel belangrijk is… Dat gaat over palliatieve patiënten dan?

**I: Ja.**

R: Heel belangrijk is dit, de behandelafspraak. Even kriskras hoor. Ja, de behandelafspraak. Wat ga je doen in acute situaties? Dat kunnen natuurlijk crisissituaties zijn, als mensen fysiek ineens helemaal onderuitgaan. Of wordt hier meer de geestelijke kant bedoeld? Dat weet ik niet. Maar goed, anders passen ze een beetje bij elkaar. Ja, wat heel belangrijk is, is dit. Ziekenhuisopnames. Dat is vooral voor klinische patiënten zo. De zinvolheid om opnieuw opgenomen te worden stel ik heel vaak – ik niet alleen, meerderen – ter discussie. Dit loopt vaak mis. Ik ben een van de weinige dokters die het al vijftien jaar oplost door onder andere de ambulancedienst op de hoogte te brengen van de situatie.

**I: Oh ja, wat u al aangaf.**

R: Dus de brieven krijgen ze ook, maar wel zodanig dat de patiënt dat ook weet en de familie dat ook weet en de huisarts dat ook weet. Dus dit wordt besproken. Ja, plaats van zorg en sterven is natuurlijk ook wel van belang. Ik praat even vrij associatief hoor. Dit is iets wat natuurlijk van belang is, maar dat geldt voor mensen die hier in het ziekenhuis zijn en dan weer weg gaan. Waar gaan ze dan naartoe? Blijven ze in het ziekenhuis of gaan ze thuis… Dat is iets wat we ook heel veel overlaten aan de verpleegkundig specialisten hè? Daar bemoei ik me iets minder mee. Mijn uitspraak is wel zo: de beste plek voor de meeste mensen is toch de eigen omgeving. Dat probeer je in het gesprek met de patiënt altijd wel te benoemen. Maar dat kan ook een hospice zijn. Ja, wilsonbekwaamheid en levenseindebeslissingen. Dat is een lastige. Ik kom dat nog niet zo heel vaak tegen. Dat vind ik een lastige hier. Ik ben dat niet zo heel vaak tegengekomen. Weet je? Een behandelbeleid en al die dingen meer; dat doe je niet alleen met de patiënt, maar ook met de familie erbij en alles. Ik bedoel, de familie moet dat ook begrijpen, vind ik. Als iemand nou wat minder wilsbekwaam is, dan vangt de familie dat wel weer op, omdat ze als groepje dat wel allemaal wat beter snappen. Nou ja, wettelijke vertegenwoordiging in de situatie van acute verslechtering. Ja, precies. Wat doen je dan in een situatie? Wettelijke vertegenwoordiging. Nou ja, inderdaad, dat je zegt, we gaan niet reanimeren, en niemand is er bijvoorbeeld om dat te kunnen zeggen of de patiënt is niet in staat om dat zelf te kunnen zeggen. Dat denk ik dan. Ja, ik moet het zeggen, niet jij hè? Ja, dan neem je besluiten, terwijl een wettelijke vertegenwoordiger er niet bij is bijvoorbeeld. Bijvoorbeeld iemand uit … (plaats in Nederland voor geestelijk gehandicapten), iemand die geestelijk gehandicapt is, die daar leeft, die dat allemaal niet kan zeggen. Dat is een lastige. Dat hangt af van wat er bekend is van de patiënten, vooral ook wat hun kwaliteit van leven is, wat je dan wel of niet gaat doen. Ja, dat vind ik een lastige, deze. Maar goed, oké. Levensbeschouwing en culturele achtergrond. Dat is op zich ook wel belangrijk. Ik denk dat deze fenomenen veel vaker voorkomen in gebieden waar allochtone mensen wonen. Dat is in deze streek minder het geval.

**I: Het is een redelijk homogene streek denk ik hè?**

R: Best wel. Dit is een hele lastige. Hoewel, levensbeschouwing; iedereen heeft zijn levensbeschouwing natuurlijk. Ja, hier zitten heus wel katholieke mensen of gereformeerden. Daar heb ik niet zoveel problemen mee. Die denken toch ook best wel reëel. Maar als je moslims tegenkomt, wanneer moslims pijn hebben, mag deze pijn niet met bewustzijns verminderende middelen behandeld worden. Zij moeten helder blijven tot aan het einde. Nou, bij dat soort mensen moet je ontzettend oppassen om morfine te geven. En als iemand doodgaat; dat moet je niet tegen de patiënt zelf zeggen. Als je dat doet, dat vindt de familie een ramp. Dat weet ik natuurlijk van anderen en van de opleiding. Als je dat namelijk tegen de patiënt zegt, gaat hij veel eerder dood en dat willen ze niet. Wij zijn in onze cultuur open. Wij zijn als Nederland op een bepaalde manier best wel ver vooruit bezig in vergelijking met andere landen, vooruitstrevend, levenseindeklinieken, noem maar op, de witte poedertjes die je kunt krijgen of niet. Maar je moet heel erg rekening houden met deze mensen. Dat is niet makkelijk, maar ik kom dat heel weinig tegen hier. Moet ik een volgorde aanbrengen? Nou, weet je, het is alles bij elkaar. Behandelafspraak, dat is wel belangrijk. Ja, zo een beetje.

**I: Oké. Ja, ik vond de toevoeging per aspect (kaartje) al voldoende eigenlijk. Ik wilde daar eigenlijk op doorvragen van, welke zorgverleners worden er dan voor gebruikt, maar dat heb ik allemaal eigenlijk al gehoord.**

R: Ja, dat is best een… Misschien kun je er nog meer over zeggen hoor, maar dat is gewoon wat me in eerste instantie te binnen schiet. Ja. Ja.

**50.37**

**I: Heeft u verder nog aanvullingen of toevoegingen aan dit interview?**

R: Nou, nee. Wij vinden het altijd leuk dat… Je merkt gewoon dat palliatieve zorg en alles wat ermee te maken heeft steeds meer aandacht krijgt. De volgende week of de week daarna heb ik twee andere interviews van onderzoekers. Jij bent ook een onderzoeker, maar vanuit het huisartseninstituut uit Leiden. Nee, je merkt gewoon dat het booming business is. Nou ja, een beetje een raar woord ervoor, maar dat het leuk is om… Nou ja, ongemerkt, dat wij er best wel wat ervaring in hebben en dat je dat best wel kwijtraakt, dat het een prettige manier is om het zo kwijt te raken. Nou ja, het is een heet onderwerp en we praten er veel over. Het is een andere manier om erover te praten. Je gaat er toch weer even wat meer over nadenken, over wat je doet als je dit soort vragen stelt. Dat vind ik wel aardig. Ja, dat vind ik goed, dat daar ook aandacht aan besteed wordt. Ja, dat is leuk. Ja. Dat is wat ik ervan vind. Ja. Nou ja, dat je er ook wat aan hebt natuurlijk.

**I: Ja, zeker.**

R: Je gaat er ook uiteindelijk een soort verslag van maken?

**I: Het wordt getranscribeerd, dit gesprek, dus het wordt uitgewerkt. Ja, dat wordt geanalyseerd en daar wordt heel nauwgezet naar gekeken. Daar gaan we uit halen wat erin zit. Dat is heel waardevol, zeker.**

R: Ja. En dan ga je dat nog verwerken in een stuk wat gepubliceerd wordt, een artikel of wat dan ook?

**I: Ja, zeker. Ja, wij doen het onderzoek. Pharos gaat daar een toolkit voor maken. Dat wordt op een gegeven moment dus, ik neem aan ook bij het … (naam ziekenhuis), geïmplementeerd en dan wordt er weer een meting gedaan. Dus in 2020 worden weer gesprekken gevoerd en wordt er weer gefilmd. Dan gaan we dus kijken wat de effectiviteit is van zo’n toolkit, wat die doet. Tussendoor wordt ook gerapporteerd en ook wetenschappelijk gepubliceerd, want daar is niet zo heel veel over. Hoe dat precies medisch zit weet ik natuurlijk niet, maar qua communicatiewetenschappen is het zeer beperkt, de palliatieve zorg. Dus dat. Er wordt zeer veel mee gedaan.**

**[54.32 – einde opname]**

Z4d01

I: interviewer

R: respondent

R: Vandaag is het 1 mei hè?

**I: Ja, 1 mei, dank je wel. Wat is jouw rol in de zorg voor patiënten in de palliatieve fase? Ik denk, ik begin open.**

R: Ja, een hele goede. Nou, ik zal eerst uitleggen wat mijn functie is, want ik ben geen medicus. Ik ben verpleegkundig specialist in opleiding. Ik was het, maar ik heb even geen patiëntencontact gehad, waardoor mijn registratie verlopen is. Die ben ik nu aan het terughalen. Ik werk sinds 1 augustus vorig jaar, dus 2017, hier als verpleegkundig specialist. Mijn rol is eigenlijk hetzelfde als de arts. We zijn een heel team. Ik vind zelf dat de rol van iemand die in het palliatieve team werkt is om met de patiënt mee te gaan vanaf het moment dat wij in consult gevraagd zijn, zolang als het nodig is. Dat kan eenmalig zijn, soms is het een hele weg en soms is het tot aan de dood van de patiënt. Maar dat wij die tijd meekijken met de patiënt, waarin we proberen het voor hem zo aangenaam mogelijk te maken. Ik zeg even “hem” maar dat kan ook “haar” zijn natuurlijk. Dus het voor de patiënt zo aangenaam mogelijk maken. Dat kan op allerlei gebieden. Heel vaak is dat op het gebied van pijn, dat we daarin adviseren en daarvoor ook vaak in consult gevraagd worden, om dat mee te begeleiden. Maar ik vind pijn een heel breed begrip, omdat ik onder pijn niet alleen somatische pijn versta, mensen die door een pijnprikkel pijn hebben, maar soms hebben mensen ook mentale pijn. Pas als alle pijnen op orde zijn, dan zeggen mensen vaak van: nu heb ik geen pijn meer. Je kan nog zoveel proberen, vind ik, om er pillen in te stoppen, maar als mensen mentale pijn beleven, krijgen wij het nooit goed voor elkaar om de pijn te stillen. Ja. Dus wat hier echt goed gedaan wordt op de afdeling, is naar alle dimensies van het bestaan van de mens kijken. We kijken naar het psychosociale, naar het existentiële, van, waarom ben ik hier op deze wereld, waar geloof ik in en waar haal ik mijn energie vandaan, naar de somatische klachten, van, wat zijn nou echt de pijnklachten of benauwdheidsklachten of ander soort klachten, en naar het psychosociale, van, in wat voor context leeft die patiënt, wat is zijn achtergrond, wat is zijn dagelijks leven, heeft hij nog een partner of niet, hebben ze kinderen of zijn ze juist nog kind. Dat alles maakt eigenlijk de mens compleet. Daarna kan je dan kijken van: waar zitten nou hiaten, waar kunnen wij op inspringen of hulp bieden, zodat we de mens verder kunnen helpen?

**03.03**

**I: Oké. En jouw rol daarin is eigenlijk heel breed, als ik jou goed begrijp.**

R: Echt heel breed. Die is echt heel breed. Je gaat met mensen in gesprek en je kijkt wat hun voornaamste klacht is. Mijn rol is om die voornaamste klacht eigenlijk aan te pakken. Dat is wel heel kort. Dat kan dus van alles zijn. Ja.

**I: Oké. Oké. Als je het goed vindt vraag ik dadelijk nog even wat door op die verschillende aspecten die je noemde. Is er altijd één centrale zorgverlener aanwezig bij een patiënt? Je zei eigenlijk net zelf al: we zijn een team. Voor zo’n patiënt, is er dan in dat team één vast aanspreekpunt?**

R: Nee. We proberen dat wel zo vast mogelijk te doen en daarin ben ik degene die het meest werkt, van de mensen, en één van de artsen, M. Wij werken allebei vier dagen per week, vier keer negen uur, dus wij zijn er het meest. Zo gauw als wij dan de patiënt kennen of de patiënt voor het eerst gezien hebben op de polikliniek, proberen we altijd wel dat dezelfde persoon de patiënt vervolgt. We hebben niet altijd weekenddienst en als verpleegkundig specialist hebben we zeker geen weekenddienst, dus dan is er een ander. Meestal proberen we dan wel degene al voor het weekend te introduceren, bij wijze van spreken. Dan hebben ze die alvast één keer gezien. Wat we nu doen; ik ben net bij een patiënt geweest, die zag ik voor de eerste keer. Dan zeg ik, ik zie u vandaag, omdat ik u morgen ook even kom bekijken. Dan weten zij al van…

**I: Dan herkennen ze je in ieder geval.**

R: Ja. Ja. Dus wij proberen echt wel… We zijn één team. Binnen dat team proberen we zoveel mogelijk dezelfde patiënten te zien, daar waar het lukt. En als het niet lukt, weten de patiënten ook vaak van: er komt een ander, maar die is van hetzelfde team als C. Dus dan weten ze wat ze kunnen verwachten. Ja.

**04.55**

**I: Oké. Oké. En jullie team, hoe werken jullie met andere zorgverleners in het ziekenhuis samen?**

R: Nou, wij worden eigenlijk bijna altijd in consult gevraagd.

**I: Oké. Dus jullie worden gevraagd en dan komen jullie.**

R: Ja. Dus wij zijn altijd adviserend naar de behandelaars. Wij behandelen in principe nooit zelf. Dus wat wij doen is, we kijken met de behandelaar samen of met de patiënt samen, van: wat is hier de beste behandeling? Wij adviseren dan de hoofdbehandelaar. Dus wij zullen nooit zelf beslissen van: dit moet, dat moet. We zullen dat wel sterk adviseren, maar in principe zijn zij degenen die uiteindelijk de beslissing nemen. Soms hebben we eigen patiënten liggen, om een voorbeeld te noemen, als mensen hier opgenomen worden om een bepaalde pijnbehandeling te krijgen en met “wij” bedoel ik dan het palliatieve en het pijnteam, omdat we eigenlijk onder één noemer zitten. Als mensen voor bepaalde pijnstilling komen, bijvoorbeeld voor een ruggenprik, met een kathetertje wat achterblijft in de rug, dan komen ze op onze bedden en zijn wij de hoofdbehandelaar als palliatief team. En dan beslissen wij wel.

**I: Ja, oké. En hoe werken jullie bijvoorbeeld samen met een huisarts, met de eerste lijn?**

R: Ja, heel erg veel, omdat wij zeker in de fase waarin wij de patiënten zien… Dat is vaak als er een einde van de behandeling is en wij eigenlijk de zorg moeten gaan overdragen of dat de hoofdbehandelaar de zorg moet gaan overdragen naar de huisarts. Wij hebben veel contact met de huisarts. Als wij het niet hebben vragen wij de hoofdbehandelaar altijd om eerst met de huisarts contact op te nemen voordat we zo’n patiënt bijvoorbeeld naar huis sturen. Wat ons adagium is, is dat de zorg niet af is als we niet zeker weten dat die doorgezet kan worden door de huisarts. Bij ons zal een patiënt bij wijze van spreken ook niet naar huis gaan, voordat we zeker weten dat de thuiszorg geregeld is, dat de huisarts de zorg kan leveren. Als de huisarts zegt, ja, ik wil de zorg wel leveren, maar morgen heb ik geen dienst, ben ik weg, dan zeggen we: dan houden we liever de patiënt nog een dag hier. Dus de zorg moet doorgaan. Dus we zijn heel erg veel in overleg met de huisarts.

**I: Oké. Dat is een mooi uitgangspunt.**

R: En wat wij ook doen, bij elke patiënt die wij kennen en die naar huis gaat en/of die bij ons terugkomt, of we zien hem op de polikliniek of hij gaat naar huis om te sterven; we vragen altijd, of we doen het zelf, dat we het in de brief naar de huisarts zetten, of de hoofdbehandelaar vragen we, om als laatste zin te zetten: voor de dienstdoend huisarts is het palliatief team 24 uur per dag zeven dagen in de week bereikbaar op dit en dit nummer. Dan geven wij ons nummer van de Palliatieve Zorg, omdat de artsen bij ons ook dienst draaien ’s avonds, ’s nachts en in de weekenden. En dan kennen we de patiënt ook. Dan hebben we ook zelf adviezen mee gegeven. Ze kunnen wel altijd op ons terugvallen en dat wordt ook regelmatig gedaan. Ja.

**I: Oké. En met anderen? Bijvoorbeeld met maatschappelijk werk of met pastorale hulp?**

R: Goede vraag. Ja, in principe wel. Pastorale hulp zit ook echt aan ons verbonden. Er is ook iemand van de pastorale dienst lid van ons team eigenlijk, die ook altijd bij het MDO aanwezig is. Eén keer in de week hebben we hier een MDO waarin…

**I: Waar staat dat voor?**

R: Multidisciplinair overleg. Dat is wel goed om te weten, daar zit altijd een huisarts in, die is voorzitter van het overleg. Dat is K., die zit ook in ons team, die werkt ook één dag in de week hier. Daar zit iemand van de Medische Psychologie in. Daar verwijzen wij ook weleens patiënten naar. Daar zit iemand van de thuiszorg in. Dat is ook fijn, dan kunnen we heel makkelijk schakelen en die zien dan de patiënten vaak ook vanuit hier. Het Pijnteam zit daarbij en wij als palliatief team zitten daarbij. Met maatschappelijk werk hebben we in die zin weinig contact, tenzij, als we denken van: we moeten doorschakelen… Maar dan is het meestal dat wij de hoofdbehandelaar adviseren van, goh, het zou goed zijn om daar maatschappelijk werk in te schakelen, als we zien dat óf de patiënt óf de naaste daar behoefte aan heeft. Ja.

**09.15**

**I: Oké. Gericht op communiceren, informeren en gezamenlijke besluitvorming; je noemde net al wat aspecten. Het fysieke, psychische, sociale en spirituele welzijn van een patiënt. Mijn vraag is in hoeverre die verschillende aspecten aan bod komen in de gesprekken met de patiënt. Volgens mij heb jij aangegeven: allemaal in ieder geval.**

R: Ja.

**I: Breng jij die zelf ook ter sprake?**

R: Ja. Stiekem in mijn hoofd heb ik een rijtje zitten.

**I: Oh, echt?**

R: Ja. Dat komt ook doordat wij een vast stramien hebben in onze anamnese en ook in onze overdracht. Zeker ook in het MDO willen we al die aspecten ook echt belicht hebben. Als ik in gesprek ga met mensen, dan kom ik spelenderwijs op die punten, zodat ik eigenlijk alle…

**I: Hoe werkt dat dan? Spelenderwijs.**

R: Spelenderwijs houdt in dat we soms een heel mooi diepgaand gesprek hebben, maar ik nog niet weet of die mensen gelovig zijn of niet. Dat vraag ik dan in één keer van: bent u gelovig, heeft u daar steun aan? Sommige mensen geven aan: nee, absoluut niet, ik heb niks met het geloof. En sommige mensen zeggen: ja, op een bepaalde manier geloof ik wel en daar kan ik wel steun uit halen. Dan kan ik daar de geestelijk verzorger misschien wel inschakelen. Anderzijds is het soms zo dat ik denk: goh, die mensen zitten met een hoop onbegrepen vraagstukken van het leven, maar ze zijn niet gelovig. Ook dan kan weleens de geestelijk verzorger iets doen, omdat die niet altijd vanuit een geloof praat, maar wel over levensvraagstukken in gesprek kan gaan.

**I: Je noemde dat zelf existentieel toch?**

R: Ja, ja, ja.

**I: Oké. Oké. Dus met spelenderwijs bedoel jij dat je het losjes een beetje aanvoelt in het gesprek.**

R: Ja. Ja. Klopt. Ja. Klopt. Wat mijn begin van het gesprek vaak is; als ik weet dat ik naar mensen toe ga, dat is iets heel bijzonders, dan kijk ik hier van tevoren natuurlijk altijd even naar wat die patiënten hebben, en wat ik ook doe is even kijken waar die mensen wonen. Als ik het adres weet, wat ik zie in dat systeem, dan ga ik even naar internet, dan ga ik naar Google Maps en dan zoek ik hun adres op. Dan kijk ik ook even op Street View, van: hoe ziet hun omgeving eruit, hoe ziet hun straat eruit, hoe ziet hun tuin eruit, is er een park in de buurt, is er een water in de buurt? Dat vinden de mensen heel… Dat gebruik ik echt. Als ik dan zeg van, goh, ik…

**I: “Wat een mooie vijver heeft u toch in de tuin.”**

R: Nou, dat niet, maar als mensen zeggen van, goh, ik houd echt van wandelen, dan zeg ik, gaat u naar het water daar en daar. Dan voelen zij zich acuut begrepen en voelen ze zich geen nummer of patiënt, maar dan denken ze: hé, die jongen weet hoe mijn omgeving eruitziet. Omdat ik veel fiets en de omgeving in de regel goed ken, weet ik ze vaak wel te plaatsen of ik weet iets van wat zich in die buurt afspeelt. Dan probeer ik altijd te levelen naar die patiënt. Ik merk dat ik dan heel vaak een ingang heb om…

**I: … moeilijke dingen te bespreken.**

R: Nou, dan gaat het heel snel. Dan ben je heel snel op een heel mooi niveau, waarin mensen makkelijk praten met je.

**I: Oké. Een soort van ijsbreker eigenlijk.**

R: Ja. Ja. Ja.

**12.38**

**I: Oké. En is dat bij al jouw patiënten hetzelfde?**

R: Ja, ik doe dat bijna bij alle patiënten, ja.

**I: En bijvoorbeeld patiënten die heel laagopgeleid zijn, die dingen niet goed lijken te begrijpen. Werkt dat zo ook?**

R: Dan werkt het juist heel goed. Ja, weet je? Dan hebben ze juist zoiets van: hé, die kent mijn straat en die weet waar ik woon. Het is anders dan dat ik bij wijze van spreken uit de hoogte praat of dat ik belerend praat. Dat is gewoon zonde, want dat is nu juist niet de bedoeling. Kijk, ik ben dit vorig jaar begonnen. Ik ben nu vijfenvijftig, dus op mijn vierenvijftigste ben ik nog met zoiets begonnen. Ik vind ook dat je… Dat is eigenlijk een mooie leeftijd, omdat je zelf al een hoop levenservaring hebt, een hoop dingen meegemaakt hebt, leuke dingen en minder leuke dingen. Dat is veel fijner ook voor patiënten dan dat je net van de schoolbanken komt en net als verpleegkundige afgestudeerd bent, bij wijze van spreken, en je dan met deze mensen in gesprek gaat.

**I: Waar je mee bent begonnen, dan bedoel je met het verpleegkundig specialisme palliatieve zorg, bedoel je dat daarmee?**

R: Ja. Ja. Ja. Ik denk ook wel dat je een bepaalde manier van communiceren moet hebben en kunnen als je zoiets gaat doen. Dat denk ik ook wel.

**I: Ja. Wat bedoel je daar dan mee, dat je je kunt inleven of zo?**

R: Ja, dat denk ik wel. Je zult wel een stukje empathie moeten hebben, ja. Nou moet je dat sowieso wel hebben in het ziekenhuis, denk ik, maar vooral in de palliatieve zorg, denk ik. Ja.

**I: Oké. Ja, al die aspecten eigenlijk hè, dat fysieke, psychische, sociale, spirituele, al die dingen, worden die ook vastgelegd in een zorgplan?**

R: In principe worden die vastgelegd in het dossier. We kijken aan de hand van de moeilijkheden die we tegenkomen, waarvan we zeggen, dat moet aandacht hebben; daar maken wij een plan voor. Dus daar adviseren wij een hoofdbehandelaar in. Ja. Anders in een zorgplan of in een zorgplan van de verpleegkundige; als wij vinden dat een patiënt veel pijn heeft en wij adviseren pijnstilling te geven, dan vragen wij aan de verpleegkundige: wil je pijnscores gaan afnemen, zodat wij kunnen zien wat het doet en we weten of we het de dag erna moeten gaan ophogen of moeten gaan minderen. Zo.

**I: Oké. Dus eigenlijk, al die aspecten komen daar wel in terug.**

R: Ja. Ja.

**15.08**

**I: Oké. Ik heb ook een vraag over het communiceren in het algemeen. Zijn er patiënten bij wie jij de communicatie moeilijk vindt? Ik noem het even het ijs breken, dat pak jij bij alle patiënten wel een beetje hetzelfde aan, wat je aangaf, met name patiënten die dus laagopgeleid zijn, die dingen niet zo goed begrijpen. Maar zijn er ook patiënten bij wie de communicatie moeilijk verloopt?**

R: Nee, die ben ik eigenlijk nog niet tegengekomen. Ongetwijfeld gaat het wel af en toe wat stroever of zo, maar er zijn eigenlijk altijd wel momenten dat je een ingang gaat vinden, het ijs kunt breken, dat je dan op het niveau zit waar je graag op communiceert. Natuurlijk heb je wel dat je met mensen soms wat minder diep op dingen ingaat, maar dan merk je van: hé, daar zit iets, daar willen de mensen het niet over hebben. Soms noem je dat even en als mensen aangeven van, nou, dat zit er inderdaad, maar daar wens ik het niet over te hebben, dan is dat prima. Ik kan aangeven van: goh, weet dat u het wel kunt en als er op een later moment behoefte aan is, dan zijn we er voor u. Dan laat ik het verder ook gaan. Maar tot nu toe heb ik gesprekken gehad met… Ja, soms zit je met een ouder van een kind van een jaar of zeven, waarbij het kind gaat overlijden op korte termijn, tot en met mensen van vierennegentig of bijna honderd. Met alles wat daar tussenin zit heb je gesprekken. Je moet constant schakelen. Je moet de hele tijd zelf even schakelen. Het jongste kind, waar ik zelf een gesprek mee heb gehad, was vijftien jaar. Dat was een heel bijzonder gesprek. Dat was een vrij introvert kind, maar op een bepaalde manier kreeg ik zijn aandacht en dat was echt een prachtig gesprek. Maar de meesten zijn ouder. Soms tref ik ook patiënten, die zijn net zo oud als mijn kinderen. Dus je bent altijd toch ook wel met je eigen zijn bezig. Van daaruit praat je, denk ik. Ja.

**I: Ja, dat kan ik me wel voorstellen. Oké. Je zegt dat je bij de jongen van vijftien goed binnenkwam, bij die introverte jongen. Maar hoe heb je dat dan voor elkaar gekregen? Kun je in het algemeen manieren noemen die je…**

R: Ja, maar dat is weer het empathische vermogen, denk ik. Zonder mezelf op de schouders te kloppen, want dat wil ik absoluut niet. Ik heb het idee dat ik me redelijk goed kan inleven vaak in hun wereld. Als je dan op dezelfde manier gaat praten als zij; zo’n jongen van vijftien was aan het gamen. Die had zijn computer nog open staan. Ik wist dat die binnen een maand zou gaan overlijden. Die had zelf eigenlijk nog niks in de gaten. Nou, ik denk het wel, maar ja, hij was ook echt de hele tijd aan het gamen. Dat was ook zijn ding. Vijftien en echt ziek zijn, dan is dat je wereld. Dus ik begon langzaam opbouwend te praten en op een gegeven moment zag ik dat hij zijn oortje uitdeed. Toen dacht ik, nu heb ik wat meer ingang. Op een gegeven moment klapte hij zijn computer dicht. Toen dacht ik, nu heb ik…

**18.20**

**I: Ja, maar hoe heb je dat dan gedaan? Echt dat aanvoelen?**

R: Ja en door op zijn niveau dingen te gaan uitleggen. Ik moet daar niet over bepaalde levensvragen gaan praten, maar meer over zijn leeftijdsgenootjes en wat hen bezighoudt, met wie hij speelt op de computer. Hij had ook lotgenoten al verloren. Hij had een bijzonder zeldzame ziekte en was aan het gamen af en toe met zijn lotgenoten. Maar hem waren er al twee ontvallen van de vier. Er was er nog eentje over, zal ik maar zeggen. Dus als je zo begint… En wat ik wel geleerd heb of dat zit in mij, is dat ik nooit om de hete brij heen draai. Ik zeg altijd waar het op staat. Ik ga nooit liegen. Ik heb tegen deze jongen ook gezegd van: wat zou je fijn vinden om te doen, want ik denk dat je dat snel moet gaan doen, want ik denk dat je snel dood zult gaan. Toen klapte hij dicht. Hij klapte niet dicht qua emoties, maar toen kreeg ik zijn aandacht. Toen dacht hij: hé, dit gaat een andere kant op. Ik merk wel, ik krijg heel vaak reacties terug van mensen die zeggen: wat fijn dat je zo open tegen ons bent en dat je nooit om de hete brij heen draait, want dat is ons al zo vaak gebeurd, dat mensen ons niet precies zeggen wat ze… Die mensen hebben zo’n behoefte aan duidelijkheid. Ze hebben er niks aan om zoete broodjes te bakken. Dat heeft in deze fase van het leven geen zin meer. Dat is ook eigenlijk niet fijn meer. Dan weten ze ook… Natuurlijk kan ik ook nooit zeggen hoelang het duurt. Dat is *de* vraag van mensen, van: hoelang denkt u dat ik nog leef? Ja, dan zeg ik: die glazen bol heb ik niet. Gelukkig heb ik hem ook niet, want dan weet ik zelf ook dingen die ik liever niet zou willen weten waarschijnlijk. Maar je gaat er ook niet omheen draaien. Ik begin ook vaak mijn…

**I: Dat zijn wel twee hele belangrijke aspecten eigenlijk...**

R: En wat ik vaak doe is dat ik begin bij mensen; als ik binnenkom zeg ik, mijn naam is H., ik ben van de Palliatieve Zorg. Mensen vinden het soms heel lastig om met de Palliatieve Zorg te gaan praten, omdat ze denken: dit is het laatste stukje.

**I: Dat is een drempel natuurlijk. Ja.**

R: Heel soms als ik dat echt weet, dan zeg ik, nee, maar ik ben van de ondersteunende dienst. Uiteindelijk weten ze aan het eind van het gesprek wel dat ik van de Palliatieve Zorg ben, maar dat komt tijdens het gesprek wel. Wat ik altijd doe is, ik geef ongeveer een meter op de tafel of op het bed aan en dan zeg ik: aan het begin van die meter heeft u te horen gekregen dat u ongeneeslijk ziek bent, hier gaat u overlijden en ergens tussenin komen wij op uw pad. Soms is dat helemaal in het begin, omdat u veel pijn heeft, en dan gaan we met u mee. Soms is dat in het midden, maar soms is dat ook al hier en regelmatig is het hier aan het einde. Soms bijna aan het einde, maar dan zijn mensen eigenlijk al haast niet meer aanspreekbaar. Dan heb je ook meer het gesprek met de familie dan met de patiënt zelf. Maar ergens zit dat ertussenin. We gaan samen dat pad op naar het uiteinde toe. Als je dat uiteinde al bespreekt of een keer laat vallen, dan kun je later ook zeggen van: ik heb daarstraks het woord “uiteinde” genoemd en daarmee bedoel ik het overlijden. Heeft u daar weleens over nagedacht, over het overlijden? Zo begin ik eigenlijk het gesprek. Zo breek je het open. Soms merk je dat mensen, partners onderling, het heel lastig vinden om het over het overlijden te hebben, maar het is oh zo belangrijk, want als ze dat niet zouden doen, dan missen ze een stukje. Zo gauw als het opengebroken is, kunnen zij zoveel meer de volgende stap gaan maken. Dat vind ik eigenlijk één van de belangrijkste taken van mij in gesprekken met mensen die toch richting het einde van het leven gaan, dat ik hen de volgende stap kan gaan laten maken, zodat de brok in de keel weg is en ze rust gaan krijgen om het laatste stukje van het leven in te gaan, waardoor ze nog heel veel wezenlijke dingen belangrijk kunnen gaan vinden en daar aandacht aan kunnen besteden, zonder bang te zijn voor de dood. Als ik kan uitleggen hoe die dood eruit zal gaan zien ongeveer, dat ze niet hoeven te lijden, dat we er daar zoveel mogelijk voor hen zullen zijn, dan is die angst weg. Mensen zijn vaak niet bang voor de dood, maar wel voor de weg ernaartoe, omdat mensen bang zijn voor pijn of voor lijden. Maar als we kunnen aangegeven van, er zijn heel veel mogelijkheden waardoor we dat zachter kunnen maken en er voor u kunnen zijn, dan valt dat stuk weg en dan kunnen we het over een ander stukje hebben. Dus zo.

**I: Oké. Jeetje, wat mooi. Ja.**

R: Ja, dat vind ik ook. Dat zeg ik ook heel vaak, ik vind elk gesprek een cadeautje. Als ik soms hoor wat ik van mensen mag horen, wat er in hun leven gespeeld heeft… Sommige mensen spreken tegen mij dingen uit die bij wijze van spreken alleen zij en de huisarts weten en verder niemand. Dan denk ik, jeetje, dat ze dat soort dingen met mij delen is wel heel bijzonder. Dat is fijn voor die mensen. Ik merk, dan zijn ze het kwijt en dan kunnen ze alsnog... Als ik daar nog even op inga of zo, dan is dat voor hen zo fijn en bevrijdend en dan zijn ze klaar voor het volgende stuk. Dat vind ik echt mooi. Ik word er wel hondsmoe van soms. Ondanks dat ik er echt moe van word, dus geestelijk moe…

**I: Ja, dat kan ik me voorstellen.**

R: Dat je thuis bent en dat je om acht uur of negen uur echt zit te knikkebollen. Dat is echt niet van de inspanning maar dat is meer van de geestelijke inspanning… Elk gesprek gaat over de dood, bij wijze van spreken.

**I: Ja. Het is geen lichte kost.**

R: Nee, maar het geeft me altijd energie. Als ik naar huis fiets, dan denk ik wel na even over gesprekken. Ik blijf er niet mee zitten. Het houdt me niet bezig. Sommige dingen wel, maar dat mag ook denk ik wel. Zo’n kind wat ik noemde blijft je echt bij. Maar goed, dat geeft mij heel erg veel energie en ik ben blij dat ik hier ben gaan werken. Ja.

**24.03**

**I: Oké. Je maakte zelf net heel mooi een tijdlijn. Als je hem een beetje anders bekijkt; je hebt hier die vijftienjarige jongen. Je gaf net ook aan: ik heb ook mensen van vijfennegentig die in die fase zitten. Bij die groep, die vijfennegentigjarigen, check je ooit of ze jou begrepen hebben? Dat is puur gericht op communiceren hè? Ik kan me voorstellen; die kant van de zaak, die jongen, die zal je ongetwijfeld begrijpen. Maar zijn er ook nog manieren om daarmee om te gaan?**

R: Ja, ik denk het wel. Nou, ten eerste, als ik ook maar enigszins de twijfel heb dat ze me niet begrepen hebben, dan spreek ik al heel snel af om hen op hele korte termijn weer te spreken, dat ik even bel of even op een andere manier contact heb, zodat ik weet van: het is aangekomen, het gesprek, wat ik verteld heb, wat ik voorstelde aan medicijnen. Als ik daaraan twijfel zeg ik: u gaat nu naar huis, u weet wat ik gezegd heb. Vaak zijn er ook kinderen bij, zeker bij mensen van die leeftijd, dan zijn er kinderen bij. Dan heb ik wel door… Of ik vraag soms van: heeft u het begrepen, kunt u herhalen wat ik bedoel? Dat is eigenlijk vaak een mooie manier om te kijken of ze het begrepen hebben. Maar als ik denk van, ze hebben het nu begrepen, maar daarna niet, dan geef ik wel aan van: als u het goed vindt, dan bel ik ook eventjes de huisarts met mijn verhaal en ik schrijf het even op…

**I: Oh ja, wat je in het begin ook al aangaf.**

R: Ja, maar dat hoort ook zo, vind ik. Ik ben ook echt van mening – en dat is ook wel waar wij voor staan in het team – dat die huisarts de spin in het web is eigenlijk. Daar moeten zij ook terugkomen als er vragen zijn of als ze weer pijn hebben tussendoor. Natuurlijk mogen ze ook bij ons terugkomen, maar bij die huisarts moeten ze ook terecht voor bijvoorbeeld recepten. Als die huisarts niet weet wat ik aan het doen ben, dan zal die denken: ja, hoe kan dat nou, toen ik hem de laatste keer zag had hij maar 2 mg morfine en nu heeft hij 10 mg morfine. Waar heb ik iets gemist? Dus dat ben ik verplicht aan de huisarts, want dat is degene die de regiefunctie kan hebben. Ik zeg dan ook tegen die huisarts van: volgens mij hebben ze het niet goed begrepen, dus wil je het een beetje in de gaten houden? Dus dat zijn wel manieren om te kijken of ze het begrepen hebben. Ja.

**26.20**

**I: Oké. Ja. En in die zorg in de palliatieve fase, in hoeverre is er sprake van gezamenlijke besluitvorming, denk je?**

R: Nou, gezamenlijk… Ik vind eigenlijk… We leggen meestal… Nou, niet meestal, maar we geven wel heel erg de patiënt de ruimte om zijn beslissingen te nemen. Dat is eigenlijk de regisseur. We kijken wel heel erg naar de wil van een patiënt. De wil van de patiënt moet wel heel gek zijn willen we zeggen: ja, maar dit gaat echt niet, dit strookt niet. Ja, dan kun je wel zeggen shared decision, maar de patiënt staat echt vooraan. Natuurlijk sturen we weleens, maar we merken dan wel dat de patiënt daar behoefte aan heeft. Vaak schetsen we diverse scenario’s en dan mag hij of zij de keuze maken. Om een voorbeeld te noemen, heel veel mensen praten met ons en men wil steeds meer weten wat euthanasie is. Die willen misschien wel euthanasie voorstellen bij de huisarts. Dat komt omdat ze niet willen lijden op het laatst. Dan leggen we precies uit wat euthanasie inhoudt en hoe dat gaat, dat er allerlei regels aan verbonden zijn, dat dat prima kan, dat het de wens van de patiënt is. Die moet het verzoek doen uiteindelijk aan de huisarts of aan de hoofdbehandelaar, maar dat er ook zoiets is als palliatieve sedatie, dat als mensen echt last krijgen in het laatste stukje van het leven, dat ze geen pijn hoeven te lijden of niet benauwd hoeven te zijn, dat als het overlijden eraan zit te komen, dat er dan ook andere mogelijkheden zijn voor een huisarts om in ieder geval medicatie te geven, zodat ze die klachten veel minder hebben of dat die klachten zelfs misschien helemaal weg zijn. Meestal zeggen mensen dan: oh, maar dan hoef ik ook geen euthanasie. Als ze dat maar weten. Van de andere kant zeggen we: nou, weet je, bespreek met de huisarts euthanasie. Als je nou echt zegt op het laatste moment, als de huisarts het wil en jij wilt het graag, dat je dat in ieder geval als uitlaatklep kunt hebben… Als het nou echt niet gaat, dan heb je dat besproken, dan is dat pad gevolgd. Je kunt op het laatst altijd beslissen van: nou, laat maar, ik vind het zo goed, zoals het nu gaat, ik heb weinig pijn, laat me maar langzaam overlijden en dan vind ik het helemaal goed. Maar dan heb je dat in ieder geval besproken. Dus mensen mogen toch zelf die keuze wel maken.

**I: Ja, precies. Je gaf aan dat jullie soms wel wat sturen. In wat voor gevallen is dat? Is dat bij per patiënt verschillend?**

R: Ik denk toch wel dat dat verschilt. Kijk, soms willen mensen ook weten natuurlijk wat hun levensverwachting nog is als ze het een doen of het ander. Als ze bijvoorbeeld wel meedoen aan een studie waar ze medicijnen krijgen voor een behandeling voor kanker of niet. Ja, daarin kunnen we niet sturen. Dat vinden we echt iets van de hoofdbehandelaar. Dus in die zin geven we dat weer terug van: dit moet u echt met de hoofdbehandelaar gaan bespreken. Die weet ook vaak meer van werking en ook bijwerkingen van bepaalde medicijnen. En ik moet wel heel eerlijk zeggen dat ik soms wel denk, als mensen echt heel veel klachten hebben gehad van hun medicijnen en ze zitten redelijk bij het laatste stukje van het leven en hun klachten zijn er veel meer tijdens medicatie dan als ze niks meer zouden doen; dat ze dan voor het laatste stukje van het leven kiezen voor kwaliteit, zonder alle bijwerkingen van medicatie, dat ik weleens aangeef van: goh, waar doe je het dan nog voor? Doe je het voor de wetenschap of zeg je van: daar heb ik nu heel veel voor gedaan, maar ik wil nog een stukje kwaliteit nastreven. Dan ben je eigenlijk een beetje sturend van: nou, volgens mij moet je dat laatste stukje gaan doen. Dan help je hen wel om die beslissing te nemen, maar uiteindelijk zijn zij degenen die beslissen.

**30.14**

**I: Ja. Oké.**

R: Hoelang hebben we nog:

**I: Ik keek al, die klok, klopt die?**

R: Geen idee.

**I: Volgens mij niet. We zijn een half uur aan het praten, dus ik ga even een beetje gas geven of in ieder geval, ik ga eventjes zelf…**

R: Hoelang hebben we?

**I: Ik had drie kwartier geloof ik. Ik heb hier een aantal kaartjes. Wettelijke vertegenwoordiging in de situatie van acute verslechtering. Dat is de langste. Wilsonbekwaamheid en levenseindebeslissingen. Levensbeschouwing en culturele achtergrond. Plaats van zorg en sterven. Ziekenhuisopnames. Crisissituaties. Niet-behandelafspraken. Nu is mijn vraag of je misschien met behulp van een rangorde kan aangeven in hoeverre deze onderwerpen aan bod komen in gesprekken met patiënten.**

R: Nou, deze komen aan bod als dat zich voortdoet, wettelijke vertegenwoordiging in situaties van acute verslechtering, maar niet in die zin dat dat vaak onderwerp van gesprek is. Niet zo vaak. Moet ik die dan wegleggen?

**I: Of aan één kant.**

R: Wilsonbekwaamheid en levenseindebeslissingen, dat komt vaak aan bod. Dat heb ik je net gezegd: als mensen echt voor euthanasie kiezen dan zeggen wij ook altijd: je moet wel helder van geest blijven in dat laatste stukje. Als u de wens heeft voor euthanasie, dan moet je dat in principe tot het laatst kunnen uiten aan de huisarts of aan degene die de euthanasie… Dus dit komt vaak wel aan bod. Crisissituaties ook vaak. Kijk, tot voor kort, als mensen dan een crisissituatie meemaakten, wat weleens gebeurde voordat ze hier kwamen, dan belden ze 112. Maar wat wij vaak bespreken is van: goh, is het niet beter om voortaan de huisarts te bellen? Als u zo ziek bent en u gaat overlijden op korte termijn, dan is het de vraag of dat u nog wil dat in een crisissituatie 112 gebeld wordt.

**I: Dat de ziekenauto komt.**

R: Precies. “Want als u dat doet, als u 112 belt, dan komen ze en dan gaat u alles doen wat u misschien niet meer wilt. Dus zorg dat u met de huisarts afspreekt: in een crisissituatie bel ik u op als er iets aan de hand is of ik ga zelf handelen.” Sommige mensen krijgen een forse bloeding. Dan kunnen ze mensen thuis al vast wat medicijnen geven, zodat de patiënt rustig is of misschien wel inslaapt. Dus dat bespreken we echt. Dat moet ook, denk ik, want dan zijn ze ook voorbereid. Dat geldt ook voor ziekenhuisopnames. Wat willen ze nog? Willen ze nog naar het ziekenhuis of niet?

**I: Of thuisblijven…**

R: Of thuisblijven. Dus ook de plaats van zorg en sterven komt altijd aan bod. Wij vragen ook altijd: wilt u naar huis, wilt u naar een hospice, wilt u in het ziekenhuis blijven? Dat ligt ook een beetje aan de tijd die… Als mensen echt heel snel achteruitgaan; sommige mensen zeggen: laat me maar lekker hier, dan heb ik alle zorg die er is, als ik hier kan blijven is dat goed. Als mensen een wat langere levensverwachting hebben van een paar weken of zelfs maanden, dan kunnen ze niet in het ziekenhuis blijven, want in principe is het ziekenhuis er voor mensen die nog beter kunnen worden of behandeld worden en niet voor mensen die sterven. Dan vragen we via het transferpunt een plaats aan waar ze kunnen sterven, met thuiszorg thuis of in een hospice. Levensbeschouwing en culturele achtergrond komt eigenlijk ook altijd aan de orde. Dat is één van die domeinen waar ik dingen over vraag, van: goh, hoe staat u in het leven, heeft u steun aan een geloof of is dat er geweest? Culturele achtergrond of de achtergrond van, wat hebben mensen gedaan in hun leven; vaak praten we met mensen die al gepensioneerd zijn. Toch is het fijn om even over hun werk te praten of over wat hen bezighoudt, zulk soort dingen, wat hun hobby’s zijn. Dat hoort bij ons bij de psychosociale as. Behandelafspraken, niet-behandelafspraken. Dat komt vaak ook wel tot uiting, omdat we het met mensen hebben over: goh, wat wenst u nog, moeten we kijken naar medicijnen die voor u nog wel van levensbelang zijn of niet meer? Sommige medicijnen, zal je je ook wel kunnen voorstellen… Om een voorbeeld te noemen, mensen die een paar maanden te leven hebben en die nog cholesterolverlagers slikken; de cholesterolverlager die je nu slikt is over tien jaar pas voordelig.

**I: Dus die hoeven we niet meer te nemen…**

R: Die kunnen we gewoon stoppen. Dus wij kijken echt naar… Maar bijvoorbeeld ook voeding. Als mensen tot op het laatst toe sondevoeding krijgen, dat zit hun vaak alleen maar in de weg. Mensen zeggen: ja, maar ik heb helemaal geen honger meer. Zeker mensen die echt op het laatst zijn, bij hen geven wij ook aan van: ja, vaak zeggen mensen, ja, maar als je niet eet ga je dood, maar u eet niet meer, omdat u doodgaat. Dat is even heel confronterend, maar daarna zoveel fijner, omdat dan ook de mantelzorger denkt van: oh, dus ik hoef het er niet meer maar in te proppen. Wij zeggen ook vaak: eet vooral wat u lekker vindt en als dat een waterijsje is of een biefstukje of een frikadel speciaal, doe dat dan vooral, maar ga niet constant die flesjes energydrank drinken, omdat je denkt: ja, maar anders sterft mijn man of vrouw. Wettelijke vertegenwoordiging in een situatie van een acute verslechtering; dat is echt alleen zo als er geen echtgenoot is of geen kinderen. Ja.

**35.52**

**I: Oké. Oké, duidelijk. Ja, dan ga ik nog even door naar het laatste hoofdstuk, even kort. Dat gaat over beperkte gezondheidsvaardigheden. Jij kent die term natuurlijk.**

R: Ja.

**I: Wat betekent die term volgens jou? Beperkte gezondheidsvaardigheden.**

R: Nou, volgens mij betekent het dat mensen niet in de gaten hebben wat goed of fout is voor hun lijf, of dat ze nou gezond zijn of ziek zijn, maar dat is in algemene zin wat mensen niet goed kunnen onderscheiden.

**I: Ja, zeker. Mooi.**

R: Volgens mij maakt het namelijk ook niet uit of mensen slim zijn of niet slim zijn of goed geletterd, ongeletterd… Dat maakt niet uit. Mijn voorstel was voor jullie om een patiënt te filmen…

**I: Is dat morgen toevallig?**

R: Nee. Nee, deze patiënt is overleden, die ik nu bedoel. Maar dat was een professor. Die kon zo absoluut niet met dit soort dingen omgaan, terwijl het echt een hoogleraar was, een bijzonder begaafd mens. Maar hij had echt geen gezondheidsvaardigheden. Hij wist niet hoe hij nou met zijn ziekte moest omgaan of met zijn medicatie, dat kon hij niet, maar op natuurkundig vlak wist hij alles. Dus zo denken we ook weleens. Dus daar moeten wij ook altijd aan denken, van: wie hebben we voor ons? Sommige mensen die laaggeletterd zijn kunnen prima opnoemen waarom ze niet mogen roken of bepaalde soorten medicijnen moeten nemen. Dat doen ze heel erg goed. Soms kunnen ze het niet onderbouwen. Soms kunnen ze het wel onderbouwen, dat ze denken van: ja, maar dat is heel belangrijk voor mij. Het heeft niet zozeer met intelligentie te maken volgens mij, maar meer met andere zaken.

**I: Oké. En hoe vaak heb jij daarmee te maken, met dat soort patiënten?**

R: Niet zo vaak. Als ik een schatting nu moet maken is dat tien procent of zo, denk ik.

**I: Oké. Oké. En hoe herken jij die? Dat lijkt me heel lastig namelijk.**

R: Dat is ook heel lastig. Ik denk, een goede verstaander heeft soms blikken nodig of een woord of een zin nodig of een blik van de mensen die ernaast zitten. Als de patiënt “ja” zegt, maar degene die ernaast zit schudt stiekem “nee”, dan weet ik eigenlijk al van: hé, hier klopt iets niet. Dus verbaal en non-verbaal gedrag zegt heel veel in een gesprek, denk ik. Zo probeer ik wel te… Dan is het goed om nog eens even… Als ik de mantelzorger die ernaast zit stiekem “nee” zie schudden terwijl de patiënt “ja” zegt, dan probeer ik daar nog eens een keer naar te vragen door nog eens een keer te zeggen van: goh, maar klopt het echt wel wat u zegt?

**I: Dus je houdt er wel echt rekening mee?**

R: Ja. Ja. Ja.

**38.44**

**I: Oké. Je gaf aan in het begin dat je empathie heel belangrijk vond, juist bij die groep eigenlijk.**

R: Ja.

**I: Zijn er nog meer manieren waarop jij mondeling of schriftelijk je communicatie aanpast?**

R: Mijn?

**I: Je communicatie aanpast op die patiënt?**

R: Anders dan mondeling of schriftelijk?

**I: Nou ja, bijvoorbeeld, patiënten die dingen niet zo goed begrijpen, daar is empathie juist heel belangrijk.**

R: Ja, maar empathie moet je denk ik niet verwarren met onduidelijkheid of duidelijkheid. Ik kijk wel hoe ik zo duidelijk mogelijk voor die mensen ben.

**I: Ah zo, en dat is het ‘niet om de brij heen draaien’, wat je zei?**

R: Nee, nee. Maar bij sommige mensen weet ik precies dat ze het heel makkelijk aannemen en daar hoef ik bij wijze van spreken weinig aandacht aan te besteden. Dan kan ik over hele andere zaken praten en weet ik toch wel dat het goed zit. Mensen van wie ik zeker weet van, ja, maar die hebben me niet goed begrepen, als ik daar niet nog een keer op terugkom, over medicatievoorschrift bijvoorbeeld; dan zorg ik wel dat ik daar toch nog een keer op terugkom, twee of drie keer in een gesprek, dat ik zeg: weet u nog, hebt u mij goed begrepen? Daar kan ik ook mee afsluiten, want dat vind ik wel heel belangrijk.

**I: Ja, dus eigenlijk die herhaling.**

R: Die herhaling. Ja. Dat ik het herhaal, maar bij wijze van spreken dat zij het ook nog een keer herhalen.

**I: Dus dat ze ook nog een keer tegen jou zeggen van: dit is wat je…**

R: Ja. Ja. Ja.

**I: En zijn er meer van dat soort strategieën die je gebruikt?**

R: Ik herinner me van één of twee patiënten dat ik het even op heb geschreven, van: ik leg u nu dit uit, ik schrijf even op wat ik bedoel. Dan kunnen ze dat mee naar huis nemen. Zo is het voor hen ook makkelijk. Of ze hebben zelf een blaadje staan waarin ik meekijk. Heel veel mensen doen dat ook, die schrijven even precies op wat ze nodig hebben. Dan kijk ik samen met hen even in hun lijstje, van: klopt het of klopt het niet? Dat is hun harde schijf. Ja.

**40.48**

**I: Ja. Oké. Ja, mooi. Had je nog iets toe te voegen aan mijn vragen?**

R: Nee. Volgens mij heb ik wel alles gezegd. Ik praat graag, dat merk je wel.

**I: Ja, dat is fijn voor mij.**

R: Ja. Zeker. Ja. Nee. Nee, ik ben blij dat we het in een team doen en mijn rol, misschien is dat nog wel iets om op terug te komen, is verpleegkundige en ik ben nog in opleiding, dus er zit altijd een arts achter mij, met wie ik mijn voorstellen bespreek. Uiteindelijk is dat het plan dat wij adviseren, dus het is een gezamenlijk plan. Ik schrijf ook altijd in mijn adviezen, in ieder geval zolang als ik niet een gediplomeerd verpleegkundig specialist ben: in overleg met die en die arts. Dat weet de huisarts dan ook. Dat weet ook degene die dienst heeft of degene die mijn rapportage leest, van: C. heeft dat gedaan in overleg met die en die arts. Dat is voor mezelf, dat ik mezelf indek, maar het is ook wel goed voor degene die het leest om te weten van: hij heeft in ieder geval overleg gehad met één van de artsen. Want zelf mag ik nog niks. Nou, dat is niet waar, maar…

**I: In de formele zin.**

R: In de formele zin.

**[43.25 – einde opname]**

Z4d02

I: interviewer

R: respondent

**00.46**

**I: Oké. Wat is jouw rol voor patiënten in de palliatieve fase?**

R: Dat is altijd zo’n hele lekkere algemene.

**I: Hij begint heel breed.**

R: Ik werk als verpleegkundig specialist in opleiding, verpleegkundig consulent bij de Palliatieve Zorg. Wat wij vooral doen zijn intakes, vervolggesprekken, in het algemeen een beeld vormen van de patiënt in de breedste zin van het woord. Ik denk dat de arts bij het medisch-inhoudelijke stukje altijd heel sterk betrokken is. Het psychosociale, zeker het sociale stukje is denk ik de focus basaal van de verpleegkundige. Daar zit denk ik wel een verschil in. Het idee is wel dat ik als verpleegkundig specialist in opleiding iets meer richting het medische stuk schuif.

**I: Oké. Ik heb gisteren H. gesproken.**

R: Ja. Die zit in precies dezelfde rol. Die is ook VS i.o. Alleen heeft hij de opleiding al achter de rug. Hij is nu stukjes aan het overdoen voor een soort herregistratie en ik ben bezig met de opleiding.

**I: Oh, dat zei hij, ja. Ja.**

R: En ik ben bezig met de opleiding die hij een aantal jaar terug gedaan heeft. Het grote verschil is denk ik dat H. vanuit de IC komt en ik al tien jaar, vanaf 2004, binnen dit team functioneer. Onze insteek of onze uitgangspositie is iets anders geweest.

**I: Oké. Vanaf 2004 zei je hè? Dus jij dacht: ik wil toch nog een opleiding gaan doen. Dat is heel ambitieus…**

R: Ja, dat denk ik af en toe ook. Ja.

**I: Het is wel bijzonder.**

R: Ja. Het kost me ook heel veel energie.

**I: Ja, dat kan ik me voorstellen. Je hebt twee banen dus eigenlijk, een soort van.**

R: Nou, nee, nee, nee. Degenen die de opleiding doen zijn allemaal mensen die vierentwintig tot tweeëndertig uur werken in de praktijk en daarnaast de opleiding doen. Dat is een eis vanuit de opleiding. Dus je hebt gewoon een opleidings-dag en daarnaast moet je een heleboel dingen doen ook in de praktijk. Dat ben ik niet meer gewend ook.

**I: Oké, oké. Nou, dan ga ik snel door naar de volgende vraag, want je hebt het dus heel druk. Je gaf aan: eigenlijk van A tot Z, van intake tot einde, ben je bij de patiënt.**

R: Ja. Ja.

**I: Is altijd een centrale zorgverlener aanwezig voor de patiënt?**

R: Als je daarmee de hoofdbehandelaar bedoelt, dan is dat meestal een zaalarts, een verpleegkundige op de afdeling. Wij zijn zelden hoofdbehandelaar als palliatief team. Uitzonderingen daar gelaten zijn wij consulterend en geven wij advies aan. Voor sommige patiënten; vandaag ligt er bijvoorbeeld eentje op de Medische Oncologie. Die heeft een keertje op de Dagbehandeling gelegen. Die heeft een keer op de Chirurgie gelegen. Dan zijn we geen hoofdbehandelaar, maar doordat we door het hele ziekenhuis komen, hebben we deze meneer wel al op de poli gezien. Ik heb hem op vier verschillende plekken al gezien. In dat opzicht ben je wel voor deze meneer een bekend gezicht, zo langzamerhand. Dat merk je ook. Die mensen denken van: hè…

**I: “Jou heb ik al ooit gezien.”**

R: Ja. Dat vind ik één van de leuke kanten daaraan. Ja.

**04.38**

**I: Oké, ja. Dus dan neem ik aan dat je ook heel veel met andere zorgverleners moet samenwerken.**

R: Ja. Ja.

**I: Hoe werkt dat?**

R: Ja… Het fijne is dat je gevraagd wordt hè, dus mensen willen iets van je. Dat vind ik het fijne. Het lastige daarvan is natuurlijk ook: als je niet gevraagd wordt, blijf je ook buiten beeld. Je hebt gewoon geen enkel zicht op wat hier voor de rest in het ziekenhuis gebeurt.

**I: Terwijl het misschien toch wel mooi had kunnen zijn bijvoorbeeld.**

R: Ja. Ja. Ja. En dat weet je niet. Je bent afhankelijk van of je gevraagd wordt en dat is lastig soms.

**I: Oké. Zijn er wel momenten dat jij zoiets had van: nou, hier hadden wij eigenlijk wel bij kunnen zijn of bij moeten zijn of zo? Of is dat lastig om te…**

R: Ja, dat vind ik natuurlijk lastig om te zeggen, want als ze mij niet vragen, dan zie ik het niet. Laat ik het zo zeggen: niet zo lang geleden heb ik nog ergens een praatje gehouden van: goh, wanneer mogen jullie het palliatieve team vragen? Dan zie je in zo’n bespreking dat er verpleegkundigen zitten en artsen niet. Wat de verpleegkundigen dan eigenlijk vooral zeggen is: de dokter wil niet en de dokter moet wel dat orderen. Je krijgt het wel terug, dat mensen daar tegenaan lopen, maar ja, dat gebeurt buiten mijn gezichtsveld. Maar dat is wel de eeuwige discussie binnen het ziekenhuis. Dat was tien jaar terug al en dat is het nog steeds. Ik denk dat dat ook nooit weg zal gaan, helemaal.

**I: Oké. Maar vanaf 2004… Dat is eigenlijk een beetje vanuit persoonlijke interesse, maar heb jij dan niet zoiets dat de palliatieve zorg serieuzer wordt, dat dat meer aandacht krijgt, dat jullie team misschien ook groter is, of zo?**

R: Jawel, jawel. In 2004… Ik ben ooit begonnen op de Medische Oncologie. Toen had je één arts, één verpleegkundige. Ik wist eigenlijk maar amper dat er een palliatief team was. Je had het IKNL, het Integraal Kankercentrum, die wat aan palliatieve zorg deed en ook van daar uit alle ziekenhuizen bediende. Dus er was überhaupt niets in het ziekenhuis. Ik ben ook begonnen met een detachering naar het IKNL zonder dat er hier een palliatief team was. Op een gegeven moment zijn er een paar mensen; R. was de eerste die vanuit het… Die hadden toen vanuit het IKNL een soort aandachtsveld … (naam ziekenhuis). Ik bleef wel in dienst van het … (naam ziekenhuis). Dat was voor mij wel een groot voordeel. Ik denk dat in het begin zelfs… Ja, P. en S.; S. deed dat eigenlijk buiten zijn normale werk om, omdat hij hart had voor de palliatieve zorg, maar er was überhaupt niets. En nu zie je dus fulltime iedere dag een verpleegkundige en iedere dag een arts die dit gewoon fulltime mag doen. Dat is natuurlijk toch op een andere manier dan hoe wij ooit begonnen zijn. Ja.

**I: Ja. Dus daar zit op zich wel een positieve beweging in.**

R: In dat opzicht wel. Sowieso, de hele onderzoek poot is daarna pas ontstaan. Met C. is hier wel een hele grote… Die heeft de palliatieve zorg veel belangrijker gekregen, het heeft ook veel meer een gezicht gekregen. Wij zaten toentertijd überhaupt niet in dit ziekenhuis. We zaten ergens op achthoog in een bejaardenhuis hier schuin tegenover, dus we waren ook letterlijk niet zichtbaar. Dit is wel een hele andere situatie dan toentertijd hoor. Jawel. Daar kun je je nu niks meer bij voorstellen, maar…

**08.31**

**I: Nee, inderdaad. Ja. Dus die samenwerking met die partijen; werken jullie bijvoorbeeld ook veel samen met huisartsen?**

R: Ik vind het vanuit het … (naam ziekenhuis) relatief weinig. Er zit heel duidelijk een focus van: als een patiënt naar huis gaat of voordat hij naar huis gaat, zorg dat die huisarts gebeld is. Dat is niet een automatisme dat bij alle artsen erin zit, zo van: dat hoort. Hij krijgt een brief. Dat gaat wel een stuk beter. Een brief gaat gewoon veel sneller met het huidige systeem dan vroeger. Dat was eerst uittypen en dan op de post en dan kwam hij echt twee weken later aan als je geluk had. Met al die digitale systemen is toch vaak een dag of een dag later die brief eruit. Ik denk wel dat wij, ik wil niet zeggen bij honderd procent, maar bij negentig procent van de patiënten toch wel zeggen van: zorg dat die huisarts gebeld is voor deze patiënt naar huis gaat, zodat die weet wie er naar huis komt en wat die moet doen. De palliatieve patiënten zijn natuurlijk toch mensen die extra zorg nodig hebben en vaak al meteen bij binnenkomst of als ze het ziekenhuis verlaten. Dus ik denk dat daar veel meer aandacht voor is gekomen. Vanuit het IKNL is er een heel duidelijke poot met huisartsen. Het Integraal Kankercentrum biedt consultaties aan hulpverleners en dan vooral voor de patiënten die niet bekend zijn bij het palliatieve team hier van … (naam ziekenhuis). Ik geloof tachtig procent van de consulten naar het IKNL toe komt vanuit huisartsen. Dus daar zit die link nog veel sterker dan binnen het ziekenhuisteam. Ik werk één dag via het IKNL, dus vandaar dat voor mij die link veel sterker zit. Ja. Ja. Als je als ziekenhuisteam mensen verkeerd ontslaat of je let er niet op, ik zeg weleens: dan krijg je de patiënt per kerende post via de andere poot terug, als het niet goed geregeld is. In zoverre word je wel terug… Het zou misschien nog wel wat sterker mogen zijn, maar je krijgt ze wel terug omdat het niet goed geregeld is. Dat is denk ik alleen maar prima. Wat wel heel lastig daaraan is; als huisarts word ik er niet gelukkig van dat je eerst moet kijken: waar is deze patiënt bekend? In het … (naam ziekenhuis)? In het … (naam andere ziekenhuis), wat ook een eigen palliatief team heeft? Weer in een ander ziekenhuis? Is hij wel bekend in het ziekenhuis, maar niet bij het palliatieve team? Dat zijn allemaal andere telefoonnummers en daar zou ik als huisarts heel ongelukkig van worden.

**I: Ja. Ja. Ja, dat kan ik me voorstellen, ja.**

R: Dat speelt dus al vanaf… Nee, aan het begin was dat niet zo omdat je geen echte teams had, maar sinds die tijd is dat al een heikel punt wat we nog niet opgelost hebben gekregen. Dat vind ik een hele lastige. Ja.

**I: Ja, oké. Oké. En zijn er nog meer partijen waar jullie mee samenwerken? We hebben de huisartsen bijvoorbeeld besproken en de andere mensen in het ziekenhuis, maar bijvoorbeeld ook met maatschappelijk werk?**

R: Ik zeg al, gewoon de andere specialisaties, de huisarts, de verpleeghuisarts of dat soort dingen, dat maakt allemaal niet uit, die vallen onder dezelfde categorie als die huisartsen en die bellen ook met IKNL. Met maatschappelijk werk doen wij, vind ik, heel weinig.

**12.21**

**I: Oké. Of pastorale hulp, zoiets.**

R: Pastorale hulp is een hele sterke poot in ons team.

**I: Oké. Oké. Ik noem maar wat voorbeelden hè?**

R: Ja. Nee, maar daarom zeg ik: met maatschappelijk werk doen wij eigenlijk heel weinig. Dat is meer incidenteel. Ik wil bijna zeggen, als er financiële problemen zijn of iemand die doodgaat, die nog een eigen bedrijfje heeft en je denkt van: oei, dat is echt iemand anders zijn expertise. Dat is dus een andere insteek. Binnen het ziekenhuis hebben we een hele grote link naar de geestelijke zorg. Helaas is onze vaste geestelijk verzorger op dit moment overspannen. Die missen we wel. Die zat van tevoren drie, vier ochtenden bij onze overdracht, dus die had ook heel veel linkjes. Je merkt dat dat nu toch een beetje verwaterd. De psycholoog zit wel bij ons MDO op dinsdag, dus als alle palliatieve patiënten besproken worden. Daar zit ze structureel bij. Daar wordt ook af en toe gezegd van: nou, verwijs maar naar mij. Maar daar merk je dat… Ja, klinisch is volgens mij… Ik heb niet de indruk dat de psychologen veel bij de klinische patiënten langsgaan. Poliklinisch… Zeker als je in de eerste lijn iets wilt doen zijn de wachttijden lang en worden ze alleen maar vergoed als er sprake is van een psychiatrische stoornis. Ja, deze patiënten gaan dood en hebben daar moeite mee. Dat is geen psychiatrische stoornis. Daar lopen wij nog heel hard tegenaan. Een aantal jaar terug had je … (aanbieder van psycho-oncologische zorg), die volgens mij in … nu verdwenen is. Het … (aanbieder van psycho-oncologische zorg), dat is nou het … geworden. Die zat op de campus bij … (plaats op campus van ziekenhuis). Dat is op een gegeven moment samengegaan ook met …. Ik denk dat die in … nog steeds zit. Die hebben dus echt een psychiatrisch labeltje nodig. Dat is een lastige voor een aantal patiënten.

**I: Ja. Ja. Dat kan ik me voorstellen, ja. [Onderbreking door iemand die binnen komt en die vraagt even van plek te verhuizen, wat vervolgens gebeurt]**

R: Even kijken. Over de psycholoog. Nou ja, dat is met name ook het financiële plaatje wat daarin lastig is en de lange wachtlijst. Als je een gemiddelde patiënt hebt, die wij zien, dan kan je zeggen: mwah, drie maanden, zes maanden, dan zijn ze toch wel overleden. Die laat je niet twee maanden wachten op behandeling, dat werkt niet. Dus dan is het altijd een beetje zoeken van: wat kan en wat kan bijvoorbeeld bij de POH-GGZ bij een huisartsenpraktijk? Dan is het altijd de vraag: is die stevig genoeg om echt deze begeleiding te kunnen bieden?

**I: Ja, dat is ook een relatief nieuwe functie natuurlijk.**

R: Ja. Daar zitten natuurlijk een paar hele goede tussen en ook een paar waarvan je denkt…

**I: Ja, de achtergronden zijn heel verschillend hè, geloof ik. Ja.**

R: Ja. Ik zit even te kijken of er nog andere… Dan zouden het de linkjes naar de wijkverpleegkundigen… Met de gewone wijkverpleegkundige is dat heel matig. Een aantal mensen van specialistische teams, die ken je op een gegeven moment gewoon omdat je langer in het vak zit. Ja. Ik denk dat ik daar van alle teams wel iemand ken, maar die groepen zijn gewoon te groot. Die krijg je niet meer goed in zicht.

**I: Ja, precies. Oké. Als je het goed vindt wil ik graag wat inzoomen op gesprekken, over communiceren, informeren en gezamenlijke besluitvorming. Je noemde net; jouw rol is eigenlijk het hele aspect, van intake tot het laatste stukje. Je hebt daar eigenlijk meerdere aspecten in: het fysieke, psychische, sociale en spirituele welzijn van de patiënt. Ik vroeg me af: in hoeverre komen deze verschillende aspecten aan bod in gesprekken met de patiënt?**

R: In principe bij een eerste intake komen ze denk ik standaard allemaal wel aan bod. Fysiek is natuurlijk vaak de ingangsvraag waarom patiënten komen. Ik denk dat het kijken naar hoe het sociale plaatje eruitziet – woont de patiënt alleen, heeft hij een mantelzorger, hoe zit hij in zijn vel? – ook wel een standaardvraag is. Ik werk meer via de SCEGS. Meer van: wat houd je op de been, wat is voor jou belangrijk? Dat type vragen. Een vraag over euthanasie of palliatieve sedatie, hoe zien mensen het einde van het leven; in die hoek kom je eigenlijk altijd wel uit. En bij de klinische patiënten ook van: wil je hier blijven, ga je naar huis, kan dat thuis, moet je dat ergens anders doen?

**19.25**

**I: En breng je die altijd zelf ter sprake, dat soort aspecten?**

R: Ja, ik denk het… Ja. Dit zijn de vijf punten en een beetje afhankelijk van wat de patiënt aangeeft komt het ene meer of minder nadrukkelijk naar voren. Ik denk dat als je iemand hebt die helemaal op het eind zit, je veel eerder zult zeggen: goh, wat zijn er nog meer voor zorgmogelijkheden? Aan het begin denk je van: ja, deze meneer is ADL-zelfstandig, niks aan de hand. Dan heb je vaak iets heel anders waar je je focus op legt. Dus het wisselt wel wat ter sprake komt.

**I: Oké. Er zitten wel echt duidelijke verschillen tussen patiënten.**

R: Ja. Ja. Ja, ik laat de patiënt meestal zelf bepalen wat voor hem het meest essentiële is op dat moment. Iemand die vergaat van de pijn denkt altijd: ik wil van de pijn af en de rest zal me een worst wezen.

**I: Ja, precies. Dan ben je op de korte termijn…**

R: Ja, soms moet je eerst de korte termijnproblemen oplossen voordat je met andere dingen aan de gang kunt.

**I: Ja, oké. En bij patiënten die bijvoorbeeld laagopgeleid zijn, die niet zo heel veel lijken te snappen, werkt dat dan ook zo?**

R: Nou, ik denk dat je daar wel in principe ook dezelfde items aan bod laat komen. Ik merk dat meer altijd bij shared decision making. Ik denk dat we bij iemand die tijd van leven heeft en dingen snapt, we veel meer proberen om dingen samen te beslissen. Ik denk dat je tegen iemand die het allemaal niet zo goed snapt veel meer zegt van: toek, toek, toek, dit gaan we doen. Zo van: jij hebt pijn, daar krijg je dat voor, punt. Dat je dat niet helemaal probeert uit te… Van: waarom kies je voor het een of voor het andere? Op een gegeven moment is het gewoon: dit is voor jou de goede optie. Dan ga je toch meer de paternalistische kant op. Maar ik geloof minder in het… Ik denk dat het shared decision making heel mooi klinkt, maar dat een hele grote groep van de mensen dat niet helemaal kan en misschien moet ik ook zeggen: misschien niet eens helemaal wil. Ik heb een patiënt gehad bij wie ik wel durf te zeggen dat die man veel en veel slimmer is dan ik. Maar die had zoiets van: nou, jongens, als ik kijk naar wat er medisch past bij mijn ziektebeeld, dan kan ik helemaal niks meer en dan kan ik het ook niet plaatsen in het goede belang. Ik krijg alleen maar …, dus ik doe er niks mee. Hij kan er niks mee. Hij zegt: als ik de bijsluiter lees; ik snap dat er ook wel iets in moet staan van wat één keer in de honderdduizend voorkomt, maar ik kan niet plaatsen wat voor mij nou relevant is hierin. Dat is echt een meneer die meerdere universitaire studies gedaan heeft in zijn verleden, dus het is echt een intelligente vent.

**I: Ja, ja. Ja, bijzonder wel, eigenlijk, toch?**

R: Daarom denk ik van: ja, als jij ergens verstand van hebt, dan kun je zeggen: oké, dit neem ik mee, dit neem ik niet mee en dit is voor mij essentieel. Maar ik geloof er ook in dat het een beetje concreet moet zijn voordat mensen iets kunnen meebeslissen.

**23.07**

**I: Ja. Ja, oké. Mag ik dadelijk nog wat doorvragen over shared decision making? Ik vroeg me nog af, in die communicatie met patiënten, die aspecten die worden besproken, worden die ook vastgelegd in een zorgplan?**

R: Ja, dat is altijd een leuke, want wat noem je een zorgplan? Laten we zo zeggen, wat wij bespreken zetten we in principe wel in onze notities in EPIC en dat is voor iedereen te lezen.

**I: In?**

R: Gewoon in EPIC, in het elektronisch dossier. In theorie heeft de patiënt daar zelfs toegang toe, afhankelijk van of je dat aangevraagd hebt of niet. We maken er ook geen geheim van. Echt een zorgplan opstellen met de patiënt, volgens mij doen wij dat niet zo. Volgens mij doen wij daar niet veel mee. Als iemand pijn heeft zeg je inderdaad: dit en dit en dit gaan we proberen, we bellen u over drie dagen op om te kijken of deze wijziging in medicatie effectief is geweest. Dat staat bij ons meer onder beleid, in drie regeltjes, dan dat we daar een heel zorgplan van maken.

**I: Oké. Ja, duidelijk. Zijn er nog patiënten bij wie jij de communicatie moeilijker vindt? Ik vroeg net van: bij laagopgeleiden of mensen die niet zoveel begrijpen, ga je – als ik jou goed heb gehoord hoor, anders moet je me natuurlijk verbeteren – een aantal dingen wat concreter maken. Maar zijn er patiënten bij wie je dat ook moeilijker vindt, de communicatie?**

R: Ja. Er zijn absoluut patiënten bij wie het gesprek soepeler loopt dan bij anderen. Ik weet alleen niet of dat ligt aan het hoog- of laaggeletterd zijn.

**I: Oké. En in algemene zin dan, dus even los van het geletterd zijn?**

R: Soms heb je van die gesprekken dat je denkt van: dit loopt vanzelf. En sommige gesprekken lopen voor geen meter. Ik vind het soms ook lastig om achteraf te bedenken van: waarom ging dit gesprek wel of niet?

**I: Ja, want in welk opzicht…**

R: Ik vind het altijd heel makkelijk of heel prettig als de patiënt gewoon vertelt en vragen stelt, waarop je iets kunt antwoorden. Degene die zo achteruitgaat zitten van: nou, kom maar… Laatst had ik iemand die zei op de vraag of ze kinderen had: ja. “Hoeveel kinderen?” “Twee.” Maar dan ook geen enkel… De meeste mensen beginnen dan te vertellen van: ik heb kinderen, ze wonen daar. Die beginnen dan meteen een heel verhaal. Maar deze meneer; het antwoord gaf hij en dan hield hij structureel zijn mond. Dat is gewoon lastig.

**I: En wat dan? Zijn er dan dingen die je doet, dat je je communicatie gaat aanpassen?**

R: Je hebt natuurlijk van tevoren wel een aantal dingen waarvan je zegt: goh, die wil ik graag weten. Maar het wordt een beetje een vraag-antwoordspelletje en dan heb ik zelf nooit het gevoel van: dit gesprek loopt. Dat vind ik dus zelf de lastigste patiënten. Ja. Ik denk dat dat ook in mijn gesprekstechniek zit, misschien is het een tekort in mijn gesprekstechniek.

**I: Oké. Nou ja. Dat weet ik niet…**

R: Maar dat vind ik een lastige. Ja, met name de hoofd hals patiënten; daarvan heeft toch een hoog percentage alcoholmisbruik, veel roken, weinig sociale context. Dat zijn eigenlijk degenen die, kort door de bocht, vaak wel in een categorie gestopt worden van: wat moet ik hier nou mee? Ik vind dat zelf niet altijd de lastigste patiëntencategorie. Die zijn vaak heel direct, je weet helemaal wat je aan hen hebt. Recht door zee. Ja. En soms weet je ook dat je er niets mee kunt. Dat kan ook.

**28.05**

**I: Maar hoezo kan je daar niks mee dan?**

R: Daar vertel je vier keer aan… Nou ja, er is één patiënt die ik echt helemaal nog op mijn netvlies heb zitten. Dan vertel je wel wat hij moet doen en vervolgens bel je hem en dan blijkt hij gewoon in plaats van met de kortwerkende met de langwerkende medicatie aan het rommelen te zijn. Of hij zegt “ja” en vervolgens bel je hem twee dagen later en heeft hij “nee” gedaan. Ja, dat is heel lastig. Ik zeg altijd: het niet stuurbaar zijn op een gegeven moment. Aan de andere kant was die man daar ook heel eerlijk in.

**I: Ja. Dat is dan wel fijn. Maar wat doe je dan? Is herhalen dan de truc of heb je hulpmiddelen dan om dat toch anders te…**

R: Nee, daar kwam ik ook niet goed uit. Je gaat toch proberen het te herhalen. We hebben volgens mij heel hard geprobeerd om er een huisarts in te krijgen, die dichtbij was, die kon zien wat er gebeuren in plaats van telefonische... Volgens mij hebben we bij die man heel lang geprobeerd om er ook thuiszorg in te krijgen van, kunnen we daar op de een of andere manier structuur in krijgen. We hebben die man laten veranderen van huisarts, omdat zijn huisarts te ver weg woonde. We hebben wel van alles en nog wat geprobeerd om daar structuur in te brengen, met zeer wisselend... Met matig resultaat, laat ik het zo zeggen. Die man had op zich wel vertrouwen hier in het ziekenhuis, maar ja, je wilt op een gegeven moment eigenlijk op een andere manier... Daar zijn we niet helemaal uit gekomen. Ik weet, toen dit onderzoek kwam, zei iedereen spontaan: dat was de ideale… Volgens mij was hij twee weken daarvoor of zo overleden. Iedereen had zoiets van: dit is het stereotiepe beeld voor jullie onderzoek. Als je hem dat gevraagd was, had hij waarschijnlijk gezegd: oh, dat is goed.

**I: Ja, ja. Hij had zo meegedaan.**

R: Ja. Als de goede het hem gevraagd had hè? Er waren twee of drie mensen hier die mochten wel… En de rest hoefde ook niet langs te komen. Een bijzondere man.

**I: Ja, precies. Ik wilde nog even doorvragen over gezamenlijke besluitvorming. Mijn vraag is eigenlijk, je hebt er al wat over gezegd, in hoeverre er volgens jou sprake is van een gezamenlijk besluit bij patiënten in de palliatieve fase.**

R: Nou, je hebt zeer zeker wel een stuk gezamenlijke besluitvorming. Ik denk dat patiënten heel goed bijvoorbeeld mee kunnen denken over: wil ik wel of niet behandeld worden, wil ik wel of niet ingestuurd worden? Dat zijn ook echt vragen die aan de patiënt gesteld worden, waarbij je ook probeert om samen te kijken van: wat zijn de voordelen, wat zijn de nadelen? Nou ja, zoals we nu al vaak doen, van: voor u is het belangrijk dat u thuis wilt overlijden, als u 112 belt wordt u ingestuurd, dus als er iets gebeurt, bel de huisarts. Maar ik denk, het moment dat ze bij ons komen is het moment dat je daarover nog netjes kunt overleggen. Eerlijk gezegd denk ik ook dat de manier waarop je je informatie brengt dusdanig kleuring geeft aan die informatie dat je daarmee ook de keuze bepaalt.

**32.07**

**I: Oh, zo. En hoe zou jij dat dan zelf…**

R: Ik weet niet of dat erg is, dat is een tweede. Maar als je gewoon kijkt naar de patiënten die wij zien; wij weten van tevoren: die oncoloog, dan denk ik dat die niet wordt doorbehandeld tot in het graf. Die oncoloog, dan worden we iets eerder ingeschakeld; die oncoloog… Dan ken je de patiënt nog niet, maar dat zou heel bijzonder zijn, als de ene oncoloog alleen maar die patiënten heeft die heel graag tot het eind doorbehandeld willen worden. Soms kun je zeggen van: goh, de kans op slagen is zestig procent. Een ander zegt: ja, de kans op effect is zestig procent, maar dat levert u dan misschien twee maanden winst op. Dat is natuurlijk maar net hoe je het brengt met dezelfde boodschap. Dus ik denk dat de manier waarop je het brengt veel meer zegt over wat die patiënt gaat doen. Ja, moet je dat onder shared decision making… Ik denk dat je daarin een ontzettend belangrijke rol hebt. En als je zelf inschat, dat moet je niet doen, dan breng je het wel wat negatiever dan wanneer je denkt, dit moet je absoluut wel doen. Natuurlijk zijn er mensen die ondanks dat jij zegt, dat moet je wel doen, zeggen, maar ik doe het niet. Die heb je er een paar tussen. Ik heb er vandaag één gehad, die zegt: en toch doe ik niks. Dat mag ze van mij.

**I: Maar met niks bedoel je?**

R: Die zegt van: ik wil niet behandeld worden, ik wacht gewoon rustig af hoe dat gaat lopen. Zij staat er helemaal achter, terwijl de chirurg toch oorspronkelijk heeft gezegd van: ik zou iets doen. Zij zegt: en ik niet. Het is voor mij natuurlijk altijd heel makkelijk als ik achteraan sta en zij heeft die keuze al gemaakt, om dan met haar verder te gaan. Dus ik heb daar ook geen… Maar ik denk dan: er zijn bijna geen patiënten die dan zeggen van, ik doe het niet meer verder, als het aangeboden wordt.

**I: Op die manier. En als je het dan omdraait, dus als je patiënten hebt die eigenlijk nog heel veel willen doen, maar waarvan jullie eigenlijk al weten van: het zou eigenlijk het beste zijn als er niets meer zou gebeuren.**

R: Dat vind ik niet moeilijk. Wacht even, ik zit even te kijken. Je hebt dus de patiënt, bij wie wij zeggen: dit is zinloos medisch handelen.

**I: Eigenlijk wel. Dat bedoel ik, ja.**

R: Misschien moet ik zeggen, ik zou willen dat dat vaker gezegd wordt. Wij krijgen weinig patiënten te zien van wie ik denk: oh, die stoppen eerder dan dat ze… Er wordt vaker te lang doorgegaan dan dat ik het gevoel heb van: nou, hier had nog wel iets meer gemogen. Maar wellicht komt dit ook doordat wij… Welke patiënten zien wij? Dat zijn de patiënten met wie het niet goed gaat. En als het goed gaat, dan verwijs je in principe niet door. De reanimaties die mislukt zijn, zie ik niet. Een reanimatie die wel lukt, zie ik ook niet. Ik zie alleen de reanimaties waar iemand wel uit is gekomen, maar waarbij die patiënt vervolgens als een kasplantje in leven blijft. Dan krijg ik ze te zien. Ja, dan krijg ik wel een gekleurd beeld. Dus ik denk dat wij daardoor ook gekleurd zijn. En ik denk dat een ziekenhuis, zeker een academisch ziekenhuis, ook een bepaalde patiëntenpopulatie binnenhaalt. Ik werkte op de Medische Oncologie. Daar heb ik vijftien jaar gezeten voordat ik hier begon. Een tachtigjarige die het prima vindt om niet meer behandeld te worden, die blijft in zijn eigen lokale ziekenhuisje. Die dertigjarige die zegt van: ja, jongens, ik wil nog eigenlijk koste wat het kost mijn kinderen groot zien worden, die wordt doorgestuurd en daar wordt het onderste uit de kan gehaald. Dus je krijgt ook een bepaalde groep mensen die hier nog een fase-III-, een fase-II- of een fase-I-studie gaat doen. Dus daarmee heb je ook een andere categorie patiënten die hier binnenkomt dan die in het … (naam ziekenhuis) binnenkomt, waar de lokale bevolking veel meer naartoe gaat. Dat levert denk ik een ander soort dokter op, maar ook een ander soort patiënt. En als die alle twee gaan voor die fase I, waar je je alle twee blijkbaar lekker bij voelt…

**I: Ja. Dat is een interessante invalshoek.**

R: Ik denk dat dat echt verschil maakt.

**37.23**

**I: Ja. Ja. Ik had nog een aantal vragen. Ik wil even letten op de tijd. Ik geloof dat we drie kwartier hebben. Ik had ook nog een aantal vragen over beperkte gezondheidsvaardigheden, waar we het ook al over gehad hebben. Wat versta jij daaronder?**

R: Nou, daar hebben we het natuurlijk hier onderling wel een paar keer over gehad in het kader van jullie inclusiecriteria. Het eerste wat toch bij je opkomt, ik zou bijna zeggen, zijn die hoofd-halstumorpatiënten, die minder intelligent zijn, die het allemaal niet goed snappen. Dat is het eerste dat bij je opkomt. Aan de andere kant denk ik dat verminderde gezondheidsvaardigheden ook om het coping gedrag van mensen kan gaan, van: ga jij op een effectieve manier met je gezondheid of met jouw ziekte om, wat jou ook kwaliteit van leven oplevert? Misschien zou ik dat nog veel essentiëler vinden dan of ze snappen dat ze een pilletje meer of minder moeten nemen. Maar die zijn wel heel lastig te vangen in zo’n…

**I: In een studie.**

R: Precies.

**I: Ja, zeker. Ja, want hoe…**

R: Ja, ik denk dat ik vandaag iemand heb gehad, die voelt zich zo onzeker binnen haar ziekte, terwijl ik denk dat zij heel adequaat de dingen doet, heel adequate keuzes maakt, heel bewuste keuzes gemaakt. Maar als er dit gebeurt, gaat ze onderuit en denkt ze: is dit nu het begin van het einde, help! Nou ja, wat ze gelukkig heel goed doet, is dat ze op dat moment gewoon gaat bellen, gaat vragen. Het is een hele pittige tante, dus die belt naar de huisarts en die belt naar ons, die zoekt haar weg er wel in. Aan de andere kant denk ik: het is eigenlijk wel bijzonder hoe snel zij onderuitgaat. Dus of het helemaal adequaat is…

**I: Ja. Maar deze patiënt in dit voorbeeld, zie jij die dan als patiënt met lage gezondheidsvaardigheden?**

R: Nee. Ik vind het denk ik niet altijd adequaat. Lagere gezondheidsvaardigheden vind ik…

**I: Ik vind het een lastige term, eerlijk gezegd. Heel beperkt is het eigenlijk.**

R: Ja. Ja. Ja. Ik heb ook niet bij haar zoiets van: goh, die zou ik voor dit onderzoek moeten vragen. En is het dan de patiënt zelf of is het vaak de context eromheen die maakt dat het zoeken is? Ik merk dat ik het ook altijd… Ik denk niet dat het toeval is dat de inclusie niet zo heel soepeltjes verloopt, omdat wij zelf al zitten te zoeken van: hoe vaak zie je die patiënt, wanneer laat je hem terugkomen? Je kunt het niet zomaar de eerste de beste keer… En soms is het: hier klopt iets niet, maar wat is dit nou?

**41.10**

**I: Bedoel je dan hoe je zo’n patiënt herkent bijvoorbeeld?**

R: Ja. Ik vind het een lastig begrip.

**I: Ja. Ja. Ja. Ja, want mijn volgende vraag zou zijn: hoe herken je patiënten met beperkte gezondheidsvaardigheden? Dat zit dus in dezelfde lijn eigenlijk.**

R: Ja. Ja. Dat vind ik echt een hele lastige: wanneer noem je iets een beperkte gezondheidsvaardigheid? Eigenlijk hebben bijna alle palliatieve patiënten moeite met de situatie, maar wanneer is het een beperkte gezondheidsvaardigheid?

**I: Ja. Er is wel een definitie, dat mensen het moeilijk vinden of dat vaardigheden eigenlijk ontbreken om informatie over gezondheid te krijgen, te begrijpen en toe te passen op zichzelf. Dan gaat het ook over besluiten over gezondheid. Dat zit er dan ook in. Dat is eigenlijk een beetje de definitie zoals we hem nu hebben. Maar ik ben het met je eens, ik vind het ook een lastige term.**

R: Ja, ik vind hem ook lastig. Het eerste wat toch bij je opkomt is een verminderde cognitie, terwijl ik denk van…

**I: Ja, maar dat is niet alles, toch? Dat is eigenlijk wat jij zegt.**

R: Dat is absoluut niet alles. Ja, ik zeg al, mensen kunnen misschien niet slim zijn, maar wel een modus hebben waarmee ze uitstekend uit de voeten kunnen. Ja. Is het daarmee een verminderde gezondheidsvaardigheid? Ik weet het niet.

**43.25**

**I: Ja. Ja. Ja, die is lastig inderdaad. Ik had ten slotte nog wat kaartjes. Wilsonbekwaamheid en levenseindebeslissingen. Dit is de langste: wettelijke vertegenwoordiging in de situatie van acute verslechtering. Niet-behandelafspraken. Crisissituaties. Levensbeschouwing en culturele achtergrond. Ziekenhuisopnames. Plaats van zorg en sterven. Nou is mijn vraag; in hoeverre deze aspecten aan bod komen in gesprekken met patiënten en of je daar misschien zelfs een rangorde aan kunt aanbrengen.**

R: Ik denk dat ziekenhuisopnames heel veel… Dat is echt iets dat vaak aan de orde komt.

**I: Oké. Ik had er ook al een aantal gehoord inderdaad.**

R: Wilsonbekwaamheid en levensbeëindiging; nou, wilsonbekwaamheid wil ik niet zeggen, maar levenseindebeslissingen heel duidelijk ook. Crisissituaties, dat komt echt ergens op het einde. Dit zijn bijvoorbeeld de COPD-patiënten/de longpatiënten van wie je zegt: goh, als u nou weer opnieuw benauwd wordt, wilt u dan nog ingestuurd worden?

**I: Ja, precies. Of ze dan 112 moeten bellen of de huisarts...**

R: Ja. Dan zie je die nog weleens naar boven komen. Levensbeschouwing en culturele achtergrond; ik vind dat dat relatief weinig aan bod komt. Misschien moet ik zeggen: het komt vooral aan bod op het moment dat je met allochtone patiënten te maken hebt en die zijn er relatief weinig. Misschien ligt daar ook het verschil tussen de patiënt die aan de bel trekt en zegt, ik wil alles uit de kast halen, die goed geletterd is en informed consent kan tekenen en noem maar op… Ik denk dat ook een deel is waardoor je op de Oncologie heel weinig allochtonen ziet. Ik denk dat daar ook nog een verschil in zit. Maar ik vind dat dit bij ons een ondergeschoven… Het is er wel, maar het is ondergeschoven. Plaats van zorg en sterven; ik zou bijna zeggen: dat is standaard. Ja, ik denk dat dat bij alle patiënten wel een keer… Misschien niet in ieder gesprek, maar het is eigenlijk altijd wel van: wilt u naar huis of gaat u naar een hospice? Dat zit echt in het standaardstramien. Niet-behandelafspraken, die zit er ook wel een beetje in, ik denk een beetje hier tussenin. Wilsonbekwaamheid. Dit ligt toch een beetje… Levenseindebeslissingen, behandelafspraken. Ik denk dat dit net iets vaker voorkomt dan behandelafspraken. Misschien moet ik zeggen: deze met zijn drieën bij elkaar. Het ligt toch vaak een beetje in elkaars verlengde. En de behandelafspraken is dan meer… Of het niet-behandelen. Ja, het is dan vaak van: goh, als u straks benauwd wordt en u krijgt een pneumonie, wilt u dan ingestuurd worden of wilt u dat thuis al dan niet behandeld… Dus iets minder nadrukkelijk dan de andere twee. Wettelijke vertegenwoordiger, dat is ook iets wat weinig… Je zou bijna zeggen: achteraan. Daar wordt niet heel vaak naar gekeken. Af en toe. Soms komt hij heel nadrukkelijk naar voren. Twee weken terug had ik een patiënt die bij de Neurologie gevonden was door een ex-partner, waarbij iedereen dacht van: wat is hier gebeurd? Waarbij toen echt gezocht is van: wie is hier de wettelijke vertegenwoordiger? Dus af en toe, als het echt juridisch gezien… Daar was het een hot topic, maar dat is iets wat niet structureel of standaard naar voren komt. En normaal gesproken heb je natuurlijk een gesprek en zeg je: wie is de zoon, wie is de partner? Zonder erover na te denken weet je het, maar als dat niet zo helder ligt… Het is niet een automatisme om erover te beginnen. Ik denk dit als minste, dan die en deze altijd.

**I: Oké, ja. Dus even voor de audio-opname: levensbeschouwing, culturele achtergrond en wettelijke vertegenwoordiging eigenlijk in de minste mate. Dat is de achterste. Wettelijke vertegenwoordiging, daarvan zeg je: die vind ik heel logisch. Maar levensbeschouwing en culturele achtergrond vind je eigenlijk een beetje een ondergeschoven…**

R: Ja, ik denk dat we daar wellicht nog iets zelf… Ja, ik denk dat ik daar meer mee zou kunnen of moeten doen dan ik nu doe. Ja.

**I: Ja, precies. Oké. En in het midden heb je dan wilsonbekwaamheid, levenseindebeslissingen, niet-behandelafspraken, crisissituaties en ziekenhuisopnames. Die zijn een beetje geclusterd met zijn vieren. En vooraan ligt de plaats van zorg en sterven.**

R: Ja. Ik denk dat dit, ik zou bijna zeggen, voor honderd procent besproken wordt. Dit bij tachtig en dan dit laatste stukje bij misschien twintig. Ja.

**I: Oké. Ja, duidelijk. Had jij nog vragen of toevoegingen hierop?**

R: Nee. Nee. Ik vind het altijd wel fijn als je concrete vragen hebt.

**I: Ja. Waren ze concreet? Redelijk toch?**

R: In ieder geval voor mij concreet genoeg. Daar kan ik mee uit de voeten.

**[53.42 – einde opname]**

Z4d03

I: interviewer

R: respondent

**00.27**

**I: Ja. Dank u wel. Nu is eigenlijk voor mij al meteen de eerste vraag; ik wil het graag gaan hebben over uw rol als zorgverlener in de palliatieve zorg, over communicatie, over patiënten en over de manier waarop het ziekenhuis georganiseerd is. Dat zijn eigenlijk de hoofdthema’s. Dus ik kan alle dingen de revue laten passeren, maar ik vraag me af of het in uw geval misschien wat interessanter is om vooral te praten over de manier waarop het ziekenhuis georganiseerd is, aangezien u toch overal bovenop zit, als ik het zo mag zeggen.**

R: Je bent aan het opnemen hè?

**I: Ja, ja, ja, absoluut.**

R: Is dat de opneemapparatuur, zo klein?

**I: Ja, ja.**

R: Ik wist niet dat het bestond, zo klein.

**I: Ja, dit is ook meteen een USB-stick, 4 Gb.**

R: En daar kun jij nu mee opnemen?

**I: Ja. Ja, hij staat aan.**

R: Heel goed. Nee, misschien even gewoon over mijn rol. Kijk, de organisatie van het ziekenhuis… Je hebt een Raad van Bestuur. Ik neem aan dat je dat wel begrijpt. Een vierkoppige Raad van Bestuur. Je hebt ook een aandachtsveld. Onze afdeling valt rechtstreeks onder de voorzitter van de Raad van Bestuur, L. Dat komt doordat de afdeling Anesthesie bij de palliatieve geneeskunde… Anesthesie is natuurlijk een hele grote afdeling: operatiekamers, anesthesie. Ik denk dat bijna veertig tot vijfenveertig procent van alle patiënten uit het huis door onze afdeling stromen, voor een operatie, voor de ic, voor cardiochirurgie enzovoort. Onder die Raad van Bestuur hangen eigenlijk in technische zin vooral afdelingshoofden. Dat is altijd een hoogleraar, een afdelingshoofd, die ondersteund wordt, maar in een tweekoppige bemanning met een bedrijfsleider, de manager, de man van de financiën, de afspraken en zo. Die twee samen sturen een afdeling aan. Dan krijg je onder de hoogleraar, het afdelingshoofd, eigenlijk de opsplitsing in wat wij dan voor onze afdeling WPM’s noemen: werkplek managing places, noemen we dat dan even. WPM’s. Het WPM P. (benaming palliatieve afdeling ziekenhuis, kan i.v.m. anonimiseren niet geheel uitgeschreven weergegeven worden) is er daar één van. We hebben ook een WPM Cardiochirurgie, we hebben een WPM Kinderanesthesie, we hebben een WPM – ik geef maar een voorbeeldje – Helikopter, even heel technisch. Dus we hebben een ploeg van anesthesiologen die met de helikopter meevliegt, vanuit Volkel. Het is alleen maar om een gevoel te geven hoor. Dus het WPM P., daar ben ik het hoofd van. Dus iedereen die met pijn en palliatie werkt; dat is in verschillende ziekten. Wij werken in … (naam ziekenhuis, het … (naam ziekenhuis), … (naam ziekenhuis) en binnenkort in de … (naam ziekenhuis). Al die mensen vallen onder mij en ik geef ook verantwoording aan mijn afdelingshoofd. Dat is in mijn geval G., maar dat is misschien niet van belang, denk ik, voor het interview. Dus dat is de rechtstreekse lijn. Daar zitten nog spannende oefeningen in. Ik geef een voorbeeldje: paramedici en verpleegkundigen vallen onder een apart hoofd, die met mij afstemt. We hebben alle verpleegkundigen van onze afdeling onder één verpleegkundig hoofd gebracht, om daar een gevoel bij te krijgen. Dus H. en H. vallen onder dat algemene hoofd en dat hoofd is dan mij dan weer een tweekoppig bemanning. Zo krijg je altijd… Kijk, ik stuur dokters aan. Dat is in dit geval M., die stuurt H., H. en de andere verpleegkundigen aan. Die stuurt ook de psychologen aan. Maar ik ben inhoudelijk verantwoordelijk. De lijn loopt altijd op naar de wet WGBO, zal ik maar zeggen. Dus dat betekent ook dat de medisch-inhoudelijke taken altijd onder mij vallen. En dat is eigenlijk een beetje de kern. Kijk, technisch gezien heeft een ziekenhuis zoals dit vijftig afdelingen: de afdeling Radiologie, Interne Geneeskunde, Oncologie enzovoort, een heel lijstje. Dat zijn er ongeveer vijftig. Dus we hebben vijftig afdelingshoofden, vijftig bedrijfsleiders en we hebben vijftig structuren daaronder. Complex.

**I: Ja, absoluut. En u gaf aan dat er hier op deze plek, waar u zit, eigenlijk twee personen zijn. Dat bent u dus en dat is iemand die wat meer vanuit het organiseren werkt.**

R: Nee, het afdelingshoofd en de bedrijfsleider vormen samen een tweekoppige bemanning in de aansturing van onze afdeling. Dus het afdelingshoofd is de hoogleraar, maar de hoogleraar is minder met financiën bezig bijvoorbeeld. Die stuurt ook veel minder processen aan. Ik geef voorbeelden: de personeelsaannamedienst, secretariaten enzovoort. Dat valt allemaal onder *de* bedrijfsleider. We hebben ook wat we noemen een bedrijfsbureau. Dat is een groepje van mensen die verantwoordelijk zijn voor het genereren van data, voor het nakijken van protocollen, om te zien of al onze processen die ondersteunend zijn aan de roosters, vakantieplanning, veiligheidsprocedures, alles wat binnenkomt aan vragenlijsten van Inspectie, Ministerie, het Zorginstituut enzovoort; dus alle controlemechanismen lopen allemaal via ons bedrijfsbureau, dat aan die manager gerelateerd is. Die zorgt er eigenlijk voor dat de trein, de motor van de trein op gang blijft.

**05.50**

**I: Ja, precies. Oké. Dus dat is eigenlijk de structuur van bovenaf gezien. Uw rol voor patiënten in de palliatieve fase, dus iets meer toegespitst…**

R: Eindverantwoordelijke.

**I: Eindverantwoordelijke. Oké.**

R: Voor alle professionals die daarin werken. Ja. In mijn lijn. Dan kom je een beetje op een interessante oefening. Kijk, een oncoloog kan beslissen om palliatieve zorg te verlenen en dan valt hij niet onder mij, want dan valt hij onder de Medische Oncologie. Een cardioloog kan een palliatieve patiënt hebben, maar is daar zelf hoofdbehandelaar in. Dat is een ander verhaal. Snap je? Afdeling P. valt onder mij. Het medisch handelen palliatieve zorg valt niet… Ja, mijn dokters vallen onder mij, maar ieder afdelingshoofd is voor zijn artsen verantwoordelijk. Dat is wel een interessante oefening hè? Dan kom je echt aan wat de KNMG (Koninklijke Nederlandsche Maatschappij tot bevordering der Geneeskunst; de artsenfederatie) organiseert. De KNMG zegt ook: een hoofdbehandelaar is de dokter die verantwoordelijk is, de centrale behandelaar van de patiënt. Je hebt een huisarts en als je je been breekt, dan heb je een orthopeed. Dan heb je twee hoofdbehandelaars: eentje voor algemene problematiek, eentje voor de heup die je gebroken hebt.

**I: Ja. Oké. Op die manier. Die vind ik wel redelijk ingewikkeld. Ik kan me voorstellen dat dat ingewikkeld is.**

R: Ik zou zeggen: even Bruins opbellen dan, de minister. Die organiseert dat centraal. Ik kom uit een land waar dat niet bestaat op die manier. In mijn land is dat veel eenvoudiger georganiseerd. Maar in Nederland kun je dus als je een palliatieve patiënt bent vijf hoofdbehandelaars tegelijkertijd hebben. Het is als professional, dokter of verpleegkundige, een hele kunst en een deel van je opleiding om te begrijpen van: bij welke vraag moet ik bij wie zijn? Er is geen centraal beleid rond de patiënt hier hè? Nog veel erger, als jij bekend bent in het Rijnstate omdat je een huidallergie hebt en je bent bekend in het Radboud omdat je diabetes hebt, suikerziekte, en je bent bekend in het CWZ – ik geef het maar even lokaal op, ik weet niet waar je vandaan komt – en je hebt in het CWZ iets lopen omdat je ooit een bril aangepast hebt moeten krijgen en andere dingen, dan heb je drie ziekenhuizen die niet met elkaar communiceren en die sinds 23 mei ook geen vragen meer mogen stellen aan elkaar. Dus je gaat drie keer dezelfde vraag krijgen en drie keer hetzelfde antwoord moeten geven. Ik kan je zeggen, als na jou de volgende komt met dezelfde vraag, dan ben ik heel geïrriteerd. De patiënt heeft dat ook natuurlijk. Maar de enorme toename van de Wet op de Privacy maakt eigenlijk dat heel veel dingen gewoon onmogelijk worden. Dat is vooral het communiceren tussen zorgprofessionals. Ik mag niet eens meer een brief naar de huisdokter sturen zonder dat de patiënt daar eerst toestemming voor geeft, terwijl ik een advies geef aan de huisdokter officieel.

**I: Ja. Ja. Oké. Ja, die is heel ingewikkeld. Dus in … (land waar respondent vandaan komt), als ik het goed hoor, is het anders geregeld.**

R: We hebben maar één hoofdbehandelaar in … (land waar respondent vandaan komt), dus één centrale dokter. Je hebt één dokter, je bepaalt zelf wie dat is en die regelt je zaken. Dat lijkt me rustiger.

**I: Ja. Ja. Inderdaad. Ik zit even te kijken naar mijn vragen. Die zijn nogal gericht op hoe bijvoorbeeld u, of mensen onder u, communiceren met patiënten.**

R: Oké, maar dat is meer inhoudelijk dan. Nu heb ik als manager hier beantwoord hè? Dat was de vraag ook hè?

**I: Ja. Zeker, zeker. Dat is heel fijn. Maar nu is het dus bij mij een beetje de vraag of ik mijn antwoorden… Het heeft namelijk twee zijdes, het kan twee kanten op, wat meer op die managementkant en hoe de zorg georganiseerd is of wat meer gericht op bijvoorbeeld hoe u, of uw mensen onder u, communiceren met patiënten en hoe de communicatie gaat. Welke kant…**

R: Ik heb tijd tot tien uur, dus jij beslist wat je nodig hebt.

**09.42**

**I: Oké. Dan denk ik, voor mijzelf… Ik vind het sowieso interessant hoor. En bijvoorbeeld, iets gaat over beperkte gezondheidsvaardigheden, wat u daarvan vindt. Ik denk dat ik ga beginnen over het ziekenhuis en de organisatie, want daar weet ik nog heel weinig van. Als ik nog tijd over heb, ga ik u dan andere vragen stellen, als dat goed is. De eerste vraag is eigenlijk of er beleid is binnen uw ziekenhuis om zorg toegankelijker te maken voor mensen met beperkte gezondheidsvaardigheden.**

R: Herhaal de vraag nog eens? Ik probeer de taal even te…

**I: Is er beleid in uw ziekenhuis om zorg toegankelijker te maken voor patiënten met beperkte gezondheidsvaardigheden.**

R: Ja, goede vraag. Een heel goede vraag trouwens. De studie heeft hier ook deze discussie geopperd. Eén, ik denk, er is geen expliciet beleid, dus wij hebben geen Q-portaal-protocol, heet dat dan. We hebben één centraal pakket waar al onze richtlijnen staan. We hebben niet een Q-portaal-protocol om te zeggen: hoe detecteer je nu beperkte gezondheidsvaardigheden? Dat weten we niet. Twee, juist in onze algemene visie en missie hebben wij staan: persoonsgerichte zorg. Dat moet je maar eens op de website nakijken. In de persoonsgerichte zorg is juist de opdracht voor iedere professional impliciet om zich juist 100% op het niveau van de patiënt te zetten. Dat is best een oefening, even heel praktisch. Daar ben ik ook nieuwsgierig naar. Hoe detecteer je dat? Soms bemerk je dat achteraf. Maar wij hebben geen expliciet protocol waarin we weten, beseffen… Tenzij de patiënt geoormerkt is: een patiënt met een verstandelijke beperking, een patiënt met dementie, een patiënt met communicatiestoornissen, hulpmiddelen, die dingen. Dan is het expliciet. Maar in een gewone burger voor ons is dat niet een expliciete route. Er zit zelfs een knelpunt in. Voor je een afspraak krijgt bij ons op onze intakepoli krijg je een vragenlijst thuisgestuurd. Dat doen heel veel ziekenhuizen tegenwoordig. In die vragenlijst zit onder andere de vraag, wat is uw opleidingsniveau, om een beetje te weten te komen… Je mag dat niet expliciet aan elkaar koppelen, gezondheidsvaardigheden en opleidingsniveau, maar er zit wel een relatie tussen. Maar door de nieuwe patiëntenorganisatie wordt eigenlijk niet aanvaard dat we die vraag stellen, terwijl die wel essentieel is. Men vindt, de patiëntenorganisatie, dat vragen die gericht zijn op het exploreren van capaciteiten van een patiënt, buiten zijn medische model, over de grens gaat van het toelaatbare wat je mag vragen.

**I: Oké, dus bijvoorbeeld opleidingsniveau, dat vindt men niet goed dat dat gevraagd wordt?**

R: Dat vindt men niet goed, daar krijgen we heel veel commentaar op. Ook als we dat vragen tijdens een poli, dan zie ik dat er een hele grote weerstand van de patiënt is, dat die vraag gesteld wordt.

**I: Oké. Van: waarom willen jullie dat weten?**

R: Ja. Men heeft de angst dat de dokter een voortgeleide persoon is van zorgverzekeraars of van de VUT of van een heleboel andere organisaties, waardoor ze denken dat ze gecontroleerd worden. Dat is de grootste hobbel van dit dossier.

**I: Oké. Op die manier. Oké. U gaf al aan: impliciet, expliciet. Als ik het goed begrijp, als ik u mag samenvatten, dan is het eigenlijk op een gegeven moment aan de zorgverlener op dat moment zelf om daar mee om te gaan.**

R: Om daar iets mee te doen. Ja. Ja.

**13.08**

**I: Is er een bepaalde methodiek of zijn er bepaalde instrumenten die dat toegankelijker maken?**

R: Nou, kijk, wij geven veel vragenlijsten af, zoals ik al zei. Als we zien dat die niet gemakkelijk ingevuld worden of fout zijn ingevuld enzovoort, is dat voor mij in ieder geval een eerste indicator tot (beperkte vaardigheden). Ik heb geen vragenlijst om te testen: hoe zit die persoon in de wedstrijd rond zijn gezondheidsvaardigheden. Wij hebben natuurlijk wel altijd een soort van preventieve analyse, zeker in de palliatieve zorg, waarin we gaan kijken: is het een persoon die rookt, is het een persoon die gezond of ongezond leeft, heeft hij een normale BMI. Dus eigenlijk alle indirecte indicatoren…

**I: Lifestylefactoren.**

R: Lifestylefactoren. Maar lifestyle in de brede zin van het woord hè? Is het iemand die vooral ’s nachts leeft, iemand die vooral overdag leeft. Is het iemand die zegt: ik ken mijn grenzen of ik ken mijn grenzen niet. Dat zoeken we heel erg op. Maar dat blijf ik impliciet noemen. Waarom? Omdat ik niet een meetinstrument heb of een protocol om te zeggen: we gaan op een gestandaardiseerde manier hier mee om. Maar het zit wel in de vaardigheden, vind ik, van een goede professional. Dan kun je nog een debat voeren van: wat is nou een goede professional? Dat is een aparte vraag. Maar in de vaardigheden van een dokter, verpleegkundige, die ik in mijn team opneem, zit wel de vraag: heb jij persoonsgerichte aandacht? Dan komt dit punt heel erg naar boven. Dat zie je ook. De groep die voor ons heel lastig is; maar die is voor iedereen lastig in het huis. Kijk, gezondheidsvaardigheden bij de eigen culturele context, dat is minder moeilijk dan bij mensen met een allochtone oorsprong of uit bepaalde minderhedengroepen of mensen die nu… Zeker in deze vluchtelingentijd. Ja, nu wordt het echt heel moeilijk, want daar weten we het helemaal niets van. Dus je kunt die niet gaan benaderen, zoals je nu… Als een Nederlander uit Ede komt of uit Ewijk of wat dan ook, dan weet ik al precies hoe die mensen structureel denken, leven, waar ze wonen en wat ze doen. Maar dat vind ik nu, in de huidige context, waarin wij in ons ziekenhuis toch meer dan vijftien tot twintig procent mensen hebben van allochtone oorsprong… Dat is natuurlijk een vergaarbak hè, een containerbegrip. Maar dat is best spannend. En daar zijn wij niet aan toe, om te zeggen: ah ja, nu snap ik dat deze man het niet snapt. Ik heb heel vaak een vluchteling die ingenieur is in zijn land en die tegenover mij zit, maar die echt niet weet waarover het gaat en er ook niks mee kan, omdat wij elkaars cultuur en elkaars taal niet spreken. Dat vind ik wel een belangrijk punt. Nu zeker. Er is nu ook nog een discussie gaande: moet de allochtoon in zijn eigen taal bediend worden, met de tolkentelefoon? Ja, dat vind ik contextueel natuurlijk wel een enorm knelpunt en hobbel. Dit zeg ik nu in het kader van een onderzoeksplan, even heel technisch. Dat snap je ook wel. Als ik dat voor de microfoon zeg, sta ik vanavond op RTL. Maar kijk, als je praat over pijn en levenseindebeslissingen en hele moeilijke domeinen en je kent niet de gezondheidsvaardigheden van die persoon en die heeft er ook nog nooit over nagedacht enzovoort, dan is dat een onmogelijk gesprek. Dat is de kern. Dus ik straal ook uit, voor een aantal van onze behandelingen, dat het goed kennen van onze cultuur en onze taal enzovoort essentieel is om tot het goede resultaat te komen. Maar dat is een gevaarlijke uitspraak.

**16.28**

**I: Ja. Ja. Ja, precies. Aanvullend hieraan, wat zou belangrijk zijn voor jullie als ziekenhuis om die zorg voor mensen met beperkte vaardigheden toegankelijker te maken?**

R: Het eerste dat belangrijk is, is dat verpleegkundigen en artsen ruimte en tijd krijgen om een consult te voeren. Kijk, in de tienminutengeneeskunde die we nu hebben… Ook bij een huisdokter, een huisdokter krijgt zeven minuten om met een patiënt te praten. Doe me een lol. Dan gaan we absoluut niet een gesprek voeren over gezondheidsvaardigheden. Dan gaan we snelle adviezen geven en hopen dat ze landen. Dus als men nog meer afknijpt, dan zal dit nooit verbeteren. Dan moet je natuurlijk hele slimme instrumenten bedenken. Dat is dan waarom men dan kijkt naar eHealth, Tele-medicine en andere faciliteiten, zodat de patiënt de boodschap die de dokter gegeven thuis kan herlezen in zijn eigen tempo, maar dat het wel een plannetje is dat je samen hebt afgesproken op de poli. Dus als er iets moet verbeteren is het die hele entourage. Wat ik daar lastig aan vind; kijk, jonge mensen, van jouw leeftijd, vinden informatica en een iPhone onderdeel van hun leven. Voor mij is een iPhone iets wat ginder ligt en waar ze mij op bellen als ik dienst heb. Snap je het verschil? Ik leef daar niet mee.

**I: Er is al iets meer afstand.**

R: Ja, een hele grote afstand. Ik en mijn patiënt… We zijn beiden gehandicapt in het gebruik daarvan, niet één persoon. Snap je wat ik bedoel? Dat is wel iets waar je over moet nadenken. Ik kom uit het papieren tijdperk hè? Ik mag het zo niet zeggen, maar het is wel zo. Kijk, heel veel zorgverleners zijn boven de vijfenveertig en hebben grote drempels ten aanzien van die eHealth en Tele-medicine en we doen er zelf onderzoek naar. Ik ben daar niet een fan van. Dat is het eerste. Twee, je moet veel meer tijd krijgen. Ik denk dat juist de kern van wat we nodig hebben is dat als we merken dat een patiënt het niet goed begrijpt, het niet goed kan verwerken… Wat we heel gemakkelijk zien, laten we dat even vooropstellen. Als je mij gewoon vraagt, tik aan wat je indruk is van deze patiënt, even los van meetinstrumenten, dan is mijn voorspelling dat we dat vrij goed kunnen detecteren. We hebben alleen geen model om het te begeleiden en op te vangen. Dat is het hoofdprobleem, vind ik. Dan kun je nadenken over instrumenten, maar ik geef bewust even een antwoord dat wat breder is. Ik denk dat de patiënten met beperkte gezondheidsvaardigheden uit de boot vallen, omdat die boot gewoon aan een bepaald speedtempo werkt.

**18.59**

**I: Ja. Dus de boot gaat eigenlijk te snel voor die mensen om…**

R: Te snel voor deze gemiddelde burger. En daar moet je dus kunnen zeggen: ho mensen, we gaan in de achteruit staan en we geven er ruimte voor dat ze ingezwommen geraken.

**I: Ja, oké. En los van, u noemde tijd. De tijd van het consult moet langer, als ik het mag samenvatten.**

R: Ja.

**I: En eHealth, er is eigenlijk de realisatie dat ook de zorgverlener een afstand heeft ten aanzien van die tools. Dat is wat u eigenlijk aangaf hè?**

R: Ja, precies. En een codering hè? Een derde punt dat ik belangrijk vind is: als jij op je T-shirt een embleem draagt, waardoor ik weet dat jij hardhorend bent, wat je nu niet toont aan mij, even heel praktisch… U bent vaardig in het opvangen van wat ik zeg, denk ik, of je denkt: och, het is afgelopen, ik zal het straks wel afluisteren. Daarover gaat het debat wel. Het is natuurlijk heel lastig, want zo’n embleem zou heel snel stigmatiserend kunnen zijn. Dat moet je op een juiste manier kunnen gebruiken. Maar het herkennen van “iemand met” is natuurlijk een essentieel onderdeel. Hoe doe je dat? Hoe weet ik dat iemand gezondheidsvaardig is? Daar ben ik heel nieuwsgierig naar. Wat ik nog lastiger vind; ik zie dat mensen bij eenvoudige dingen best… Maar in het hele pakket van ziek zijn gaat gezondheidsvaardigheid over vier dimensies. Je moet gezondheidsvaardig zijn in de somatische dimensie, namelijk: je moet gezond eten, slapen, drinken. Je moet gezondheidsvaardig zijn in de psychologische dimensie: je moet jezelf onder controle hebben, coping. In de sociale dimensie moet je gezondheidsvaardig zijn, want ik moet met andere kunnen delen en contact hebben. En ik moet in de spirituele gezondheid vaardig zijn, wat nog de moeilijkste is. Ik moet nadenken over het leven en mijn eigen rol daarin bepalen. Vroeger hadden de simpele mensen – dat zeg ik verkeerd hoor, dat besef ik wel – in ieder geval een conventioneel kader waarbinnen heel veel bepaald was voor hen. Dat is weggevallen. Nu moet iedere burger voor zichzelf iets gaan uitmaken en dat is spannend.

**I: Ja. En dan doelt u bijvoorbeeld, spiritueel gezien zou je dat misschien allemaal in de kerk doen, op dat moment.**

R: Ja, conventionele lijnen waren duidelijk, want je deed dat en je deed zus en dat was goed en dat was slecht. Dat is weggevallen. Ik denk dat de gewone burger – en dat zeg ik dan maar voorzichtig – in ieder geval een grotere ruimte heeft gekregen, maar de beperkte burger heeft een spannendere ruimte gekregen, heeft een ruimte gekregen die veel moeilijker is. Dat denk ik.

**I: Ja, oké. Ja, aanvullend aan die verbeterpunten; op organisatieniveau… Ik wil daar dadelijk nog wel even iets over vragen nog.**

R: Jij zegt: stop, volgende. Jij bent de baas nu, ik niet.

**I: Nee, ja, nee.**

R: Ik vind het fijn hoor. Ik mag er toch van uitgaan, gewoon even.

**21.48**

**I: Ja, zeker. Nee, graag. Maar bijvoorbeeld op organisatieniveau, welke barrières ziet u** **dan nog? U noemt dus die verbeterpunten, meer tijd, en die eHealth...**

R: Nou, de organisatie is altijd lastig hè? Bedoel je dan echt *de* organisatie van de poli, bedoel je dan *de* organisatie van een ziekenhuis, *de* organisatie van de gezondheidszorg. Dat zijn drie grote lijnen hè?

**I: Ja, ik denk van het ziekenhuis.**

R: Kijk, even kijkend naar onze website, waar wij enorm op investeren , met een enorm grote ploeg van communicatieadviseurs, is: hoe moet een website eruitzien, hoe moet je beeld op de iPhone eruitzien? Wat wij nu meemaken - dat is best spannend - is dat dat beeldje op die website; daar zegt de communicatieadviseur van: dat moet zo zijn dat de eenvoudige burger direct weet wat daar gebeurt. Dus in de eerste drie regeltjes moet staan wat wij doen. Dat vind ik heel goed, heel sterk. Er wordt enorm over nagedacht dat dat op dat schermpje past, dat dat visueel aangenaam is om te lezen, dat dat vorm heeft, dat daar non-verbale communicatie in zit, vormgeving, kleur enzovoort. Als ik daarnaar kijk, dan denk ik: dat is toch niet wat wij doen, dat is veel te beperkt. Snap je? Maar de burger… Rond de vormgeving in een ziekenhuis en websites enzovoort wordt met communicatiedeskundigen juist hier heel hard gewerkt, denk ik. Ik weet niet of dat goed is.

**I: Ja. Maar u heeft dat toch ook op de site?**

R: Wat zeg je?

**I: Wat u net omschrijft van op de website, dat is toch ook nu zo?**

R: Ja, ja, natuurlijk. Nee, maar dat bedoel ik juist. Maar dat is heel bewust zo gebouwd. Dus als je vraagt, welke instrumenten heeft het ziekenhuis op organisatorisch niveau, dan is het eigenlijk niet pr, maar juist educatie en informatie, gevangen in de moderne systemen. Punt één. Daar zit nog wel een knelpunt. Kijk, onze poli’s en onze communicatie binnen die kamer of buiten die kamer telefonisch is daar nog niet op aangepast, op dat nieuwe denken. Als ik een patiënt heb van wie ik merk… Kijk, op mijn poli is het om de twintig minuten de volgende patiënt, zeg ik nu als voorbeeld. Als ik een zeer gezondheidsvaardige patiënt heb, die alles al heeft nagelezen op Google en we zijn het eens over de behandeling en we hebben een goede shared decision making, dan ben ik na tien minuten klaar. Maar iemand die dat niet gedaan heeft, die dat niet snapt, die niet weet waarover dat gaat, die drie familieleden bij zich heeft die het ook niet snappen; daar red ik het in twintig minuten niet. Het systeem laat niet toe dat ik daar zeg: jongens, ik trek even aan de noodrem, ik zet mijn trein stil en ik ga met die mensen aan de slag. Ik kan wel zeggen, bent u bereid om binnen twee weken terug te komen, zodat ik meer tijd aan u kan besteden, maar als dat meer dan tien procent is, is mijn directeur kwaad. Dan zegt hij: C., je haalt je productie niet. Dus daar zit even een spanningsveld. Daar zoeken wij dus naar tussenoplossingen. Wat doe ik dan als dokter? Dan zeg ik: oh, met deze patiënt moet H. aan de slag gaan, of moet H. aan de slag gaan.

**24.44**

**I: Oké, want dat zou mijn volgende vraag zijn.**

R: Ja. Dus dan gaan wij eigenlijk in een soort van cascade werken. Wat ik fijn vind en ook wel leuk, even voor mij dan als hoogleraar, ik praat altijd moeilijke taal. Ik praat helemaal geen Jip en Janneketaal. Nog veel erger, mijn dokters en ik zijn ons er vaak niet van bewust dat we een onmogelijke taal spreken. Ik kan met jou communiceren, want ik ben zelf onderzoeker, maar ik heb een heleboel woorden waarvan ik denk, dat moet ik niet doen. En daar let ik al wel op, want ik gebruik bij een patiënt een andere taal dan ik nu zou gebruiken bij jou. Ik ga ook explicieter praten, ik ga veel meer in beelden praten. Ik ga veel meer zoeken naar wat het beroep van de patiënt is geweest. Bij een houtbewerker ga ik voorbeelden uit de houtbewerking gebruiken om te begrijpen wat rugpijn is. Dat is heel dynamisch. Dat is heel plezant. Ik doe dat graag. Ja. Ik heb ook soms een bulldozerchauffeur. Wij zien heel veel lage rugpijn bijvoorbeeld op de pijnpoli. Daar ga ik mij honderd procent op aanpassen en dat werkt heel goed. Dan zegt hij: dat snap ik dokter. Ik neem een model erbij. Op mijn poli hier heb ik een rug-model en dan zeg ik: meneer, dit gebeurt er nu met uw rug, dit is een hefboom. “Oh ja, dat is logisch, een hefboom, dat heeft nog nooit iemand mij gezegd.” Snap je? Dus de hulpmiddelen kunnen ook figuratieve voorbeelden zijn die je op je bureau hebt staan of een filmpje: bekijk dat eens. Die dynamiek. Het lastige is, dat valt in de theoretische zin, qua categorisering, onder ‘patient education’, patiënten educatie. Patiënten educatie in een diagnostische en behandelingsmachine… Ik praat vanuit organisatorische hoek hè? Ja, dat spant met elkaar. Ik geef een voorbeeld: wij zijn aan het experimenteren, zo zal ik het dan maar voorzichtig noemen, bij de nieuwe patiënten die aankomen… Dat is niet zozeer in de palliatieve zorg, want dan kan dat niet. Palliatieve zorg is te acuut. Mensen hebben slecht nieuws gekregen, dat ze dood zullen gaan enzovoort. Maar bij pijnpatiënten doen wij aan patiënten educatie op de woensdagnamiddag. Dus iedereen die de volgende maand naar onze poli gaat komen, krijgt een pijnles. Dat is superinteressant. Ja, dat is echt een hele mooie oefening. Dat deed ik al twintig jaar geleden, maar dat doen we nu ook hier in het … (naam ziekenhuis). Wat zit daar als probleem? Dan komen er tien patiënten die de volgende maand op de intakepoli komen, als voorbeeld. Die brengen hun partner mee. Twintig mensen. En er is een verpleegkundige, één van de fysiotherapeuten die dat doet en soms een dokter. Wat zien we? Dat het eigenlijk niet goed werkt. Dat is onze eerste indruk. Waarom? Iedere patiënt heeft een andere ziekte, dus men herkent zich niet in een algemeen verhaal. Dat is al het eerste punt. Dat kun je dus ook gezondheidsvaardigheden noemen, want wij brengen generiek: pijn wordt veroorzaakt door, maar patiënten zitten met hun knie, met hun enkel, met hun rug, met hun kanker, met dit, met dat. Daarom denk ik dat de … visie (naam ziekenhuis) en aanpak heel goed is: persoonsgericht. Daar ga je niet aan collectieve educatie doen, maar dan ga je naar een specifieke educatie. Daar hebben we dan eigenlijk H. en H. voor, die dan de rol hebben van casemanager, waarbij we zeggen: H., bij deze patiënt moet jij uitleg gaan geven over dit. Die neemt meer tijd daarvoor. Dat is de bedoeling.

**I: Oké, op die manier.**

R: Dus dat is een behoorlijk dynamisch proces. Er zit nog veel inventiviteit in. Ik denk dat we een aantal testen gedaan hebben om te kijken van: wat zou nu werken en niet werken? Dat is de oefening. Is daar al common sense, even heel praktisch? Nee. Moet daar veel common sense zijn? Nee, want ik zou het liefste hebben dat mijn dokter en verpleegkundige zo persoonsgericht zijn dat ze zich aanpassen aan de capaciteiten van de persoon die tegenover hen zit.

**28.37**

**I: Ja. Ja. Wat eigenlijk ook in het begin van het gesprek… Wat u zei van: dat is eigenlijk wel een voorwaarde hier.**

R: Het is een voorwaarde om hier te werken, ja. Iemand die in zijn eigen wereld leeft en niet over de brug kan komen… We tekenen dat altijd in onze educatie. Als iemand hier binnenkomt, dan zeggen wij, de ik-jij brug… Ik weet niet of je die kent. Het is simpele psychiatrie hè, even heel praktisch. Maar de ik-jij brug wil zeggen: ik ga als dokter tot hier, de patiënt komt als patiënt tot daar, of de cliënt of de gast, dat maakt even niet zoveel uit. Wanneer die dokter te veel over de brug moet stappen of de patiënt te veel aan de andere kant van de brug komt, heb je een onevenwichtige balans en heb je dus een situatie waar je wat mee moet als professional. Dat leren wij aan de mensen. Shared decision making zit mooi in de middenbaan, in het ideale geval, in de ideale wetenschappelijke wereld. Shared decision making moet natuurlijk leren dat die dokter meebeweegt over die middenlijn. Hij moet zich bewust zijn van het feit dat hij over de middenlijn gaat. Een te meelevende dokter is voor die dokter slecht, want die houdt dat niet vol, die krijgt een burn-out. Dat is slecht voor die patiënt, want die patiënt denkt: goh, die dokter regelt het wel voor mij. Die leert juist geen gezondheidsvaardigheden, want men past zich continu aan. Een patiënt met te veel gezondheidsvaardigheden is bedreigend. Daar gaat die dokter van zeggen: ik wil u niet meer zien, want u zit altijd te noteren, u verwijt mij heel veel, dit en dat. Dat is een slechte relatie. Dat kan een teken zijn van weinig gezondheidsvaardigheden. Dus deze brug in ons model is essentieel. En waarom is dat zo essentieel? Omdat in onze populatie een derde van de patiënten, denken wij, beperkte gezondheidsvaardigheden heeft, want anders ontwikkel je een aantal chronische ziekten niet. Een derde heeft co morbiditeit in de betekenis van dat daar psychiatrische co morbiditeit is. In onze populatie van het … (naam ziekenhuis) hè, derdelijns. Wij zien niet eerstelijnspatiënten hè, dat moet je ook even weten. Een derde van onze patiënten heeft borderline kenmerken, volgens de DSM-classificatie. Daar kun je geen normaal gesprek mee voeren. Dus wat hebben wij gedaan? Voor deze groep van patiënten hebben wij een aparte getrainde klinische psycholoog, die mee pijnpoli doet met deze patiënten, om mij onder controle te houden. Dat is punt vijf. Dus die psycholoog gaat met mij in de polikamer zitten. Die zit niet aan mijn kant, die zit niet aan jouw kant, die zit tussen ons in en die dwingt eigenlijk om met ons samen er goed uit te komen. Dat kun je opzoeken als je dat wil, dat is T. Dat is best een kluif qua werk. En een derde van de mensen heeft een normale kwalificatie. Daar moet je dus passende acties op ondernemen. Deze groep, dus de mensen met psychiatrische co morbiditeit, zo noem ik het dan maar, in de brede zin van het woord, is een ongelooflijk moeilijke groep om te detecteren, die een bijzondere benadering vragen qua aanpak, zowel van de professional als van de structuur van de gezondheidszorg. Daar moeten we dus enorm op gaan studeren: hoe doe je dat?

**I: Oké. En als ik inhoudelijk inga op één van die groepen; communicatie is dus moeilijker als ik u goed begrijp.**

R: Dat is anders. Ik zeg niet dat het moeilijker is, maar de professional is niet getraind op dit type patiënt in de generieke zin. Een huisarts heeft één of twee patiënten op een praktijk van duizend mensen. Dus als je die tegenkomt, zegt die huisarts: goh, dat vind ik niet fijn, dat die komt. Als wij dat analyseren; dat hebben we nagekeken op onze poli. We hebben natuurlijk een geconcentreerde patiëntengroep, mensen die juist omwille van honderden redenen geen effect hebben en daarom staan ze hier. Over die groep mensen gaat de discussie. Dat zijn de mensen die overal de deur is gewezen en geen oplossing is gevonden voor hun probleem… Ik zou die bij uitstek niet noodzakelijkerwijs communicatiearm of weinig gezondheidsvaardig noemen. Ik denk dat ze verkeerde gezondheidsvaardigheden hebben. Dat is nog iets anders dan wat jullie noemen. En daar zit het probleem ook. Ik denk… Maar ik heb nu vandaag geen meetinstrument om te zeggen dat iemand verkeerde of beperkte gezondheidsvaardigheden heeft. Wat ik lastig vind; het woord “verkeerd” heeft al een heel erg beoordelende waarde en dat wil ik er juist niet aan geven. Iedereen is gebouwd zoals die is en is opgevoed zoals die is. Daar zit een beetje de knoop…

**I: Ja. Ja. Kunt u een voorbeeld geven van verkeerde gezondheidsvaardigheden bijvoorbeeld?**

R: Een voorbeeld van verkeerde…

**I: U geeft net aan dat het met name bij deze groep niet zozeer beperkte gezondheidsvaardigheden zijn, maar verkeerde gezondheidsvaardigheden.**

R: Heel praktisch, mensen die denken dat door een tablet pijn weggaat. Terwijl dat eigenlijk zo is door te bewegen, gezond te leven, je gewicht met 20 kg te laten verminderen en te stoppen met roken. Dat is een verkeerde attributie. De attributie, daar zit het hoofdprobleem. Zo heet dat bij psychologen hè, attributies. En dat aanleren, van: mevrouw, deze attributie is verkeerd… Daarom zeg ik, het is niet weinig gezondheidsvaardigheden, maar ze hebben het gewoon verkeerd aangeleerd gekregen. Als jij in de … woont (voorbeeld van een wijk) en in alle huizen wordt door alle mannen gerookt, dan ben je niet communicatiearm, niet gezondheidsarm in vaardigheden, maar je hebt het nooit juist gezien. Zo proberen wij daarnaar te kijken. En dat is een dynamisch proces, wat vastgeroest is in concepten en in attributie. Ik zie bij heel veel mensen, als ze dan naar een cursus gaan of naar een langdurige revalidatie of anderzijds, dat als je die mensen drie maanden hard traint dat toch een deel het heel goed gaat doen. Dat vind ik fijn.

**34.44**

**I: Ja, oké. Dus er zijn wel degelijk mogelijkheden.**

R: Ja, natuurlijk. Dat bestaat ook, maar zeer intensief, precies waar ik mee begon eigenlijk hè: krijgen we er tijd voor, krijgen we er een ruimte voor en is er een speciaal budget met een codering waarachter staat: patiënt met beperkte gezondheidsvaardigheden: code 27.8, dus alles mag maal drie qua time. Daar gaat de zorgverzekeraar niet mee lachen.

**I: Nee. Nee. Oké. Dus bij deze groep… Om dat te verbeteren, wat u zei, dan zit er iemand bij.**

R: We leveren specifieke deskundige zorg.

**I: Ja. En bij deze groep?**

R: Wat doen we bij de anderen? Bij de anderen stoppen we er meer tijd in bijvoorbeeld, nemen we contact op, bellen we terug enzovoort, enzovoort.

**I: Oké. Dus u heeft die lijst die in eerste instantie iedereen krijgt. Dan merkt u dat iemand die niet heeft ingevuld of verkeerd heeft ingevuld. Dat gaf u aan hè, van: nou dat is in ieder geval al een indicatie. Ik zeg maar even wat, die mensen komen dan op consult of zo?**

R: Ja, zeker. Ja. Precies zoals jij het zegt, dan ga ik H. en H. (verpleegkundigen palliatieve zorg) veel meer inschakelen. Ja, natuurlijk. Ik heb daar de tijd niet voor en ik krijg daar ook niet de tijd voor.

**I: Oké. Dus dat is dan een oplossing, op die manier. Oké.**

R: Ja en ook… Kijk, het gezondheidsmodel; daarom noem ik die vier dimensies. De dokter heeft een erg grote rol in de somatische dimensie en anderen moeten in de andere dimensies een rol hebben. Wat hebben wij praktisch uitgebouwd in de palliatieve zorg? Kijk, de psychologische dimensie, daar hebben we een klinisch psycholoog voor. De sociale dimensie, daar hebben we een maatschappelijk werker voor, want die gaat kijken: is er thuis een hulpmiddel nodig, wat moeten we daar mee? Dat is heel intensief uitgewerkt. De vierde dimensie is dan de spirituele. Daar hebben we de consulent spirituele zorg voor. Dat zijn de vier personen die daarvoor rondlopen. De meeste van deze mensen worden geactiveerd vanuit ons wekelijkse MDO.

**I: Oké. Dat heb ik ook gehoord van H. en H. inderdaad.**

R: Ja, precies. We hebben een wekelijks MDO. Dat wordt voorgezeten, bewust, door een huisdokter die we daarvoor inkopen hier. We betalen die huisdokter om hier te komen werken, om dat MDO voor te zitten en om juist deze problemen, die we dan detecteren; dat is nog wel een punt dat ik misschien wat onderbelicht heb gelaten, maar kijk, de gezondheidsvaardigheden zijn niet alleen bij de patiënt niet aanwezig, maar ook bij de mantelzorgers. Dat is meestal een collectief gebeuren. Het kan ook omgekeerd zijn: de patiënt snapt het niet, maar de mantelzorger wel. Dan is het niet zo moeilijk. Dan gaan we aan de mantelzorger veel uitleg geven en zeggen: legt u thuis nog een keer uit dat… Maar als het hele systeem daarin zit, dan is dat bij uitstek de patiënt waar H. en H. van zeggen: ho, die moet naar het MDO en hier gaan wij een plan maken over hoe we verder met die persoon omgaan. Dan ga je natuurlijk orgelregisters uittrekken die passend zijn bij die patiënt in zijn leefomgeving met zijn omstandigheden. En daar zitten ook… Dat vind ik wel fijn, dat dat beter en beter wordt uitgevoerd. Nu zien we dat consulenten spirituele zorg… de geestelijke verzorgers moet je zeggen. Maar die beginnen ook te bewegen in de eerste lijn, om maar een voorbeeld te geven. Daar werken wij hier wel aan. We hebben een aparte stichting gemaakt. We hebben nu negen geestelijke verzorgers in de thuiszorg en die kunnen bij mensen thuis langsgaan om dat verder op te vangen. Die mensen – en dat vind ik wel heel fijn – zoeken heel erg naar de betekenis van dingen, van: wat betekent dit nu voor u? Als je personen met beperkte gezondheidsvaardigheden hebt; die heeft ook een betekenis, maar een andere dan een gezondheidsvaardig persoon. Daar kun je heel mooi rond werken. Dat is een heel verpersoonlijkt traject, waar we eigenlijk heel blij mee zijn. We zien ook… En dat is wel een punt. Kijk, in die studie van J. hebben wij een model uitgebouwd waar wij als centrum vooral diagnostisch werken, dus wij gaan patiënten één keer zien, daar een heel plan voor maken. Daar nemen we ruim onze tijd voor. We nemen daar gemiddeld een uur voor. Dan maken we een plan en we zeggen tegen de huisarts en tegen de vrijwilliger en tegen de buddyzorg, als die aanwezig is bij kankerpatiënten: jullie moeten het volgende gaan doen bij deze persoon. Die uitvoering willen wij heel erg in die eerste lijn terugleggen. Waarom? Anders slippen we dicht. Ik bedoel, ieder van ons heeft maar dertig werkuren, veertig werkuren. Dan kan ik misschien twintig patiënten doen in een week. Dat is niet de bedoeling hè? Om dat systeem niet te laten dichtslibben proberen we het juist heel erg bij de huisartsen en de praktijk van de huisartsen, de POH, de praktijkondersteuner, te zeggen van: ga ermee aan de slag. Lukt het niet, bel dan terug. Dus wij hebben ook een transmurale teamstructuur om te zeggen van: als je er niet uitkomt, bel dan even. Daarin zijn we heel laagdrempelig. Niet voor de patiënt, maar wel voor de patiëntenzorgverlener. Die mogen eigenlijk 24/7 bellen hiervoor. Ik heb het natuurlijk niet gemeten aan de achterkant, maar zijn we op zich tevreden over dit systeem? Ja. Wat ik een lastige vind, dat is nog iets wat ik met J. zal gaan doornemen in deze studie, is dat afhankelijk van het type team eigenlijk de plek om te gaan meten bij een palliatief team niet de goede plek is voor deze studie, denk ik. Dat is een aparte discussie, maar die moet ik niet met jou voeren, niet in dit interview. Wat is eigenlijk het signaal? Eigenlijk denk ik dat de recruitment van de patiënten door huisartsen zou moeten gebeuren idealiter. Die kent de patiënt twintig jaar, die weet precies hoe hij zal omgaan met het wel of niet horen van de boodschap, of die aankomt, hoe die erop reageert. Dus ik denk dat deze studie…

**I: Die kan die inschatting misschien beter maken.**

R: Nou, dat sowieso, maar de aanvliegroute… Kijk, wij zien die patiënt één, maximaal twee keer. De film moet genomen worden op het tweede moment dat we die patiënt terugzien, maar dat gebeurt thuis, bij die huisdokter. Snap je? Dus dat is even… Maar daar zal ik J. wel op aanspreken, want we hebben met E. daar een heel debat over gevoerd. Dat had ik in het begin al als gevoel hoor. Helemaal in het begin van de studie, een half jaar geleden, heb ik al tegen de studiecoördinatoren gezegd van: ik denk dat de aanvliegroute anders moet. Maar dat is even… Ik weet niet of dat voor dit interview belangrijk is.

**41.10**

**I: Ja. Oké. Mag ik nog één vraag stellen, want we zijn eigenlijk door de tijd heen.**

R: Ja, je hebt nog vijf minuten. Ja, dat is de promovendusklok. Dan gebeurt dit. “Mag ik nog één vraag stellen?” Heel goed. Ik heb daar een klok, dus ik zie een andere tijd.

**I: Ah, precies. Dat is met opzet zo.**

R: Dat is met opzet.

**I: Oké. Oké. Ja, het woord is al een paar keer gevallen, gezamenlijke besluitvorming. Ik heb ten slotte de vraag: gezamenlijke besluitvorming in die groepen die u beschrijft, is die…**

R: Die is driemaal verschillend.

**I: Oké. Die is driemaal verschillend.**

R: Totaal verschillend.

**I: Kunt u daar iets meer over vertellen?**

R: Kijk, wat ik lastig vind in het gesprek; kijk, dit is een voorbeeld dat ik haal uit de pijnwereld en niet noodzakelijkerwijs uit de palliatieve wereld. Dat is even een premisse die je moet meenemen. In de palliatieve wereld hebben we dit nog niet uitgezocht, dus dat is nog wel een lastige. Dus wij weten helemaal nog niet wat de psychiatrische co morbiditeit is bij palliatieve patiënten op een poli of op een ziekenhuisbed. Dat is een heel groot probleem. De shared decision making in de drie modellen is enorm verschillend. Je decision making maakt de verwachting dat je twee gelijke partijen hebt die het besluit gezamenlijk kunnen nemen. Dat verwacht dus ook heel veel gezondheidsvaardigheden. Deze groep – dat noem ik maar heel eventjes oneerbiedig, maar meer vanuit de psychiatrische kant: de borderline patiënt – heeft in zijn hoofd: ik wil bij deze dokter dit bereiken. Daar is geen shared decision making. Deze patiënt zit hier, als dit de dokter is. Die zit gewoon aan de verkeerde kant. En er is geen stap te maken. Dus mijn pleidooi is: deze groep op een aparte wijze met deskundigen en in dit geval zijn dat de psychiatrische verpleegkundigen en psychologen, GZ-psychologen, die gewend zijn om hiermee om te gaan en die ook weten hoe je een punt moet maken bij deze patiënten. Dat is het probleem van landlopers, het probleem van psychiatrie. Die mensen hebben in hun hoofd zitten: dit wil ik. Wat jij zegt in technische zin vinden ze niet belangrijk, zelfs niet als je dokter bent. Zij gaan ook niet zeggen: oh, dokter, vertel eens wat ik ga doen nu? Dus vandaar, die groep eruit halen zuivert het terrein wel enorm. Dus eigenlijk zeg ik daarmee dat de identificatie van de patiënt in die vier dimensies een essentieel onderdeel is, ook het kennen van de patiënt is zijn voorgeschiedenis, zijn autobiografische voorgeschiedenis om te begrijpen tot welke groep hij behoort en om dan aan de slag te gaan met die patiënt. Maar dat vraagt dus deelprogramma’s, denk ik. Dus als je dat gewoon generiek – wat jullie aan het doen zijn – op de hele populatie loslaat, dan denk ik dat het effect lager is dan dat je naar deelpopulaties gaat zoeken. Dat zou mijn antwoord hierop zijn. En dat is heel mooi, want dat betekent enerzijds dat dit de eerste opstap is, maar anderzijds dat die persoons-/autobiografische informatie essentieel is om te begrijpen hoe het werkt. Ik praat half als onderzoeker ook hè? Dat hoor jij ook wel aan mij hè? Maar dat is ook mijn rol. Ik moet hier leidinggeven om binnen vijf jaar dit goed voor elkaar te hebben, die gezondheidsvaardigheden. Ik denk dat we al een hele mooie weg hebben afgelegd, vind ik zelf. Ik denk dat de meeste professionals, juist in de palliatieve zorg, weten: wij moeten aandacht hebben hiervoor. Ten tweede, Bruera twitterde een uur geleden… Ik weet niet of je het gezien hebt. Ken je Bruera? Dat is één van de leading guys in de wereld van de palliatieve zorg. Die heeft net getwitterd. Er is een prospectieve trial geweest waar de dokters rechtop stonden en een ander deel van de dokters zat. Er is gekeken bij wie de boodschap het beste overkwam bij de patiënt. De dokter die zit heeft de beste boodschap. Dat is wat wij uitstralen. Ik zeg tegen al mijn dokters, al hebben ze maar drie minuten tijd: ga zitten. Geen deurgesprek. Geen ganggesprek. Zitten op een stoel bij de patiënt. Dat is misschien nog het belangrijkste. De laatste zin die ik dan zeg in het interview. Maar ik heb die ervaring absoluut, dat dat het belangrijkste moment is.

**45.23**

**I: Interessant. Ja.**

R: Ja. Ik zeg maar hoe ik ernaar kijk. Dat is het onderdeel van het interview. Het zal weinig passen bij de andere, denk ik.

**I: Nou, dat weet ik niet. Dat weet ik niet.**

R: Ik ben benieuwd.

**I: Dank je wel.**

R: Had je nog veel vragen over? Moeten we nog een verlenging op een ander moment doen? Dat laat ik aan jou over.

**I: Die sla ik natuurlijk nooit af, maar ik verwacht van niet.**

R: Als je mij nog wil spreken, moet je het met L. afspreken. Hoor even van J. en anderen hoe ver je moet gaan. Dat is denk ik het belangrijkste.

**I: Ja. Ja, precies. Is het goed dat J. misschien contact opneemt met u over…**

R: Ja, ik heb nog een mail staan van haar die ik nog moet beantwoorden. Ze heeft mij een mail gestuurd, dus dat klopt wel. Daarom. Ik was deze week met E. aan het kijken van: wat is nu de beste weg van dat traject? Dat is ook zo. Ik hoor dat rekrutering van de patiënten overal een probleem is.

**[48.18 – einde opname]**

Z4d04

I: interviewer

R: respondent

0:58

**I: Mijn eerste vraag is altijd heel breed natuurlijk, wat is uw rol in de zorg voor patiënten in de palliatieve fase?**

R: Ja, mijn rol is meer als alleen patiëntenzorg, mijn rol is, binnen in ieder geval het Radboud ziekenhuis deels als senior lid van het palliatieve team. Daarbij heb ik naast de patiëntenzorg ook onderwijstaken en opleidingstaken en research. Maar we zullen nu alleen hebben over de patiëntenzorg. Binnen de patiëntenzorg heb ik natuurlijk door mijn senioriteit ook een functie in het bewaken van de kwaliteit van zorg, zowel op de dag dat ik klinische dienst heb als de dagen dat ik geen klinische dienst heb. Dat betekend dat ik meestal bij ieder ochtendoverleg ben, dat ze mij op ieder moment kunnen consulteren als er problemen zijn en dat ik consequent ook aanwezig ben in het multidisciplinair overleg waarin alle patiënten besproken worden. Als we het dan puur over de directe patiëntenzorg hebben, als ik de directe zorg van de patiënten heb, heb ik zelf zorg voor de patiënten die wij in onze eigen bedden hebben opgenomen, waarbij ik op de dag dat ik de klinische dienst heb ook hoofdbehandelaar ben, of op dat moment de behandelaar van de patiënt. Ik heb mijn verantwoordelijkheden niet in diensten, die doe ik niet meer wegens mijn senioriteit. Ik heb verantwoording om de consulten op de best mogelijke manier uit te voeren, en ik heb verantwoordelijkheid naar mijn poliklinische patiënten, zowel natuurlijk face-to-face als dat ik ook andere afspraken heb gemaakt voor telefonische consulten, en alles volgens de richtlijn palliatieve zorg zoals die weergegeven staan in principe, in [www.pallialine.nl](http://www.pallialine.nl). Bij gebrek aan een hele duidelijke vakgroep palliatieve zorg hebben we dat omarmd als casus, als onze richtlijnen. En als ik daarvan afwijk, dan doe ik dat om duidelijke redenen, of direct door de patiënt veroorzaakt, of anderszins. En daarbij gelden de principes doe goed en zorg dat je geen kwaad doet.

**I: Oké, ja, ja. U bent dus centrale zorgverlener op een zeker moment van patiënten, u had het net ook over, en daar wilde ik graag op inhaken, over de best mogelijke manier? Wat is volgens u de best mogelijke manier?**

R: Nou, dat is, net als bij een raamwet hé, in een raamwet geef je aan, de best mogelijke manier, maar de invulling die kan variëren. De invulling kan alleen al variëren of iemand wel of niet alleenstaand is, of hij wel of geen goeie verhouding heeft met zijn huisarts, of hij wel of geen thuiszorg heeft, als ie thuiszorg heeft, of hij van een redelijk niveau is, een hoog niveau of een laag niveau, dus, de best mogelijke zorg, kan wezenlijk variëren. Het kan ook variëren met de aandoening. Als ik een slow release opiaat voorschrijf, terwijl die persoon diarree heeft, ja, dan werkt het als een immediate release, een slechte immediate release tablet, [5:00] dan is zijn pijnstilling nog niks, terwijl het volgens de richtlijn, die het nu nooit over diarree heeft, een uitstekende behandeling is.

**I: Oké, ja.**

R: Dus, de best mogelijke behandeling, is net als de wetgeving, ja, volgens de normen van de vakvereniging, dus die pallialine, met daarbij aangevuld dat ik mijn persoonlijke best doe om het aan te passen aan de patiënt, aan te passen aan zijn sociale omgeving, aan te passen aan zijn zingeving.

**I: Ja, precies, dus dan bedoeld u afwijken van die lijn…**

R: Ja, maar beredeneerd.

**I: Precies, kunt u een voorbeeld geven wanneer u af…**

R: Ja, dat had ik net, net wat ik zei, als je een pijnbehandeling geeft, om het maar heel eenvoudig te houden, als je opiaten geeft, zal dat de tweede stap zijn, de eerste stap zal zijn eenvoudige paracetamol, al dan niet aangevuld met (moeilijk te verstaan; 6:07). De tweede stap, in tegenstelling tot de WHO-pijnladder is sterkwerkende opiaten, maar als je dan sterkwerkende opiaten geeft, moet dat na instellen een langwerkend opiaat zijn aangevuld met kortwerkend opiaat voor incidentpijn. Maar als iemand geen goed werkende (moeilijk woord, moeilijk te verstaan; 6:33) heeft, dan moet ik een pleister geven. Als iemand nierfunctiestoornissen heeft, moet ik bedenken of dat niet via de nier geklaard wordt. Als hij andere medicamenten gebruikt die het CYP 450 systeem in de war gooien en ik geef fentanyl wat daardoor geklaard wordt, dan moet ik, of een ander opiaat nemen, of mijn behandeling daarop aanpassen. Als ik ‘m oraal behandel en hij heeft plotseling diarree, dan moet ik voorbereid zijn dat een langwerkend middel niet meer werkt. Als hij met zijn fentanyl*-*pleister regelmatig naar de sauna gaat, moet ik weten dat hij tijdens dat saunabezoek een hogere dosis krijgt. Ja, dat is puur nog maar het fysieke element. Maar als iemand klaagt over pijn, en ik heb een goede pijninventarisatie gedaan, en zeg, de pijn wordt vooral veroorzaakt omdat hij niet in staat is te lopen, laat staan een triatlon te doen, dan zal iedere pijn verschrikkelijk zijn, totdat hij weer kan lopen. Dat betekent dat ik die pijn misschien op een andere manier moet aanpakken als ik weet dat hij nooit meer aan lopen toekomt, of maar beperkt, of als de kans daarop heel klein is, want, zou ik dan de gewone pijnrichtlijn, de directe pijnrichtlijn volgen, dan blijf ik ‘m doorbehandelen, want hij blijft klagen, tot hij geïntoxiceerd wordt, want lopen kon hij toch niet meer. Dus, het is van belang dat je al de aspecten die van invloed zijn daarbij betrekt. Het tweede wat je erbij moet betrekken is, ik kan de persoon heel goed behandelen, maar als zijn omgeving…

**I: Oké, dat was mijn volgende vraag inderdaad…**

R: Als zijn omgeving het gevoel heeft, het moet dit zijn of het moet dat zijn, bijvoorbeeld, de pijnbehandeling moet zo gebeuren dat zijn cognitie intact blijft, want we hebben er zo’n last van als hij minder helder is, dan kan de patiënt het misschien heel prettig vinden dat hij minder helder is, maar de omgeving heeft er last van. Maar dat moet ik afwegen, moet ik de omgeving dat duidelijk maken, dat het voor de patiënt van groot belang is om dat te doen, en ze daarbij leren dat te accepteren, of moet ik misschien toch eens goed naar die omgeving luisteren en zoeken naar een pijnbehandeling die die man zijn cognitie wel laat behouden, ook al vindt ie dat niet zo erg, want dan kan tenminste zijn omgeving er ook beter mee omgaan en dat komt ook weer ten goede aan de patiënt. Dus, die verhoudingen spelen dan. Wat nog meer speelt, als ik weet dat in de nabije toekomst een patiënt zodanig derieert dat hij niet meer zal drinken, dan zullen onmiddellijk zijn nefrogeen geklaarde middelen stijgen in het spiegel, en…

**I: Zijn nieren?**

R: Ja, bijvoorbeeld, als iemand niet meer drinkt, dan intoxiceert zo iemand op morfine [10:00]. Als ik dat van tevoren kan beredeneren, dan moet ik ‘m niet op morfine zetten. Of ik moet op tijd dat beleid aanpassen, of ik moet de huisarts waarschuwen, als hij zijn oxycodon niet meer kan slikken, moet je ‘m geen morfinepomp geven, maar geef ‘m dan iets anders. En dat staat allemaal niet in de richtlijnen. Maar dat betekent dat mijn invulling van proactieve zorg, ook inhoudt dat ik kennis en begrip van de toekomst heb. Als ik, van iemand hoor dat hij alleenstaand is, maar dat allemaal heel goed kan regelen, dan moet ik ook beseffen als hij in het beloop van zijn fase te verwachten is dat hij steeds minder kan, wat misschien ook cognitief minder wordt, dat dan al de ingewikkelde schema’s die ik ‘m geef die hij nu aankan, straks niet meer aankan. En dat ik op tijd in de gaten krijg, dat het afneemt, dat er misschien hulp ingezet moet worden, ook al is het iemand die zijn zelfstandigheid heel hoog schat en heel belangrijk vindt, ja, dan kom ik in de toekomst ergens in de knoop, van, moet ik met bemoeizucht regelen dat hij geen problemen overkomt, dat hij zichzelf intoxiceert of tekortdoet, of moet ik dat maar toestaan en, hé, want we hebben ook het recht om, op onze eigen manier te rotten, als iemand kiest om te leven onder een brug, moet ik dat toestaan, ook al is dat niet handig als je in een eindfase zit van een pancreas carcinoom. Dus, dat zijn afwegingen die niet in een richtlijn staan, maar waarbij wel rekening gehouden wordt met de authenticiteit van de patiënt, met zijn sociale omgeving, met zijn emotionele beperkingen, of wensen, etc. etc. Dus, het puur goed medisch technisch handelen is in de palliatieve zorg te beperkt.

**I: Oké, ja. Oké. Als ik mag inhaken op wat u aangaf, en dit moet ik eerst even introduceren. Ik sprak C. vanmorgen, ik heb H. en H. ook al gesproken, en die zeiden eigenlijk, we zien patiënten hier één keer, soms twee keer, maar relatief kort heb ik begrepen. En uw advies, uw plan, gaat vervolgens naar een huisarts en die pakt het eigenlijk weer op. U noemde ook op een gegeven moment dat u het dus ook aangeeft aan de huisarts, maar hoe werkt dat precies? Met de andere zorgverleners?**

R: Eigenlijk zit er een mening achter uw vraag.

**I: Oh echt? Dat is niet mijn intentie. Overigens moet u mij ook verbeteren als ik iets verkeerds zeg.**

R: Er zit een mening achter die vraag, of een mening, je kunt instappen in een organisatiemodel wat er is, of een mening, ik twijfel of zo’n organisatiemodel bestaat, eigenlijk zit er een vraag achter je vraag hé.

**I: Oké?**

R: Als ik met een patiënt te maken heb, waar allerlei vormen van zorg gecoördineerd moeten worden, dan is één van mijn vragen, hoe is uw relatie met uw huisarts? Als hij zegt, niet goed, dan weet ik al dat ik daar problemen mee krijg. Als ik zeg maar zorg, en handelingen, en vooruitzichten, enzovoorts, aan een huisarts meegeef, want, of die huisarts er nu bewust of onbewust is van de mening van de patiënt dat er een slechte relatie is, er ontstaat op het moment dat ik de zorg doorgeef, transfer, ontstaat er een breuk. Een breuk in de continuïteit, in ieder geval een breuk die de patiënt ervaart, omdat die zegt, ja, die is niet zo goed. Dan heb ik minstens de verantwoordelijkheid om bij de huisarts te polsten, van, hoe zit dat in uw visie? En hoe gaat u dit weer zodanig oppakken dat de patiënt daar andere gevoelens over heeft. Ik ga ook vragen aan de huisarts, wilt u dit wel en kunt u dit wel [15:00]? Er is namelijk ooit een onderzoek geweest in Nijmegen, waarbij gevraagd wordt, de persoonlijke palliatieve zorg, hoeveel van de artsen vinden dat ze dat ook moeten uitvoeren, dus ook persoonlijke zorg moeten geven? En daarvan zegt 70%, ja, ook als het buiten mijn kantoortijd valt, ook als het buiten mijn dienst valt…

**I: En dan bedoelt u huisartsen…**

R: Ja, huisartsen, maar dat geldt ook voor specialisten, dat maakt allemaal niet uit.

**I: Oké, ja.**

R: 30% zegt, nee, ik doe dat in een organisatiemodel, als het buiten mijn diensttijd valt, buiten mijn kantoortijd valt, dan valt het gewoon bij de acute huisartsenpost en ik zorg wel dat er een briefje ligt van hoe het met de patiënt zit en welke keuzes gemaakt zijn. Van de 70% van de huisartsen die de persoonlijke palliatieve zorg willen geven, daarvan zegt, ja, palliatieve zorg is eigenlijk de jus in mijn vak, zegt 40%, maar ik kan het organisatorisch niet waarmaken. Ik heb kinderen, ik heb een partner die ook werkt. Er zijn momenten dat ik gewoon niet afwezig kan zijn, dus ik kan het niet waarmaken, die persoonlijke palliatieve zorg, 24/7. En 30% van de huisartsen zegt, dat kan ik. Dus het overdragen aan de huisarts is ook, van, kunt u dat ook georganiseerd krijgen? En hoe? Het maakt me niet uit of u het persoonlijk doet, of via de huisartspost, maar er moet iets georganiseerd worden. En verder, zal ik aangeven wat op dat moment van mijn intake er allemaal speelt bij patiënten. Dat is puur het fysieke, maar dat zijn ook sociale domein, emotionele domein, zingevingsdomein, en ook ons idee over welke toekomstige problemen er kunnen ontstaan en welke sterfscenario’s. Dat omvat natuurlijk ook de kans dat de mantelzorger overbelast raakt, of misschien nu al overbelast is. Hé, dus ja, en dan bespreek ik ook altijd met de huisarts, hoe wilt u dit in de toekomst gaan doen? De patiënt had deze wens, bijvoorbeeld, de patiënt zegt, prima, de huisarts en ik, daar heb ik een goede band mee, ik ben blij als de huisarts weet wat de problemen in de toekomst kan zijn, als hij maar de trucjes krijgt om ze dan op te pakken, heeft u daar voldoende aan? Als u daar niet voldoende aan heeft, kunt u ons 24/7 bellen. Het kan ook zijn dat die patiënt vraagt aan mij, ja, ik zou toch wel willen dat u met mij contact houdt, en daarmee vraag ik een huisarts, ja, daarmee lopen we het risico dat er een driehoek ontstaat van twee hulpverleners en een patiënt met zijn familie, en die driehoek heeft altijd in zich, de kans dat er van mijn kant iets gezegd wordt, terwijl u de verantwoordelijkheid heeft. Het is, hoe gaan we dat met elkaar goed regelen, dat het handig is, en vindt u dat wel prettig en handig, of vindt u het juist heel onprettig? Dus, dat overdragen van de, gewoon, in een organisatiemodel, is niet mogelijk, of is niet vast te leggen in een bepaalde manier, want, dat organisatiemodel past niet, want ieder moment kan het anders zijn. Bijvoorbeeld, ik kan een huisarts bellen, de patiënt heeft duidelijk aangegeven dat hij in bepaalde omstandigheden, euthanasie zal verzoeken en zal hij, hij wil dat bespreken met u. En die huisarts, ja, als het een actueel verzoek is heb ik een probleem, want zaterdag ga ik met vakantie. En dan blijft het echt tot na mijn vakantieperiode, want ik kan het niet overdragen aan een dienstdoend of aan een collega. Dan zit je al onmiddellijk, als het een actuele wens was, met een probleem. Dus, dat je organisatorisch wel weet hoe het moet, iemand in een thuissituatie moet je niet proberen om vanuit het ziekenhuis allemaal te regelen, dat moet je proberen, hoe dat prettig en handig ligt bij de huisarts, botst op allerlei praktische bezwaren. Dus, de uitdaging om dat op te lossen en dan kan het soms linksom of rechtsom gaan. En ja, ik heb een aantal patiënten die ik wel chronisch vervolg, omdat (moeilijk te verstaan) [20:00], of omdat ik zeg, ja, hij is nog niet gestabiliseerd met bijvoorbeeld bepaalde vormen van pijnproblemen of bijvoorbeeld met zingevingsproblemen of wat dan ook en als ie stabiel is dat ie dan toch definitief overgaat naar de huisarts. Of dat ik de huisarts erbij betrek en steeds meer zorg laat geven om die stabiliteit te garanderen, of een patiënt zegt, ja, ik het zo’n mot met de huisarts, ik moet echt eerst een nieuwe huisarts zoeken voor ik, voor die zorg overgedragen kan worden. Tot en met patiënten toe die helemaal niet in Nederland wonen en als Nederlander even teruggekomen zijn omdat ze zich niet goed voelden. En dan blijkt in één keer acuut dat ze, in Frankrijk wel een huis hebben maar niet in Nederland wonen, dat ze in Frankrijk, of geassimileerd zijn om daar aan de gezondheidszorg zich over te geven, dat ze nu, acuut, eigenlijk thuisloos zijn, als ze dat in Nederland zijn en daar ook weer een arts bij moeten zoeken, maar zelfs hun thuishonk niet hebben, dat ze eigenlijk vermoeden dat ze wel richting euthanasie gaan en dat in deze situatie die helemaal onmogelijk is. Dus je, je komt nogal eens voor punten te staan die bijzonder zijn en alle bijzondere keuzes die mensen ooit eerder gemaakt hebben.

**I: Ja, ja. Bijzonder ingewikkeld lijkt het me…**

R: Mja, bijzonder uitdagend.

**I: Ja, dat is inderdaad een mooie positieve twist ☺. Als ik u mag vragen, wat meer gericht op uw gesprekken met patiënten, u noemt zelf al het fysieke, u heeft het ook gehad over het sociale aspect wat u bespreekt met uw patiënt en ook het spirituele en psychische…**

R: Dimensies…

**I: Ja, dimensies…**

R: Het spirituele is natuurlijk maar een onderdeel van zingeving…

**I: Ja, u noemde het zingeving inderdaad…**

R: Er zijn mensen waarin zingeving een belangrijke spirituele component bevat en er zijn mensen met nauwelijks een bewuste spirituele component binnen hun zingeving. En zingeving is alleen al van, wat heeft u plezier gegeven in uw leven en kunt u dat nu nog, en wat betekend het voor u dat het veranderd is, hé, dat is ook zingeving…

**I: Ja, ja. Brengt u die zelf ter sprake?**

R: Ja…

**I: Die aspecten met de patiënt?**

R: Ja.

**I: Oké.**

R: Het is gewoon een vast onderdeel van ons inventarisatieproces. Het kan zijn dat het totale proces te heftig is binnen een eerste gesprek, maar dan volgt het binnen een tweede gesprek. Het is zowel in ons MDO als in ons, het MDO doe je omdat je andere mee laat kijken of er, of er, wat je geïnventariseerd hebt, wat je behandelplan is, welk onderdeel van het behandelplan geaccepteerd is door, patiënten en hoe je dat dan gaat uitvoeren, of jij degene bent die het kan, of dat je hulpbronnen aansnijdt, bijvoorbeeld huisarts, bijvoorbeeld maatschappelijk werk, bijvoorbeeld psycholoog, geestelijk verzorger, en ja, een vast, persoon van de geestelijke verzorging, zit ook bij onze ochtendoverdracht en zit ook bij onze MDO’s. Dus die, als ik het niet inventariseer, wat is de reden dat dit niet aan de orde is gekomen.

**I: Oké. En dat MDO, is dat wekelijks overleg wat u heeft met alle…**

R: Ja…

**I: Partijen om de tafel…**

R: Ja, klopt, woensdagmiddag tussen half 1 en half 2, waar in principe alle mensen langskomen van de polikliniek, en alle mensen waar we, in medebehandeling zijn. Dus alleen de mensen waar we één of twee keer langs geweest zijn en niet meer binnen de kliniek, waar de hoofdbehandelaar, zeg maar de gene is die de verantwoordelijk heeft over het reilen en zeilen van de patiënt, houden we geen MDO. Mensen die desondanks erg interessant zijn kunnen we toch binnen het MDO bespreken, omdat we daarbij gewoon ook hulp vragen. En mensen die we drie keer of vaker gezien hebben worden ook altijd, de klinische patiënten, binnen het MDO gespard. En, het MDO van mensen die we poliklinisch zien, eehm, als we ze langer dan drie maanden onder controle houden, komt er een tweede MDO, zelfs een derde MDO. Dus, dat we, er zijn ook wel veel mensen die we veel langer zien dan.

**I: Ja, oké. Ja, ja.**

R: En, als, mijn verpleegkundige de zingeving, want dat doen ze allemaal wel, maar heel soms valt dat ertussen [25:00], dat ze mij niet geïnventariseerd hebben, dan vraag ik altijd, wat kun je daarover vertellen? En dat blijkt dat ze het wel geïnventariseerd hebben, maar dan zijn ze het vergeten op te schrijven of zo…

**I: Aah, op die manier.**

R: Dus, alleen, ja, een enkele keer, denkt een persoon niet. Maar ze hebben zelfs een vast stramien, waarin het, puntinventarisatie en dan krijg je ook de zingevingsdimensie die ingevuld moet worden…

**I: Die komt ook voorbij, oké.**

R: En dan is het wel eens niet geïnventariseerd, maar daar is ook altijd een reden voor.

**I: Ja, precies. Oké. En die verschillende domeinen, merkt u bij verschillende patiënten verschillen?**

R: Jazeker. Net als dat jij of, ik weet niet hoe jij in de gezondheidszorg zit, want hoe ben jij zelf in de gezondheidszorg verzeilt geraakt?

**I: Via dit project.**

R: Ja? En wat is jouw achtergrond?

**I: Communicatiewetenschap…**

R: Communicatiewetenschap. Goed. Als je iets over communicatie uitlegt aan mensen, dan zeg je, dit is het algemene, maar in individuele problemen kan het liggen aan gehoorstoornis, aan een uitspraakstoornis, het kan ook liggen omdat de ene partij de andere partij een enorm verschil heeft in basis. Het kan zijn dat het een combinatie is, dat iemand doof is en vanuit een verschillende basis spreekt, en zo zijn in communicatiestoornissen, zijn er natuurlijk heel veel mogelijkheden. En alles tegelijk kan een aanwezig zijn of er kan een deel aanwezig zijn, het hoofdprobleem kan doofheid zijn, het hoofdprobleem kan spreekproblemen zijn, enzovoorts. Of een taalprobleem. Zo hebben wij dat in de palliatieve zorg ook, het kan puur alleen nociceptieve pijn zijn, dat de patiënt en ik allebei ervan overtuigd zijn dat dat het probleem is en dat de afwijking lichamelijk ook zodanig is dat ie nociceptieve pijn geeft, maar dat is een unicum. Vaak speelt er van alles…

**I: Eerder een uitzondering dan…**

R: Ja. En, soms hebben we dat pijnen écht ontstaan, alleen door neuropathie, hé, dus door een zieke zenuw. Soms ontstaan pijnen echt omdat mensen verwachten pijn te hebben. Soms verdwijnen pijnen omdat er door suggestie, een mens, hebben laten overtuigen dat ie geen pijn heeft. En misschien ben je daar verwonderd over, maar als, als je in een nieuwe ruimte komt met een tikkende klok, dan hoor je ‘m. Als je daar een intensief gesprek hebt met een persoon, dan hoor je die klok niet meer tikken. Tot dat die persoon zegt, verhip, hij is onregelmatig. Wat? Ja, die klok. En dan hoor je ‘m onregelmatig tikken in ene keer. Dus het in en uitschakelen van het kennen van wat er met je zintuigen gebeurd, dat is echt op een ander niveau ook geregeld. Dat is ook met pijn zo. Soms maken we daar gebruik van, maar soms hebben we daar ook last van, als iemand ervan overtuigd is dat hij pijn heeft, fysieke pijn en wij vermoeden, als dat vermoeden steeds duidelijker wordt dat het niet zozeer in het fysieke vlak ligt, maar in een hele andere dimensie. Zoals ik het voorbeeld al gaf, van degene die hardloper is geweest, triatlon loper is geweest en eigenlijk moet zien dat dat helemaal afbreekt, dat hij dat wijdt aan zijn ziekte die ook pijn geeft en dan de combinatie maakt, als ik niet kan lopen, heb ik, is mijn ziekte progressief en heb ik dus pijn en naar de dokter communiceert, ik heb pijn. Maar in feite heeft hij zielenpijn. Hij heeft ook angst voor de toekomst, want niet lopen betekent fysiek onderuitgaan en dat betekent de dood en daar wil hij niet over praten. Dus, dat laat die complexiteit zien en daarom, je vraag was of het één ding is en altijd hetzelfde is, nee.

**I: Nee. Oké.**

R: Nou, en, als het uit je zingevingsdomein komt, ik ben die sterke man, ik ben die man die zoveel kon, ik ben die bijzondere man omdat ik winnaar ben [30:00] in zo’n intensieve sport, en dat jouw, belangrijke pijlers zijn van, van, van jouw bestaan, en het feit dat je dat niet kunt uitvoeren een ondermijning is van je bestaan en het begin van het einde aankondigt en je dat einde niet kan en wilt realiseren, of niet op geen enkele wijze kan inbouwen in je leven, dat er ook een einde aankomt, dan, dan ontstaat eigenlijk het probleem vooral uit je zingevingsdomein door heel duidelijke fysieke componenten. Kan een dokter dat oplossen? Nou, als hij in het fysieke domein blijft niet. Goed. Dus, zingevingsdomein, heeft natuurlijk een wisselende betekenis, ons vak is arts zijn, dat betekend dat je in principe heel goed opgeleid wordt in dat fysieke domein, wat minder goed in het psychosociale domein en heel matig in het zingevingsdomein. Maar je moet wezenlijk in al die domeinen kunnen opereren om een heel goed arts te zijn. En daar streven bijna alle artsen naar, dus we moeten dat, als we dat niet meegekregen hebben in ons geneeskunde onderwijs, dan moeten we dat ontwikkelen in de loop van ons leven.

**I: Oké. Ja.**

R: Is dit een antwoord op je vraag?

**I: Ja. Zeker weten. Ik wilde graag wat meer toespitsen op de communicatie. Zijn er patiënten waarbij u de communicatie moeilijker vindt?**

R: Mja. Er zijn heel veel aspecten. Op de eerste plaats, er is een, er is een asymmetrische relatie. Dat betekent dat ik, ik kan redelijk neutraal tegenover problemen staan. Maar de patiënt staat nooit neutraal tegenover die problemen, want die overkomt het. De patiënt kan een idee hebben waar dat door veroorzaakt wordt, of hoe het opgelost kan worden. En het kan zijn dat ik daar medisch een hele andere reden toe heb. Ik kan dat ontzettend goed gaan uitleggen aan de patiënt, ik ben nu de bevraagde, dus ik mag veel praten, maar ik realiseer me altijd, als ik meer praat als de patiënt, dat op dat moment de communicatie al aan het foutgaan is. Want, eigenlijk, ben ik er voor de patiënt. En als ik veel moet praten, dan ben ik aan het zwoegen. En als ik aan het zwoegen ben, dan verloopt de communicatie niet lekker. En als ik niet aan het zwoegen ben en de patiënt veel meer in de lead is van ons gesprek, kan zijn, dan is het dus geen probleem. Dus, ik ben me heel goed bewust dat, rondom communicatie, het weer, de vraag is eenvoudig, maar ligt het veel lastiger. Het kan zijn dat de communicatie op zich niet optimaal verloopt, omdat de patiënt doof is. Zo hebben we een patiënt die, als ik rustig en zwaar spreek, mij verstaat. Onze, fellow, met een hoge vrouwelijke stem, wordt nooit door die patiënt gehoord. Het valt helemaal binnen zijn, hij is niet doof, maar haar hoort hij niet. Dus hij kan niet communiceren. Tenminste, niet op een normale manier. Het kan zijn dat ik een zakelijke uitleg geef, waarbij ik, medische termen gebruik die ver over het hoofd van de patiënt gaan. Het kan ook zijn dat ik de, met een directeur te maken heb, van een, en dan ga je ervan uit dat het een man is met allerlei achtergronden, maar die, medisch gezien echt niet weet waar zijn lever zit, hé [35:00], dat geldt ook voor allerlei andere medische aspecten. Dus hij is een organisatiedeskundige, maar begrijpt niet goed hoe die medische organisatie in elkaar zit. Dan kan het zijn dat ik ervan uit gegaan ben dat hij het wel begreep, maar dat ik op een veel basale manier, misschien, met hem had moeten spreken, ook al heeft hij de universiteit achter de rug. Het feit dat je voldoende leercapaciteit hebt en voldoende, leerachtergrond, misschien zelfs een geweldige organisatieachtergrond, kan het toch zijn dat je van het huidige probleem het gewoon niet begrijpt. Dan heb je mensen die zijn altijd in een distress, of fysiek, of we hadden pas een patiënt waarvan onze geestelijke verzorger zei, het is heel duidelijk dat hij in de zingevingproblematiek hierin vastzit, ik zeg ja, dat is ook zo, maar ik kan er niet met hem over praten. Waarom niet, zegt ze. Ik zeg gewoon, omdat die man zoveel pijn heeft, dat ieder gesprek bijna onmogelijk is. Ik moet eerst zorgen dat hij van die vreselijke distress af is, dat hij gewoon ligt te draaien en kronkelen van de pijn, en dan ontstaat er ruimte om het zingevingsdomein beter op te pakken. Datzelfde geldt voor emoties, datzelfde geldt voor sociale problematiek. Als ik alleen maar kan denken aan goh, als ik, niet meer terugkom op mijn werk, dan kan ik mijn hypotheek niet betalen en dan wordt het huis onder m’n gat uit verkocht, en dan heeft mijn vrouw, en ik ben ziek, en ik ga dood, maar dan heeft mijn vrouw ook nog eens het huis verloren, ik ben maar in één ding geïnteresseerd; dokter, of het pijn doet of niet, hoe kunt u me oplappen dat ik weer aan het werk ga. Dus, de doelen enzovoorts, die zijn van belang, de drempels die je moet nemen, die zijn van belang, de fysieke problemen, zeg maar het idee van, binnen de asymmetrische collatie, ik kan wel weten hoe het zit, ik kan wel denken te weten hoe het moet, maar de patiënt moet hierin meegenomen worden. Niet iedere patiënt, ook al heeft hij een hoog cognitief vermogen, kan het op het niveau van onze beraadslagingen begrijpen, pas dat aan. Dat zijn allemaal aspecten die in de communicatie, tot problemen kunnen leiden. En dan tenslotte, vertrouwt de patiënt mij? Heeft hij of zij, bij het eerste contact, waarin iedereen open ingaat, krijgt hij zoveel elementen te voelen en te horen dat hij zegt, Ja, het is een deskundig persoon en een empathisch persoon, daar wil ik wel mee verder. En soms komen mensen binnen die zijn zo geschaad door eerdere ervaringen of problemen, dat ze denken, nou, ik vertrouw er geen bal van, maar vertel het maar eens. Hé, ik bedoel, dan begin je al met een handicap.

**I: Ja. Maar komt u dan wel binnen bij die mensen?**

R: Nou, soms niet.

**I: Oké, want dan vraag ik me vervolgens af, want ik probeer dat dan voor me te zien, u omschrijft situaties die in communicatie de symmetrie kunnen verstoren, maar mijn volgende vraag zou dan zijn, hoe lost u dat dan op?**

R: Ja, dat is…

**I: Bijvoorbeeld vertrouwen, wat u zegt…**

R: Ja, nou, misschien nog door op dit soort dingen te letten. Heeft de patiënt zijn verhaal eerst kunnen doen? Ben ik begonnen met mijn eigen agenda? Of ben ik begonnen met de agenda van de patiënt? Heb ik me wel daaraan aangepast? Besef ik of deze persoon de juiste cognities heeft, de juiste zintuigen heeft om in communicatie mee te doen, welke drempels zijn er bij deze patiënt, ik kan wel bedenken dat hij meer of minder cognitieve functies heeft, maar heeft hij ook de achtergronden om de medische redeneerwijze [40:00] te kunnen begrijpen en hoe moet ik het aanpassen dat hij het wel kan begrijpen…

**I: En in het voorbeeld van de directeur wat u hiervoor aangaf?**

R: Ja, dus in feite is het een directeur met een universitaire achtergrond, maar medisch heeft hij er geen grip op, en nooit ziek geweest. Dus geen enkele ervaring met ziek zijn.

**I: Ja.**

R: En een beetje een autoritair figuur, omdat dat nu eenmaal moest binnen zijn vak. Dus, eerder de rode karaktervorm, de rode communicatievorm. Ik wil, dit moet. Wat ik ook een beetje besef van mijzelf, hoe ik altijd op dit soort dingen reageer, dat ik vooral niet primair moet reageren.

**I: Oké.**

R: Soms wel.

**I: Oké, want u, u bent zich zeer bewust van hoe u zelf communiceert, dat hoor ik steeds, maar er zijn denk ik, weet ik niet hoor, maar ik vermoed dat niet iedere zorgverlener dat in zich heeft.**

R: Nee, net als ieder mens.

**I: Ja, precies.**

R: Anders heb je geen communicatiedeskundigen.

**I: Precies, maar ik vraag mij dus af, hoe zouden mensen die dat wat minder hebben, die ook in dezelfde, op hetzelfde gebied werkzaam zijn als u, hoe zouden die dat kunnen leren en kunnen die dat leren?**

R: Ja, dat denk ik wel.

**I: Oké.**

R: Ja. Eigenlijk, kijk, je moet er ook vanuit gaan, ik maak maar héél soms mee dat er een arts is die niet het beste voor zijn patiënten wil. Ik maak het toch vaker mee dat een arts de plank misslaat. Ik maak het ook mee dat artsen en verpleegkundigen de plank misslaan en volharden in hun idee op basis van eigen beperkingen enzovoorts. Maar, de basis is dat men het beste wil voor de patiënt. En als je daarvan uit gaat, kun je ook, ten eerste is dat de basis waar ik zelf persoonlijk vanuit ga, dus ook altijd openstaan voor anderen die zeggen, jij denkt dit wel, maar ik ervaar hier dit en dat mee, nou, kom op, vertel me, dan kan ik in ieder geval verbeteren, of we kunnen er samen uitkomen hoe jij en ik dit denken. Nou, heeft iemand een bepaalde karakterstructuur, ja, dan moet je daar een beetje als buigend riet mee omgaan. Dus dan moet je zoeken van, wat zou bij hem de ingang zijn, of bij haar. En net zoals je rode en groene en blauwe figuren hebt binnen patiënten, heb je hetzelfde net artsen.

**I: Ja, precies. Ja. Overigens triggerde u mij ook met de vraag, ik moet nooit primair reageren, maar soms wel.**

R: Ja.

**I: Ik dacht, oké, maar hoe ontstaat dat, vanuit een gevoel, of vanuit goed luisteren?**

R: Ja, goed luisteren en steeds bewust zijn van, wat gebeurt hier. Dat doe je niet in het begin, maar leer je gaandeweg. Als er nu een patiënt bij me komt, schat ik in welke vorm van communicatie die persoon wat aan heeft. En, ik heb een patiënt waar ik echt gewoon heel direct zeg van joh, dit kun je niet maken. En, ik heb wel medicijnen voor u, maar a, weet ik niet wat je nodig hebt, maar b, ik heb wel nodig dat je doet wat ik zeg. Nou, dat is ook echt een figuur die dat nodig heeft.

**I: Ja, precies.**

R: Maar zou ik dit bij een ander doen, dan zou je werkelijk de ellende over de hals heen halen. Nou daar ga ik niet vaker heen, dat is een boer van een kerel. Terwijl bij deze eerste patiënt, hé, waarbij ik dit zo doe, omdat die helemaal niet met die grijstinten om kan gaan. Die heeft een handvat nodig en die heeft een duidelijke uitspraak nodig. Dus, daar communiceer je anders mee.

**I: Ja. Oké. Ik had ook nog een vraag over wat u aangaf, en verbeter mij als ik het verkeerd zeg, u gaf aan, als ik merk dat ik aan het woord ben, dan ben ik aan het zwoegen en u gaf aan, ik ben er voor de patiënt en de patiënt moet dus [45:00] degene zijn die het meeste aan het woord is. Ik zoek nu naar een koppeling met gezamenlijke besluitvorming. Zoals ik het zelf heb geleerd, is dat gezamenlijke besluitvorming, dan zou die precies gelijk of evenwijdig kunnen zijn. U schudt al nee, dus ik ben heel benieuwd. Of niet, dan zou ook heel anders kunnen liggen, maar ik ben in ieder geval benieuwd wat u vindt van gezamenlijke besluitvorming van patiënten in de palliatieve fase.**

R: Niet anders dan bij mensen buiten de palliatieve fase, zij het dat communicatie op allerlei manieren verstoord zou kunnen worden, in de palliatieve fase kan dat op nog meer manieren als buiten de palliatieve fase. En als je als techneut weet hoe het moet, een arts is gewoon een techneut, dan heb je altijd een enorme voorsprong, maar je hebt een enorme achterstand in communicatie. Nou, je moet maar eens bedenken dat je naar de, fietsenmaker gaat en dat die fietsenmaker tegen je zegt, goh, wat erg, jouw fiets rijdt niet meer soepel. Wat doet dat met je? Sjeeeee. Ja, en wat verwacht je eigenlijk van de toekomst? Ik denk dat je het risico loopt dat je onmiddellijk omkeert en denkt, hier moet ik niet zijn…

**I: Ja, ik ga wel iets verder op de hoek.**

R: Ja. Een fietsenmaker die zegt tegen je, tja, je hebt pech. Je achter-lager lijkt stuk te zijn, ik maak ‘m wel open en kijk wel even, maar goede kans dat ie stuk is. En dan kan ik er een nieuwe inzetten en dat kost wat werk, en die lagers, die, daar zit ook nog een derailleur op, die moet ik ook nog demonteren, dat gaat je €150 euro kosten. En als ik naar die fiets kijk, is het eigenlijk een barrel, vind je dat het nog wel waard? Dan denk je, nou, goeie fietsenmaker. Hij heeft door wat er aan de hand is en hij is eerlijk over…

**I: Hier kan ik wat mee, denk je dan...**

R: Ja. Maar wel autoritair, alleen denk je daar niet aan, dat hij autoritair is. En daarmee heb je jezelf eigenlijk helemaal overgeleverd aan die fietsenmaker die gewoon van wanten weet. Maar als die fietsenmaker eigenlijk naar jouw fiets gekeken heeft, en dacht, verhip, het is een museummodel en die past goed in mijn verzameling en ik bied ‘m aan dat ie ook een wat goedkoper tweedehandsje kan krijgen van wat ik nu aan reparatiekosten opgeef, dan heb ik dat museumstuk bijna cadeau. Jij bent ingegaan op dat blinkende model wat er stond, wat nu gereduceerd van €150, €100 euro kost, totdat je hoort dat het fietsenmuseum op de Waalkade die prachtige Franse merkfiets uit het verleden verworven heeft voor €1500 euro…

**I: Van de fietsenmaker…**

R: En je ziet je eigen barrel terug. Dus, die asymmetrie, die is er natuurlijk. Dat betekent ook, dat zelfs met uitleg, zelfs met bespreken van zaken, weet je als arts meer van die achterkant dan dat je, je kunt een patiënt niet de gehele medische wetenschap aanleren. Dus je geeft ‘m een clou, maar met die clou zal het altijd zijn dat ie minder kennis heeft als jou. En dat betekent dat hij besluiten gaat nemen die toch gericht zijn naar de voorbesluiten die de arts genomen heeft. En dat bedoel ik met die asymmetrische relatie. Dus ben je er consequent en steeds weer van bewust dat informed consent flauwekul [50:00] is, dat het in het leven geroepen is door managers. Dat het in leven geroepen is door, goedwillende profeten en gepakt is door verzekeraars en journalistiek en politiek en die zegt van ooh, informed consent, natuurlijk, patiënt centraal, prachtig, en in werkelijkheid staat de patiënt nooit centraal, want je laat de patiënt naar jou toekomen in plaats van dat je naar de patiënt toegaat. Dat betekent al dat het asymmetrisch is, want als ik bij de patiënt thuiskom, gedraag ik me heel anders als dat de patiënt bij mij komt. We hadden laatst ook een voorbeeld dat een van onze assistenten, die bij de patiënt thuis is geweest zegt, goh, dat is toch wel een ervaring. Hoe anders dat dan gaat. Dus, asymmetrie in allerlei vormen, dat informed consent, heeft ook aspecten die, waarbij er nooit naar het midden gezocht wordt. Er wordt wel gepoogd dat goed te doen, maar ligt ver van de waarheid af, dat het daar ligt. Wat ligt wel, het oprechte zoeken van een team, een arts, om de patiënt zodanig te informeren dat hij de centrale elementen begrijpt, maar niet verward wordt door randelementen die ook spelen. Dat het op zo’n manier gebracht wordt het ook binnen zijn kenniskaders valt, bewust van de drempels die een patiënt heeft om dingen op dit moment te begrijpen en dat betekend ook dat eigenlijk, besluitvorming, altijd, vanuit het team eigenlijk de richting al aangegeven heeft, waarin medische kennis en kunde en verpleegkundige kennis en kunde het meest waarschijnlijke het beste is. En dan, rekening houdend met dat patiënten ook echt bepaalde dingen niet zou willen. Ik kan wel zeggen, die operatie kan het beste plaatsvinden terwijl er zes zakken bloed klaarstaan, maar als ik Jehova’s getuigen ben, wil ik dat bloed niet. En het risico van doodgaan, na de operatie, is mij veel liever, dan het bloed krijgen. Allerlei vormen, het is niet alleen dat er asymmetrie is, dat er begrips-asymmetrie is, er zijn kennishiaten, het is ook drempels, het is ook mijn cultuur- en zingevingsverschil, wat maakt dat het midden zoeken er niet is, maar dat wel de best mogelijke vorm gekozen wordt waarbij je steeds bewust moet zijn van die asymmetrie.

**I: Oké. Ja. Interessant. Hoeveel vragen mag ik u nog stellen?**

R: De assistent komt pas terug om 13:00 uur.

**I: Oké. Mag ik u dan nog een aantal vragen stellen over beperkte gezondheidsvaardigheden? Heeft u wel eens gehoord van dat begrip?**

R: Jazeker.

**I: Wat is volgens u, wat betekent dat volgens u?**

R: Ja, beperkte gezondheidsvaardigheden, ze kunnen acuut, laat ik het zo zeggen, iemand met een EMV van 6, die heeft zeer veel storingen daarin en praat eigenlijk alleen maar met familie. EMV 6 betekent eigenlijk dat je in een diepe coma bent.

**I: Oké, ik wilde al vragen wat dat betekende.**

R: Ja, in een diepe coma waarbij geen enkele prikkeling nog ontvangen wordt. Maar je hebt, en dat kan acuut ontstaan zijn hé, je hebt je hersenbloeding gekregen en je bent weggezakt in een coma, dan heb je absoluut gezondheidsvaardigheden verlies. Het kan ook zijn dat je het in een bepaald, dat je heel veel weet van het menselijk lichaam en van de organisatie, maar dat je emotioneel overweldigd bent over wat er aan de hand is en dat je gewoon verkeerde keuzes maakt omdat je op dat moment, alleen nog maar de angst hebt een kind te verliezen, of wat dan ook. Het kan zijn dat je op allerlei manieren gezondheidsvaardigheden hebt, maar dat je per se het model [55:00] van, het moet biologisch zijn, het mag niet uit een chemisch potje komen…

I: Oh, ja op die manier.

R: Ja, dan heb je een beperking in je gezondheidsvaardigheden. Je kunt gewoon niet denken over andere aspecten. Omgekeerd kan ik als dokter ook gezondheidsvaardigheden missen omdat het nu eenmaal mijn vakgebied niet is en denk dat ik er toch over kan praten. Maar goed, dat is een ander gezondheidsvaardigheidsverlies. Het kan zijn dat je, cognitieve functies mist, en bij een coma is dat natuurlijk heel diep, maar het kan ook zijn dat je chronisch cognitieve beperkingen hebt, of bij geboorte meegekregen, of verworven, of nu door je ziekte verworven, maar het kan ook zijn dat je uitstekende cognitieve functies hebt, maar gewoon het begrippenkader hiervan mist. Waar we het al even over gehad hebben, de directeur van puntje puntje, enzovoorts, die kan toch onder gezondheidszorg eigenlijk heel beperkte informatie hebben en op het moment dat hij niet vooruit kan vanwege de pijn, ja, die dokter, die moet die pijn wegnemen, en dan kan ik weer verder. En hoe, dat maakt niet uit. Maar het moet wel nu. Goed. Dus die gezondheidsvaardigheden, die kan op allerlei manieren.

**I: Ja, oké. Ik heb al een aantal voorbeelden van u gehoord van mensen die u zelf tegen bent gekomen met beperkte gezondheidsvaardigheden, en ook hoe u die patiënten herkent en hoe u daarmee omgaat. Ervaart u barrières in de zorg die u die mensen wilt geven?**

R: Ja, het lijkt nu wel alsof je denkt dat het een kleine groep is, in feite is…

**I: Nee, dat weet ik helemaal niet. Is dat een grote groep?**

R: Ja, een héél grote groep. Op het moment dat je, met je vriend, of met je kind, of met je ouders binnenkomt op die eerste hulp, heb je beperkingen in je gezondheidsvaardigheden. Alleen al omdat je niet weet hoe het hoort op de 1^e^ hulp. Je loopt waarschijnlijk naar de balie, maar dan zeggen ze, ga daar maar zitten. Maar je denkt, tja. Het feit dat je zou moeten zeggen, ja, dat kan niet, want de persoon die, ik ben vooruitgelopen, maar die persoon, die heeft zo’n ademgebrek, ik kan ‘m zelfs niet uit de auto krijgen. Ja, als het zo erg is zal je dat wel zo zeggen. Maar dat je aan moet geven, zou moeten geven op een schaal van zus of zo zodat het sneller of minder snel en zus en zo, misschien roep je wel heel veel aandacht af terwijl het niet nodig is en misschien vraag je te weinig aandacht terwijl wel snelheid nodig is.

**I: Ja.**

R: En, weet jij dat?

**I: Of ik dat zou weten? Als ik op een eerste hulp komt?**

R: Bijvoorbeeld met je kind, of met je ouder?

**I: Dat weet ik op dit moment niet, ik denk het niet…**

R: Nee, dus dan heb je beperkingen in je gezondheidsvaardigheden. Is het dan niet zo dat misschien wel heel veel mensen beperkingen hebben in hun gezondheidsvaardigheden?

**I: Ja, vanuit die definitie heeft in principe iedereen die het ziekenhuis instapt beperkte** gezondheidsvaardigheden.

R: Bingo. Toen ik naar beneden gedonderd was en mijn heup gebroken had…

**I: Oh echt?**

R: Ja, daarom loop ik met een stok. Sprak ik met de chirurg en we gingen het een en ander doen, allemaal bekend en het leek me keurig, enzovoorts. En mijn echtgenote zei, ja, ik zat naast je en ik dacht ja, je begrijpt het allemaal. Maar achteraf had je beter die en die vraag kunnen stellen en realiseer ik me, via mijn vrouw zeggende, realiseer ik me. Dus met alle kennis en kunde, werkzaam in hetzelfde vak, kon ik toch als patiënt niet alle goede vragen stellen. Dus ik was beperkt in mijn gezondheidsvaardigheden. Dat jij me de vraag stelt, doet dus vermoeden dat je denkt dat er mensen zijn zonder beperkingen in gezondheidsvaardigheden.

**I: Nou, dat weet ik niet.**

R: Nee, maar ik denk dus niemand.

**I: Ja, precies. Ja [1:00:00]. Ja, want er is niemand die alles kan weten natuurlijk.**

R: Nee, niet alle compilaties en zeker als je met pijn of stress of wat dan ook, dan kun je gewoon niet overal aan denken. En dat betekend dus dat je altijd te maken hebt met beperkingen in je gezondheidsvaardigheden. En dat het een meer opvalt als het andere en dat is dus een heel gradueel verschil. En dat betekent meteen dat je vragen hierna, of het met IQ samenhangt, of met opleiding samenhangt, of met biologische kennis samenhangt, of met medische kennis samenhangt, kan ik op alles ja zeggen. Maar hangt het ook samen met pijn, met shock, met emotionele shock, met, angst voor de andere, het speelt allemaal, het heeft allemaal met elkaar te maken.

**I: Ja, nou mijn volgende vraag zou eigenlijk zijn wat volgens u nodig zou zijn om die beperkte gezondheidsvaardigheden, die iedereen heeft, wat volgens u nodig zou zijn om daar toch beter aansluiting bij te kunnen vinden.**

R: Ja, en ik denk dat veel mensen dat, onbewust doen en onbewust zijn van hun beperkingen daarin. En, sommige mensen zelfs, doen het bewust maar zijn ook onbewust van hun beperkingen. Sommige mensen zijn bewust en zijn zich ook bewust van hun beperkingen. En sommige mensen hebben geleerd om over hun beperkingen heen te stappen. En, passen alle kennis en kunde bewust toe. Sommige mensen zijn hier vergeten dat ze dat traject doorlopen hebben en voeren onbewust alle noodzakelijke handelingen uit die over die beperkingen heenstappen. Dat betekent ook dat een systeem om hieraan te werken eerst eens boven water mag halen waar de hulpverlener zelf zit in dat hele systeem.

**I: Oké, dus als ik u goed begrijp is het een soort van bewustwording van de zorgverlener zelf in eerste instantie?**

R: Ja, eigenlijk een inventarisatie waar, waar zijn hulpverleners zich wel bewust van en niet bewust van en, is er een groep hulpverleners die zich, meestal onbewust zijn van dit soort problemen en moeten we daar niet eerst eens zich bewust laten worden dat deze problemen er zijn en letterlijk zou de vraag moeten zijn, zijn er mensen met, beperkingen in gezondheidsvaardigheden, en als niet het antwoord wordt, ja, geen mens is onbeperkt in zijn gezondheidsvaardigheden, iedereen heeft wel beperkingen op het moment dat het speelt, als die vraag anders beantwoord wordt, dan is de gezondheidswerker in ieder geval voor een deel niet bewust van de beperkingen.

**I: Ja. Dus dat zou een goede test zijn om te kijken hoe de zorgverlener er zelf voorstaat.**

R: Ja. En dan heb je laaghangend fruit natuurlijk, dat als je mensen hebt met een lage cognitie, of een laag sociaal opleidingsniveau, dat je, wat eerder beperkingen hebt in het begrijpen van alle aspecten van de gezondheidszorg en van datgene dat de dokter vraagt of zegt. Maar problemen, rondom gezondheidsbeperkingen, die vinden net zo goed bij de directeur plaats, omdat hij toevallig van dat gebied niets wist en vinden net zo goed bij de dokter zelf plaats omdat hij op dat moment pijn heeft, dus het, het is eigenlijk door alles heen…

**I: Maar die patiënten die u net noemde, met die lagere vaardigheden, hoe gaat u daar zelf mee om? Hoe werkt dat?**

R: Niet anders als bij de directeur. Gewoon categoriseren waar, waar zouden de potentiele problemen liggen. Hoe moet ik mijn communicatie voeren om in ieder geval, of laat ik het zeggen, hoe moet ik precies inventariseren [1:05:00], kan ik het beperkt inventariseren, of betekend het een verandering van communicatie van mij, betekend het dat ik, andere hulpverleners moet inschakelen om dit te laten begeleiden, of kan ik dit beter zelf doen, moet ik ‘m hulpmiddelen geven om ondanks de beperkingen in ieder geval handvatten zijn die voor hem duidelijk maken en het kan gaan om heel eenvoudige dingen, bijvoorbeeld, als ik een behandeling aan iemand geef als oncoloog, of als palliator, dan kan die behandeling gepaard gaan met bijwerkingen, die bijwerkingen probeer ik meestal te vertellen. Mensen met heel veel beperkingen van hun gezondheidsvaardigheden zeg ik gewoon, u krijgt nu de pijnstiller, ik ga u morgen bellen hoe het gegaan is en of u er last van had. Maar als u dit of dat krijgt, belt u mij onmiddellijk op. En meer lig ik niet uit, want dat is het maximum van wat iemand hebben kan. Als ik ‘m ga uitleggen, je kunt dit krijgen, je kunt dat krijgen, zus en zo, en dit en dat, dan ben ik ‘m allang kwijt. Maar ook bij die directeur, kan het zijn dat ik zo kort door de bocht kan gaan, omdat ik merk dat hij door omstandigheden echt niet meer hebben kan. En dan kan ik ook heel kort zijn. We gaan nu dit doen en ik hoop dat daarmee de pijn verbeterd. Bij de meeste mensen lukt het, dus ik verwacht het wel, maar morgen bel ik je en dan heb ik ‘m ook niets uitgelegd. Bewust dat het geen beperking is, maar dat het een absolute barrière is.

**I: Oké, ja. Want u gaf ook aan, dat is dus de wijze waarop u uw communicatie eigenlijk aanpast.**

R: Ja.

**I: En het moment, de inschatting die u maakt, die staat eigenlijk aan de grondslag van hoe u vervolgens met uw patiënten communiceert, als ik het goed begrijp?**

R: Ja. En niet alleen communiceert, maar ook toetsend, iedere keer weer, klopt het wel wat ik denk? Het is ongelofelijk, je denkt bij die eerste woorden, dit is iemand waarbij ik iets eenvoudiger moet zijn en die geeft zoveel reflectie dat ik onmiddellijk doorga naar een ander niveau.

**I: Oh, dus u stapt eigenlijk redelijk laag in en dan ziet u op een gegeven moment hoe laat het is, als u begrijpt wat ik bedoel, en dat ziet u oh…**

R: Ja, zeker pas ik me aan. En omgekeerd ook.

**I: Ja, oké. Ja, interessant.**

R: Alleen niet handig om een systeem te bouwen natuurlijk…

**I: Nee, dat is absoluut waar…**

R: Nee, maar daarom zeg ik ook, laaghangend fruit, er is natuurlijk laaghangen fruit, ik ben bijna zeker dat iedereen dat noemt, dat laaghangend fruit. En dat zal ook echt zo zijn, en dat zal ook echt, als je in de gezondheidszorg met zeg maar die, die, verborgen, maar toch bekende gezondheidszorgproblemen en verborgen is, er zijn zoveel mensen met leesbeperkingen, met schrijfbeperkingen, met rekenbeperkingen, dat zijn allemaal mensen die écht beperkingen hebben en door bepaalde tools, heel gemakkelijk door alle beperkingen heen geleidt kunnen worden. Maar je moet je bewust zijn dat die mensen er zijn en dat je aan een actief opsporingsbeleid doet. Want, ja, hoeveel mensen hebben problemen met lezen en schrijven? Volgens mij 10, 20 procent, 10 procent zeker, en dat mensen het absoluut niet kunnen dat is lager, dus als iemand wat opschrijft, wil helemaal niet zeggen dat ie lees- of schrijfvaardigheden heeft van voldoende niveau. Het kan zijn dat als hij een briefje krijgt wat vier regels dik is, dat het voor hem niet meer te volgen is.

**I: Ja. Ja, maar dat klopt inderdaad, dat laaghangend fruit en dat systeem dat gebouwd wordt, daarom vind ik het ook interessant dat ik het kwalitatieve deel mag doen en ook aan zorgverleners mag vragen, van nou, oké, maar vertel dan maar eens hoe u het doet.**

R: Ja, precies.

**I: Dus dan staat u voor de patiënt, wat doet u dan, wat gebeurt er dan [1:10:00], dus dat, dat vind ik er heel interessant aan, maar dat is mijn persoonlijke…**

R: Zeker, zeker, ja. Goed, heb je alle vragen kunnen stellen die je ook wilde stellen?

**I: Ja.**

R: Goed.

**I: Heeft u nog iets toe te voegen?**

R: Nee. Heb je nieuwe dingen geleerd?

**I: Ja, ja, zeker.**

R: Goed.

Z4d05

I: interviewer

R: respondent

**00.37**

**I: Wat is jouw rol in de zorg voor patiënten in de palliatieve fase?**

R: Ik ben oncoloog en als oncoloog werk ik in het palliatieve team. Wat wij doen en wat mijn taak in het team is, is eigenlijk vooral meedenken in alle scenario’s die kunnen ontstaan, het team ook extra uitleg geven over mogelijke complicaties en te verwachten scenario’s bij dat specifieke ziektebeeld. Dat is mijn rol in het team. Maar ook natuurlijk gewoon samen patiënten zien, samen met de verpleegkundigen. Ja, de rol; die bestaat eigenlijk wel uit meerdere dingen. Het is aan de ene kant arts zijn, maar aan de andere kant onderwijs geven in allerlei verschillende blokken en aan de andere kant ook bezig zijn met onderzoek dat hier loopt. Ik weet niet precies welke rol je specifiek zou willen weten.

**I: Nou, voor het interview is de rol van jou als arts denk ik wel het meest interessant.**

R: Ja. Wat wij doen in onze consultteams is eigenlijk; we worden gevraagd door de afdeling meestal om in consult te komen, om mee te denken waarmee wij patiënten zouden kunnen helpen. Die consultvraag is meestal een hele eendimensionale vraag. De patiënt heeft heel veel pijn, het gaat niet. Als je met iemand gaat praten is er veel meer te ontleden dan alleen het somatische begrip pijn. Dan zitten de andere dimensies er veel meer onder, maar die zijn nog niet herkend door de patiënt, door de familie of door de verpleging of door de dokter, of die zijn nog niet zoveel aan de orde gekomen. Ik denk dat je als palliatief team wat meer tijd en ruimte en ook minder reserves hebt om al die dingen gewoon te bespreken en dat we binnen onze taak in het palliatieve team als dokter ook heel veel onderwijs geven, ad hoc, aan de verpleegkundigen en aan de dokters van de afdelingen waar we komen.

**I: Dus echt op het moment dat je daar bent eigenlijk.**

R: Ja. En daarbij geven we ook wel op vaste tijden onderwijs aan afdelingen enzovoort, maar binnen onze klinische taak van het consult is het heel veel van: er komt een patiënt, er is een vraag. Op de afdeling Oncologie komen we sowieso elke dag langs om ook wat eerder in het traject mee te denken. Dat hangt een beetje van de afdeling af, waar we meer of minder komen.

**I: Ja, precies. Dus als ik jou goed begrijp en verbeter me als ik het verkeerd zeg: jullie hebben eigenlijk een meer adviserende rol en jullie worden bij consulten betrokken als er heel veel pijn is. Ik zeg het even heel simpel hè?**

R: Ja, maar pijn is heel vaak een ingangsprobleem, maar het is ook vaak: de patiënt heeft slecht nieuws gehad, graag meedenken over hoe de zorg te organiseren is. Of: de patiënt is heel erg misselijk. Of: de patiënt is heel erg angstig. Of: er is een indicatie voor palliatieve sedatie. Hier in het ziekenhuis is bij elke patiënt bij wie overwogen wordt om palliatieve sedatie te starten het beleid om het palliatieve team in consult te vragen, om mee te denken of de indicatie van palliatieve sedatie juist is. Heel af en toe zijn wij hoofdbehandelaar van een patiënt, maar wij zijn denk ik in vijfennegentig procent van de gevallen consulent.

**I: Mijn volgende vraag zou zijn of er altijd een centrale zorgverlener aangewezen is bij patiënten. In de meeste gevallen zijn jullie niet de centrale zorgverlener...**

R: Nee. Dat proberen we ook zo te doen, het ook heel concreet te maken. In principe schrijven we geen medicatie voor, maar we geven wel het advies welke medicatie voorgeschreven zou kunnen worden. Eigenlijk wordt dat in negenennegentig van de honderd keer ook direct opgevolgd. Zij vragen ons niet voor niks en hebben van bepaalde dingen niet zoveel verstand. En soms helpen we weleens mee om bijvoorbeeld… pompen en dergelijke, dat is soms wat lastig voor iemand om voor te schrijven, zeker als mensen naar huis gaan, om de juiste order in het juiste systeem te krijgen. Onze verpleegkundig specialisten schrijven dan die specifieke orders voor, omdat dat nou eenmaal wat minder ingewikkeld is voor onze verpleegkundig specialisten dan voor de zaalarts daar. Maar in principe geven wij een advies, dat koppelen we terug aan de arts en die bepaalt of hij of zij wel dat advies uitvoert, ja of nee.

**05.26**

**I: Dus dat geldt binnen het ziekenhuis. Maar werken jullie bijvoorbeeld ook samen met huisartsen?**

R: Nou, binnen ons team werkt sowieso al een huisarts en die is ook altijd de voorzitter van ons MDO. Dat is bij uitstek ook degene die heel erg zorgt voor wat meer het beleidsniveau voor de transmuraliteit. Daarnaast hebben wij voor huisartsen en andere specialisten een vierentwintig uur bereikbaarheidsnummer voor patiënten die we kennen. Dus als huisartsen een vraag hebben bij een patiënt die bij ons gezien is, kunnen huisartsen vierentwintig uur per dag met iemand van ons direct overleggen. Tijdens kantooruren krijgen ze dan vaak eerst de secretaresse of de verpleegkundige, maar buiten kantooruren krijgen ze altijd direct een dokter aan de telefoon. Dus in zoverre hebben we ook die samenwerking. Huisartsen kunnen ook rechtstreeks een patiënt naar het palliatieve team verwijzen, voor de poli. Dus als een huisarts zegt van, goh, ik loop vast, of, ik wil hierover nadenken of over sparren, wat zou ik nog meer kunnen doen, dan zou dat een reden kunnen zijn om patiënten eenmalig bij ons te laten zien, zodat wij in ieder geval met een huisarts mee kunnen denken over hoe verder in het palliatieve traject. Ja. Dus in die zin werken we in de praktijk samen. Ja.

**I: En bijvoorbeeld met maatschappelijk werk of andere zorgverleners?**

R: Ja. Binnen ons team werkt ook een geestelijk verzorger, maar eigenlijk is dat binnen ons team vooral een spiritueel consulent.

**I: Dus iets breder.**

R: Ja. Zij zit in ons team voor een aantal vaste uren. Daarnaast werken we ook met een psycholoog. Maar dat zijn meer mensen, die zitten bijvoorbeeld wel bij ons MDO altijd standaard, maar die zitten niet dagelijks in ons team waarmee we de kliniekdienst draaien. De dagelijkse kliniekdienst wordt gedraaid door een dokter en een verpleegkundige met het aandachtsgebied palliatieve zorg, soms een verpleegkundig specialist of een gewone verpleegkundige met dat aandachtsgebied, en bijna altijd een stagiaire, iemand die in opleiding is tot oncoloog die een stage doet palliatieve zorg of een specialist in opleiding tot algemeen internist. Bijna altijd is er iemand die stageloopt. Elke dag van de week is er een andere dokter die de hoofddienst heeft. Ja. En al die andere mensen, zoals de spiritueel consulent of maatschappelijk werk of de psycholoog, kunnen wij wel zelf in consult vragen waar nodig.

**I: Ja, precies. Die worden er eigenlijk bij gevraagd als het…**

R: Ja. Dat heeft er ook mee te maken; de afdeling Oncologie heeft bijvoorbeeld eigen mensen met wie zij samenwerken. Dus als wij een consult doen op de afdeling Oncologie is het heel handig dat er een maatschappelijk werker is van de afdeling Oncologie die daar in consult komt, omdat het juist diegene is die bij uitstek weet hoe de problemen zitten voor de oncologiepatiënt. Bij hartfalen is dat net weer anders, dan heb je daar weer een maatschappelijk werker. Maar dat is meer hoe het ziekenhuis georganiseerd is. Ja.

**08.47**

**I: Wat mij betreft is de rol heel duidelijk. Dan wilde ik wat gaan vragen over communiceren, informeren en gezamenlijke besluitvorming. Ik wilde inhaken op iets wat je zei. Je zei, je hebt dus het somatische aspect, maar je hebt ook nog meer aspecten. Dus je hebt het fysieke, maar ook het psychische, sociale en spirituele welzijn. In hoeverre komen die aspecten aan bod in jouw gesprekken met de patiënt?**

R: Eigenlijk altijd. Althans, dat is wel de bedoeling, dat het altijd naar voren komt. Wij hebben een soort standaard; wij werken in ons systeem met allemaal smart phrases, welke dingen we allemaal willen bespreken. In onze standaardgespreksvelden zit dus een kopje “psychosociale anamnese”, een kopje “existentieel”, een kopje “levenseindebesluiten”. Dat betekent dat we al die punten… We hebben de somatische, psychische, sociale, existentiële en levenseindebesluiten. Dat zijn allemaal dingen die standaard voorgedrukt staan in ons standaard bespreekdomein. Dat betekent ook dat als de verpleegkundigen een intake doen, we verwachten dat ze ook alles bespreken. Als iemand voor het eerst een consult heeft en je begint gelijk in dat consult over levenseindebesluiten…

**I: Ja, dat is wel heftig.**

R: Ja, maar je kan soms wel bespreken van: goh, in dit soort gesprekken hebben we het vaak over dingen die belangrijk zijn en ook over zaken rondom het levenseinde, hebt u daar weleens over nagedacht, wat u daarmee zou willen? Je merkt vanzelf wel of patiënten daar helemaal door ontregeld raken of dat ze zeggen: ja, daar had ik toch eigenlijk wel een vraag over. En soms is het dan ook heel makkelijk, van: zal ik u er iets over uitleggen? Dan houd je het vrij open. En als de patiënt zegt, ja, misschien, dan leg ik de verschillen uit tussen euthanasie, palliatieve sedatie en doodgaan. We hebben ook altijd een kopje “toekomstscenario’s” wat we bespreken…

**I: Een ingewikkeld kopje lijkt me dat.**

R: Ja, dat is het ook. Ik vraag er ook altijd bij van: goh, zou u dat willen weten? Ik zeg altijd: zou u willen weten wat een aantal dingen kunnen zijn die in de toekomst zouden kunnen gebeuren bij het ziektebeeld dat u heeft? Sommige mensen zeggen: ja, ik wil heel graag weten wat me te wachten staat. Andere mensen zeggen: ja, misschien in grote lijnen een beetje. En andere mensen zeggen: ja, dat hoef ik echt niet te weten. Dan stel ik altijd wel de vervolgvraag, van: vindt u het goed dat ik wel uw huisarts informeer over welke toekomstscenario’s zouden kunnen ontstaan, want dan heeft de huisarts wat meer inzicht in dat wat zou kunnen gebeuren. Het vervelende is… Of “vervelende” of “mooie”, het is maar hoe je het bekijkt. Alles is tegenwoordig openbaar hè? Dus als ik een brief schrijf aan de huisarts, staat daar een kopje “toekomstscenario’s” in. Dus ik waarschuw patiënten tegenwoordig wel: u kunt mijn brief die ik aan de huisarts schrijf lezen, maar houdt er rekening mee dat ook dit onderdeel daarin staat, dus als u het niet wilt lezen, moet u het niet lezen, maar ik ga geen schaduwboekhouding bijhouden. Ik zeg het nu maar, want die brieven zijn best wel concreet of duidelijk of soms wel precair. Dus ik zeg het wel tegen patiënten, dat als ze het niet willen weten ze die brief maar even moeten overslaan, wat ik soms best wel lastig vind, maar dan denk ik, ik heb in ieder geval de patiënt dan geïnformeerd en wat zij dan met die informatie doen moeten ze dan zelf weten.

**I: Dan is het in ieder geval gezegd, toch?**

R: Ja. Ja. En dan kunnen ze uiteindelijk zelf beslissen of ze daar wel iets mee willen. Zeker als patiënten heel specifiek geen scenario’s willen weten; ja, dat is bij uitstek vaak iets wat de huisarts wel moet weten. Dan is het toch een beetje een soort patstelling. Patiënten vinden het eigenlijk prima, als je het zo uitlegt. Tenminste, ik heb nog nooit gehad dat iemand het niet goed vond. Ja.

**12.48**

**I: Wat je aangeeft; die aspecten komen eigenlijk altijd ter sprake, omdat jullie dus die lijn volgen. Die aspecten komen ter sprake. Is dat eigenlijk bij alle patiënten zo? Is dat bij alle patiënten hetzelfde?**

R: Ja, bij alle patiënten volgen we dezelfde lijn en verwachten we het. Het kan natuurlijk wel zo zijn dat op een gegeven moment de patiënt zegt: ja, maar hier wil ik het echt niet over hebben, dat levenseindebesluit. Of dat een gesprek heel lang gaat over het levenseindebesluit en dat dat kopje “toekomstscenario’s” nog niet besproken is. Wat we doen, is dat we zeggen: nog niet besproken in dit consult, graag in het volgende consult opnieuw bespreken. Maar het is wel één van onze standaarditems. Het wordt wel geacht dat je hem invult of dat je in ieder geval erbij zet waarom je hem dan niet hebt ingevuld op dat moment. Ja.

**I: Dat was eigenlijk mijn volgende vraag inderdaad, of die afspraken rondom die aspecten die dan besproken worden ook vastgelegd worden in een individueel zorgplan of zoiets. Een zorgplan is misschien niet het goede woord hoor.**

R: Het is dus wel vastgelegd of althans, het wordt door ons gedocumenteerd. Wij hebben niet een apart zorgplan. We maken wel een MDO van elke patiënt, waarbij we de patiënt bespreken met een pijnarts, met een palliatief arts, met de gespecialiseerd verpleegkundige, met een psycholoog, met onze spiritueel consulent. Daar bespreken we die patiënten. Bij elke patiënt, elke nieuwe patiënt op de poli en bij elke patiënt in de kliniek die meer dan twee keer gezien is, moeten we een MDO doen en dan staat dat weer als MDO Palliatieve Zorg gedocumenteerd, dus daar kan je het dan heel snel terugvinden. Maar we hebben niet een heel duidelijk kopje ACP of zo, advanced care plan, en dat je dan gelijk dat bij de patiënt terugvindt. Dus je moet wel enigszins weten waar je het moet vinden.

**I: Maar dat komt natuurlijk ook, denk ik, door jullie rol, toch? Doordat jullie een wat meer adviserende rol hebben, wat je ook al aangaf.**

R: Ja, we zijn in dit vak natuurlijk nooit hoofdbehandelaar. We worden nog weleens in consult gevraagd bij een patiënt die nog een maximaal beleid heeft, waar alles nog open staat. Dat zie je wel bij ons in de header gelijk ziekenhuis-breed. Dan is vaak wel de eerste vraag: goh, prima, wij komen in consult, maar willen jullie in ieder geval vast na gaan denken over het beleid wat bij deze patiënt genoteerd staat. Als jullie ons vragen omdat jullie verwachten dat deze meneer nu zo’n groot probleem heeft dat er geen behandelopties meer zijn; verwachten jullie wel dat deze man nog naar de ic moet? Meestal krijg je dan te horen: nee, nee, natuurlijk niet. Dan zeg ik: dan moet je er ook iets mee doen. Dan moet je het niet alleen aanpassen, maar er ook een gesprek over voeren. Ik blijf wel van mening dat het niet aan de palliatieve zorg is om een gesprek te voeren over doodgaan, maar dat dat vooral hoort bij de hoofdbehandelaar.

**I: Dus wat meer bij de centrale zorgverlener dan…**

R: Ja, op zijn minst ook een deel. Die mag best zeggen, ik ben er niet zo goed in, of, ik heb er weinig tijd voor, maar heel vaak zitten patiënten oneindig lang bij een bepaalde hoofdbehandelaar en het zou natuurlijk heel gek zijn als je op een gegeven moment zegt: sorry, maar ik heb nu niks meer te behandelen, dan stuur ik u nu naar het palliatieve team. Ik vind dat je als elke dokter; je bent niet voor niks hartspecialist of nierspecialist. Ook als je nieren of je hart of weet ik veel wat voor orgaan het niet meer zo goed doet, moet je daar op zijn minst ook iets van vinden. En dat je daar extra hulp bij nodig hebt in de vorm van een palliatief team is uiteraard prima, maar je kan het niet ontwijken, het gesprek, als zorgverlener. Dat vind ik althans. Ik heb wel het idee dat dat langzamerhand wel wat beter gaat en in ieder geval beter gedocumenteerd wordt. We zullen zien of het daadwerkelijk zo is. Ja.

**17.14**

**I: Als je het goed vindt wil ik een beetje inzoomen op communicatie.**

R: Ja.

**I: Communicatie is de basis voor goede zorg, zodat de patiënt goed geïnformeerd is en er sprake kan zijn van gezamenlijke besluitvorming. Zijn er patiënten bij wie jij de communicatie moeilijker vindt?**

R: Ik vind communicatie heel erg leuk en heel erg belangrijk en ook een enorme uitdaging, om dat goed te doen. Patiënten bij wie ik het moeilijk vind; dat zijn denk ik vooral de patiënten die de hele tijd zo gefocust zijn geweest op genezen, vaak in combinatie met een dokter die zo gefocust is op genezen, dat zowel patiënt, dokter als familie helemaal teleurgesteld is dat er gefaald is, in welke vorm dan ook. Die vinden dan vaak een gesprek met een palliatief team echt heel erg bedreigend of: nu is er iets helemaal misgegaan. Dat heeft denk ik ook een beetje te maken met het huidige: kanker is er om bestreden te worden, als je maar hard genoeg strijd dan is de wereld maakbaar. Die categorie patiënten vind ik altijd wel een uitdaging, om ergens een vinger tussen de deur te krijgen om te laten zien dat hoe graag je ook zou willen je soms te dealen hebt met iets dat groter is dan jijzelf. Of je je nou omdraait of niet, je zult er iets mee moeten. Dat is misschien niet datgene wat je wenst en misschien niet datgene wat je wil en misschien wel datgene waar je heel erg boos over wordt of verdrietig, hoe dan ook, maar uiteindelijk kan je het niet keren. Dat vind ik wel echt een uitdaging, om ook die groep patiënten… Soms helpt het wel om te vragen van: goh, zijn er ook dingen die u helemaal niet zou willen? Mensen weten heel vaak niet wat ze wel willen, maar je kan soms wel vragen: goh, ik merk dat u nog helemaal in het stuk zit van ‘gaan voor genezing’, dat u nog naar Amerika gaat. Als dat is wat bij u past, dan moet u die kant op gaan, maar stel nou dat het wonder niet gebeurt of stel nou dat het niet afloopt zoals u nu hoopt, zijn er dan nog dingen die u helemaal niet zou willen of scenario’s waarvan u denkt… De meeste mensen komen dan gelijk met het kasplantjesverhaal, van: ik wil nooit een kasplantje worden. “Ja, maar wat betekent dat dan voor u, hoe ziet dat kasplantje er dan uit, waar staat dat kasplantje dan, wie is dat dan precies?” Soms kan je patiënten nog wel verleiden om daar iets over te zeggen of te zeggen van, misschien is het goed om een soort noodplan te maken, van: wat moet u partner doen op het moment dat het niet gaat zoals gewenst. Probeer er één keer over na te denken, leg het in een laatje of leg het bij de huisarts, zodat iedereen in ieder geval weet wat ze zouden moeten doen als het niet gaat zoals u eigenlijk zou wensen. Dat vind ik altijd wel een boeiende uitdaging, om daar iets mee te doen. Ja.

**20.32**

**I: Ik zat even te denken, dat zou mijn volgende vraag zijn, van: in welk opzicht is de communicatie moeilijker bij deze mensen? Maar dat heeft eigenlijk meer met houding te maken, denk ik. Klopt dat?**

R: Ja, dat denk ik wel. Heel vaak zijn het mensen die heel erg boos zijn, mensen die de opmerking maken: nou heb ik mijn hele leven lang heel gezond geleefd en altijd gesport en nu krijg ik kanker en mijn buurman is veel te dik, die rookt als een ketter en die heeft helemaal niks. Dat is echt een soort onmacht of boosheid. En ook van: ja, maar kanker is toch te genezen, het is toch een chronische ziekte. Of van die mensen die heel internet napluizen en dan met hele bijzondere extreme dingen komen, wat in Nederland niet geregistreerd is, die dan vinden dat wij als dokters niet genoeg compassie hebben voor ons werk of die in allerlei alternatieve hoeken gaan zoeken en dan jou eigenlijk verwijten maken, waarbij ik ook weleens zeg van: ja, maar denk je nou echt dat ik stiekem iets in mijn kast heb staan waarmee ik jou zou kunnen helpen, maar dat ik jou dat stiekem niet geef? Is dat nou echt hoe jij hier tegenover me zit? Dan realiseren ze zich vaak ook wel dat dat misschien een beetje raar is, van: oh nee, natuurlijk niet. Dan zeg ik, ik wil wel hetzelfde als jij, ik zou namelijk ook willen dat je hier niet zat, dat wij dit gesprek nooit zouden hoeven te voeren, maar jammer genoeg is het wel zo. Hoe kan ik nou iets voor je betekenen op de weg die gaat komen? Ik vind het vooral belangrijk dat je aansluit bij datgene wat bij diegene past. Als iemand altijd de regie heeft gehouden, probeer dan mee te denken hoe iemand de regie kan blijven houden. Als iemand altijd op wereldreis is geweest en eigenlijk nog heel graag op reis wil; wat was het wat in die wereldreizen zo belangrijk was en kan dat ook op een andere manier? Kan die wereldreis dichter bij huis of kan het korter? Ja, ik denk dat je best wel met creatieve gedachtes een eind kan komen, maar dan moet je heel goed weten wat die onderliggende laag is. Dan moet je het wel vragen.

**I: Ik zat dus al meteen te denken: hoe kom je achter die onderliggende laag? Maar dat is dus gewoon door te vragen wat interesses zijn?**

R: Ja, of als mensen zeggen: ik had nog het plan om op wereldreis te gaan, dat zou ik dan en dan doen. Dan zeg je: goh, wat zou u dan het liefste willen, wat trekt u aan in zo’n wereldreis, is dat dan het avontuur of het weg zijn of het maken van een plan? Zo kwam ik er een keer bij iemand achter dat vooral het feit dat hij een plan maakte om ergens naartoe te gaan – hij hoefde dat plan helemaal niet uit te voeren – hem eigenlijk al heel veel rust gaf. Dus het feit dat hij eigenlijk in zijn hoofd op wereldreis ging was eigenlijk al veel boeiender dan het echt doen. Terwijl mijn collega dacht: die man wil een ticket gaan kopen naar India en dat kan toch helemaal niet.

**I: Het ging niet zozeer om het naar India gaan, maar het ging om…**

R: Als hij zich heel goed had gevoeld, had hij dat heel graag gewild. Daar was hij heel erg over aan het praten. Maar als je zou vragen, zou u nou echt daarnaartoe gaan, dan was het: dat is misschien toch niet zo verstandig. Ja. Eigenlijk het gewoon uitvragen. Daar kom je niet altijd in één gesprek achter, maar soms ken je patiënten wat langer en dan kan je erop terugkomen, van: goh, u zei eerder dat en dat, hoe denkt u daar nu over?

**24.07**

**I: Even denken hoor. Ik noem het steeds “houding”, ik weet niet of dat klopt hoor. Een manier om dus de communicatie bij de mensen bij wie jij het lastiger vindt… Dat is eigenlijk dus door het nog meer uit te vragen en je eigenlijk nog meer in te leven in die patiënt zelf en daar eigenlijk op in te haken. Klopt dat, wat ik zeg? Anders moet je het verbeteren hoor.**

R: Dat denk ik en ik denk dat het heel belangrijk is als dokter om je eigen mening een beetje weg te laten. Kijk, natuurlijk moet je binnen de… Je weet natuurlijk heel goed als dokter wat wel of niet kan en wat wel of niet zinvol of zinloos is. Dus ik ga mijn patiënten niet bewust een zinloze behandeling aanpraten, maar ik kan wel met patiënten bespreken wat de voor en tegens van een behandeling zijn. Als de patiënt zegt, voor mij is drie maanden extra tijd helemaal niet belangrijk, ik wil gewoon niet in het ziekenhuis komen; misschien vind ik als dokter drie maanden tijd heel erg belangrijk, dat maakt mij niet uit. Ik kan als dokter daar wel een andere mening over hebben, maar als deze patiënt zegt, ik weet dat ik niet beter word en die drie maanden extra vind ik te weinig om deze behandeling in te gaan, dan is dat voor mij prima, als die patiënt heel goed weet wat de voor en tegens zijn. Maar ik ga niet een patiënt ondersteunen om een eenvoudige levensreddende behandeling niet te doen. Dan zal ik wel goed proberen te horen van: wat is nou datgene wat de patiënt echt niet wil, waar komt dat nou door, heb ik het dan niet goed uitgelegd of moet iemand anders een keer praten? Ik probeer wel goed de drijfveren te achterhalen van iemand en niet zozeer mijn… Ja, je hebt natuurlijk altijd je eigen ideeën. Die kan je natuurlijk nooit helemaal uitschakelen. Maar ik probeer in ieder geval zo open minded mogelijk daarin mee te gaan om aan te sluiten bij de wensen en gedachten van een patiënt.

**I: In die lijn van gedachten; in hoeverre is er dan volgens jou sprake van gezamenlijke besluitvorming bij patiënten in de palliatieve fase?**

R: Dat blijf ik best een beetje lastig vinden. Het ligt er namelijk heel erg aan hoe je het brengt als dokter. Iemand komt bij jou… Dat heb ik ook misschien wel een beetje als oncoloog. Dan heb je heel veel patiënten in de palliatieve fase. Dan komt een patiënt bij jou, die is verwezen door de chirurg voor het bespreken van behandelopties bij een gemetastaseerd pancreascarcinoom. Dus die patiënt komt al met een intentie: ik ga behandeld worden door de oncoloog, want de chirurg heeft gezegd: de oncoloog heeft nog wel wat. Dus de patiënt komt met een gedachte. Dan kan ik als oncoloog heel simpel gaan uitleggen: nou, dit is de volgende behandeling, die zouden we kunnen doen, dit zijn de voor en dit zijn de tegens. Of je kan als oncoloog zeggen: goh, er zijn een aantal verschillende dingen die we zouden kunnen doen. Je kan kijken hoe het gaat en de ziekte zijn beloop laten hebben en u ondersteunen waar u klachten van krijgt, dat we niets doen aan de onderliggende ziekte en kijken: waar krijgt u last van? Krijgt u ergens last van, kunnen we die klachten zo goed mogelijk behandelen. We kunnen ook ziektegericht gaan behandelen, maar dat betekent dat u wel een intensievere behandeling krijgt met vaker in ieder geval bezoeken, enzovoort. Maar ik probeer zelf het niet-ziektegericht behandelen ook als een net zo serieuze behandeloptie mee te nemen voor de behandelingen waarbij dat reëel is. Maar als je als oncoloog begint met, nou, we kunnen chemotherapie geven enzovoort, en je als laatste zin zegt, oh ja, trouwens we kunnen ook niks doen, dan is dat natuurlijk geen gelijkwaardig alternatief. “Oh ja, we kunnen ook niks doen.” Zo wordt het nog heel vaak wel gebracht. Ja, dan vind ik dat geen goede gezamenlijke besluitvorming, want dan is het gewoon: wie gaat nou een oncoloog geloven als je eerst tien minuten uitleg hebt gegeven over alle behandelingen en dan in één zin zegt: we kunnen ook niks doen. Dat is dan toch geen gelijkwaardig alternatief. Dan moet je daar op zijn minst ook iets over uitleggen. Dus ik denk dat je best wel goed met patiënten aan gezamenlijke besluitvorming kan doen, maar dan moet er wel echt iets serieus te besluiten en te kiezen zijn en dan moet je wel weten van een patiënt wat hij of zij wenst, hoe hij of zij in het leven staat, wat diegene belangrijk vindt, heel veel thuis zijn of een ziekenhuisbezoek, zijn er nog doelen dichterbij of verder weg die iemand per se wil halen of in zijn gedachten heeft, wil iemand nog op reis en is dat dan reëler met of zonder mijn behandeling en is die reis belangrijker dan wel of niet behandelen? Daar moet je wel iets voor weten. Uiteindelijk vind ik het wel heel belangrijk dat het een gezamenlijk besluit is, dat niet de patiënt het idee krijgt van: oké, ik mocht kiezen, wel of niet behandelen, ik heb gekozen voor niet-behandelen. Dan krijgt iemand bijvoorbeeld drie weken later koorts op basis van een of andere infectie en dan krijgt diegene spijt, van: heb ik nou wel de juiste keuze gemaakt? Ik vind het wel heel erg dat je als dokter moet zeggen en het liefst ook moet uitspreken: oké, alle voor en tegens meegenomen besluiten wij nu gezamenlijk dat dit en dit het beste bij de lijn past die u volgt. Dat je heel erg duidelijk maakt dat jij mede dat besluit neemt en dat je dat echt gezamenlijk neemt, dat het niet is: u vraagt, wij draaien.

**I: Ja, dus dat je het nog even benadrukt.**

R: Ja, ik vind dat je dat echt moet uitspreken. De reden daarvan is dat ik heel vaak patiënten heb gehad; dan waren wij het erover eens na een heel lang traject waarin we elkaar zagen met alle mitsen en maren. Op een gegeven moment kom je op het punt van, nu hebben we geen ziektegerichte behandeling meer, en voor de patiënt is dat meestal oké. Dan bel ik een week of twee weken later nog eens op van, hoe is het nou allemaal gevallen en hoe gaat het thuis, en dan is er altijd nog weer een buurvrouw van een vriend of een nichtje van een neef, die dan nog met allerlei alternatieve opties komt, waarbij de patiënt dan toch nog even gaat vragen: het zal wel niet hoor, maar ik kreeg toch nog even… Daarom zeg ik ook weleens tegen patiënten: zeg maar dat ik besloten heb dat het echt niet meer kan. Soms vinden patiënten het makkelijker om te zeggen van: volgens de dokter kon het echt niet meer, want de lever was niet goed. Weet ik veel. Soms is het makkelijker om de dokter de schuld te geven dan dat ze zelf ergens nog… Patiënten hebben soms het gevoel dat ze zich moeten verdedigen.

**I: Dat ze zich moeten verantwoorden eigenlijk voor de keuze.**

R: Dat ze moeten verantwoorden voor de omgeving waarom ze iets niet meer doen. Dan zeg ik ook vaak echt letterlijk tegen hen: geef mij maar de schuld. Ik heb besloten dat dit niet meer haalbaar is en wij weten samen het echte verhaal waarom het zo is, maar als je er last van hebt, dan zeg maar dat ik dat gezegd heb, dat de dokter heeft gezegd dat het echt niet anders komt. Voor sommige patiënten is dat heel fijn.

**I: Misschien ook wel geruststellend, voor patiënten.**

R: Dan ligt het buiten henzelf, dan hoeven ze het minder te verdedigen.

**I: Dat lijkt me ook ingewikkeld, met naasten bijvoorbeeld.**

R: Ja. De directe naasten die vaak meekomen op de poli, die zitten in dat gesprek en dat is natuurlijk nooit een eenmalig gesprek, maar die zie je dus verschillende keren, soms jaren, waardoor dat gesprek… Op een gegeven moment ben je bij datgene wat je al honderd keer van tevoren een stukje hebt besproken. Dan is soms een slechtnieuwsgesprek heel kort. “Het is niet goed hè?”. “Nee, het is niet goed.” “Ja, zullen we dan maar afscheid nemen?” Soms is dat het enige slechtnieuwsgesprek wat dan volgt, omdat dat al zo vaak tevoren is besproken. Maar af en toe heb je een familielid dat in het buitenland woont en die dan een keertje meekomt naar een gesprek en dan helemaal over de flos gaat van: waarom is het dan nu opeens…. Nee, het is niet opeens, maar je hebt de afgelopen twee jaar eigenlijk gemist omdat je op afstand was. Dan krijg je van: ja, maar mijn zoon wil dat ik nu een tweede mening aanvraag. Dan zeg ik, dat vind ik prima, dat mag altijd, maar dan wil ik eerst dat uw zoon komt luisteren naar de eerste mening. Als u dan samen hier op de poli bent en u hebt samen een eerste mening gehad dan schrijf ik per direct na het consult een brief voor een tweede mening, maar ik wil wel graag dat uw zoon hier komt voor een eerste mening. Dan hoeft het eigenlijk nooit meer. Heel soms nog weleens een keer, maar meestal is het angst en paniek. Het helpt enorm dat te bespreken. Ja.

**33.25**

**I: Ik moet denk ik eventjes een beetje gaan opschieten. Hoelang zijn we… Ja, een half uur.**

R: Ja, ik praat altijd heel veel.

**I: Even kijken hoor. Ik heb hier een aantal belangrijke thema’s in de zorg voor patiënten in de palliatieve fase. Levensbeschouwing en culturele achtergrond. Niet-behandelafspraken. Ziekenhuisopnames. Plaats van zorg en sterven. Crisissituaties. De wettelijke vertegenwoordiging in situaties van acute verslechtering. Wilsbekwaamheid en levenseindebeslissingen. Mijn vraag is in hoeverre deze onderwerpen aan bod komen tijdens gesprekken met patiënten.**

R: Levensbeschouwing vragen we wel bij het existentiële eigenlijk altijd na, in ons kopje afspraken. Nou is het hier ook heel erg uni cultureel vergeleken met mijn vorige ziekenhuis. Bijna iedereen komt hier uit Brabant en is katholiek.

**I: Oh ja?**

R: Er zijn relatief weinig mensen van een andere origine. Het komt wel aan de orde, maar het is hier gewoon niet zo divers. Niet-behandelafspraken bespreken we vaak wel rondom het kopje levenseindebesluiten, maar dat bespreek ik veel meer als oncoloog zijnde dan als consulent van de palliatieve zorg, omdat het echt wel iets is van de hoofdbehandelaar. Wel of niet dialyseren; dat hoort trouwens bij de nefroloog, om daar definitief iets over de bespreken, maar je kan als consulent palliatieve zorg bij een nefrologische patiënt best bespreken van: goh, eens komt het moment dat uw nefroloog met u gaat bespreken of u nog verder wilt dialyseren. Hebt u daar zelf weleens over nagedacht? Uiteindelijk is degene die dat definitief maakt in ons systeem de hoofdbehandelaar. Ziekenhuisopnames… We vragen wel bij patiënten die bijvoorbeeld opgenomen zijn vanwege bijvoorbeeld een ileus of voor een ander probleem…

**I: Wat is een ileus?**

R: Een ileus is een darmprobleem, waarbij de darmen niet meer goed op gang komen. Dat is een veel voorkomende complicatie bij allerlei patiënten met kanker. Daar vragen we vaak wel van: goh, u bent nu wel een beetje opgeknapt, maar wat zullen we nou gaan doen als dit nog een keer ontstaat? Wilt u dan weer opnieuw opgenomen worden of kan dat dan thuis? Dus dat bespreken we vaak wel aan de hand van een opname. De plaats van zorg en sterven. Ook meestal rondom die levenseindebesluiten, van: wat zou u willen, heeft u weleens nagedacht over een hospice? Maar we zijn er meestal nog een stukje voor. Op de afdeling wordt dat ook vaak door de verpleging besproken, van: wat is haalbaar, hoeveel zorg is er… Wij zullen dat ook wel bespreken in onze consulten. Crisissituaties bespreken we eigenlijk ook bij het onderdeel “toekomstscenario’s”, met name als je bijvoorbeeld… Sommige patiënten met hoofd-hals kanker hebben een groot risico op een grote bloeding, waarbij ze acuut kunnen komen te overlijden. Dat is een hele vervelende en nare complicatie, maar wat ook heel erg beangstigend kan zijn voor naasten.

**I: Voor de mensen eromheen…**

R: Een longbloeding bijvoorbeeld. Dan is het heel fijn dat je weet wat je kan doen, hoe het gaat. Het verhaal horen is niet prettig, maar het verhaal aantreffen en niet weten wat het is, is vaak nog onprettiger. Dus dan bespreken we wat er zou kunnen gebeuren, wat je dan zou kunnen doen. Dan krijgen mensen vaak een neusspray mee met midazolam, zodat je ook in slaap valt. Als je ernaast staat is het fijn dat je weet wat het is en dat je iets kan doen. Dat bespreken we dan ook. Die wettelijke vertegenwoordiging in een situatie van acute verslechtering bespreken we eigenlijk nooit van tevoren.

**I: Nou ja, misschien is dat ook niet nodig altijd.**

R: Nee. We hebben weleens op de afdeling dat er wat onduidelijkheid over is. Dan is iemand gescheiden, dan is er nog een ex-partner en een ouder, dan moeten we even nazoeken wie dan officieel de wettelijke vertegenwoordiger is en hoe dat dan werkt, maar ik bespreek het nooit van tevoren. We hebben wel een lijst met contactpersonen, wie dan eerste contactpersoon is. Dat is alleen degene aan wie informatie gegeven mag worden.

**I: Ja, dat zal vaak dezelfde persoon zijn toch?**

R: Ja, vaak wel inderdaad. Wilsonbekwaamheid en levenseindebeslissingen; ja, die levenseindebeslissing bespreken we eigenlijk altijd. Die wilsonbekwaamheid niet altijd, maar we bespreken wel bij de levenseindebeslissingen – zeker als patiënten het er helemaal niet over wil hebben – van: goh, maar het is wel fijn dat iemand weet wat u in ieder geval niet zou willen op het moment dat u het zelf niet meer kan uiten. Dus die wilsonbekwaamheid; ik weet niet of we dat altijd zo letterlijk bespreken van: wat nou als u het niet meer zelf zou kunnen beslissen. Soms als het aan de orde komt of als het op dat moment al heel erg aan de orde is. Bij patiënten die al een beetje in de war zijn of patiënten die een grote tumor hebben in hun hoofd waardoor ze wisselend alert zijn. Ik denk dat dat één van de dingen is die je het slechtste terug kan vinden in onze dossiervoering.

**39.10**

**I: Als je het goed vindt; ik heb nog een topic en dat gaat over beperkte gezondheidsvaardigheden. Daar ga ik dan even vlug doorheen. Dan zitten we ook nog een beetje in de tijd. Ja, de eerste vraag is of je weleens gehoord hebt van het begrip beperkte gezondheidsvaardigheden.**

R: Nee, eigenlijk pas sinds het onderzoek.

**I: Pas sinds het onderzoek hè? Ja, dat had je volgens mij al ooit verteld inderdaad. Wat betekent het volgens jou?**

R: Ik denk dat het betekent de mate waarin je in staat bent om zelf regie te voeren over alles wat met je eigen gezondheid te maken heeft. Zelf de regie voeren, filteren of informatie voor jou van belang is, ja dan wel nee, besluiten of je iets wel of niet wil, maar ook op de juiste afspraak op het juiste moment verschijnen, de logistiek van alle afspraken kunnen bevatten en coördineren. Ja, eigenlijk op meerdere vlakken, op inhoud en op logistiek, in staat zijn om ook de vragen die jij hebt goed te kunnen communiceren naar jouw zorgverleners.

**I: Ja, precies, je zegt: niet alleen puur functioneel, maar ook interactief en kritisch.**

R: Ja. Ja.

**I: Hoe vaak heb jij zelf te maken met patiënten met beperkte gezondheidsvaardigheden?**

R: Ik denk heel vaak. Ik denk dat… Het ligt er maar aan. Ik denk dat er bijna geen enkele patiënt is die zich in een emotioneel slechtnieuwsgesprek helemaal, honderd procent gezondheidsvaardig voelt. Ik denk dat iemand die hoog opgeleid is prima de afspraken kan coördineren, vragen kan bedenken, op het juiste moment op het juiste tijdstip komt en in principe in staat is om goed zijn klachten kan verwoorden, maar op het moment dat er een rotdiagnose komt uit een CT-scan of iets heel erg onverwachts, dan kan ook diegene acuut beperkt gezondheidsvaardig worden, dus dat de emotie die bij palliatieve zorg speelt of bij slechtnieuwsgesprekken iedereen eigenlijk wel een beetje beperkt gezondheidsvaardig zou kunnen maken en dat mensen die bijvoorbeeld ook nog eens slecht kunnen lezen of rekenen of wat chaotisch zijn nog sneller de weg kwijtraken. Als ik zie wat al onze patiënten moeten doen, aan afspraken, aanmelden bij zuil 1, dan bloedprikken bij balie 2 en dan controles bij balie 3. Dan door de poli, de juiste nummertjes… Ja, je vraagt nogal wat van patiënten. Dan gaat het nog niet eens over de inhoud. Dus ik denk dat iedereen in principe daar wel een beetje last van zou kunnen hebben. Ja.

**42.08**

**I: Dus wat je aangeeft: die mensen met een lage health literacy, wat meer gericht op lezen en schrijven en zo, die hebben daar nog meer last van.**

R: Ja. Ik denk dat die daar nog meer last van hebben, omdat die ook al last hebben van de hele logistieke rompslomp.

**I: Ja, dat kost al heel veel energie.**

R: Ja, moet je dan al die bordjes lezen, al die routebewegwijzering. Waarom moet je de ene keer op de route letten en de andere keer op het nummer? Waarom moet je je de ene keer elektronisch aanmelden en de volgende keer bij een balie? Ja, die zijn al de weg kwijt voordat ze bij een dokter zijn. Ja.

**I: En in gesprekken met bijvoorbeeld zo’n soort patiënten, hoe herken jij dan die beperkte gezondheidsvaardigheden?**

R: Ik probeer altijd patiënten in ieder geval zelf te laten uitleggen in hun eigen woorden wat ze hebben, wat ze tot nu toe al weten en wat ze al gehoord hebben. Dan heb je in ieder geval enig zicht op welke woorden iemand gebruikt, welke informatie iemand heeft gekregen. Iemand komt van de chirurg bijvoorbeeld. “Wat heeft de chirurg al met u besproken, wat was de belangrijkste reden om nu hier te komen, is het u duidelijk wat het doel van dit gesprek is?” Sommige patiënten raken daar al de klus kwijt. Dan begin ik daar maar eerst met uitleggen wie ik ben, wat een oncoloog doet en wat ze van mij mogen verwachten in dat gesprek. Ik ga niet gelijk aan het begin vragen: goh, wat is uw opleidingsniveau? Dat hoeft helemaal niet gerelateerd te zijn aan die gezondheidsvaardigheden. Dat kan natuurlijk wel, maar dat komt dan meer bij het stukje psychosociale anamnese. Ja.

**I: Wat is volgens jou nodig om… Ik moet eerst vragen misschien: ervaar jij ook weleens barrières met patiënten met beperkte gezondheidsvaardigheden?**

R: Ja. Nou, niet zozeer barrières, maar dat je er achteraf achter komt dat het toch nog niet duidelijk genoeg is, dat ze dan bij elk nieuw probleem weer helemaal de weg kwijtraken. Met een patiënt die om de haverklap hierheen belde had ik uiteindelijk afgesproken… We gaan bijna nooit op huisbezoek als palliatief team, maar we dachten, we maken bij deze patiënt een uitzondering. Dan gaan we op huisbezoek met de huisarts, zodat we al die communicatieve rompslomp en verschillende ideeën…

**I: Dan heb je dat in ieder geval niet.**

R: Dan gaan we aan de keukentafel zitten met patiënt en partner en de huisarts en de HAIO (huisarts in opleiding) en ik en dan gaan we alles bespreken, hoe het dan moet. Dat werkt dan kortdurend en dan gebeurt er iets nieuws en dan zijn ze weer helemaal de kluts kwijt.

**I: Dan kun je eigenlijk weer bijna van voren af aan beginnen.**

R: Ja, dan kun je van voren af aan beginnen. Is dat dan een barrière of niet? Ja, dat is wel een barrière. Zij hebben toch hele beperkte gezondheidsvaardigheden en op het moment dat er iets dan niet helemaal gaat zoals gedacht of er komt een nieuw event bij, dan raken ze weer zo ontregeld dat eigenlijk alles wat daarvoor besproken is vergeten is. Nu gaat het beter. Nu zetten we er gewoon meer zorg op, meer verpleegkundigen, dat die gewoon altijd komen en niet alleen maar zo nodig. Ja, dat vind ik af en toe wel lastig. Dan denk je: ik heb echt zo erg mijn best gedaan om dat allemaal in Jip en Janneketaal uit te leggen, met iedereen die erbij was. Maar je loopt het huis uit en je denkt al: als dat maar goed gaat.

**45.44**

**I: Mijn laatste vraag is: wat zou nodig zijn om de zorg beter te kunnen laten aansluiten bij dat soort patiënten?**

R: Ik denk dat eigenlijk een soort van casemanager of verpleegkundige die laagdrempelig bereikbaar is… Eigenlijk hebben we dat team al wel, een verpleegkundig specialist palliatieve zorg voor in de thuissituatie; ik denk dat dat een heel geschikt iemand is om ook juist… Ja, het zou ideaal zijn, denk ik, als die verpleegkundige ook een keer bij een gesprek is met een dokter. Dat kan ook natuurlijk prima de huisarts zijn. Dat hoeft niet per se de specialist te zijn of het palliatieve team. Maar dat die verpleegkundige een hele belangrijke schakel is en dat die laagdrempelig en snel wordt ingeschakeld om juist al dit soort vragen te bespreken. Ja.

**I: Heb jij verder nog wat toe te voegen hieraan of te vragen eventueel?**

R: Nee. Ik ben benieuwd wat er uit jullie onderzoek gaat komen.

**[48.02 – einde opname]**
